# Upregulation of CRABP2 by TET1-mediated DNA hydroxymethylation attenuates mitochondrial apoptosis and promotes oxaliplatin resistance in gastric cancer

**DOI:** 10.1038/s41419-022-05299-2

**Published:** 2022-10-04

**Authors:** Xiaolong Tang, Yahang Liang, Guorui Sun, Qingsi He, Zhenyu Hou, Xingzhi Jiang, Peng Gao, Hui Qu

**Affiliations:** 1 Department of General Surgery, Qilu Hospital of Shandong University, Jinan, 250012 China; 2 Department of Pathology, Qilu Hospital of Shandong University, Jinan, 250012 China

**Keywords:** Gastric cancer, Cancer prevention

## Abstract

Oxaliplatin is the main chemotherapy drug for gastric cancer (GC), but quite a few patients are resistant to oxaliplatin, which contributes to the poor prognosis of GC patients. There is therefore an urgent need to identify potential targets for reversing chemotherapy resistance in GC patients. In this study, we analyzed the tumor samples of GC patients who received neoadjuvant chemotherapy based on oxaliplatin through quantitative proteomics and identified the potential chemoresistance-related protein cellular retinoic acid binding protein 2 (CRABP2). CRABP2 was significantly upregulated in the tumor tissues of chemoresistant GC patients and was closely related to prognosis. The results of cell function experiments showed that CRABP2 can promote the oxaliplatin resistance of GC cells in vitro. Coimmunoprecipitation and GST pulldown assays showed that CRAPB2 expedited the binding of BAX and PARKIN in GC cells and facilitated the ubiquitination-mediated degradation of BAX. Furthermore, both the in vitro assay and cell-derived xenograft (CDX) in vivo model verified that CRABP2 promoted oxaliplatin resistance by inhibiting BAX-dependent cell apoptosis. Further experiments proved that the abnormally high expression of CRABP2 in oxaliplatin-resistant GC cells was affected by TET1-mediated DNA hydroxymethylation. The patient-derived xenograft (PDX) model suggested that interference with CRABP2 reversed oxaliplatin resistance in GC in vivo. In conclusion, the results of our study show that CRABP2 was a key molecule in oxaliplatin resistance regulation and could be a new target for reversing the chemoresistance of GC.

## Introduction

Gastric cancer (GC) is one of the most frequent cancer types and the second most common cause of cancer-related deaths in the world. The incidence of GC is particularly high in east Asia, eastern and southern Europe, and parts of central and south America [1]. Although surgery is the primary treatment for GC, a considerable number of patients are diagnosed at advanced stages, with a 5-year survival rate of <50% [2]. Multimodality strategies are being used to improve survival in GC patients. Currently, chemotherapy is routinely used for the management of patients with advanced-stage GC. Among the various chemotherapy regimens available for GC patients, oxaliplatin (OXA) is a fundamental drug [3]. OXA is a DNA alkylating agent that causes cell death by inducing endogenous apoptosis [4]. However, a considerable proportion of GC patients do not respond well to chemotherapy [5], and the molecular mechanism of OXA resistance is still unclear. Therefore, there is an urgent need to clarify the molecular mechanism of OXA resistance in GC patients.

Focusing on a cohort of GC patients who received neoadjuvant chemotherapy before surgery, we used quantitative proteomics and identified cellular retinoic acid binding protein 2 (CRABP2), which was significantly upregulated in chemotherapy-resistant GC tissues. CRABP2 was related to the poor prognosis of GC patients and promoted OXA resistance in GC cells. Moreover, CRABP2 promoted oxaliplatin resistance in vitro and in vivo. The coimmunoprecipitation and GST pulldown assays showed that CRAPB2 could directly bind to apoptosis regulator BAX (BAX) and E3 ubiquitin-protein ligase parkin (PARKIN) in GC cells and facilitated the ubiquitination degradation of BAX. Furthermore, CRABP2 promoted OXA resistance by inhibiting BAX-dependent cell apoptosis in vitro and in vivo, and the expression of CRABP2 was affected by TET1-mediated DNA hydroxymethylation. The patient-derived xenograft (PDX) model suggested that interference with CRABP2 reversed the OXA resistance of GC in vivo. Our study identified the important role of CRABP2 in OXA resistance and could be a potential target for reversing chemoresistance in GC patients.

## Results

### CRABP2 was upregulated in chemotherapy-resistant GC patients

**Fig. 1:**
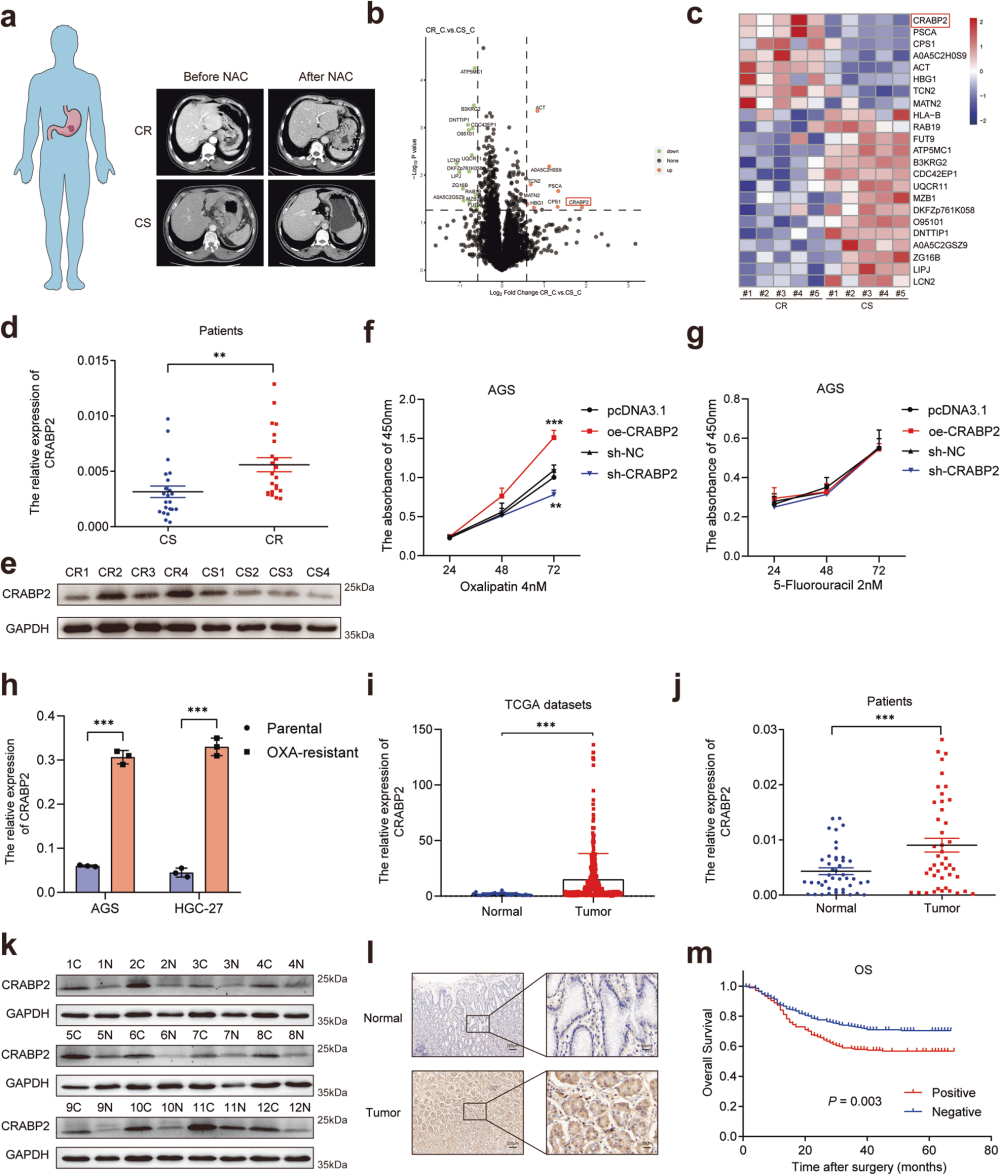
The expression of CRABP2 was upregulated in chemoresistant GC patients and predicted prognosis. **a** Typical image examples of CR and CS gastric cancer patients who received NAC. The volcano map (**b**) and heatmap (**c**) of the most upregulated/downregulated differentially expressed proteins in CR and CS tumor tissues. The qRT–PCR (**d**, *n* = 22) and western blotting (**e**, *n* = 4) results showed the expression of CRABP2 in tumor tissues of CR and CS patients. The CCK-8 results of AGS cells treated with OXA (**f**) or fluorouracil (**g**) after knockdown/overexpression of CRABP2. **h** The relative expression of CRABP2 in parental and OXA-resistant GC cells by qRT–PCR. **i** The expression of CRABP2 from the TCGA database (tumor = 335; normal = 26). Mann–Whitney U test (Wilcoxon rank sum test). The outlier values (median±2 IQR) and extreme values (median ± 3.5 IQR) were excluded. IQR: interquartile range. The expression of CRABP2 in tumor tissues and adjacent normal tissues of GC patients by qRT–PCR (**j**, *n* = 44) and western blotting (**k**, *n* = 12). **l** Typical IHC images of CRABP2-positive and CRABP2-negative tumor tissues. **m** The correlation curves between CRABP2 expression and OS of GC patients (*n* = 488). (CR chemotherapy resistance, CS chemotherapy sensitivity, OXA oxaliplatin, C tumor tissue, N normal tissues, IHC immunohistochemistry, OS overall survival). *P < 0.05, **P < 0.01, ***P < 0.001.

To search for chemotherapy resistance-related proteins in GC patients, we first obtained tumor tissue samples from GC patients after neoadjuvant chemotherapy (NAC) and divided the patients into a chemotherapy-sensitive (CS) group and a chemotherapy-resistant (CR) group according to the RECIST 1.1 criteria (Fig. 1a, Supplementary Table 1). For two groups of different tumor tissues (CS group, *n* = 5; CR group, *n* = 5), we carried out tandem mass tags (TMTs) based on quantitative proteomics (Supplementary Table 2). Then, we selected proteins by *P* < 0.05 and | log_2_ fold change (FC) | > 0.58 (Fig. 1b). Through the above methods, we identified 23 protein molecules, with 8 molecules upregulated and 15 molecules downregulated (Fig. 1c). Among them, we selected cellular retinoic acid binding protein 2 (CRABP2), whose expression was most upregulated (log_2_ FC = 1.89). Moreover, the expression levels of CRABP2 were significantly upregulated in the tissues of CR patients (Fig. 1d, e). As the patients enrolled in this study received the SOX (oxaliplatin plus fluorouracil) regimen, to specify which drug CRABP2 was mainly affected, we overexpressed CRABP2 and conducted a CCK-8 assay. The results showed that overexpression of CRABP2 promoted the resistance of cells to oxaliplatin (Fig. 1f) but not to fluorouracil (Fig. 1g). To further investigate the mechanism by which CRABP2 promotes oxaliplatin resistance, we obtained oxaliplatin-resistant AGS and HGC-27 cell lines, named AGS-OXA and HGC-27-OXA, respectively (Supplementary Fig. 1). The qRT–PCR results showed that the expression of CRABP2 in oxaliplatin-resistant cells was significantly higher than that in the corresponding parental cells (*P* < 0.001, Fig. 1h).

### The expression of CRABP2 predicted the overall survival of GC patients

To verify the expression of CRABP2 in GC tissues, we downloaded the expression data of GC tumors (*n* = 335) and normal tissues (*n* = 26) from The Cancer Genome Atlas (TCGA) database. The data analysis results showed that the expression of CRABP2 in GC tumor tissues was significantly upregulated (*P* < 0.001, Fig. 1i). To further verify this in clinical samples, we performed qRT–PCR in 44 pairs of GC tumor and normal tissues (Fig. 1j) and western blotting in 12 pairs of GC and normal tissues (Fig. 1k). The results showed that CRABP2 expression in GC tissues was significantly increased at both the protein and mRNA levels (*P* < 0.001). In addition, we performed immunohistochemistry (IHC) in 488 pairs of GC tumors and adjacent normal tissues (Fig. 1l). Correlation analysis showed that CRABP2 expression was related to tumor diameter, pT stage, pN stage, and pTNM stage (*P* < 0.05, Table 1). Univariate analysis showed that tumor diameter, surgical type, tumor differentiation, pT stage, pN stage, pTNM stage, lymphovascular invasion, and CRABP2 expression were associated with OS in GC patients (all *P* < 0.05, Fig. 1m, Table 2, and Supplementary Fig. 2). Multivariate analysis showed that pTNM stage, lymphovascular invasion, and CRABP2 expression were independent prognostic factors in GC patients (all *P* < 0.05, Table 2).

**Table 1:** Correlation between CRABP2 expression and the clinicopathological characteristics of GC patients.

Variables	No. of patients	CRABP2 expression		χ^2^	*P* value
		Positive	Negative		
Age (year)				0.00	0.949
≤60	208	89 (42.79)	119 (42.5)		
>60	280	119 (57.21)	161 (57.5)		
Sex				0.03	0.853
Male	397	170 (81.73)	227 (81.07)		
Female	91	38 (18.27)	53 (18.93)		
Tumor diameter (cm)				12.97	**0.000**
≤5	349	131 (62.98)	218 (77.86)		
>5	139	77 (37.02)	62 (22.14)		
Surgical type				1.42	0.233
Subtotal gastrectomy	381	157 (75.48)	224 (80)		
Total gastrectomy	107	51 (24.52)	56 (20)		
Tumor differentiation				3.44	0.064
Poor	272	126 (60.58)	146 (52.14)		
Well/Moderate	216	82 (39.42)	134 (47.86)		
pT stage				4.15	**0.042**
T1-3	263	101 (48.56)	162 (57.86)		
T4	225	107 (51.44)	118 (42.14)		
pN stage				7.07	**0.008**
N0	224	81 (38.94)	143 (51.07)		
N+	264	127 (61.06)	137 (48.93)		
pTNM stage				10.61	**0.001**
I–II	276	100 (48.08)	176 (62.86)		
III	212	108 (51.92)	104 (37.14)		
Lymphovascular invasion				2.94	0.086
Present	93	47 (22.6)	46 (16.43)		
Absent	395	161 (77.4)	234 (83.57)		

All *P* values < 0.05 are indicated in bold print.

**Table 2:** Univariate and multivariate analysis of factors associated with overall survival in GC patients.

	Overall survival	
	HR (95% CI)	*P* value
Univariate analysis		
Age (≤60 years vs. >60 years)	1.32 (0.97–1.80)	0.082
Sex (male vs. female)	0.72 (0.47–1.10)	0.130
Tumor diameter (≤5 cm vs. >5 cm)	2.31 (1.71–3.12)	**<0.001**
Surgical type (subtotal gastrectomy vs. total gastrectomy)	2.07 (1.50–2.85)	**<0.001**
Tumor differentiation (poor vs. well/moderate)	0.44 (0.31–0.61)	**<0.001**
pT stage (T1–3 vs. T4)	3.39 (2.44–4.69)	**<0.001**
pN stage (N0 vs. N+)	7.15 (4.64–11.02)	**<0.001**
pTNM stage (I–II vs. III)	7.75 (5.31–11.30)	**<0.001**
Lymphovascular invasion (present vs. absent)	0.43 (0.31–0.60)	**<0.001**
CRABP2 expression (negative vs. positive)	1.57 (1.16–2.11)	**0.003**
Multivariate analysis		
Age (≤60 years vs. >60 years)	1.24 (0.9–1.7)	0.190
Sex (male vs. female)	0.66 (0.43–1.02)	0.059
Tumor diameter (≤5 cm vs. >5 cm)	1.11 (0.79–1.55)	0.545
Surgical type (subtotal gastrectomy vs. total gastrectomy)	1.23 (0.9–1.68)	0.198
Tumor differentiation (poor vs. well/moderate)	0.9 (0.63–1.28)	0.545
pT stage (T1–3 vs. T4)	1.02 (0.68–1.52)	0.936
pN stage (N0 vs. N+)	1.98 (0.94–4.19)	0.073
pTNM stage (I–II vs. III)	3.71 (1.77–7.8)	**0.001**
Lymphovascular invasion (present vs. absent)	0.72 (0.51–1.01)	0.054
CRABP2 expression (negative vs. positive)	1.41 (1.01–1.98)	**0.043**

All *P* values < 0.05 are indicated in bold print.

### CRABP2 promoted the oxaliplatin resistance of GC cells

**Fig. 2:**
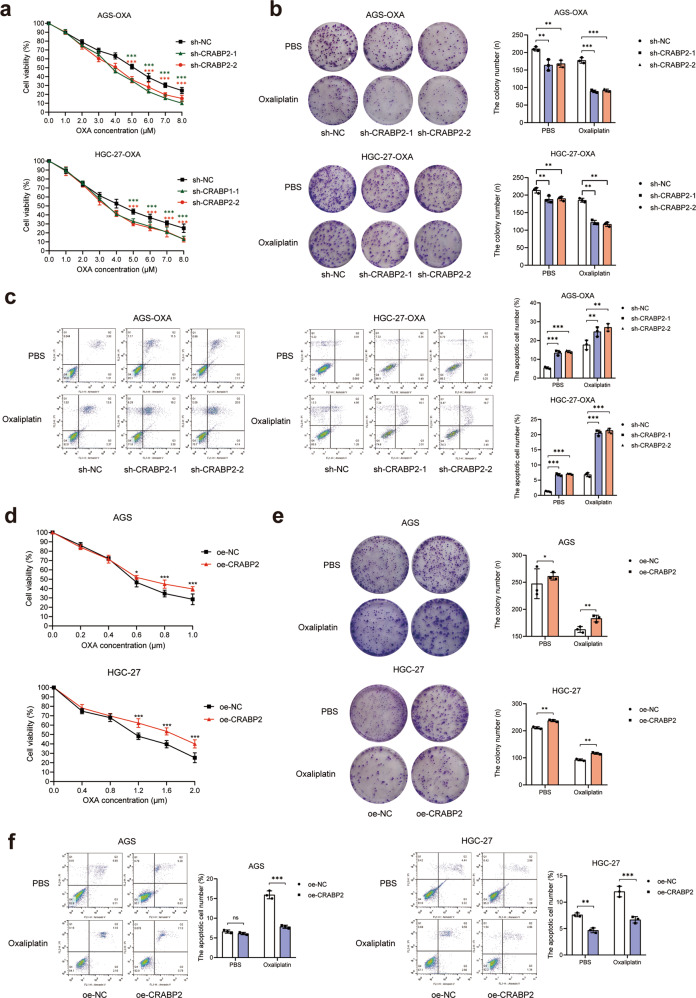
CRABP2 promoted the oxaliplatin resistance of GC cells. **a** After knocking down CRABP2, the viability of AGS-OXA and HGC-27-OXA cells was determined under different concentrations of OXA. **b** After knocking down CRABP2, the colony forming ability of AGS-OXA and HGC-27-OXA cells was determined in the absence or presence of OXA (concentration: 2.0 μM). **c** The cell apoptosis results of AGS-OXA and HGC-27-OXA cells after knocking down CRABP2 in the presence or absence of OXA (concentration: 2.0 μM) by flow cytometry. **d** In the presence of different concentrations of OXA, AGS and HGC-27 cells overexpressing CRABP2 were determined by cell viability assay. **e** In the presence or absence of OXA (concentration: AGS 0.2 μM, HGC-27 0.4 μM), AGS and HGC-27 cells overexpressing CRABP2 were determined by the colony forming assay. **f** In the absence or presence of OXA (concentration: AGS 0.2 μM, HGC-27 0.4 μM), the cell apoptosis results of AGS and HGC-27 cells overexpressing CRABP2 were determined by flow cytometry. All experiments were performed in three replicates. The data are presented as the mean ± SD. *P < 0.05, **P < 0.01, ***P < 0.001.

To confirm the effects of CRABP2 on the chemotherapy resistance of GC cells, we constructed stable knockdown AGS-OXA and HGC-27-OXA cell lines with lentivirus-carrying sh-RNA targeting CRABP2 (named sh-CRABP2) and stable overexpression AGS and HGC-27 cell lines (named oe-CRABP2). Cell functional experiments showed that after knockdown of CRABP2 and addition of different concentrations of oxaliplatin for 48 h, the cell viability of AGS-OXA and HGC-27-OXA cells decreased significantly (*P* < 0.001, Fig. 2a). Then, CRABP2 was knocked down in the two oxaliplatin-resistant cell lines with or without oxaliplatin, and the results showed that the colony-forming ability of the cells decreased significantly (*P* < 0.01, Fig. 2b) and that cell apoptosis increased significantly (*P* < 0.001, Fig. 2c). Moreover, the overexpression of CRABP2 in AGS and HGC-27 cells showed the opposite results in the presence or absence of oxaliplatin (Fig. 2d–f). We further performed Transwell and wound healing assays to investigate the migration and invasion ability of CRABP2 in GC cells. However, after interference with CRABP2, the results showed that the migration and invasion capabilities of AGS and HGC-27 cells did not change significantly (Supplementary Fig. 3). To ensure that cellular features of OXA-resistant GC cells did not change during the oxaliplatin induction procedure, we performed cell functional experiments in parental AGS and HGC-27 cells (Supplementary Fig. 4). These results suggested that CRABP2 can promote proliferation and inhibit apoptosis of GC cells in the presence or absence of oxaliplatin.

### CRABP2 expedited the binding of BAX and PARKIN in GC cells

**Fig. 3:**
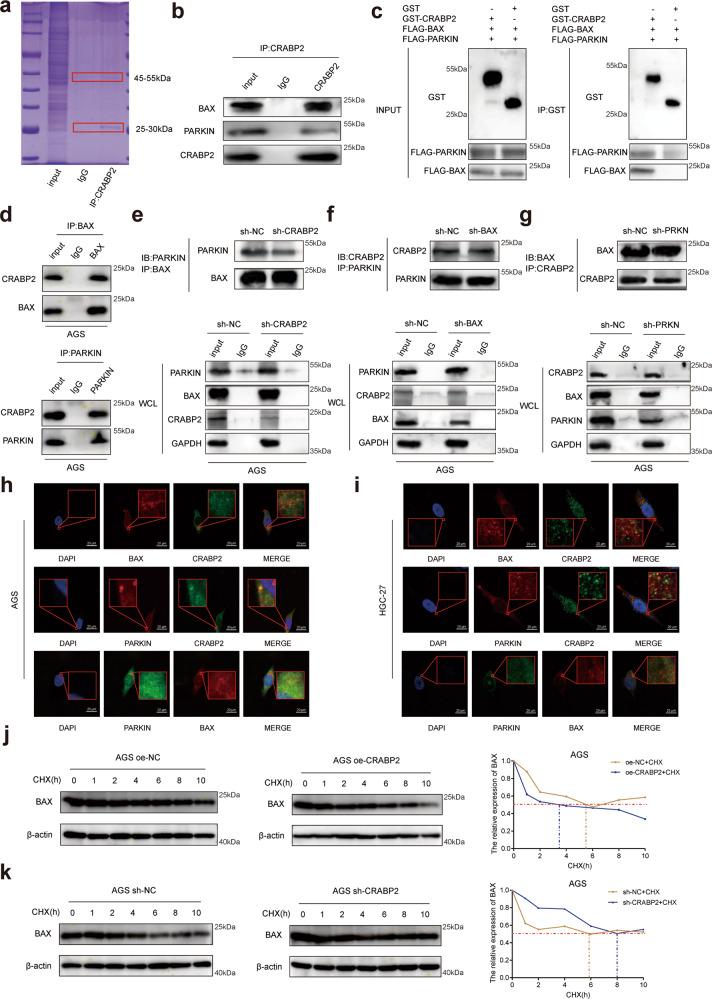
CRABP2 expedited the binding ability of BAX and PARKIN in GC. **a** Total proteins from Flag-CRABP2 plasmid-transfected AGS cells were separated via SDS–PAGE. PARKIN and BAX were identified by LC/LC–MS in the CRABP2 protein complex. **b** Mutual interactions of BAX/PARKIN and Flag-CRABP2 were verified by the Co-IP assay. **c** The BAX and PARKIN proteins were pulled down by GST-CRABP2 fusion protein-bound beads by SDS–PAGE analysis. **d** The interaction between BAX and CRABP2 and between PARKIN and CRABP2 was verified by co-IP assay and western blotting. **e** After interference with CRABP2 in AGS cells, the binding ability of BAX to PARKIN decreased. **f** After BAX interference in AGS cells, the binding ability of CRABP2 to PARKIN was not affected. **g** After interference with PARKIN in AGS cells, the binding ability of CRABP2 to PARKIN was not affected. The immunofluorescence assay showed the subcellular localizations (red frame) of CRABP2/BAX/PARKIN in AGS (**h**) and HGC-27 (**i**) cells. AGS cells overexpressing (**j**) or knocking down (**k**) CRABP2 were treated with cycloheximide (CHX) for 0, 2, 4, 6, 8, or 10 h. The expression of Bax was detected by western blotting. All experiments were performed in three replicates. *P < 0.05, **P < 0.01, ***P < 0.001. (IB immunoblotting, IP immunoprecipitation, WCL whole cell lysates).

To explore the molecular mechanisms of CRABP2 in regulating chemoresistance in GC cells, we conducted Co-IP experiments and analyzed the interacting proteins by mass spectrometry (Fig. 3a, Supplementary Table 3). The results of the Co-IP assay and western blotting verified that CRABP2 interacted with BAX and PARKIN (Fig. 3b). Then, we employed a GST pull-down assay to study the mutual relationship. When both proteins were purified, BAX and PARKIN proteins were pulled down by GST-CRABP2 fusion protein-bound beads but not with control beads by SDS–PAGE analysis, indicating the physical interaction of CRABP2 and BAX/PARKIN (Fig. 3c). These findings indicated that CRABP2 can directly interact with BAX and PARKIN. Moreover, we performed co-IP assays and western blotting to verify the interaction between BAX and CRABP2 and between PARKIN and CRABP2 (Fig. 3d). To further confirm the interaction, we interfered with the expression of CRABP2 in AGS cells, and the binding ability of BAX to PARKIN decreased (Fig. 3e). However, the binding ability of CRABP2 to PARKIN in AGS cells was not affected by the expression of BAX (Fig. 3f), and the binding ability of CRABP2 to BAX in AGS cells was not affected by the expression of PARKIN (Fig. 3g). Furthermore, immunofluorescence experiments showed the common subcellular locations of CRABP2/BAX/PARKIN proteins in both AGS and HGC-27 cells (Fig. 3h, i). These results showed that CRABP2 can be independently combined with BAX and PARKIN. PARKIN is a protein that activates E3 ubiquitin-protein ligase activity [6]. To verify whether the combination of PARKIN and BAX promoted the degradation of BAX, we overexpressed CRABP2 in AGS cells and treated cells with cycloheximide (CHX) for 0, 2, 4, 6, 8, or 10 h. The results suggested that the half-life of the BAX protein decreased significantly (Fig. 3j). In AGS cells with CRABP2 knockdown, CHX showed the opposite effects (Fig. 3k). In summary, these results showed that CRABP2 promoted the degradation of BAX by expediting the binding of BAX and PARKIN in GC cells.

### CRABP2 facilitated the ubiquitination degradation of BAX and induced oxaliplatin resistance

**Fig. 4:**
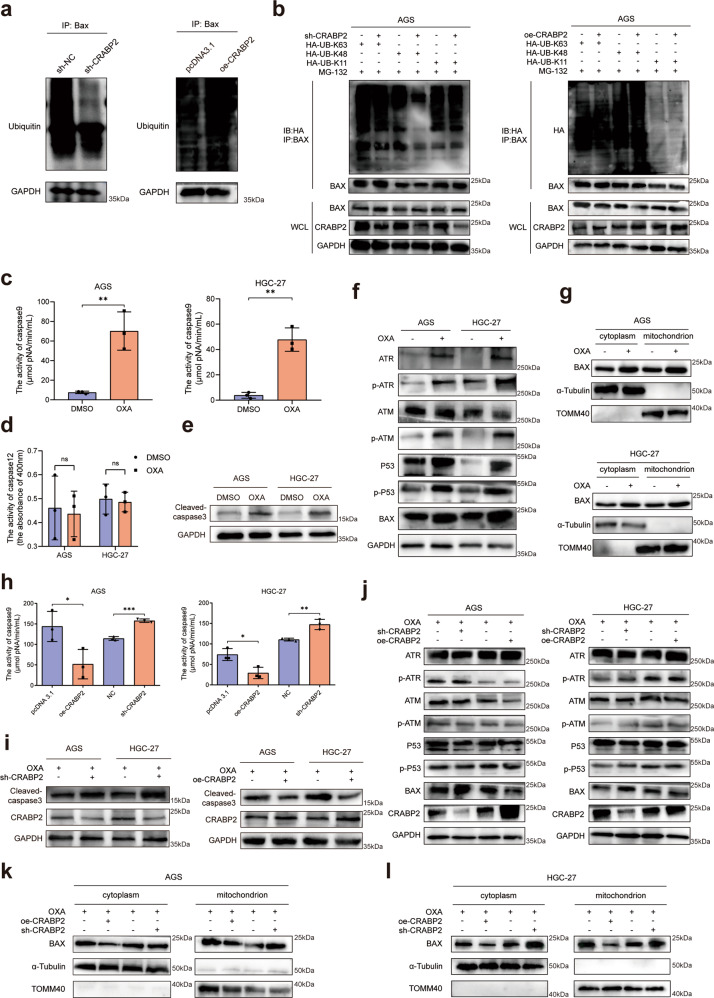
CRABP2 promoted chemoresistance by regulating ubiquitination degradation of Bax. **a** After adding MG-132 to AGS cells with knockdown or overexpression of CRABP2, Co-IP of Bax was performed, and ubiquitin was detected by western blotting. **b** GC cells with CRABP2 knockdown or overexpression were transfected with HA-labeled ubiquitinated plasmids of different sites, and Co-IP experiments of HA were performed. After adding OXA to AGS/HGC-27 cells, the activities of caspase 9 (**c**) and caspase 12 (**d**) were detected. **e** After adding OXA to AGS/HGC-27 cells, the expression of CC3 was detected. **f** After adding OXA to AGS/HGC-27 cells, the expression of DNA damage/repair-related proteins was examined by western blotting. **g** After AGS/HGC-27 cells were treated with OXA, the cytoplasm and mitochondria were separated, and the expression of BAX was examined. **h** The activities of caspase 9 were detected after CRABP2 knockdown/overexpression in AGS/HGC-27 cells. **i** The expression of CC3 was examined after CRABP2 knockdown/overexpression and OXA added to AGS/HGC-27 cells. **j** After adding OXA to AGS/HGC-27 cells and knocking down/overexpressing CRABP2, the expression of DNA damage/repair-related proteins was examined by western blotting. **k**, **l** After AGS/HGC-27 cells with CRABP2 knockdown or overexpression were treated with OXA, the cytoplasm and mitochondria were separated, and the expression of BAX was examined. (OXA oxaliplatin, CC3 cleaved caspase 3, TOMM40 translocase of outer mitochondrial membrane 40).

To further study the molecular mechanism of BAX degradation, we used MG-132 (Z-Leu-Leu-Leu-al, a proteasome inhibitor) to block the formation of the proteasome in AGS cells after knocking down or overexpressing CRABP2. The results of Co-IP and western blotting confirmed that CRABP2 could promote the ubiquitination degradation of BAX (Fig. 4a). To study the specific mechanism by which CRABP2 promotes BAX ubiquitination degradation, we interfered with/overexpressed the expression of CRABP2 and transfected HA-labeled ubiquitinated plasmids at different sites. Then, we carried out a Co-IP experiment of HA. The results confirmed that CRABP2 affects the ubiquitination of BAX at the 48th lysine residue (Lys48) (Fig. 4b). After adding OXA to AGS/HGC-27 cells, the activity of caspase 9 was significantly upregulated (Fig. 4c), but the activity of caspase 12 did not change significantly (Fig. 4d), which indicates that OXA mainly affected the mitochondrial apoptotic pathway. After adding OXA to AGS/HGC-27 cells, the expression of cleaved caspase 3 (CC3) was upregulated (Fig. 4e). Moreover, after adding OXA to AGS and HGC-27 cells, the expression of DNA damage/repair-related proteins increased (Fig. 4f), and OXA triggered apoptosis of the mitochondrial pathway caused by BAX (Fig. 4g). After CRABP2 knockdown/overexpression in AGS/HGC-27 cells, the activity of caspase 9 changed significantly (Fig. 4h). Moreover, after knocking down CRABP2, the expression levels of cleaved caspase 3 (CC3) were upregulated in both AGS and HGC-27 cells, while overexpressing CRABP2 led to the opposite results (Fig. 4i). To verify the effects of CRABP2 on OXA resistance, we added OXA to AGS and HGC-27 cells and knocked down/overexpressed CRABP2. The results suggested that CRABP2 can inhibit the activation of the P53-BAX apoptosis pathway induced by OXA (Fig. 4j). Moreover, after adding OXA to AGS/HGC-27 cells and overexpressing CRABP2, the apoptosis of the mitochondrial pathway triggered by OXA was inhibited, and vice versa (Fig. 4k, l). These results suggested that CRABP2 can inhibit cell apoptosis caused by the mitochondrial BAX-dependent pathway and induce oxaliplatin resistance in GC.

### CRABP2 promoted oxaliplatin resistance by inhibiting BAX-dependent cell apoptosis

**Fig. 5:**
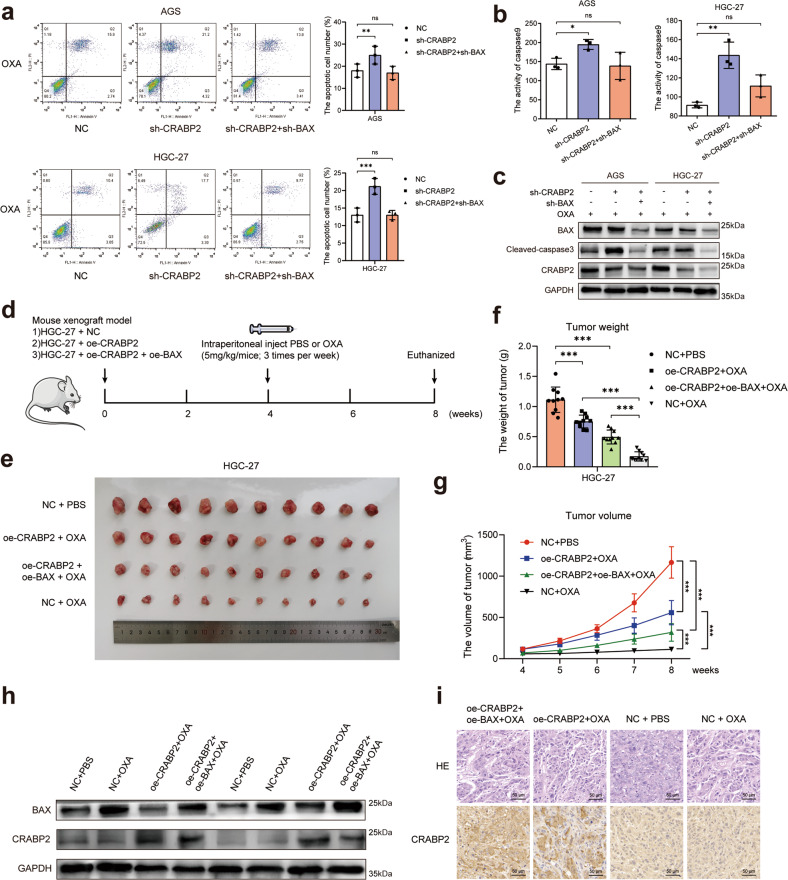
CRABP2 promoted oxaliplatin resistance through the BAX-dependent cell apoptosis pathway. **a** After knocking down CRABP2/ BAX and adding OXA (concentration: AGS 0.2 μM, HGC-27 0.4 μM), the percentage of apoptotic cells was examined by flow cytometry. **b** After knocking down CRABP2/ BAX and adding OXA (concentration: AGS 0.2 μM, HGC-27 0.4 μM), the activity of cleaved caspase 9 was examined. **c** After knocking down CRABP2/ BAX and adding OXA (concentration: AGS 0.2 μM, HGC-27 0.4 μM), the expression of cleaved caspase 3/BAX/CRABP2 was examined by western blotting. **d** Schematic diagram of the experimental procedures of the cell-derived xenograft model in nude mice. **e** Photo showing tumor formation in the four groups of nude mice. Tumor weight (**f**) and tumor volumes (**g**) of four groups of nude mice. **h** The expression of CRABP2 and BAX in tumors from four groups of nude mice by western blotting. **i** HE and CRABP2 IHC staining of tumors in four groups of nude mice. All experiments were performed in three replicates. *P < 0.05, **P < 0.01, ***P < 0.001.

Previous experiments suggested that CRABP2 inhibited BAX-dependent cell apoptosis caused by OXA. To verify the oxaliplatin resistance mechanisms of CRABP2, we performed experiments in vitro and in vivo. After knocking down CRABP2 and BAX simultaneously, the increased percentage of apoptotic cells caused by knocking down CRABP2 was restored (Fig. 5a), the activity of caspase 9 was restored (Fig. 5b), and the expression of cleaved caspase 3 was restored (Fig. 5c). These results suggest that the cell apoptosis inhibition of CRABP2 depends on BAX in vitro. To perform the in vivo experiments, we constructed a cell-derived xenograft (CDX) model. First, lentiviruses overexpressing CRABP2 or BAX (named oe-CRABP2 or oe-BAX, respectively) were used to infect HGC-27 cells. Then, forty nude mice were randomly divided into four groups (NC + PBS, oe-CRABP2 + OXA, oe-CRABP2 + oe-BAX + OXA, and NC + OXA) and were administered different treatments (see the Methods section and Fig. 5d). Finally, all mice were euthanized, and the tumors were removed and measured (Fig. 5e). The weight and volume of tumors from the four groups of CDX mice suggested that after overexpressing CRABP2 and BAX simultaneously, the increased chemoresistance ability of HGC-27 cells caused by overexpressing CRABP2 was reversed (Fig. 5f, g). Half of the tumors were used to examine the expression of CRABP2 and BAX in tumors from different groups by western blotting (Fig. 5h), and the other half of the tumors were examined with HE and IHC staining (Fig. 5i). The results showed that CRABP2 promoted oxaliplatin resistance through the BAX-dependent cell apoptosis pathway.

We further investigated the role of PARKIN in oxaliplatin resistance. After adding oxaliplatin, overexpressing CRABP2 and knocking down PARKIN simultaneously, the decreased level of cleaved caspase 3 (Supplementary Fig. 5a), the percentage of cell apoptosis (Supplementary Fig. 5b), and the activity of cleaved caspase 9 (Supplementary Fig. 5c) were restored. The in vivo experiments showed that knocking down PARKIN significantly reduced the tumor size (Supplementary Fig. 5f), volume (Supplementary Fig. 5g) and weight (Supplementary Fig. 5h). The expression of BAX/PARKIN was examined by western blotting (Supplementary Fig. 5i) and IHC (Supplementary Fig. 5j).

### The expression of CRABP2 was affected by TET1-mediated DNA hydroxymethylation

**Fig. 6:**
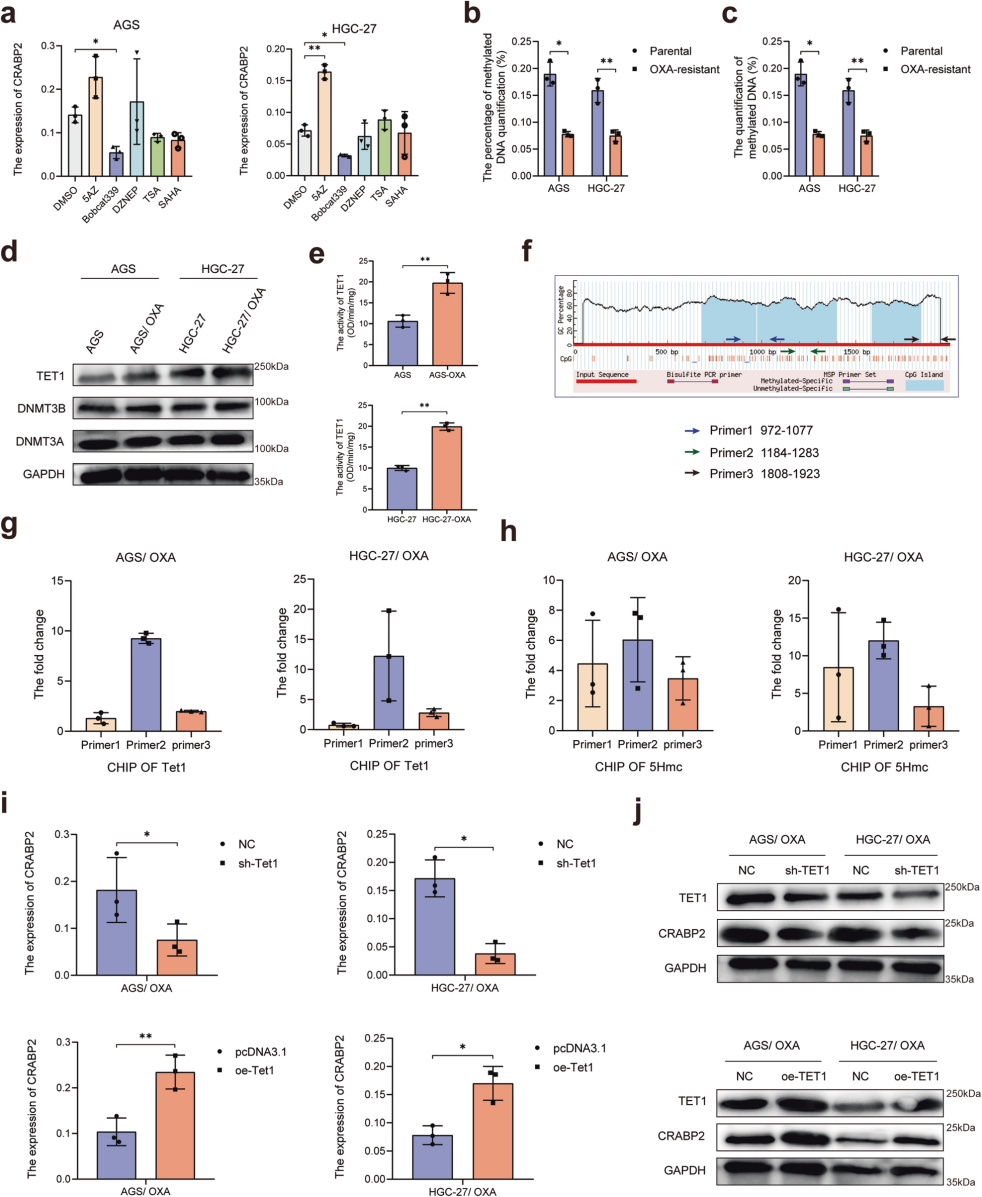
TET1-mediated DNA hydroxymethylation expedited the expression of CRABP2. **a** After adding 5AZ, Bobcat339, DZNEP, TSA, or SAHA to AGS and HGC-27 cells, the mRNA expression changes in CRABP2 were detected. The methylation (**b**) and hydroxymethylation (**c**) levels of OXA-resistant GC cells and parental GC cells were examined. **d** The expression and activity of DNA methylase, demethylase, and hydroxymethylase from OXA-resistant and parental GC cells were examined by western blotting. **e** The activity of hydroxymethylase TET1 was detected in OXA-resistant GC cells and parental GC cells. **f** Schematic diagram of the predicted and designed methylation sites in the promoter region of CRABP2. **g** ChIP experiments using a TET1 antibody were performed in OXA-resistant GC cells. **h** Hydroxymethylated ChIP assays were performed in OXA-resistant GC cells. After knocking down or overexpressing TET1 in OXA-resistant GC cells, the expression of CRABP2 was examined by qRT–PCR (**i**) and western blotting (**j**). *P < 0.05, **P < 0.01, ***P < 0.001, ns not significant.

Preliminary experiments showed that the expression of CRABP2 was significantly upregulated at the mRNA level in OXA-resistant GC cells compared with parental cells (Fig. 1h). Therefore, we verified the effects of epigenetic modification on the expression of CRABP2. After adding 5AZ (DNA methylase inhibitor, 5 mM), Bobcat339 (DNA hydroxymethylase inhibitor, 1 mM), DZNEP (histone methylase inhibitor, 5 mM), TSA (histone deacetylase inhibitor, 2 mM), SAHA (histone deacetylase inhibitor, 2 mM) to AGS and HGC-27 cells for 48 h, the mRNA expression changes in CRABP2 were detected. The results confirmed that CRABP2 was sensitive to methylated and hydroxymethylated modifications (Fig. 6a). We further examined the methylation/hydroxymethylation level of OXA-resistant GC cells and parental GC cells and found that the methylation level of OXA-resistant cells decreased (Fig. 6b), while the level of hydroxymethylation increased (Fig. 6c). After that, we detected the expression and activity of DNA methylase, demethylase, and hydroxymethylase from the OXA-resistant and parental GC cells and found that the expression levels of methylase and demethylase did not change significantly (Fig. 6d), while that of the hydroxymethylase TET1 was enhanced (Fig. 6d, e). Next, we used Methyl Primer Express v1.0 (https://products.appliedbiosystems.com) to predict and design the methylation sites in the promoter region of CRABP2 (Fig. 6f) and performed ChIP experiments using a TET1 antibody. The results confirmed that TET1 can be enriched in the promoter region of CRABP2 (Fig. 6g). The results of the hydroxymethylated ChIP assay also confirmed that there is a hydroxymethylated modification in the promoter region of CRABP2 (Fig. 6h). After knocking down or overexpressing TET1, the expression of CRABP2 was affected by TET1 in both OXA-resistant GC cells at both the mRNA and protein levels (Fig. 6i, j).

To confirm the important role of TET1 in oxaliplatin resistance, we performed in vitro and in vivo experiments. After adding oxaliplatin, overexpressing TET1 and knocking down CRABP2 simultaneously, the decreased level of cleaved caspase 3 (Supplementary Fig. 6a), the percentage of cell apoptosis (Supplementary Fig. 6b), and the activity of cleaved caspase 9 (Supplementary Fig. 6c) were restored, and vice versa. Furthermore, the expression of TET1 and CRABP2 in tumor tissues of GC patients was positively correlated, as shown by qRT-PCR (Supplementary Fig. 6d). The in vivo experiments showed that knocking down/overexpressing TET1 significantly affected the tumor size (Supplementary Fig. 6e), volume (Supplementary Fig. 6f) and weight (Supplementary Fig. 6g). The expression of TET1/BAX/CRABP2 was examined by western blotting (Supplementary Fig. 6h).

### Interference with CRABP2 reversed the oxaliplatin resistance of GC in vivo

**Fig. 7:**
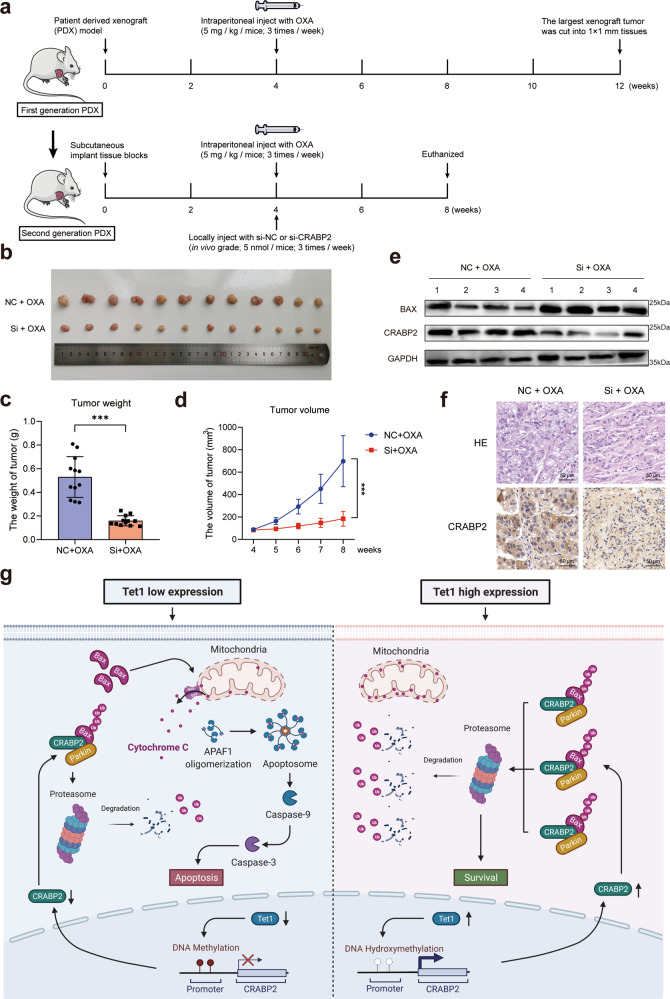
Interference with CRABP2 reversed oxaliplatin resistance in GC in vivo. **a** Schematic diagram of the patient-derived xenograft model of GC. **b** Photos of different tumor formations in the two groups of NOD-SCID mice. Tumor weight (**c**) and tumor volume (**d**) measurement in the two groups of mice. **e** Expression of CRABP2 and BAX in tumors from the two groups of mice, as determined by western blotting. **f** HE staining and CRABP2 staining of tumor slides from the two groups of mice. **g** Under normal physiological conditions, the synthesis and degradation of Bax in the cytoplasm are in equilibrium. When cells are treated with oxaliplatin, Bax integrates into the outer mitochondrial membrane, leading to the release of cytochrome C and cell apoptosis. In chemoresistant GC cells, in which the expression of CRABP2 in the cell is at a high level, CRABP2 can promote the ubiquitination degradation of Bax by combining Bax and Parkin, ultimately allowing the cells to survive. *P < 0.05, **P < 0.01, ***P < 0.001.

To further confirm the significance of CRABP2 in mediating oxaliplatin resistance in GC in vivo, we constructed a GC patient-derived xenograft (PDX) model (Fig. 7a). The details of the PDX model construction are shown in the Methods section. In brief, the tissues from tumors of GC patients were transplanted subcutaneously into the right axilla of mice and treated with OXA. The largest xenografts (indicating the most resistant to OXA) were isolated and transplanted subcutaneously into the right axilla of another mouse for the second-generation PDX model. Then, we constructed in vivo-grade siRNAs of CRABP2. After treatment with OXA and local injection of siRNAs, the mice were euthanized, and the tumors were removed and measured (Fig. 7b). We weighed the tumors and calculated the tumor volume of each group of mice (*P* < 0.001, Fig. 7c, d). The expression of CRABP2 and BAX was measured by western blotting (Fig. 7e). Tumor specimens were treated with formalin, embedded in paraffin and then examined via HE and IHC staining (Fig. 7f). The results suggested that the abnormally high expression of CRABP2 is affected by TET1-mediated DNA hydroxymethylation and promotes the ubiquitination of BAX by enhancing the binding of BAX and PARKIN, inhibiting cell apoptosis under oxaliplatin treatment (Fig. 7g).

## Discussion

Gastric cancer (GC) is one of the most common malignant tumors of the digestive system worldwide. Although surgery is the main treatment for GC, most patients still need chemotherapy to improve their prognosis [7]. Currently, chemotherapy is routinely used as an adjuvant treatment after surgery for GC patients [8]. In recent years, many scholars have recommended preoperative chemotherapy for advanced GC patients, which is called neoadjuvant chemotherapy (NAC) [9]. However, regardless of whether postoperative or preoperative chemotherapy is used, a considerable number of GC patients do not respond well, and the molecular mechanism of chemotherapy resistance is still unclear [10].

Among the various chemotherapy regimens for GC, oxaliplatin (OXA) is a basic drug for chemotherapy in GC patients [11]. Oxaliplatin belongs to the third generation of platinum antitumor drugs, which have no toxicity overlap with most chemotherapy drugs used and especially have a low incidence of blood and gastrointestinal toxicity [12]. The 2016 National Comprehensive Cancer Network (NCCN) guidelines established regimens containing oxaliplatin as the first-line chemotherapy regimen for the treatment of gastrointestinal malignant tumors [13]. At present, oxaliplatin is mainly used clinically to treat various malignant tumors of the digestive system, such as GC, liver cancer, and colorectal cancer [14, 15]. However, many patients with gastrointestinal tumors show resistance to oxaliplatin, leading to treatment failure and poor outcomes [16]. Some studies have explored the potential molecular mechanism of oxaliplatin resistance, including upregulation of ATP-binding cassette transporters (promoting efflux of chemotherapy drugs), oxaliplatin-induced DNA damage repair, enhanced resistance to apoptosis and autophagy, DNA methylation, and histone modification, but thus far, no effective molecule has been found to reverse the resistance to oxaliplatin [17].

The development of quantitative proteomics provides a possible method for us to discover key oxaliplatin-resistant molecules in GC patients [18]. In this study, we used this method and found that cellular retinoic acid binding protein 2 (CRABP2) was the most significantly upregulated protein in the tumor tissues of chemotherapy-resistant patients. CRABP2 belongs to the family of retinoic acid binding proteins and is the main regulator in the process of retinoic acid metabolism [19]. CRABP2 transports retinoic acid to the retinoic acid receptor in the nucleus and regulates cell proliferation, apoptosis, invasion, and migration [20]. It has been reported that the abnormal expression of CRABP2 is closely related to the development of neuroblastoma, Wilms tumor, head and neck squamous cell carcinoma, and non-small-cell lung cancer [21–23]. CRABP2 promotes the invasion and metastasis of pancreatic cancer cells, and inhibiting the expression of CRABP2 can inhibit the migration of cancer cells [24]. In esophageal squamous cell carcinoma, Koreeda et al. found that downregulating CRABP2 inhibits cell proliferation and induces cell apoptosis [25]. In addition, CRABP2 regulates the metastasis and invasion of breast cancer through the Hippo pathway [26].

In this study, we investigated the OXA resistance-related protein CRABP2, which was significantly upregulated in both chemoresistant GC tissues and cell lines. Moreover, the expression of CRABP2 was increased in the GC tumor tissues of our cohort and the TCGA database. We performed IHC staining of CRABP2 in 488 GC cases and confirmed that the expression of CRABP2 was significantly associated with the OS of GC patients. These results all implied that CRABP2 may play an important role in the chemoresistance of GC. To verify the molecular functions of CRABP2 in GC cells, we overexpressed CRABP2 in normal GC cells and knocked down CRABP2 in both OXA-resistant and normal GC cells. Then, we performed a series of cell functional experiments in the presence or absence of OXA in two GC cell lines. The results of these experiments all showed that CRABP2 promotes cell proliferation and inhibits cell apoptosis in GC cells, especially in the presence of OXA. Most chemotherapeutic drugs have anticancer effects by inhibiting cell proliferation and promoting cell apoptosis [27]. Thus, these results support the hypothesis that CRABP2 is an important molecule in the OXA resistance of GC.

The apoptotic cascade triggered by OXA is characterized by the translocation of BAX to mitochondria, the release of cytochrome c into the cytoplasm, and the activation of caspase 3 [28]. By the Co-IP assay, we identified the interacting proteins of CRABP2 in GC. The results of the GST pulldown assay verified that CRABP2 could directly interact with BAX and PARKIN in GC. The BAX protein belongs to the BCL-2 protein family, which is a proapoptotic effector [29]. It controls the mitochondrial apoptotic pathway, which is engaged by physiological stimuli or by cytotoxic drugs, including OXA. The interaction between BCL-2 pro- and antiapoptotic family members sets the apoptotic threshold that determines the life/death decision: once the balance is tipped toward the formation of BAX oligomers, the cell is committed to suicide [30]. Apoptosis signaling and caspase activation result in a conformational change in the normally monomeric BAX, which exposes the BH3 domain of BAX, leading to dimerization and resistance to ubiquitin degradation [31]. BAX then translocates into the mitochondria, resulting in the release of proapoptotic mitochondrial factors such as cytochrome c and second mitochondria-derived activator of caspase. This results in the activation of caspase-3 and formation of the apoptosome [32].

Ubiquitination is a common form of protein degradation, and the PARKIN protein is a highly effective product that activates E3 ubiquitin-protein ligase activity [33]. Our results suggested that CRABP2 binds to BAX and PARKIN. Thus, we assumed that CRABP2 can promote the degradation of BAX. Our study proved that CRABP2 promotes the binding of BAX and PARKIN, leading to the ubiquitination and degradation of BAX. This effect finally inhibited cell apoptosis and achieved OXA resistance in GC cells. Apoptosis induces a conformational change in BAX that inhibits proteasome degradation [34]. In nonapoptotic cells, BAX dimerization is inhibited because the hydrophobic C-terminus of BAX masks the BH3 domain [35]. This configuration is also more susceptible to proteasome degradation. After apoptosis signaling, a caspase-dependent conformational change results in resistance to proteasome degradation and translocation of BAX to the mitochondria, which correlates to the release of cytochrome c that amplifies apoptosis signaling events [36]. BAX integrates into the outer mitochondrial membrane, leading to the release of cytochrome C and cell apoptosis [37]. Furthermore, to verify the OXA resistance mechanisms of CRABP2, we performed rescue experiments in vitro and in vivo. The results showed that CRABP2 promotes OXA resistance through the BAX-dependent cell apoptosis pathway. Then we performed in vitro and in vivo experiments to prove the role of PARKIN in oxaliplatin resistance (Supplementary Fig. 5). Finally, through the above experiments, we confirmed that the mechanism of CRABP2 involved in oxaliplatin resistance is to inhibit cell apoptosis through the degradation of BAX by PARKIN.

In this study, we also investigated the molecular mechanisms of abnormally high expression of CRABP2 in OXA-resistant GC. The results confirmed that CRABP2 was sensitive to methylated and hydroxymethylated modifications. The methylation level of OXA-resistant cells decreased, while the level of hydroxymethylation increased. We further confirmed that the activity of the hydroxymethylase TET1 was enhanced and that TET1 could be enriched in the promoter region of CRABP2. TET1 belongs to the ten eleven translocation (TET) family of proteins, which are important regulators of DNA methylation. TET proteins can oxidize 5-methylcytosine (5mC) to 5-hydroxymethylcytosine (5hmC), which is the basis for the demethylation of TET proteins [38]. In our study, the hydroxymethylated ChIP assay confirmed that there is a hydroxymethylated modification in the promoter region of CRABP2. TET1 plays important roles in embryonic development, germ cell formation and bone marrow hematopoiesis [39]. In hepatocellular carcinoma, the upregulation of TET1 could drive cell growth through aberrant enhancer hydroxymethylation of HMGA2 [40]. TET1 has been reported to inhibit the progression of nonalcoholic fatty liver disease by hydroxymethylation of the PPARα promoter [41]. In this study, we proved the role of TET1 in oxaliplatin resistance through in vitro and in vivo experiments (Supplementary Fig. 6). The results of our study verified that the expression of CRABP2 is affected by TET1-mediated DNA hydroxymethylation in OXA-resistant GC.

To explore the value of CRABP2 as a potential therapeutic target, we established a patient-derived xenograft (PDX) model. We further constructed a small interfering RNA (siRNA) targeting CRABP2 and verified the interference efficiency. siRNA is a short fragment of 21–23 bp double-stranded RNA that can induce RNA interference [42]. Since siRNA drugs for cancer treatment entered clinical trials for the first time in 2008, siRNA is expected to become an effective treatment method for tumor treatment due to its specificity and high efficiency [43]. In this study, we constructed in vivo-grade siRNA injections using LIPID In Vivo Transfection Reagent (Altogen Biosystems) to ensure the interference efficiency of CRABP2 in vivo [44]. The results of the PDX model suggested that interference with CRABP2 reversed OXA resistance in vivo. These results reveal the potential of CRABP2 as a molecular therapeutic target in chemoresistant GC patients.

In conclusion, the results of our study show that increased expression of CRABP2 by TET1-mediated DNA hydroxymethylation in GC is related to OXA resistance and could be a poor prognostic factor in GC patients. In OXA-resistant GC cells, CRABP2 was found to promote the binding of BAX and PARKIN, leading to the ubiquitination and degradation of BAX and ultimately preventing the apoptosis of cells under OXA treatment. This article revealed the key role of CRABP2 in regulating OXA resistance, which could be a potential new target for reversing the chemoresistance of GC.

## Materials and methods

### Patient samples and clinical data collection

From 2017 to 2019, patients diagnosed with advanced GC who received neoadjuvant chemotherapy (NAC) at Qilu Hospital of Shandong University were enrolled in this study. All patients received the SOX regimen [oxaliplatin+S-1(tegafur gimeracil oteracil potassium capsule)] (oxaliplatin 130 mg/m^2^, intravenously, day 1; and S-1, 40–60 mg, twice a day, orally, days 1 to 14) for 2 to 4 cycles and were evaluated for response to treatment before surgery [45]. All patients received enhanced computed tomography (CT) before the first and after the last NAC. The chemotherapy response was evaluated following the Response Evaluation Criteria in Solid Tumors (RECIST) 1.1 criteria [46]. Partial response (PR) was defined as at least a 30% decrease in the sum of the diameters of target lesions, taking as reference the baseline sum diameters. Progressive disease (PD) was defined as at least a 20% increase in the sum of the diameters of target lesions, taking as reference the smallest sum on the study. Stable disease (SD) was defined as neither sufficient shrinkage to qualify for PR nor an increase to qualify for PD [47]. The pathological features were evaluated according to the National Comprehensive Cancer Network (NCCN) tumor regression grading (TRG) score [48]. A response was defined as no evidence that the tumor had upstaged and a TRG score of 0 to 3. We collected tumors and matched adjacent nontumor (nearby tissues that were greater than 5 cm away) tissues within 30 min after resection of the specimen. Each specimen was rinsed with sterile saline and cut into two pieces. One piece was formalin-fixed and paraffin-embedded for pathology examination. Another piece was restored in liquid nitrogen and then transferred to a −80 °C refrigerator and used for quantitative proteomics and western blotting. Then, based on a rigorous evaluation of radiological and pathological evidence, the cases were classified into two groups: chemotherapy sensitive (group PR, *n* = 5) and chemotherapy resistant (group SD, *n* = 5) (Supplementary Table 1) [49]. All patients received follow-up every 6 months from the date of surgery. All patients provided written informed consent before treatment and sample collection. This study was approved by the Ethics Committee of Qilu Hospital of Shandong University.

### Tandem mass tags (TMT) based quantitative proteomics

Total protein extraction was carried out according to the flow of the protein extraction kit (Thermo Fisher, MA, USA). Total protein extraction was performed following the manufacturer’s protocols [50]. BSA standard protein solution was prepared according to the instructions of the Bradford protein quantitative kit, with gradient concentrations ranging from 0 to 0.5 g/L. Proteins were extracted, digested with trypsin, and labeled with TMT reagents. The pooled peptides were separated into 15 fractions using a C18 column (Waters BEH C18 4.6 × 250 mm, 5 μm) on a Rigol L3000 HPLC. A total of 120 μg of each protein sample was used. Then, 100 μL of 0.1 M TEAB buffer was added for reconstitution, 41 μL of acetonitrile-dissolved TMT labeling reagent was added, and the sample was mixed with shaking for 2 h at room temperature. Then, the reaction was stopped by adding 8% ammonia. All labeled samples were mixed with equal volumes, desalted, and lyophilized. For transition library construction, shotgun proteomics analyses were performed using an EASY-nLCTM 1200 UHPLC system (Thermo Fisher, MA, USA) coupled with a Q Exactive HF-X mass spectrometer (Thermo Fisher, MA, USA) operating in the data-dependent acquisition (DDA) mode. The resulting spectra from each run were searched separately against the database by the search engine Proteome Discoverer 2.2 (Thermo Fisher, MA, USA). The protein quantitation results were statistically analyzed by *t test*. The proteins whose quantitation was significantly different between the experimental and control groups (*P* < 0.05 and |log2FC | >1 (ratio >1 or ratio <1 [fold change, FC])) were defined as differentially expressed proteins (DEPs).

### RNA extraction and quantitative real-time PCR (qRT–PCR)

RNA was extracted from cultured cells and tumor tissues using the RNeasy Micro Kit (Qiagen, Hilden, Germany). Total RNA was reverse transcribed into cDNA with PrimeScript RT Master Mix (TaKaRa, otsu, Japan). SYBR green qRT–PCR was performed using PCR Master Mix (Life Technology, NY, USA). The expression of the target gene was determined relative to β-actin, and relative expression was calculated by the ΔΔCt method. The primer sequences for qRT–PCR used in this study are shown in Supplementary Table 4.

### Cell culture and chemoresistant cell line induction

The human gastric cell lines AGS and HGC-27 were purchased from the China Center for Type Culture Collection (Shanghai, China). The cells were cultured in RPMI 1640 (Life Technology, NY, USA) supplemented with 10% fetal bovine serum (HyClone, UT, USA), 100 units/mL penicillin, and 100 mg/mL streptomycin (HyClone, UT, USA) in humidified 5% CO2 at 37 °C. Oxaliplatin of 99.9% purity was purchased from MCE (Monmouth Junction, NJ, USA). The drug-resistant cancer cell lines AGS-OXA and HGC-27-OXA were obtained from the AGS and HGC-27 cell lines by selection with 0.1 μM OXA for 6 months (culture medium was changed with fresh medium containing OXA every 3 days). All human cell lines were authenticated using STR profiling within the last 3 years, and all experiments were performed with mycoplasma-free cells. Drug resistance was confirmed using a cell viability assay after treatment with increasing doses of OXA. AGS-OXA and HGC-27-OXA cells were cultured in the same medium containing 5 μM OXA [51].

### Lentiviral vector construction and cell infection

The coding sequences of CRABP2/BAX/TET1 were inserted into a lentiviral vector (pUbi-MCS-3FLAG-SV40-PURO, GV611) to stably overexpress CRABP2/BAX/TET1 (named oe-CRABP2/oe-BAX/oe-TET1). Moreover, the sh-RNA sequences of CRABP2/PARKIN/TET1 were inserted into lentiviral vectors (phU6-MCS-pCMV-PURO, GV112) to stably knock down CRABP2/PARKIN/TET1 (named sh-CRABP2/sh-PRKN/sh-TET1). The overexpression and knockdown lentiviral vector and the empty lentiviral vector (NC) were constructed by GeneChem Co., Ltd. (Shanghai, China). Then, the cells were infected with lentiviruses and selected with puromycin (1.2 μg/ml). After infection, the expressions were examined by western blotting (Supplementary Fig. 7).

### Cell transfection

The sh-RNAs used in this study were designed and synthesized by Hanbio Life Science Co., Ltd. (Shanghai, China). The sh-RNA sequences used in the study are shown in Supplementary Table 4. The cells were transfected using Lipofectamine RNAiMAX Transfection Reagent (Invitrogen, MA, USA). The cells were harvested after 48 h, then examined by western blotting (Supplementary Fig. 7).

### Cell Counting Kit (CCK)-8 assay

After 48 h of cell transfection, the cells were seeded in 96-well plates (2–3 × 10^3^ cells/well), and the plates were cultured in an incubator. After cell attachment, 10 μL of CCK-8 solution (Apexbio, TX, USA) was added to each well on days 1–5. After incubation for 2 h, the absorbance was measured at 450 nm using an enzyme-linked immunosorbent assay. All results are expressed as the mean ± SD of three independent experiments.

### Colony formation assay

For the colony formation assays, the cells were counted after trypsinization following transfection for 48 h, and 500 cells were added to each well of a 6-well plate. After being cultured for 8–14 days, the cells were counted using 0.05% crystal violet staining. Then, the cell colonies were counted and analyzed.

### Cell viability assay

The cells were seeded in a 96-well plate and cultured for 24 h, and then drugs were added after the cells adhered. Different concentrations of OXA were added to the wells of plates according to the experimental requirements. Each concentration was assessed in 3 experimental replicates. An adjustment well without cells and a control well without drugs were used as a control. After being cultured for 48 h, the culture medium was discarded, 100 μl of fresh complete medium was added to each well, and 10 μl of CCK-8 (Dojindo, Tokyo, Japan) solution was added to each well. After being cultured for 1–4 h, cell viability was measured at OD450 nm with Thermo Scientific Multiskan FC (Thermo Fisher, MA, USA). According to the cell survival rate for different drug concentrations, GraphPad Prism 8 software (USA) was used to draw the curves and calculate the 50% inhibitory concentration (IC50) value.

### Flow cytometry assay

For the apoptosis assay, cells cultured in 96-well plates were collected, and then 5 μl of Annexin V-FITC (Sigma–Aldrich, MO, USA) and 5 μl of propidium iodide (PI) staining (50 μg/ml, Sigma–Aldrich, MO, USA) were added. Cell apoptosis was analyzed on a flow cytometer (Thermo Fisher, MA, USA). FlowJo 7.6.2 software (BD Pharmingen, NJ, USA) was used for analysis.

### Activity detection of caspase 9 and caspase 12

The activities of caspase 9/12 were detected following the instructions of the caspase 9 and caspase 12 activity detection kits (BioVision, CA, USA). After knockdown or overexpression of CRABP2 in cells and treatment with OXA, the activity changes in caspase 9 and caspase 12 were detected.

### Co-immunoprecipitation (Co-IP) assay

For the endogenous CRABP2 binding assay, 1 × 10^7^ GC cells were collected and washed twice with PBS. For the validation of endogenous CRABP2, BAX, PARKIN binding, sh-NC/sh-CRABP2/sh-BAX/sh-PRKN were transfected into GC cells. The supernatant fluid was collected by ice lysis with 1 ml of IP lysate for 20 min and centrifuged at 12,000 × *g* for 15 min at 4 °C. The supernatant fluid was divided into three groups: IgG (450 µl), IP (450 µl), and input (100 µl). Co-IP was carried out following the instructions of Protein A + G Agarose (Thermo Fisher, MA, USA). Co-IP samples were subjected to SDS–PAGE electrophoresis [52]. Further analysis was performed by mass spectrometry and verified by western blotting. For ubiquitination type detection, sh-NC or sh-CRABP2 with HA-UB-K63/HA-UB-K48/HA-UB-K11 plasmids were co-transfected into GC cells. The samples were treated with Co-IP after 48 h, BAX antibody was used for pull-down, and the antibody was used to detect ubiquitination levels. At the same time, the interference efficiency of CRABP2 in the lysate was detected.

### Immunohistochemistry (IHC) and scoring

Paraffin sections of GC patient tissues and xenografts were prepared for IHC following standard protocols. To eliminate interference factors, patients with distant metastasis (M1 stage) were excluded. The slices were observed by IHC Imager (Leica, Germany), and the expression level of the target protein was evaluated by the ratio and intensity of the positive cells detected in 5 fields of view on each slide for scoring. The IHC intensity was scored as follows [53]: 0 (negative), 1 (weak), 2 (medium) or 3 (strong). The percentage of positive cells was scored as 0 (negative), 1 (1–25%), 2 (26–50%), 3 (51–75%), or 4 (76–100%). IHC score = percentage of positive cells score × IHC intensity score. A final score ranging from 0–3 was considered “negative”, and 4–12 was considered “positive”.

### Immunofluorescence

Immunostaining was performed as standard protocol. Briefly, AGS cells were seeded in 24-well slides. Then, the cells were fixed with 4% paraformaldehyde for 15 min and rinsed three times with PBS. The cells were neutralized with 5% glycine for 5 min and penetrated with 5% Triton X-100 for 15 min. The slides were incubated with anti-goat serum for 1 h and then washed three times with PBS. Next, the slides were incubated with a mouse-derived CRABP2 (ProteinTech, Cat. #66468-1-Ig) primary antibody/ rabbit-derived BAX (ProteinTech, Cat. #50599-2-lg) primary antibody/ mouse-derived BAX (ProteinTech, Cat. #60267-1-Ig) primary antibody/rabbit-derived PARKIN (ProteinTech, Cat. #14060-1-AP) primary antibody overnight. The rabbit-derived fluorescent secondary antibody with red fluorescence and the mouse-derived fluorescent secondary antibody with green fluorescence were incubated at room temperature for 1 h and washed three times with PBS. The slides were stained with DAPI (Thermo Fisher, MA, USA) for 5 min and washed three times with PBS. Then, the slides were transferred to carrier glass, and the slides were photographed by confocal microscopy LSM980 (Carl Zeiss AG, Oberkochen, Germany).

### Glutathione S transferase (GST) pull-down assay

To study the interaction between CRABP2 and BAX/PARKIN, we cultured *Escherichia coli (E. coli)* B21 transformed with plasmid encoding GST or GST-CRABP2 fusion protein overnight at 37 °C to purify GST or GST-CRABP2 fusion protein. The plasmids used in this experiment were constructed by the Biosune Biotechnology Co., Ltd. (Shanghai, China). Then, isopropyl-β-D-thiogalactoside was added and incubated for 4 h. Lyse the bacteria with PBST buffer (10 mM sodium phosphate, 2 mM potassium phosphate, 140 mM NaCl, 3 mM KCl, 0.1% Tween 20). Ultrasound was then used to destroy the cell wall. Then, 100 μl of 20% Triton X-100 was added and placed on ice for 30 min. The bacterial lysate supernatant was incubated with glutathione agarose (Thermo Fisher, USA) for 30 min at 4 °C to isolate the GST or GST-CRABP2 fusion protein. Resuspend the magnetic beads bound to the purified GST or GST-CRABP2 fusion protein. At the same time, *E. coli* B21 transformed with GST-BAX/PARKIN fusion protein guided by 6×His was cultured overnight at 37 °C. The subsequent steps were the same as those for studying the GST or GST-CRABP2 fusion protein. For GST pulldown, the empty carrier protein, GST-BAX/PARKIN fusion protein, and the supernatant of the cell lysate were incubated with the purified GST-CRABP2 fusion protein (50 μg/ml) at 4 °C for 6 h. The incubation was terminated by adding agarose beads and washing 3 times with PBST buffer. The BAX/PARKIN protein bound to the CRABP2-GST fusion protein was analyzed by western blotting.

### Quantitative analysis of genomic methylation and hydroxymethylation

The genomic DNA of the AGS and HGC-27 parental and OXA-resistant cells was extracted by the TIANamp Genomic DNA Kit (TIANGEN Biotech, Beijing, China). The absorbance value of 100 ng genomic DNA at 450 nm of each sample was detected by the Global DNA Methylation Assay Kit (Abcam, MA, USA; ab233486) and the Global DNA Hydroxymethylation Assay Kit (Abcam, MA, USA; ab233487). The percentage of methylated/hydroxymethylated modifications was calculated according to the following formula: 5-mC% = (Sample OD − Negative Control OD)/Slope x S * 100%; 5-hmC% = (Sample OD − Negative Control OD)/Slope x S * 100%.

### Quantitative detection of methylation modification of the CRABP2 promoter

Methyl Primer Express^TM^ v1.0 (Applied Biosystems, MA, USA) software was used to predict the methylation site of the CRABP2 promoter region and design relevant primers. Then, genomic DNA was extracted from the parental and OXA-resistant GC cells. Ultrasonication was performed for 10 sec, followed by a 30-s pause, which was repeated 4 times with 48 W of power. Genomic DNA was sheared into 200- to 1000-bp fragments under ultrasonic conditions. Then, according to the instructions of the Hydroxymethylation Quantitative Detection Kit (Abcam, Cambridge, UK), methylation-modified DNA fragments were obtained, and the difference in methylation modification between the two cell lines was detected by qRT–PCR. The parental and OXA-resistant GC cells were crosslinked with 1% formaldehyde, and the genomic DNA was sheared into 200- to 1000-bp fragments under the same ultrasonic conditions. Then, the CRABP2 promoter region was enriched using an anti-TET1 antibody (Abcam, MA, USA; ab272900) and was detected by the Chromatin Immunoprecipitation Kit (Abcam, MA, USA; ab500).

### Western blotting

Cells were first lysed (Solarbio, Beijing, China) and then denatured in SDS buffer to obtain total protein. The protein was separated on a 10% SDS-polyacrylamide gel (30 mg per lane) and transferred to a PVDF membrane (Merck Millipore, MA, USA). After blocking in 5% skim milk for 1 h, the membrane was incubated with primary antibody overnight at 4 °C and then incubated with the secondary antibody (1:15,000, GE Healthcare, Cambridge, UK) for 1 h at room temperature. Then, visualization was performed with ECL chemiluminescence reagent (Merck Millipore, MA, USA).

The antibodies used in this study were as follows: CRABP2 (1:1,000, ProteinTech, Cat. # 10225-1-AP), BAX (1:1,000, Abcam, ab32503), BCL-2 (1:1,000, Abcam, ab32124), cleaved caspase-3 (1:1,000, Abcam, ab32042), PARKIN (1:1,000, ProteinTech, Cat. # 14060-1-AP), ATR (1:1,000, Abcam, ab2905), p-ATR (1:1,000, Abcam, ab178407), ATM (1:1,000, Abcam, ab32420), p-ATM (1:1,000, Abcam, ab81292), P53 (1:1,000, Abcam, ab32389), p-P53 (1:1,000, Abcam, ab33889), TET1 (1:1,000, Abcam, ab272900), DNMT3A (1:1,000, Abcam, ab188470), DNMT3B (1:1,000, Abcam, ab2851), P62 (1:1,000, ProteinTech, Cat. # 18420-1-AP), TOMM40 (1:1,000, Abcam, ab185543), α-tubulin (1:5,000, Abcam, ab7291), β-actin (1:5,000, ProteinTech, Cat. # 66009-1-Ig), GAPDH (1:20,000, ProteinTech, Cat. # 60004-1-Ig).

### Nude mice xenograft assays

All animal experiments were performed humanely and in compliance with the protocol reviewed by the Ethics Committee of Qilu Hospital of Shandong University. Four-week-old male BALB/c nude mice were purchased from Vital River Laboratory Animal Technology Co., Ltd. (Beijing, China). For the cell-derived xenograft (CDX) model, the mice were randomly divided into three groups: 1 × 10^6^ HGC-27 cells infected with negative control lentivirus (group NC, 20 mice), CRABP2 overexpression lentivirus (group oe-CRABP2, 10 mice) or CRABP2 plus BAX overexpression lentivirus (group oe-CRABP2 + oe-BAX, 10 mice) were injected into the right axilla of nude mice. Four weeks after the injection, the growth of tumors was observed. Then, the mice in the NC group were randomly divided into two groups (10 mice in each group) and intraperitoneally injected with the same amount (5 mg/kg) of PBS or PBS-dissolved oxaliplatin into three times per week. The mice in the other two groups were given the same amount of PBS-dissolved oxaliplatin three times per week.

For the PARKIN and TET1 in vivo assay, the same amounts of HGC-27 cells infected with the negative control lentivirus (group NC), PARKIN knocking down lentivirus (group sh-PRKN), TET1 knocking down lentivirus (group sh-TET1) or TET1 overexpression lentivirus (group oe-TET1) were injected into the right axilla of nude mice. After four weeks, each nude mouse was intraperitoneally injected with the same amount (5 mg/kg) of PBS-dissolved oxaliplatin three times per week.

For the patient-derived xenograft (PDX) model, we selected a GC patient who did not receive adjuvant chemotherapy before surgery. GC tissues were collected from this patient within 30 min after surgery. Then, the tissues were cleaned and cut into 1 mm^3^ tissue blocks and transplanted subcutaneously into the right axilla of twenty NOD-SCID (Non-Obese Diabetes- Server Combined Immune Deficiency) mice. After four weeks, when the xenografts reached approximately 100 mm^3^, all the mice were intraperitoneally injected with saline-dissolved oxaliplatin (5 mg/kg/mouse; 3 times/week). Eight weeks later, the largest xenografts (indicating the most resistant to OXA) were isolated and cut into 1 mm^3^ tissue blocks. Then, the tissue blocks were transplanted subcutaneously into the right axilla of another twenty NOD-SCID mice for the second-generation PDX model [54]. After four weeks, when the xenografts reached nearly 100 mm^3^, the mice were randomly divided into two groups. Then, we constructed in vivo-grade siRNA injections using LIPID In Vivo Transfection Reagent (Altogen Biosystems, NV, USA). All mice were treated with oxaliplatin using the above scheme, and each xenograft was intraperitoneally injected with normal saline-dissolved oxaliplatin (5 mg/kg/mouse; 3 times/week) and locally injected with 5 nmol siRNA (si-CRABP2, Group 1) or its negative control (si-NC, Group 2) three times a week for four weeks. The sequences of siRNA oligonucleotides used were as follows: si-CRABP2, 5ʹ-GCCAGCAGTGGAGATCAAATT-3ʹ; si-NC, 5ʹ-TTCTCCGAACGTGTCACGT-3ʹ.

In these nude mice xenograft models, the longest and shortest tumor diameters were measured every three days and recorded as L and W, respectively. The tumor volume was calculated as follows: V (mm^3^) = L×W^2^×0.5. After all treatments were administered, the mice were euthanized, and the xenografts were removed. Half of the tumors were frozen in liquid nitrogen, and total proteins were extracted following the abovementioned method. The expression of CRABP2 and BAX in tumors from different groups was examined by western blotting. The other half of the tumors were immersed in formalin and embedded in paraffin, followed by hematoxylin-eosin (HE) staining to confirm the pathological diagnosis, and the expression of CRABP2 was detected by IHC.

### Statistical analysis

The Cancer Genome Atlas (TCGA) data were downloaded from The Cancer Genome Atlas-Stomach Adenocarcinoma (TCGA-STAD) server through client software, and then the data were extracted and analyzed. We downloaded the RNAseq data of gastric cancer samples from TCGA, converted the data into TPM (transcripts per million reads) and performed log2 transformation. Because the sample distribution did not conform to the normal distribution, we used the Mann–Whitney U test (Wilcoxon rank sum test). Outlier values [median±2 interquartile range (IQR)] and extreme values (median ± 3.5 IQR) were excluded. All enrolled cases were staged according to the 7th edition American Joint Committee on Cancer (AJCC) staging system. Patient demographic information, histopathological information, pathological tumor stage (pT stage), pathological lymph node stage (pN stage), pathological tumor-lymph node-metastasis stage (pTNM stage), treatment details (including chemotherapy details, surgical types), and survival data were collected. Overall survival (OS) was defined as the time interval from the date of initial surgical resection to the date of the last known contact or death. All values are expressed as the mean ± standard deviation (SD). SPSS 20.0 (IBM, USA) and GraphPad Prism 8.0 (GraphPad Software, USA) software were used to analyze the data and make graphs. The correlation between CRABP2 and clinicopathological characteristics was assessed by *χ*^2^ or Fisher’s test. The Kaplan–Meier method was used to analyze the survival curves, and the log-rank test was used to compare the statistical significance. Important variables in univariate survival analysis were included in the Cox proportional hazards regression model. The *t test* was used to compare two independent groups, and one-way analysis of variance was used to compare multiple groups. *P* < 0.05 was considered statistically significant.

## Supplementary information

Supplementary legends

Supplementary Figure 1

Supplementary Figure 2

Supplementary Figure 3

Supplementary Figure 4

Supplementary Figure 5

Supplementary Figure 6

Supplementary Figure 7

Supplementary Table 1

Supplementary Table 2

Supplementary Table 3

Supplementary Table 4

The original gels of western blot

Reproducibility Checklist

Language Editing Certificate

## Data Availability

The datasets used and/or analyzed during the current study are available from the corresponding author upon reasonable request.
