# Supplementary material for: Upregulation of CRABP2 by TET1-mediated DNA hydroxymethylation attenuates mitochondrial apoptosis and promotes oxaliplatin resistance in gastric cancer
Source: Cell Death Dis. 2022 Oct 4;13(10):848. doi: 10.1038/s41419-022-05299-2 (PMC9532395; doi:10.1038/s41419-022-05299-2)
Supplement: Supplementary file 1 — Supplementary legends [file 41419_2022_5299_MOESM1_ESM.docx]

**SUPPLEMENTARY LEGENDS**

**Supplementary Figure 1. The cell viability and IC50 values of parental and oxaliplatin-resistant GC cell lines.** a-b. Cell viability curves and IC50 values of AGS (a) and AGS-OXA (b) cells. c-d. The cell viability curves and IC50 values of HGC-27 (c) and HGC-27-OXA (d) cells.

**Supplementary Figure 2.** **Correlation curves between the tumor diameter (a), surgical type (b), tumor differentiation (c), pT stage (d), pN stage (e), pTNM stage (f), lymphovascular invasion (g), and overall survival in GC patients (n=488).**

**Supplementary Figure 3.** **CRABP2 showed no significant effects on the invasion and migration ability of GC cells.** a. The results of the Transwell migration assay after interference of CRABP2 in AGS and HGC-27 cells. b. The results of the Transwell invasion assay after interference with CRABP2 in AGS and HGC-27 cells. c-d. The results of the wound healing assay after interference with CRABP2 in AGS (c) and HGC-27 (d) cells. All experiments were performed in three replicates.

**Supplementary Figure 4.** **Knocking down CRABP2 inhibits oxaliplatin resistance of AGS and HGC-27 cells.**

a. After knocking down CRABP2, the viability of AGS and HGC-27 cells was determined in the presence of different concentrations of OXA.

b. After knocking down CRABP2, the colony forming abilities of AGS and HGC-27 cells were determined.

c. Results of AGS and HGC-27 cell apoptosis after knocking down CRABP2, as determined by flow cytometry.

All experiments were performed in triplicates. The data are presented as the mean ± SD. **P* <0.05, ***P* <0.01, ****P* <0.001.

**Supplementary Figure 5. Interference with PARKIN expression inhibits oxaliplatin resistance *in vitro* and *in vivo*.**

a-c. After adding OXA to cells as indicated, the expression of the BAX/PARKIN/CC3/CRABP2 proteins (a), percentage of apoptotic cells (b), and activity of caspase 9 (c) were examined.

d. Photograph showing tumor formation in different groups of nude mice.

e. Tumor volumes in different groups of nude mice.

f. Tumor weights in different groups of nude mice.

g. Expression of PARKIN and BAX in tumors from different groups of nude mice, as determined by western blotting.

**Supplementary Figure 6. TET1 contributed to oxaliplatin resistance *in vitro* and *in vivo*.**

a-c. After adding OXA to cells as indicated, the expression of the BAX/TET1/CC3/CRABP2 proteins (a), the percentage of apoptotic cells (b), and the activity of caspase 9 (c) were examined.

d. Expression of TET1 and CRABP2 in GC tumor tissues was positively correlated, as detected by qRT-PCR.

e. Photograph showing tumor formation in different groups of nude mice.

f. Tumor volumes in different groups of nude mice.

g. Tumor weights in different groups of nude mice.

h. Expression of TET1/BAX/CRABP2 in tumors from different groups of nude mice, as determined by western blotting.

**Supplementary Figure 7. The expression of proteins was examined by western blotting after transfection with sh-RNAs.** a-d. After knocking down the CRABP2 (a)/BAX (b)/PARKIN (c)/TET1 (d) in AGS and HGC-27 cells, western blotting was used to detect protein expression. e-f. After overexpressing CRABP2 (e)/TET1 (f) in AGS and HGC-27 cells, western blotting was used to detect protein expression.

**Supplementary Table 1. Clinical characteristics of the GC patients enrolled for quantitative proteomics in this study.**

**Supplementary Table 2. Differentially expressed proteins identified by tandem mass tag (TMT)-based quantitative proteomics.**

**Supplementary Table 3. Differentially expressed proteins after coimmunoprecipitation and mass spectrometry.**

**Supplementary Table 4. Primer sequences used in this study.**
