# Supplementary figures and images for: Upregulation of CRABP2 by TET1-mediated DNA hydroxymethylation attenuates mitochondrial apoptosis and promotes oxaliplatin resistance in gastric cancer
Source: Cell Death Dis. 2022 Oct 4;13(10):848. doi: 10.1038/s41419-022-05299-2 (PMC9532395; doi:10.1038/s41419-022-05299-2)

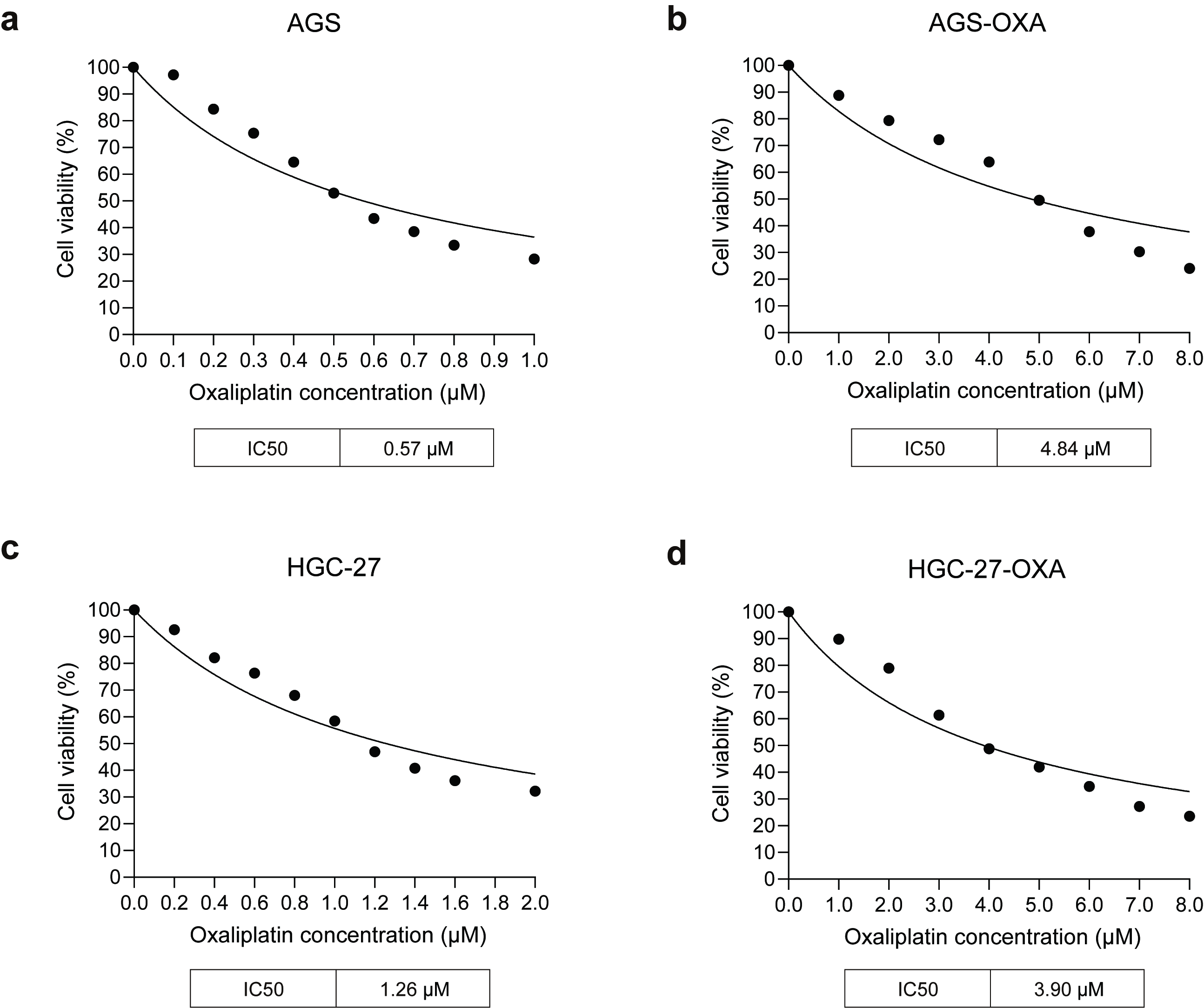

Supplement: Supplementary file 2 — Supplementary Figure 1 [file 41419_2022_5299_MOESM2_ESM.tif]

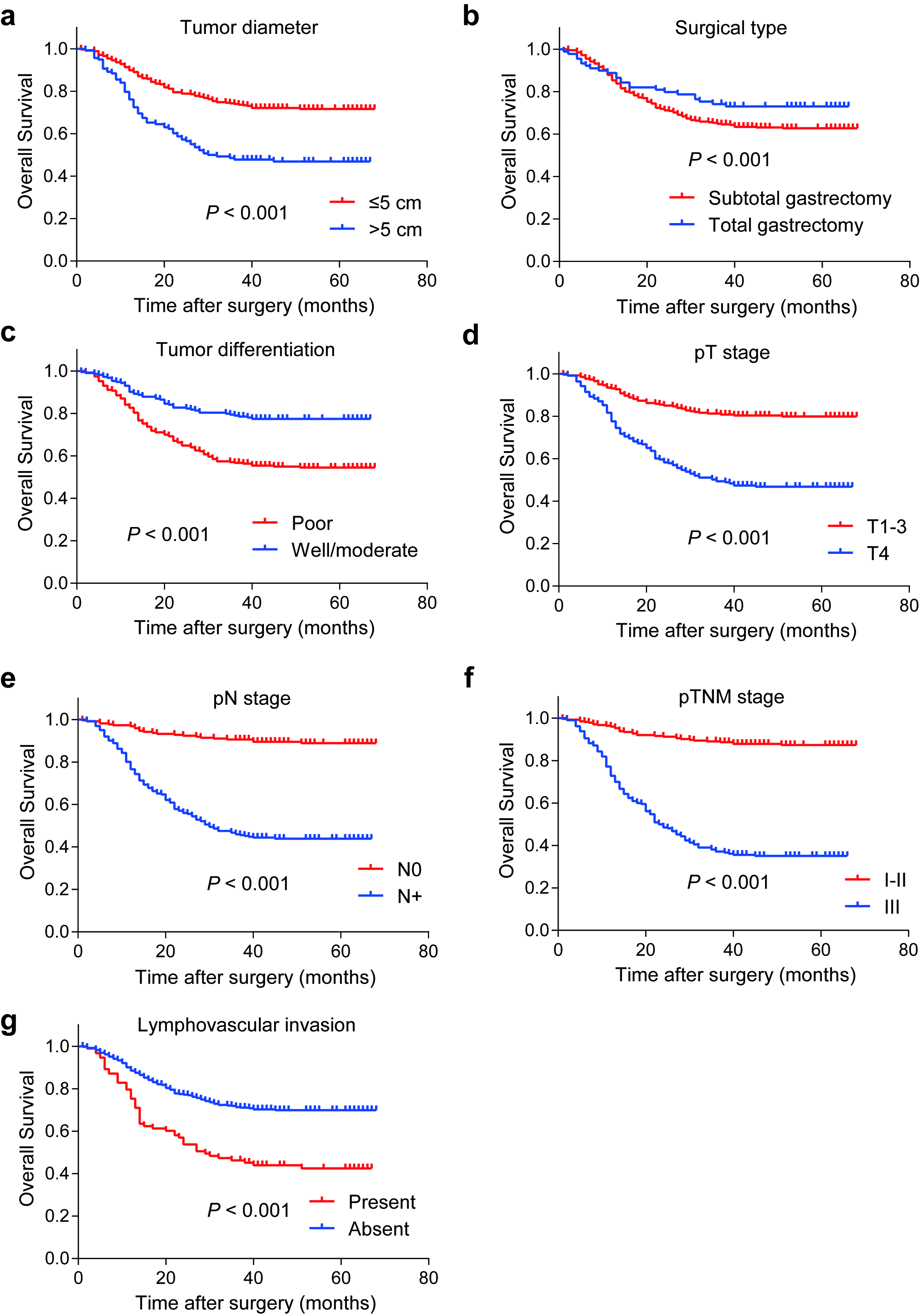

Supplement: Supplementary file 3 — Supplementary Figure 2 [file 41419_2022_5299_MOESM3_ESM.tif]

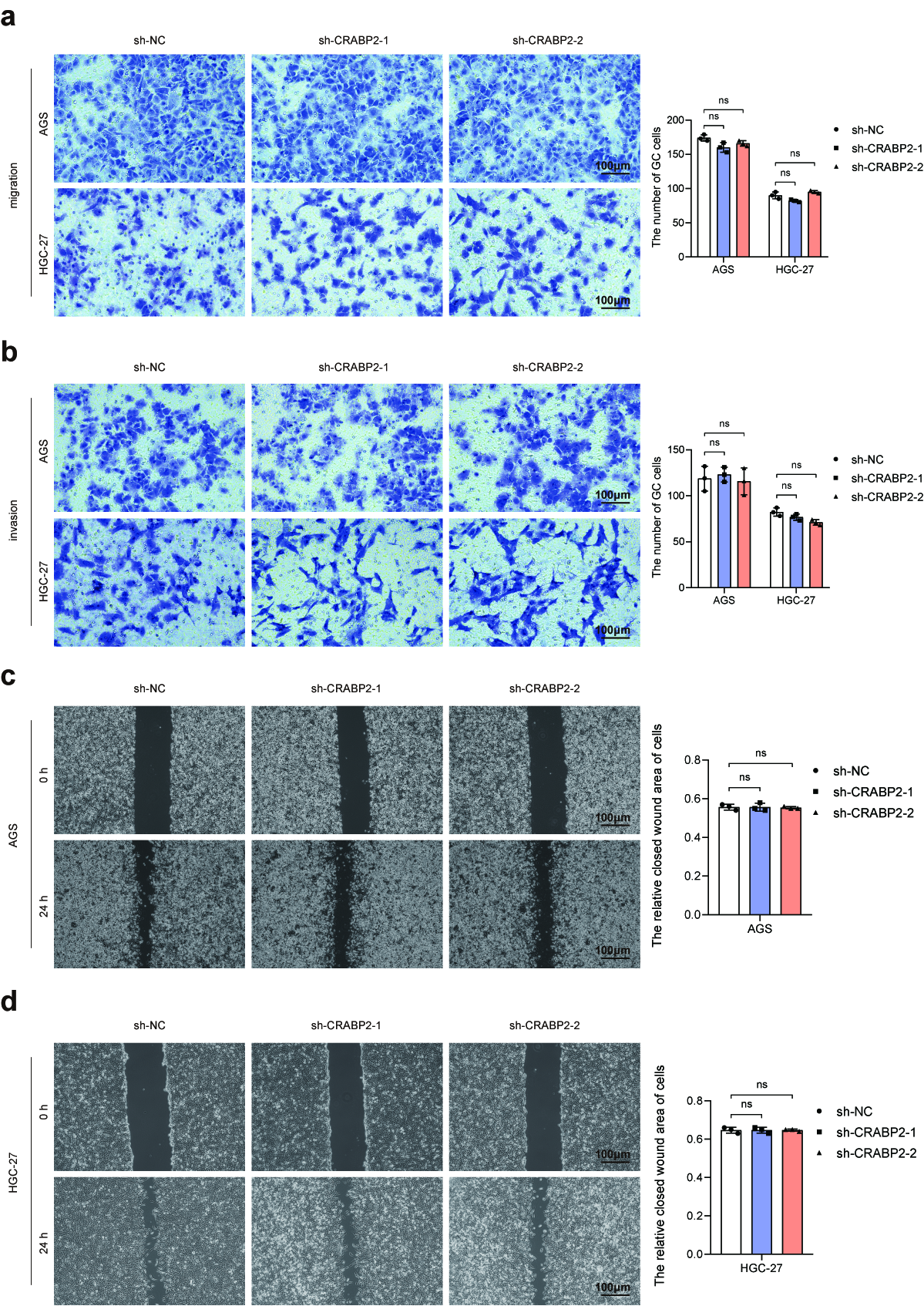

Supplement: Supplementary file 4 — Supplementary Figure 3 [file 41419_2022_5299_MOESM4_ESM.tif]

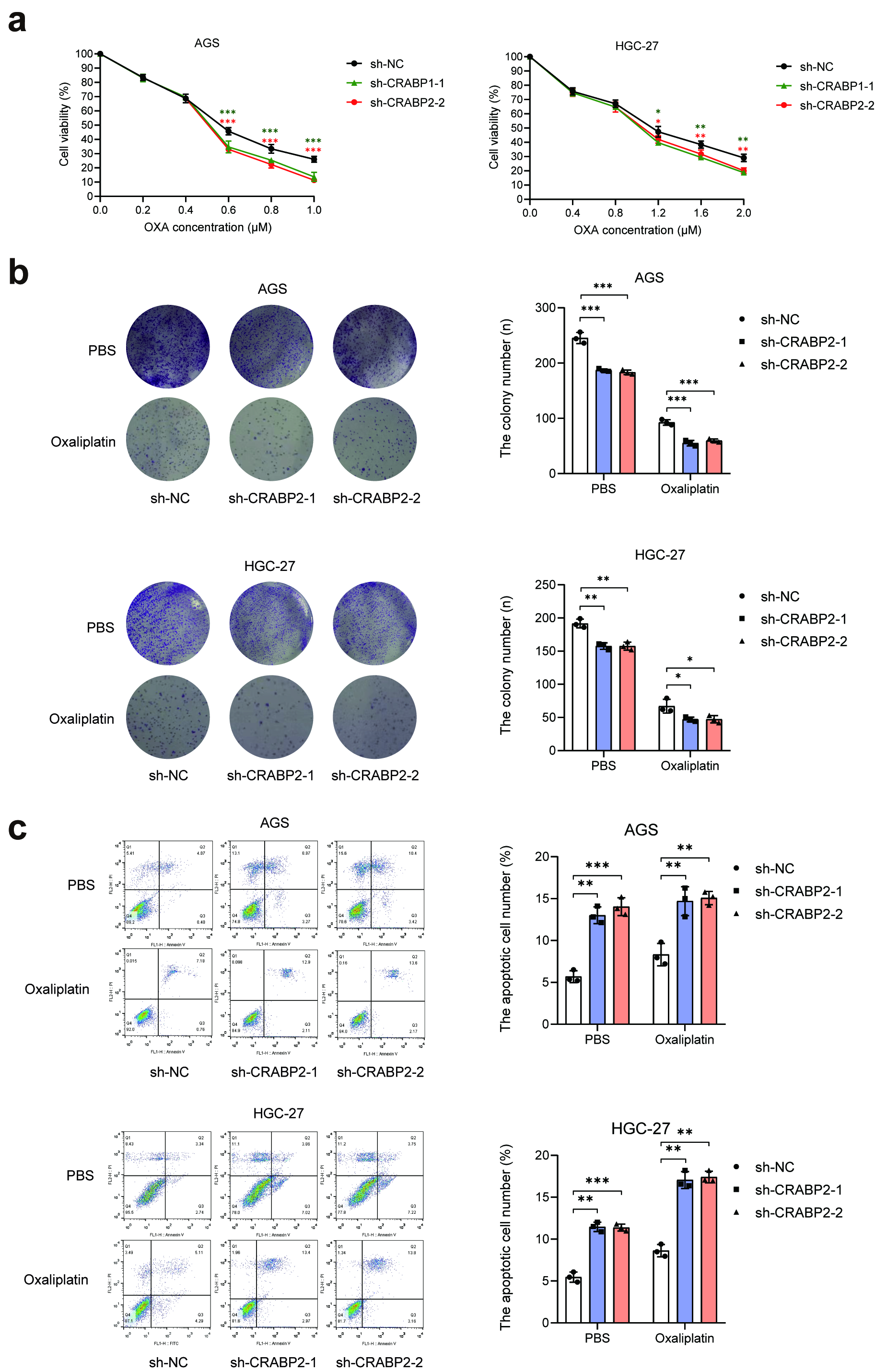

Supplement: Supplementary file 5 — Supplementary Figure 4 [file 41419_2022_5299_MOESM5_ESM.tif]

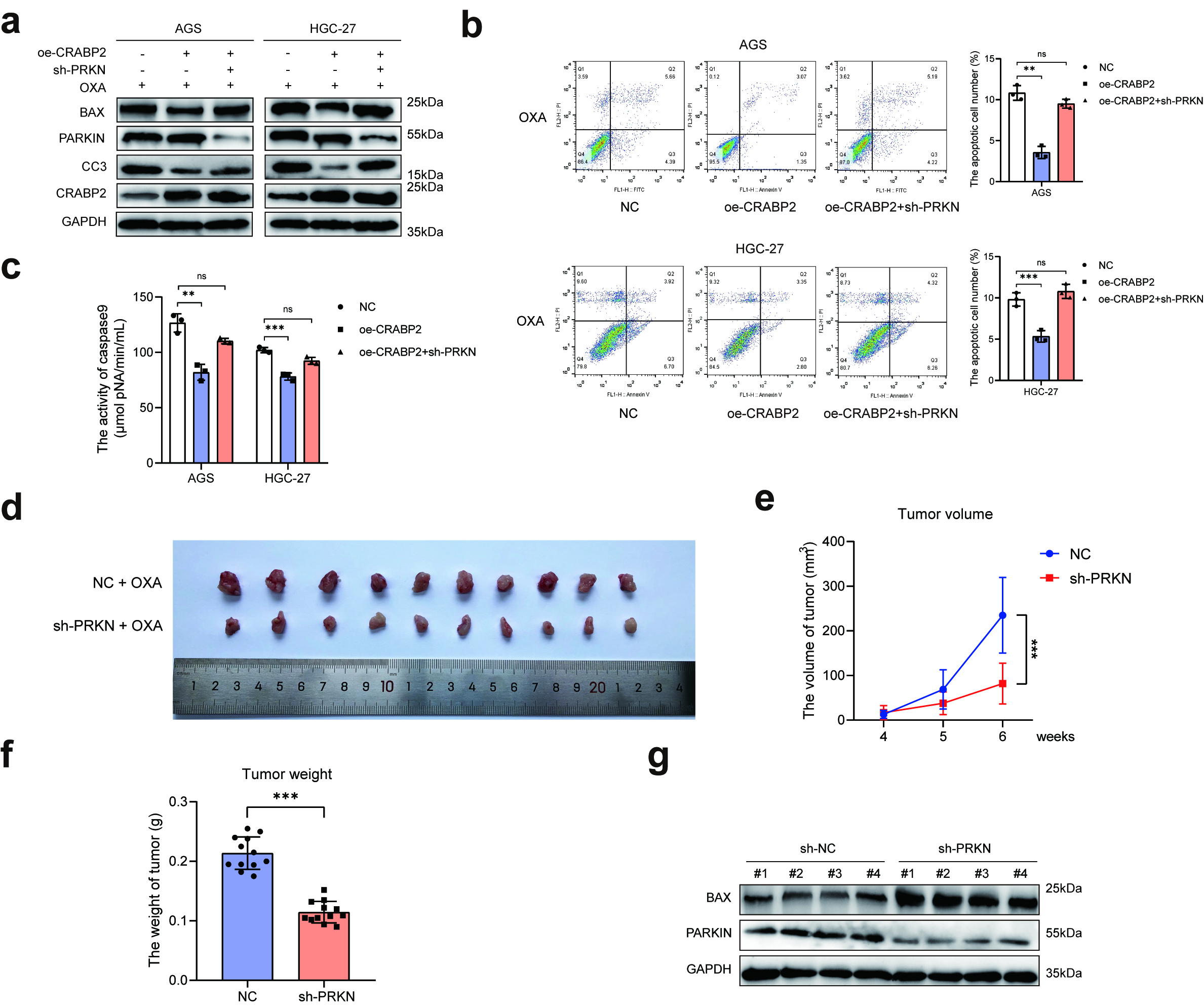

Supplement: Supplementary file 6 — Supplementary Figure 5 [file 41419_2022_5299_MOESM6_ESM.tif]

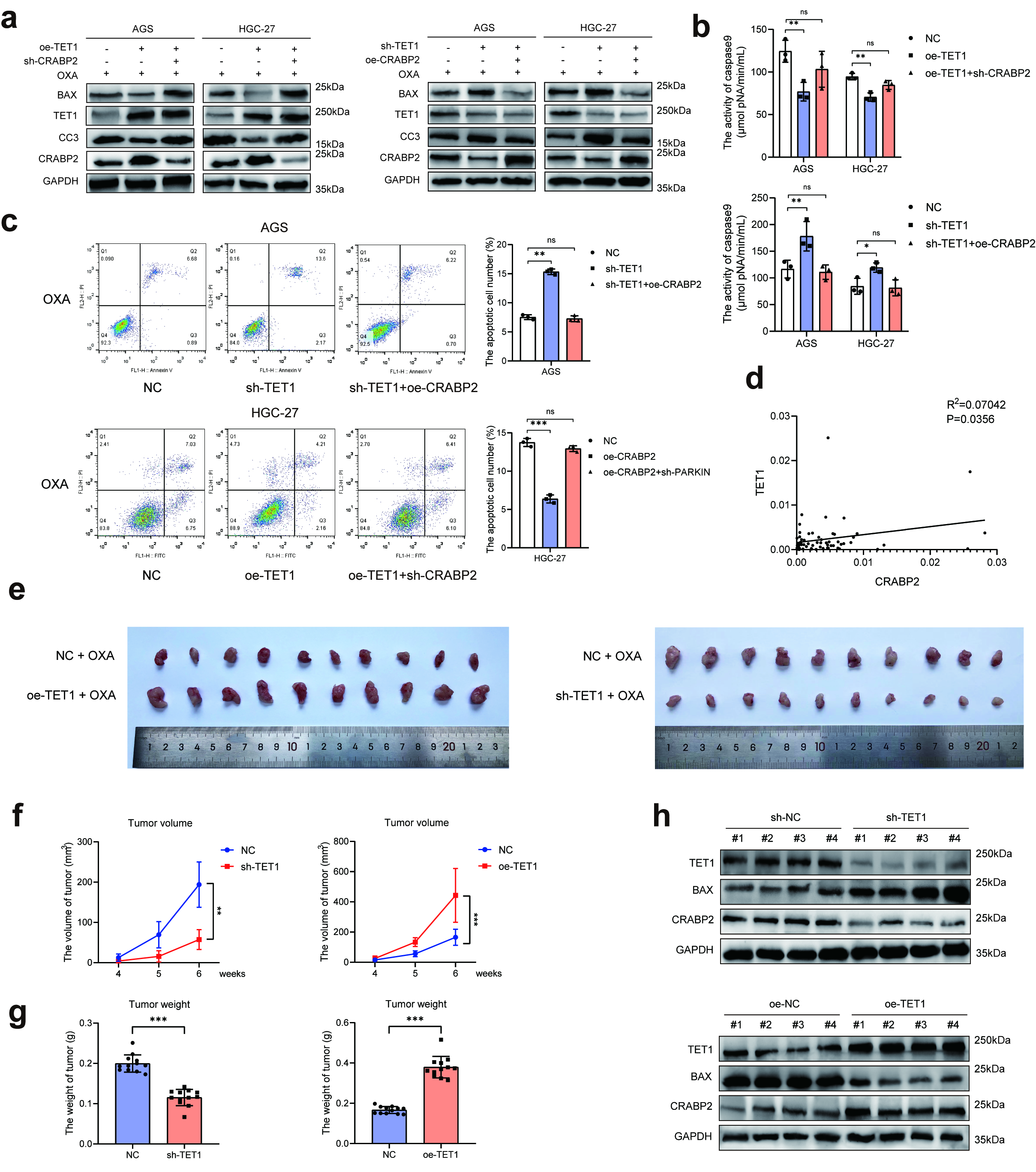

Supplement: Supplementary file 7 — Supplementary Figure 6 [file 41419_2022_5299_MOESM7_ESM.tif]

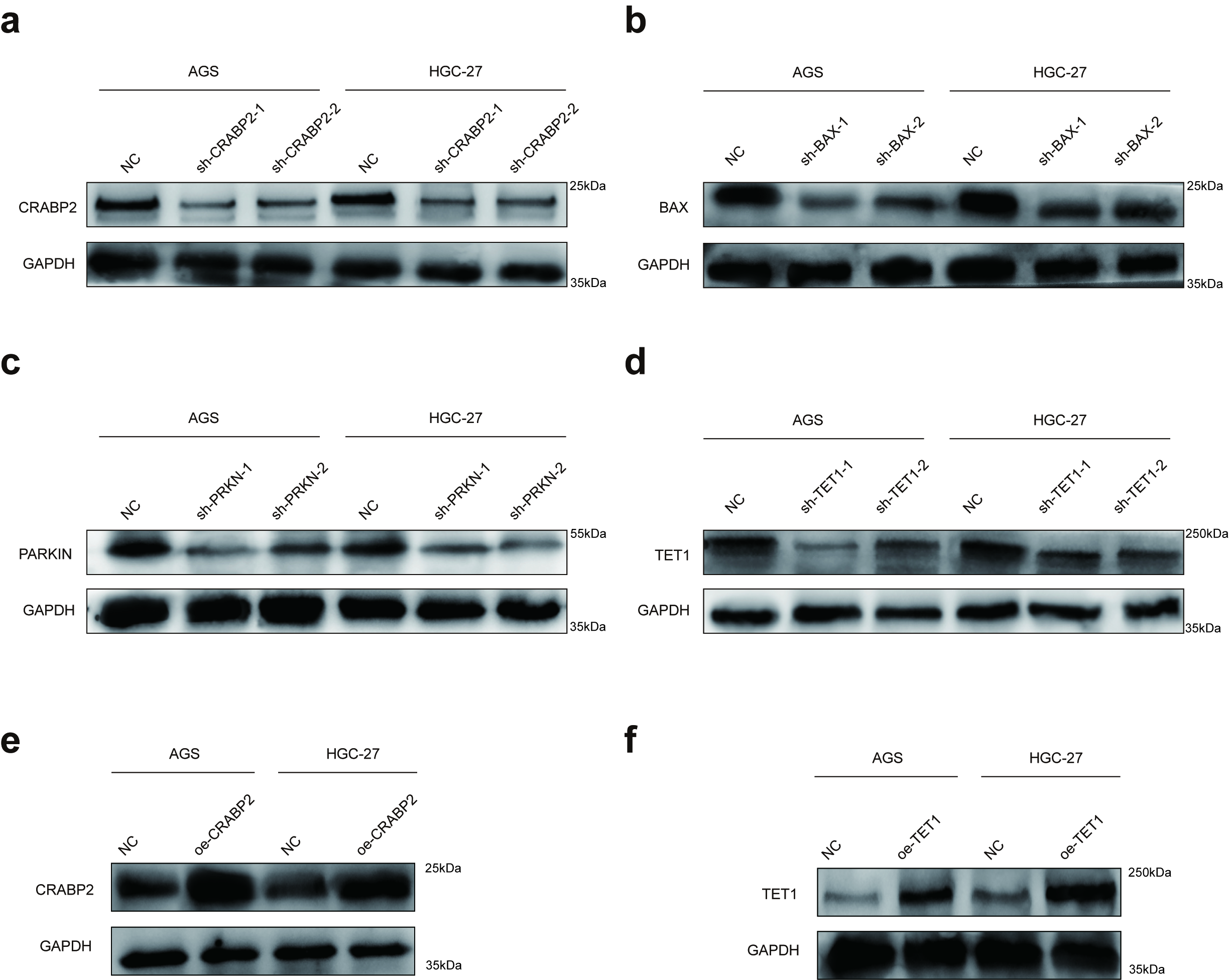

Supplement: Supplementary file 8 — Supplementary Figure 7 [file 41419_2022_5299_MOESM8_ESM.tif]
