# Supplementary material for: Upregulation of CRABP2 by TET1-mediated DNA hydroxymethylation attenuates mitochondrial apoptosis and promotes oxaliplatin resistance in gastric cancer
Source: Cell Death Dis. 2022 Oct 4;13(10):848. doi: 10.1038/s41419-022-05299-2 (PMC9532395; doi:10.1038/s41419-022-05299-2)
Supplement: Supplementary file 12 — Supplementary Table 4 [file 41419_2022_5299_MOESM12_ESM.docx]

**Supplementary Table 4. The primer sequences used in this study**

| Category |  | Primer sequences |
| --- | --- | --- |
| Genes |  |  |
| CRABP2 | Forward | CTGGGGGTGAATGTGATGCT |
|  | Reverse | CCTCAAACTCCTCCCCAACC |
| GAPDH | Forward | GATTTGGTCGTATTGGGCGC |
|  | Reverse | TTCCCGTTCTCAGCCTTGAC |
| sh-RNAs |  |  |
| sh-CRABP2-1# |  | 5’- AGGAGGGAGACACTTTCTACA -3’ |
| sh-CRABP2-2# |  | 5’- CTGTAGCCTATACAGTTTAGA -3’ |
| sh-BAX-1# |  | 5’- GGCCTGAGTCCAGCTCTTTAA -3’ |
| sh-BAX-2# |  | 5’- CAGCTCTGAGCAGATCATGAA -3’ |
| sh-PRKN-1# |  | 5’- GGATCAGCAGAGCATTGTTCA -3’ |
| sh-PRKN-2# |  | 5’- GGTCAAGAAATGAATGCAACT -3’ |
| sh-TET1-1# |  | 5’- GCTACGAAGCACCTCTCTTAG -3’ |
| sh-TET1-2# |  | 5’- GCAAATCAACAGGAAGTTTCT -3’ |
| sh-negative control |  | 5’- TTCTCCGAACGTGTCACGT -3’ |
