# Supplementary material for: Upregulation of CRABP2 by TET1-mediated DNA hydroxymethylation attenuates mitochondrial apoptosis and promotes oxaliplatin resistance in gastric cancer
Source: Cell Death Dis. 2022 Oct 4;13(10):848. doi: 10.1038/s41419-022-05299-2 (PMC9532395; doi:10.1038/s41419-022-05299-2)

F1 e

CRABP2

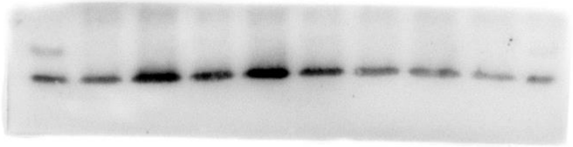

GAPDH

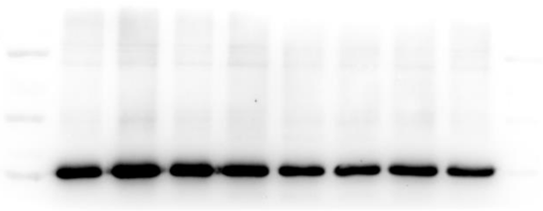

F1 K

CRABP2

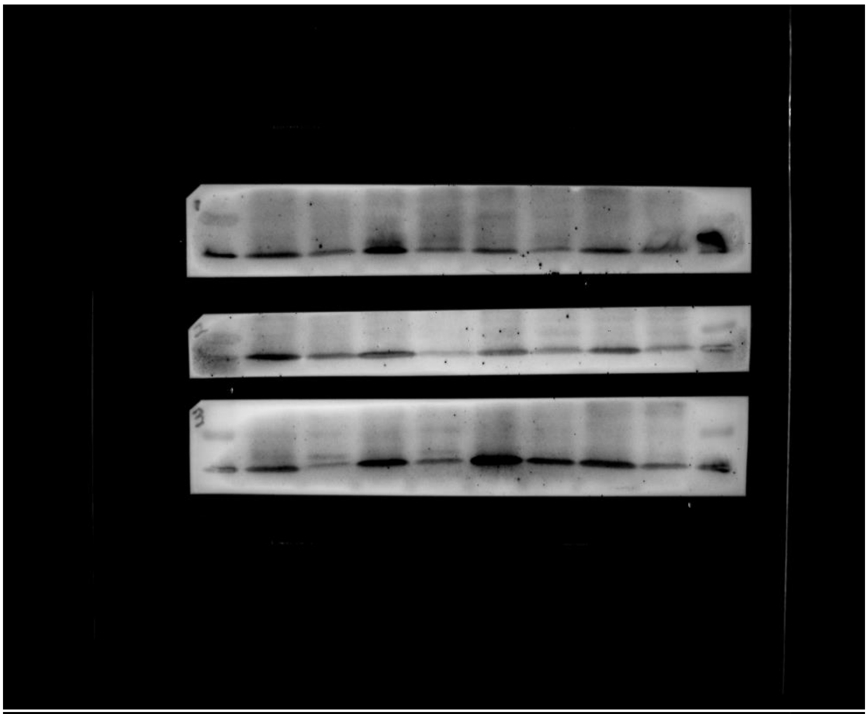

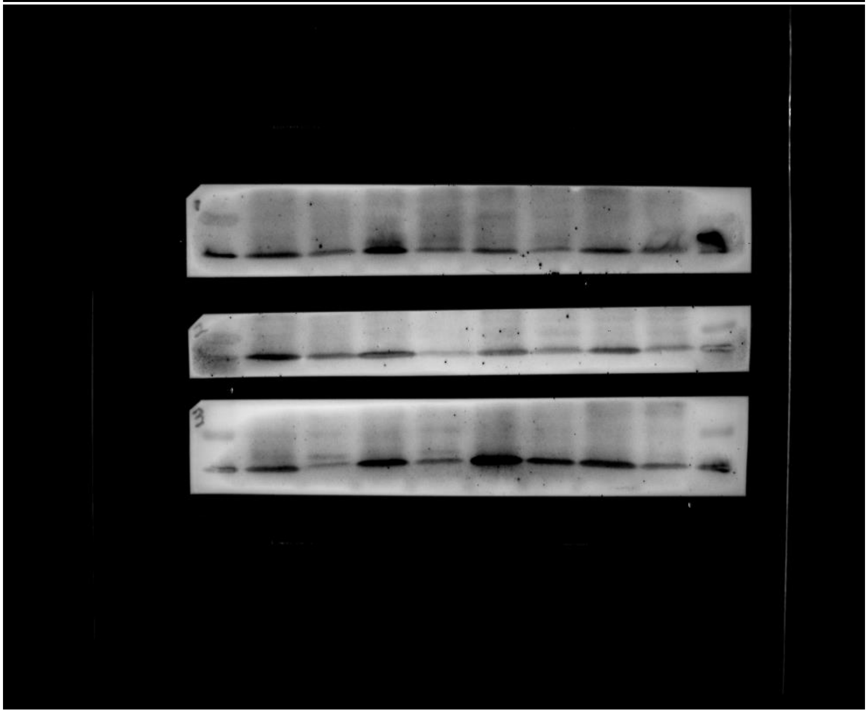

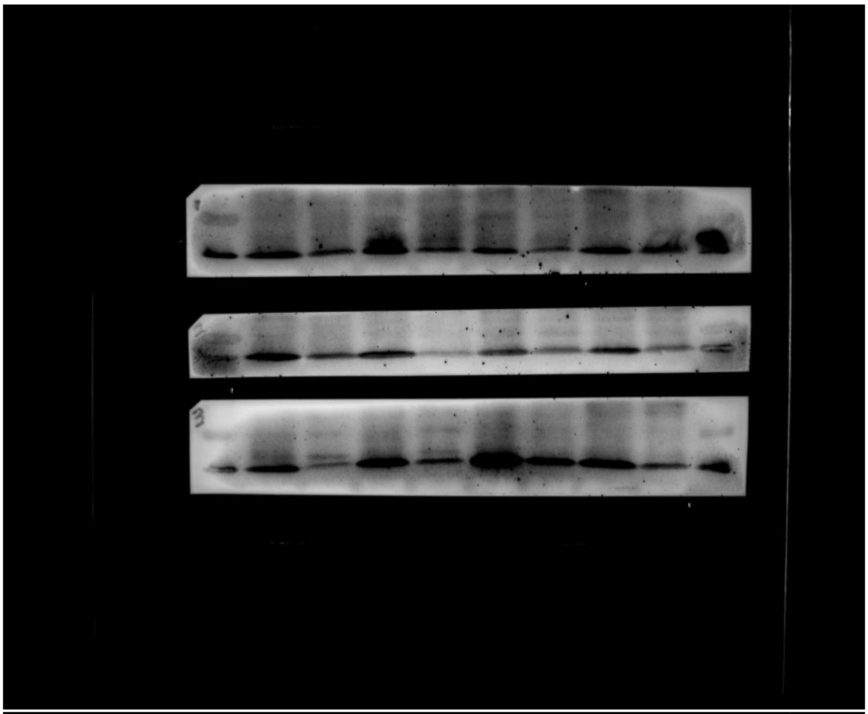
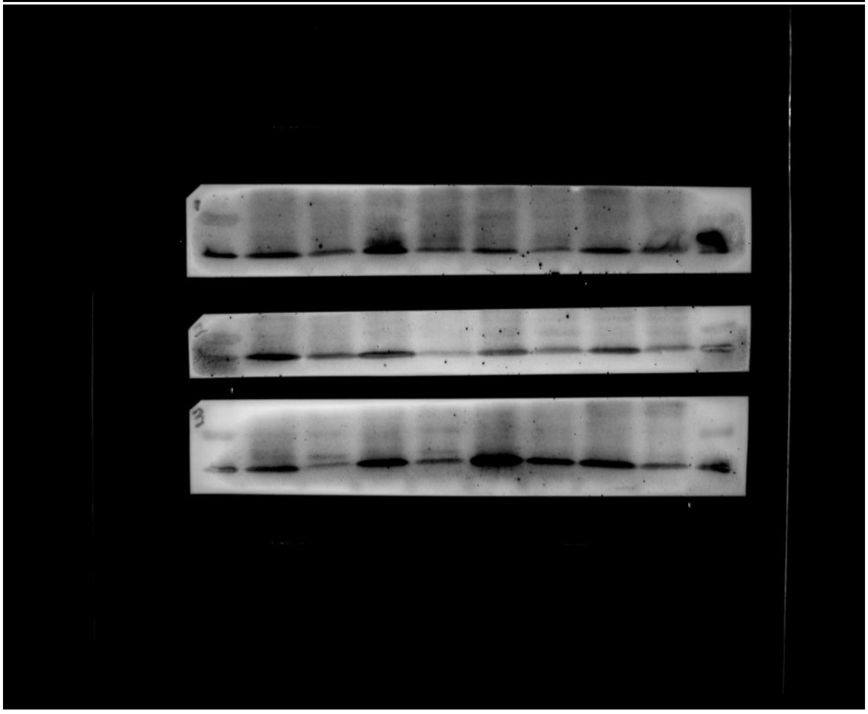

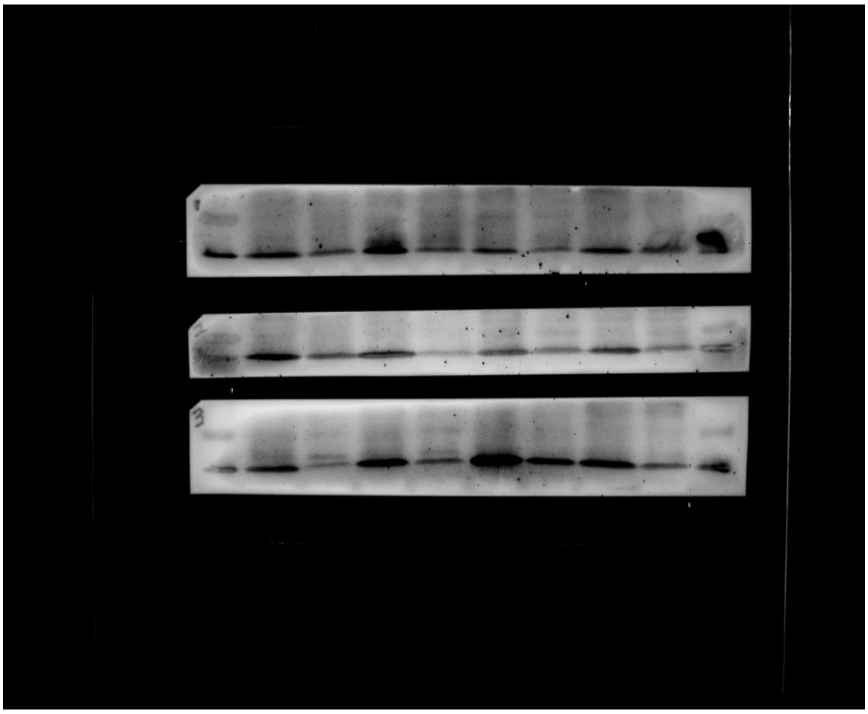

GAPDH

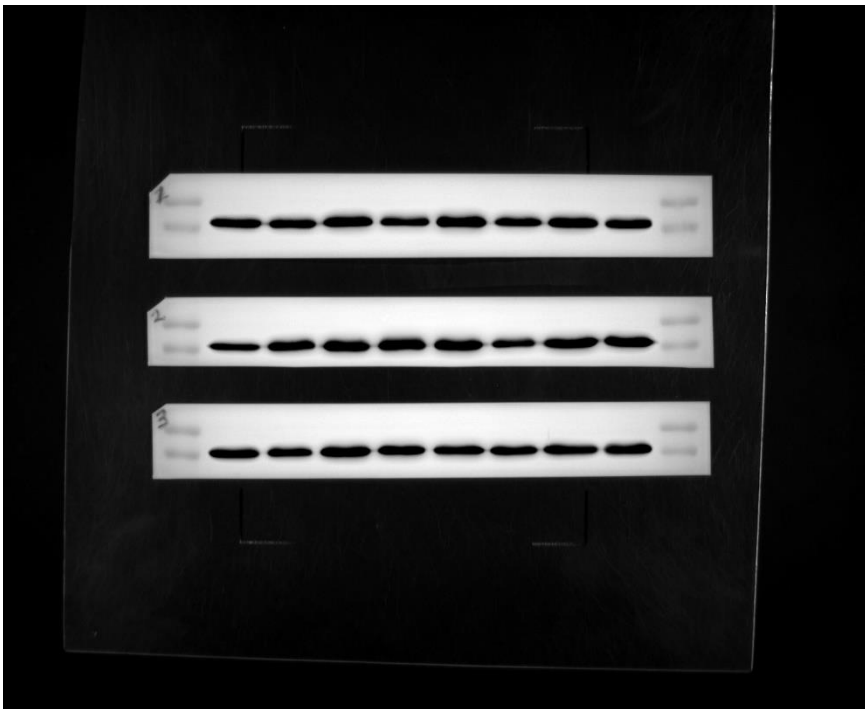

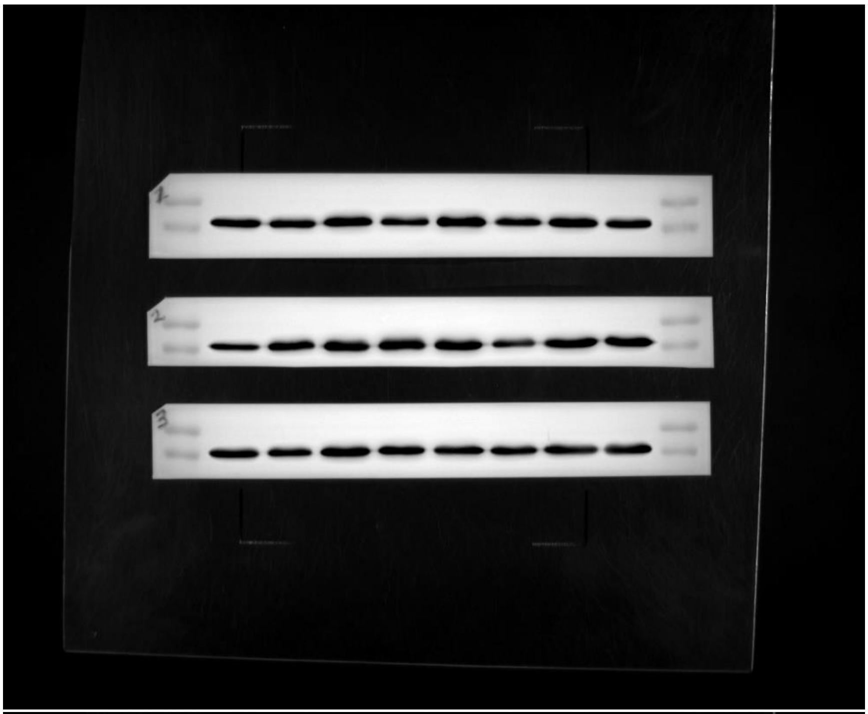
1
2
3

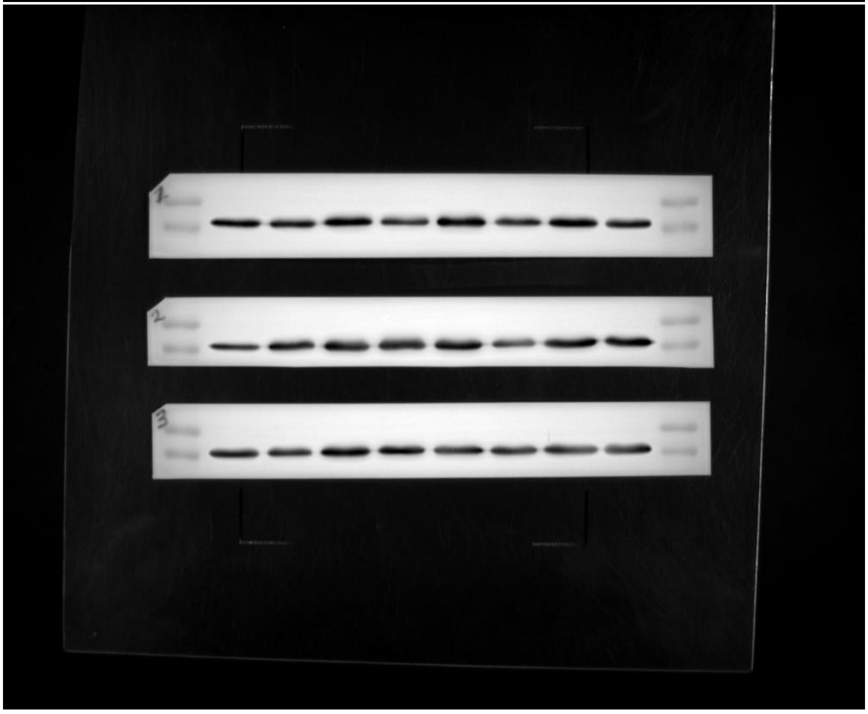
1
2
3

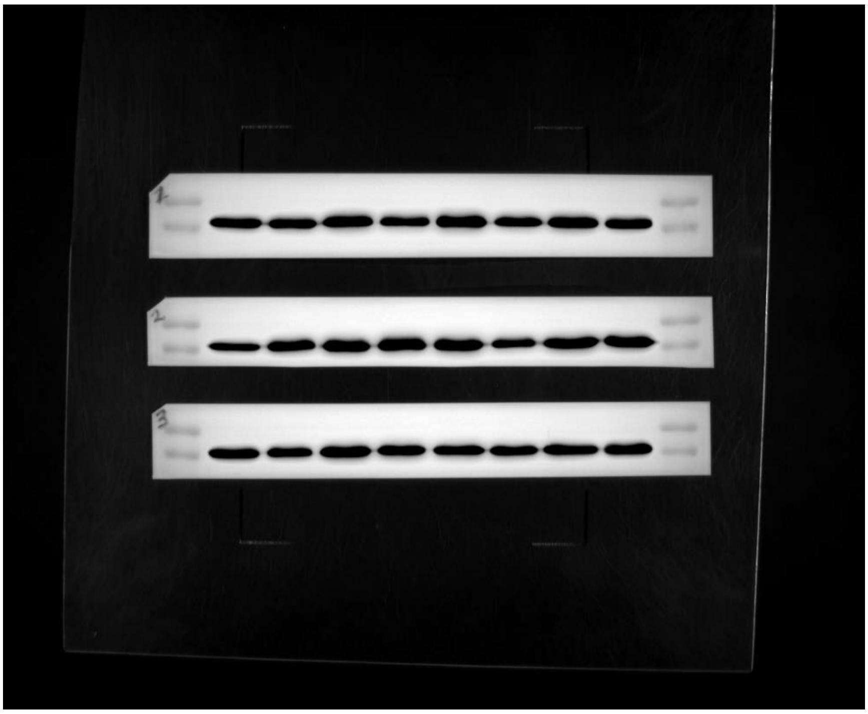

F3 B

BAX

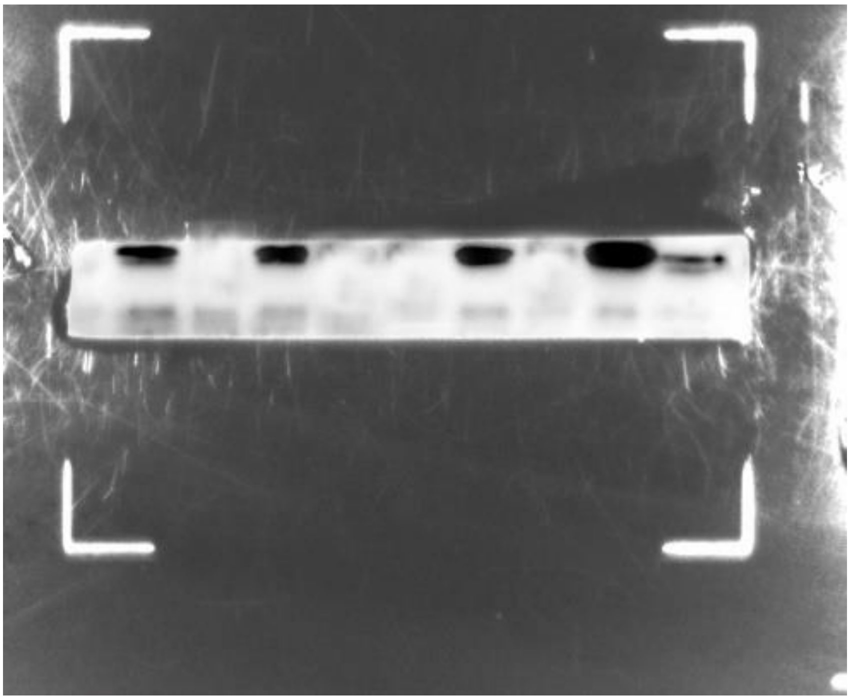

PARKIN

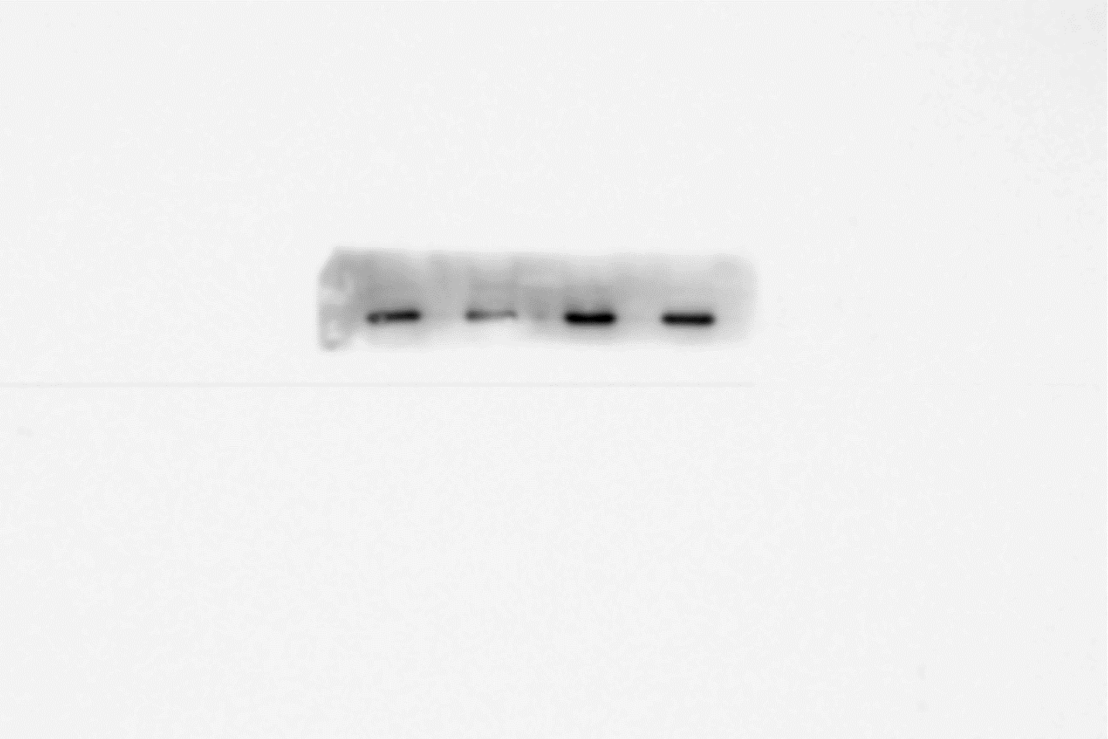

CRABP2

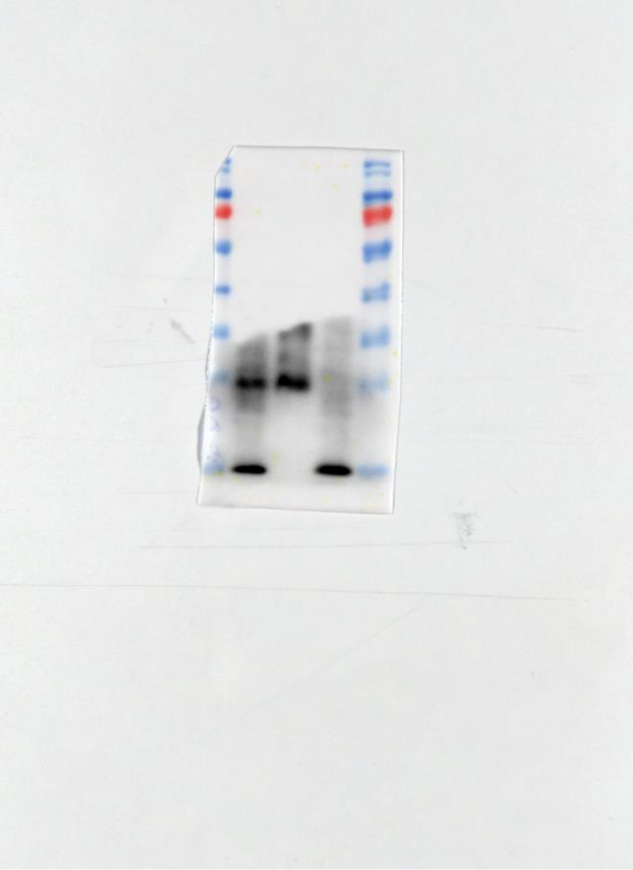

F3 C

GST

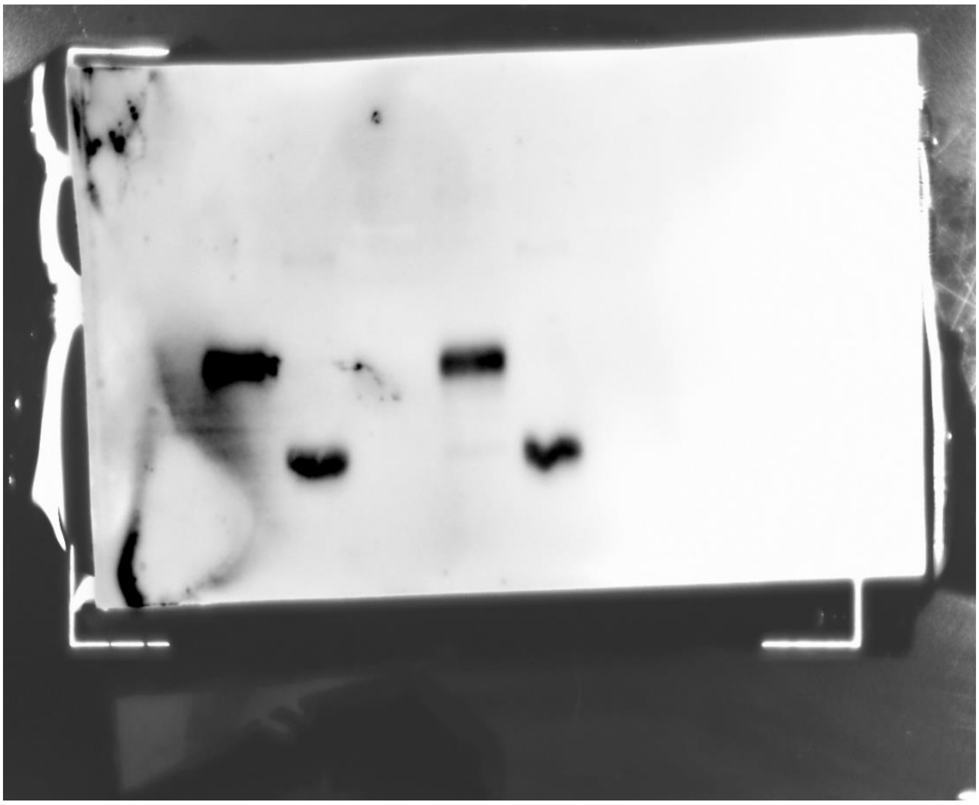

FLAG

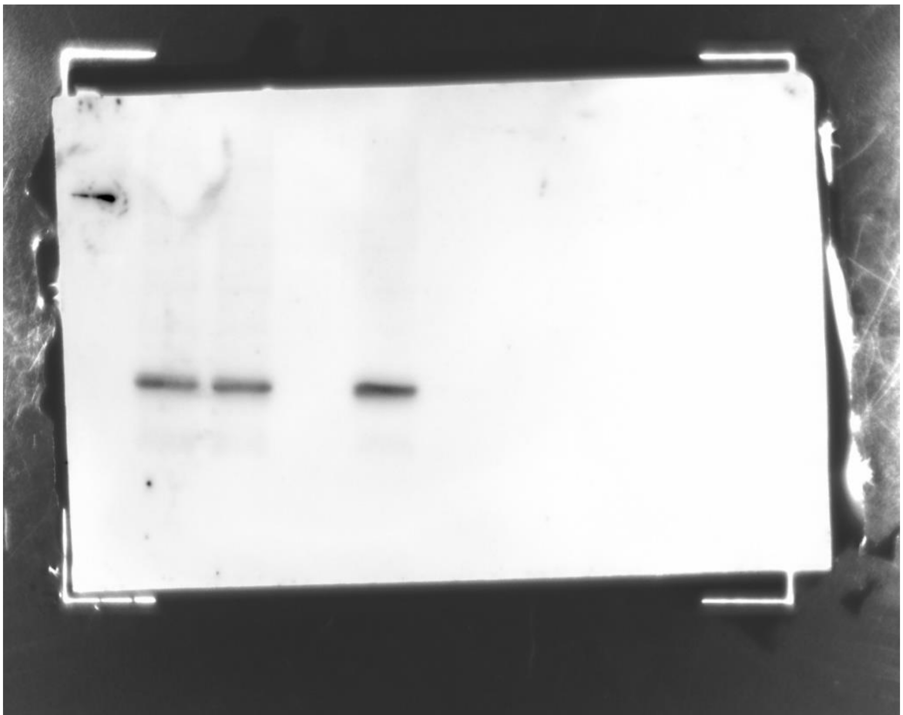

F3 D

Up BAX

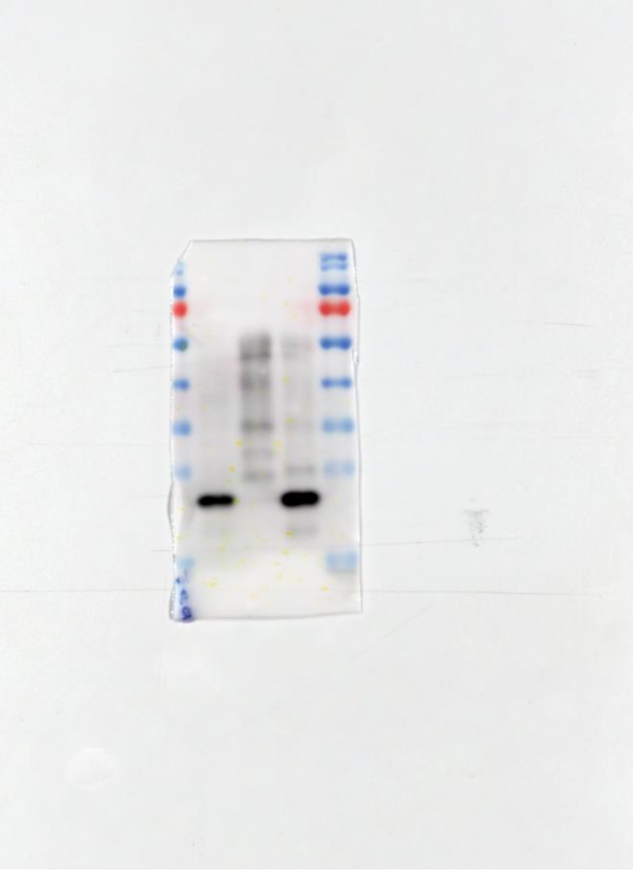

CRABP2

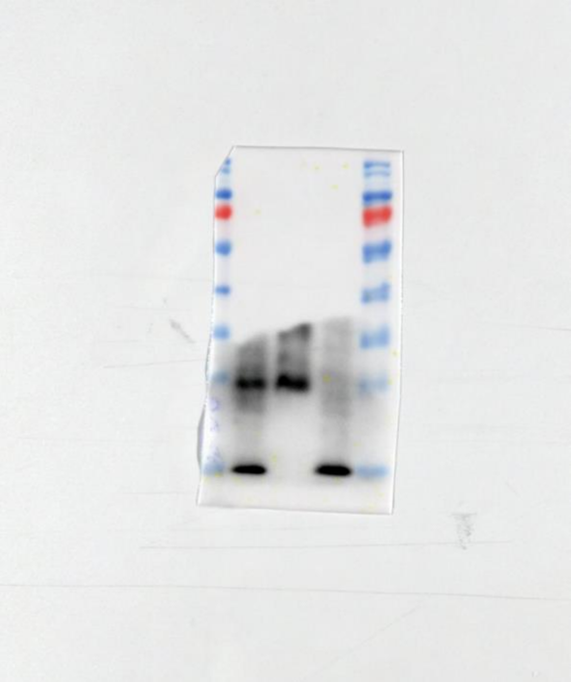

Under

CRABP2

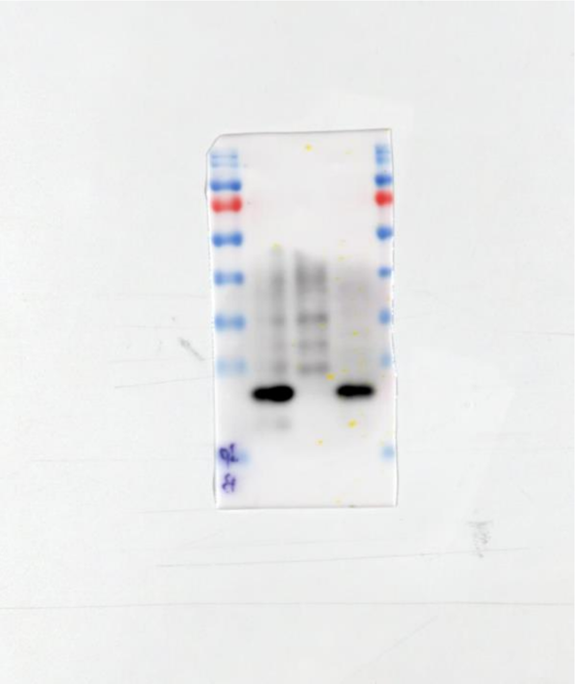

PARKIN

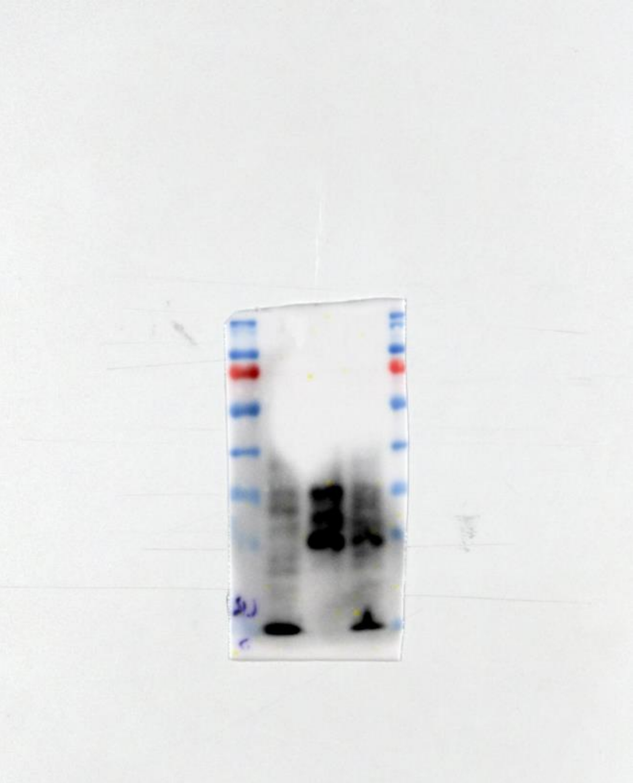

F3 E

BAX

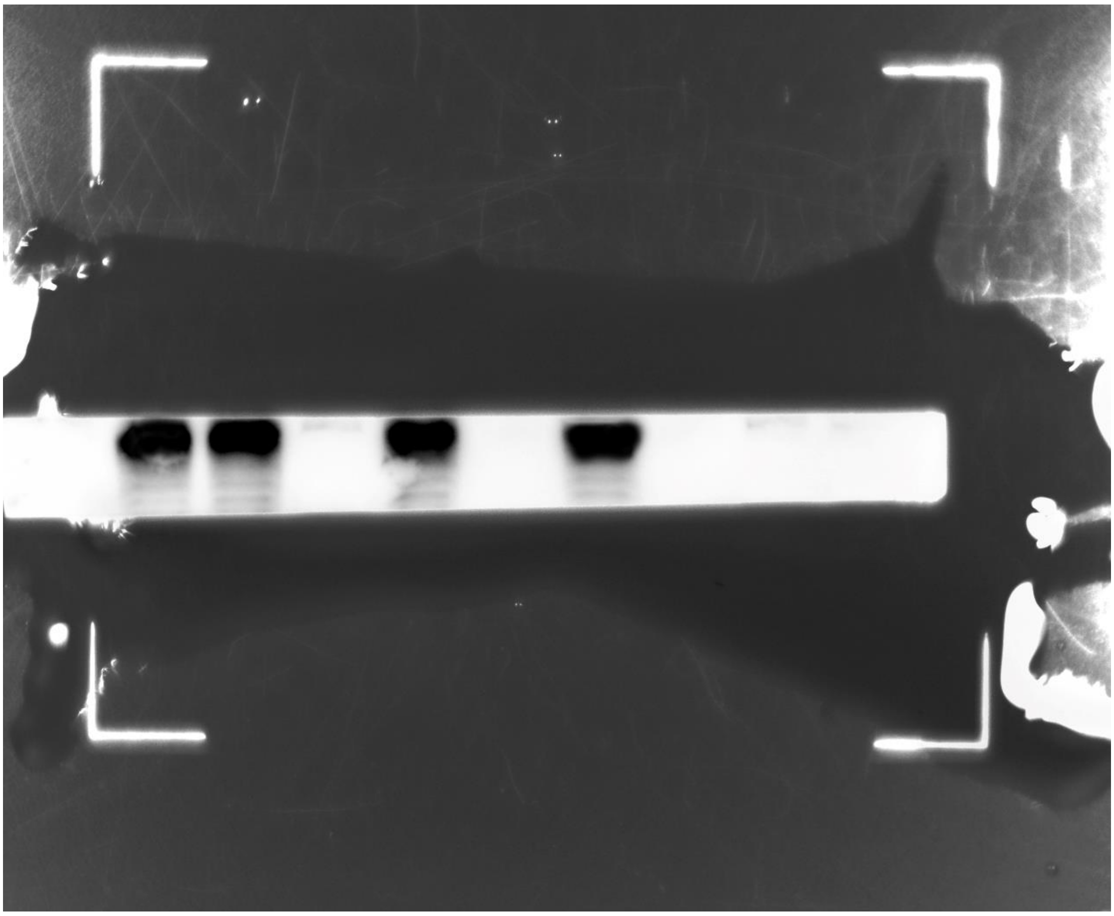

CRABP2

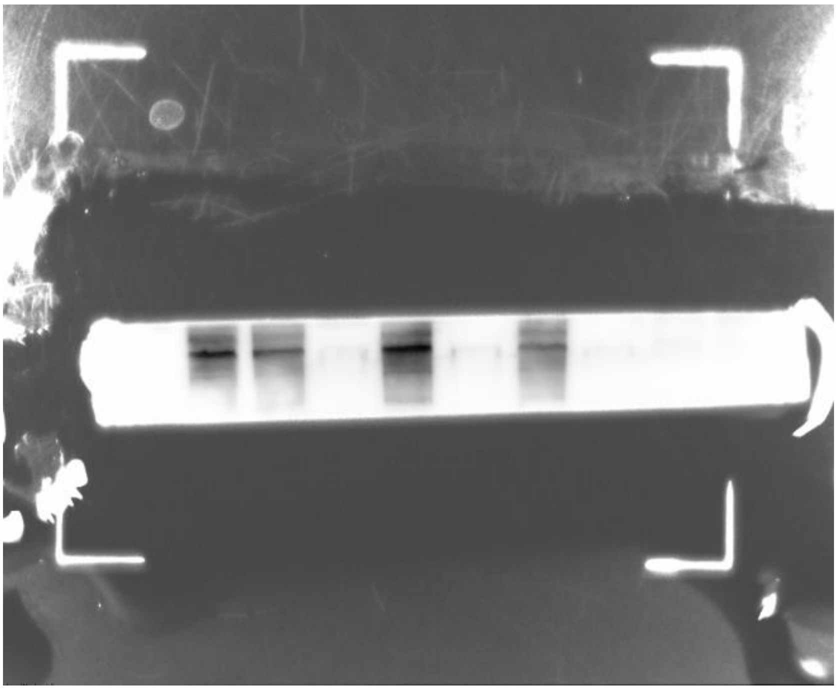

GAPDH

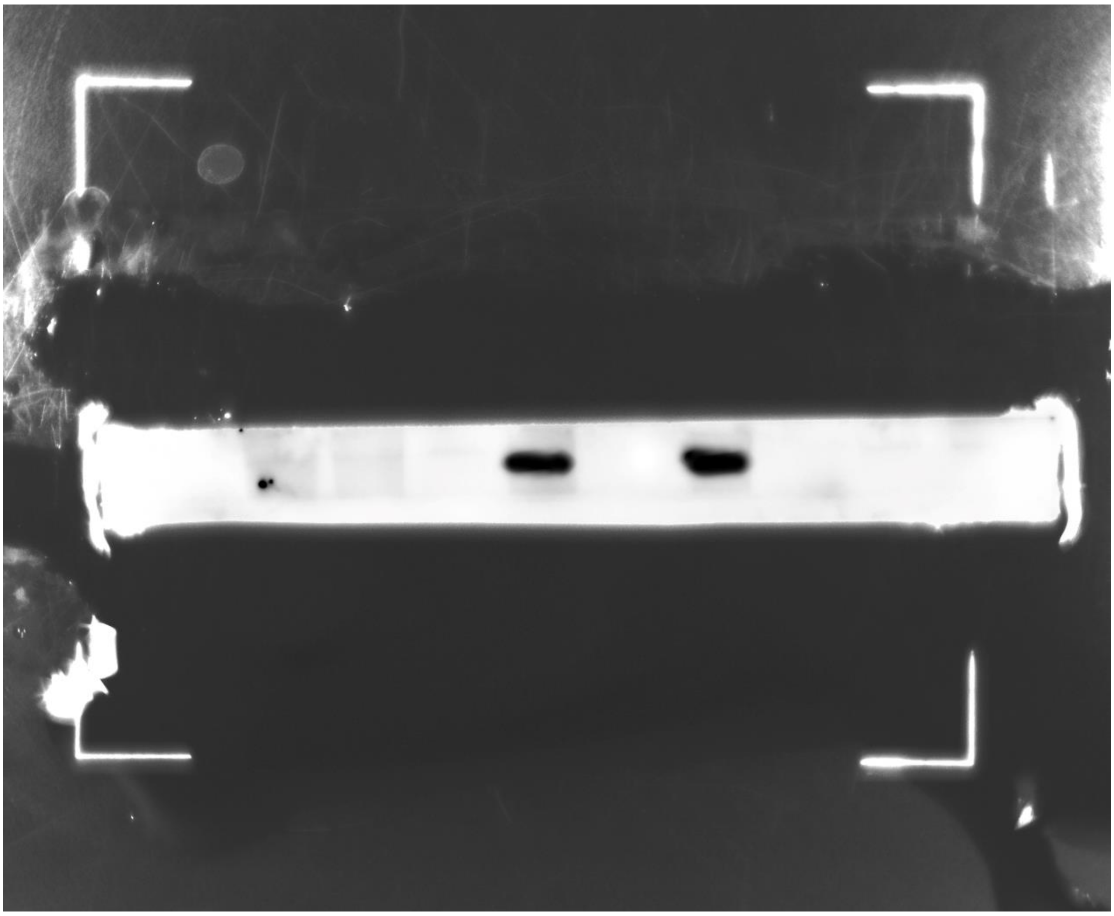

PARKIN

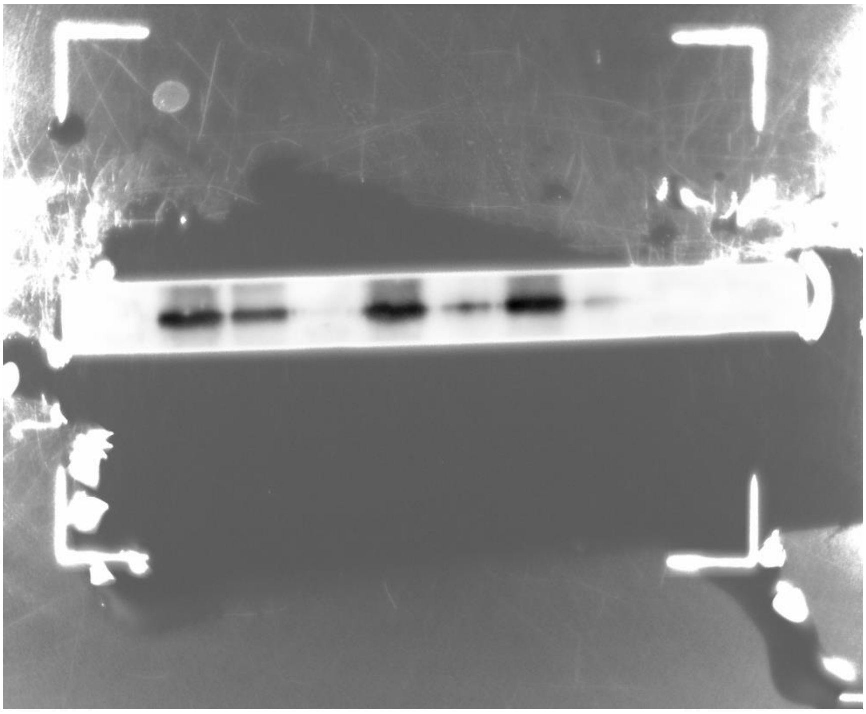

F3 F

BAX

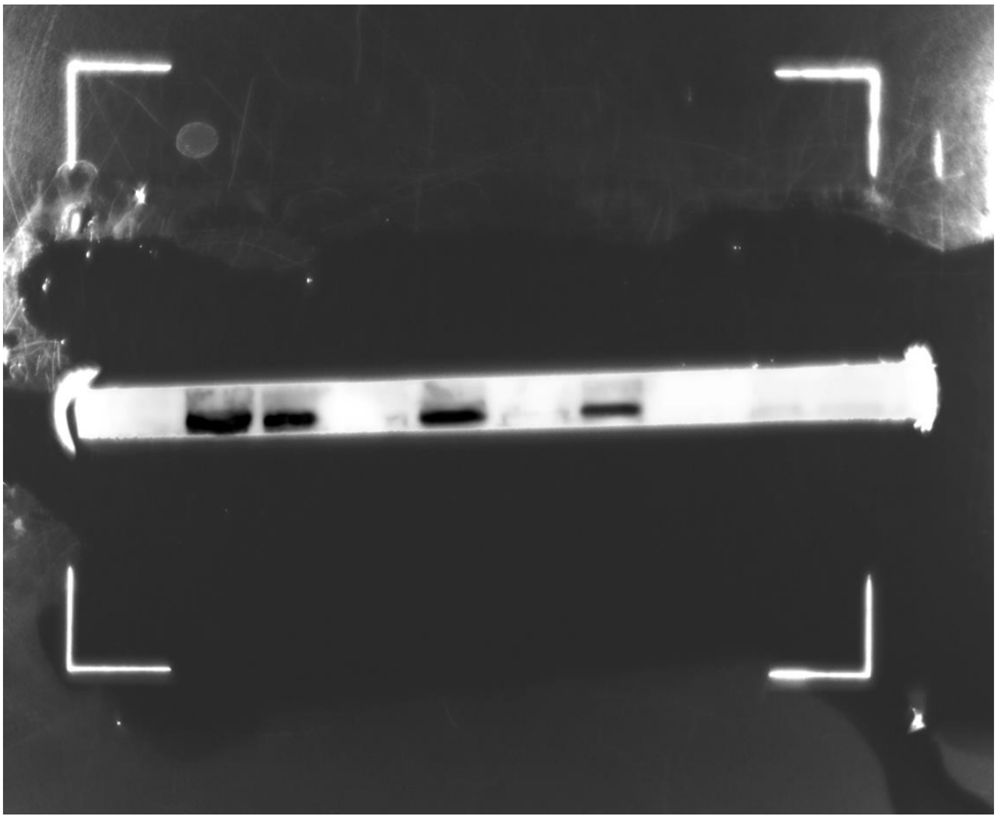

CRABP2

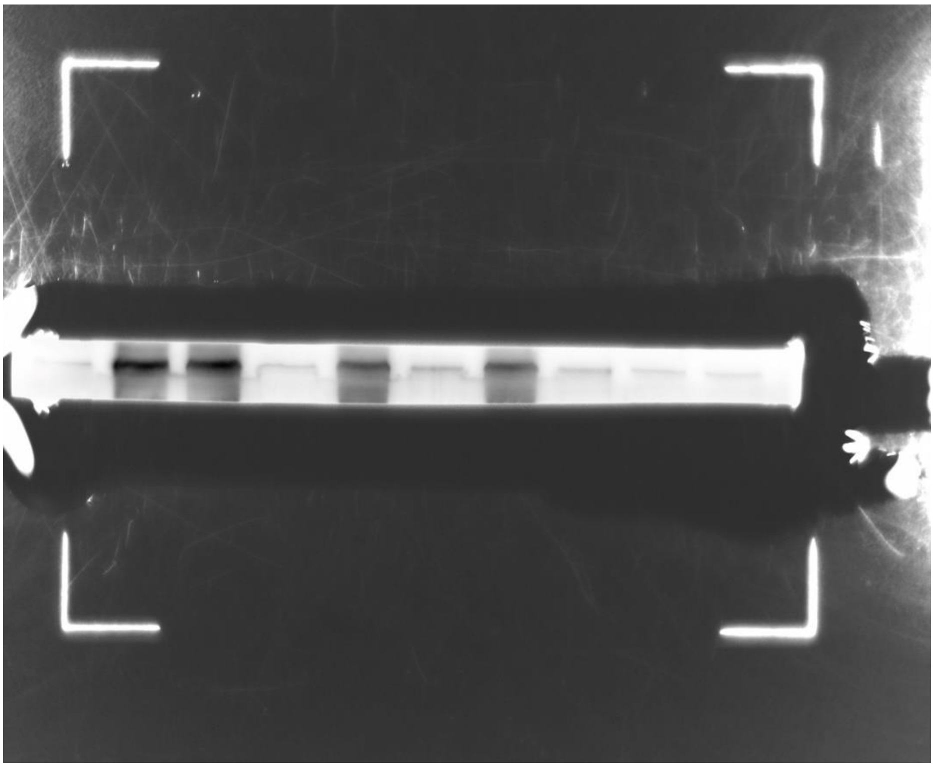

GAPDH

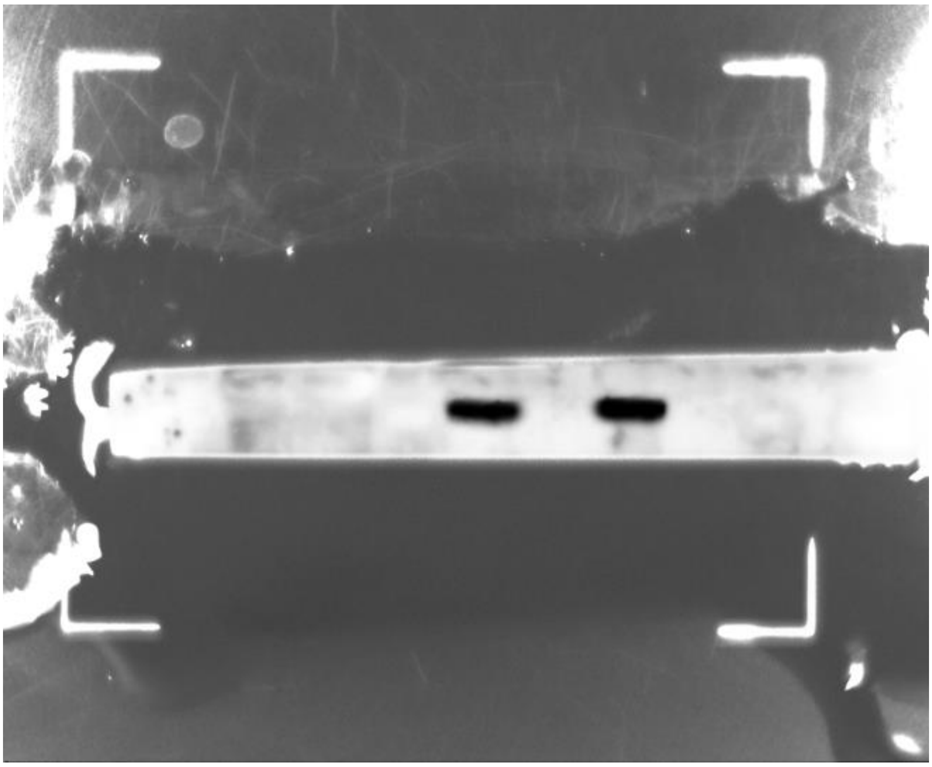

PARKIN

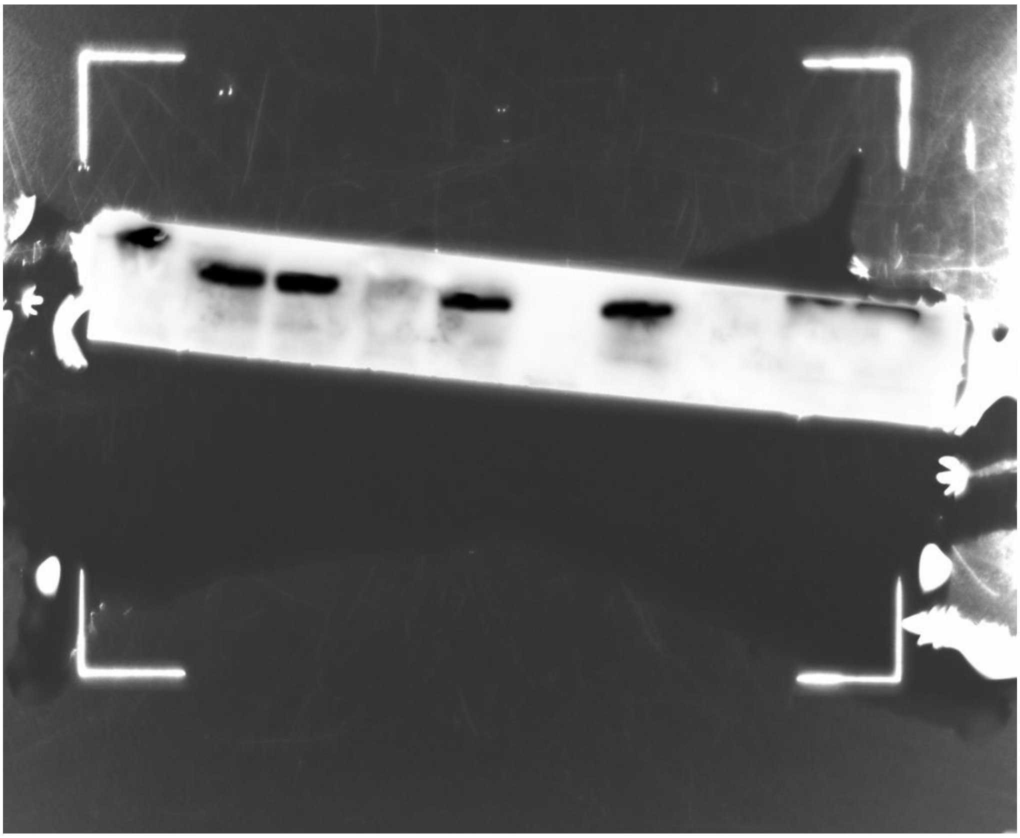

F3 G

BAX

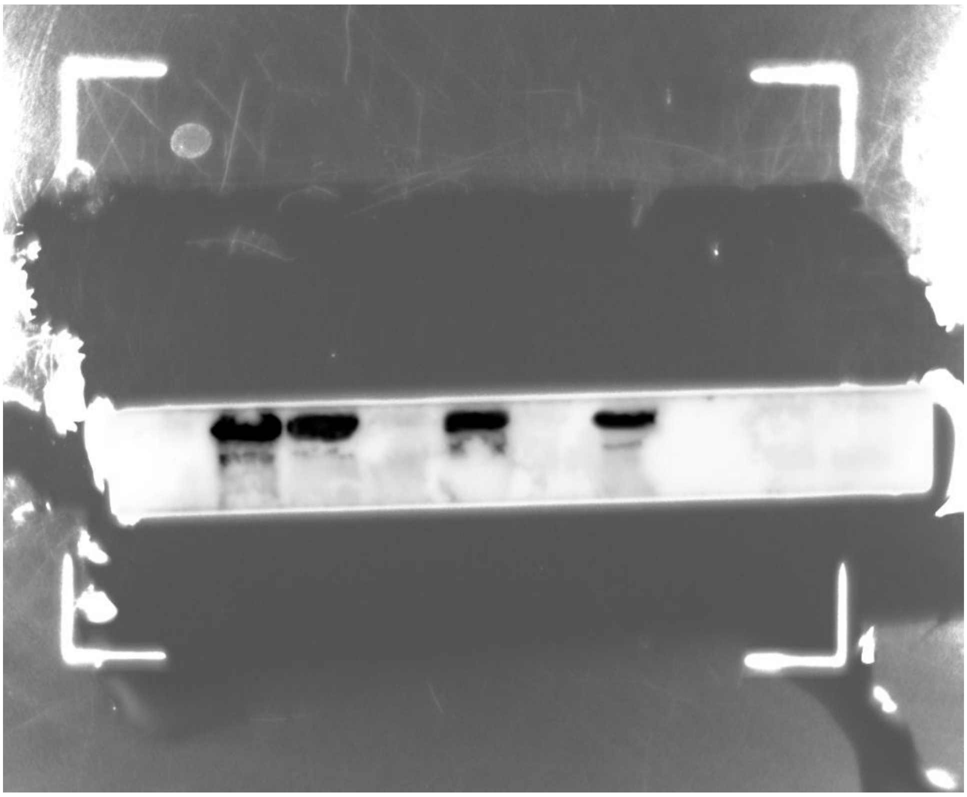

CRABP2

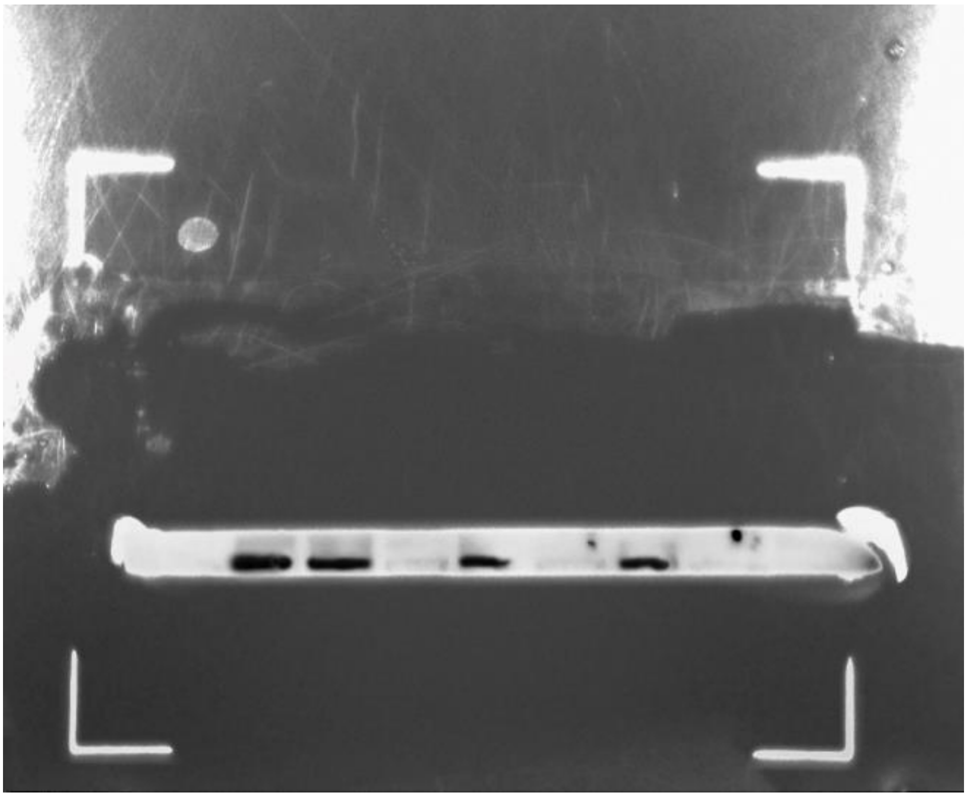

PARKIN

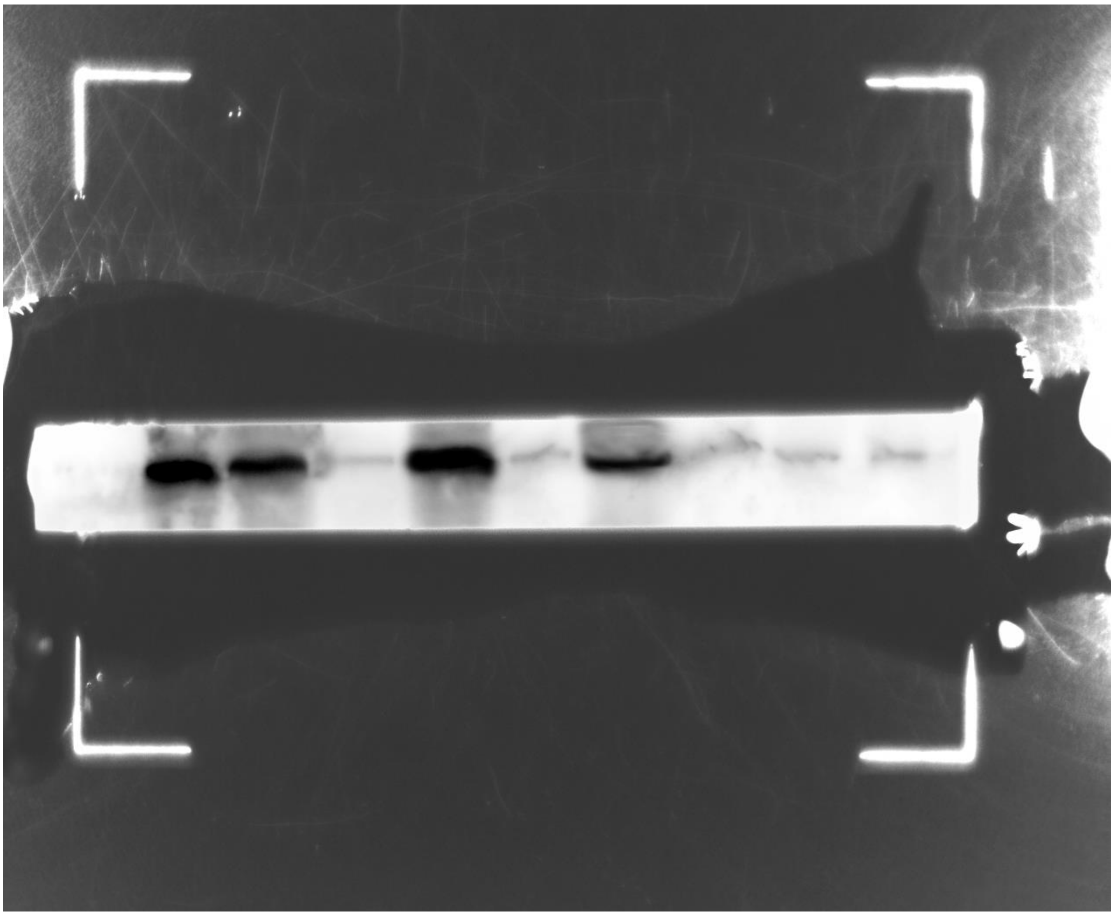

GAPDH

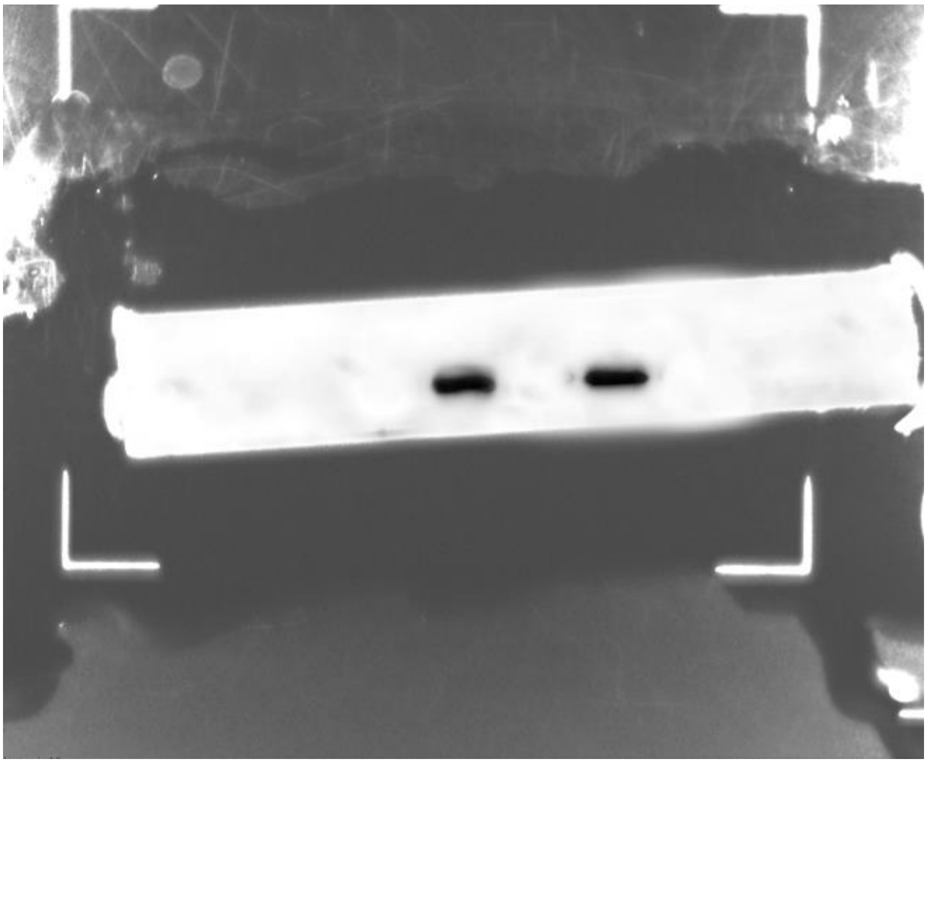

F3 J F3 K

Bax

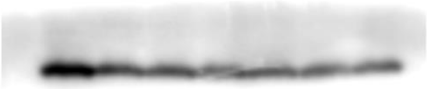

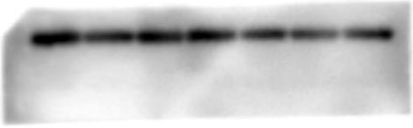

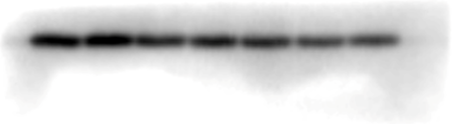

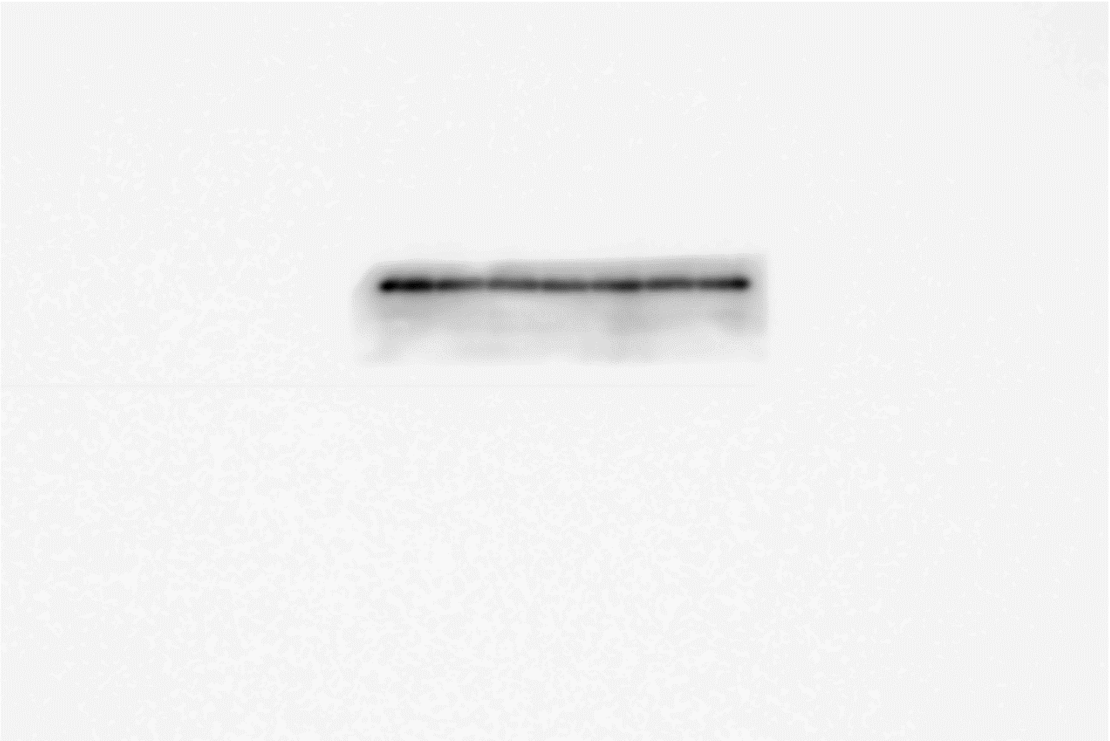

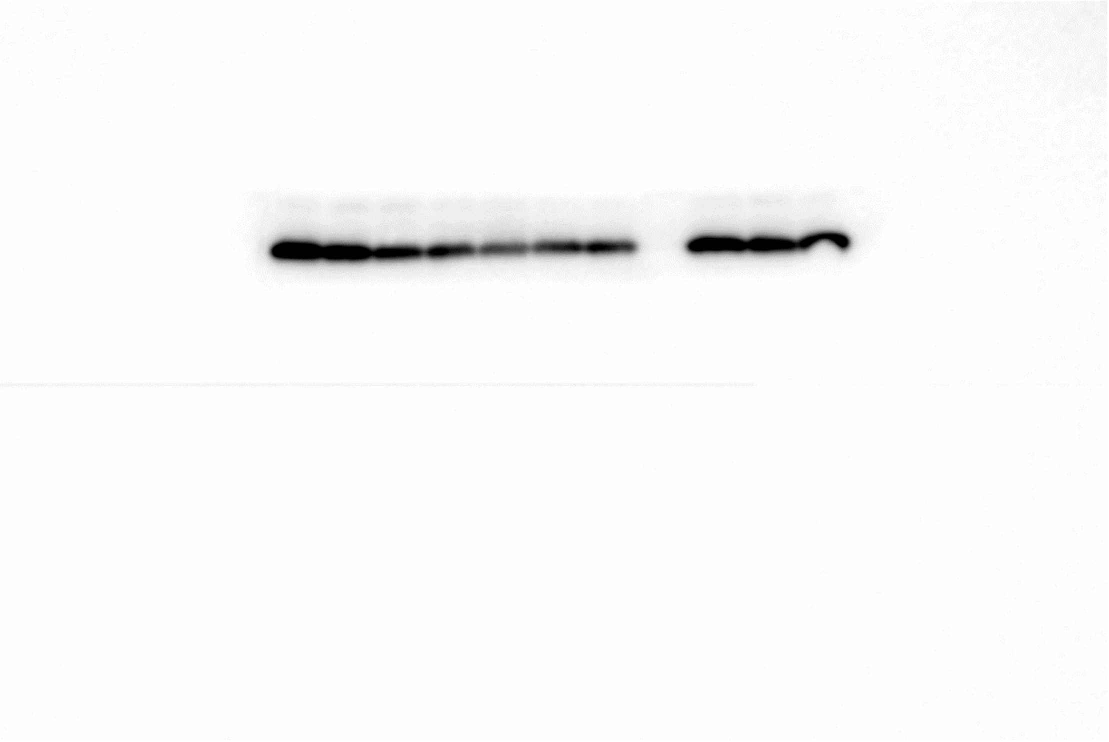

ACTIN

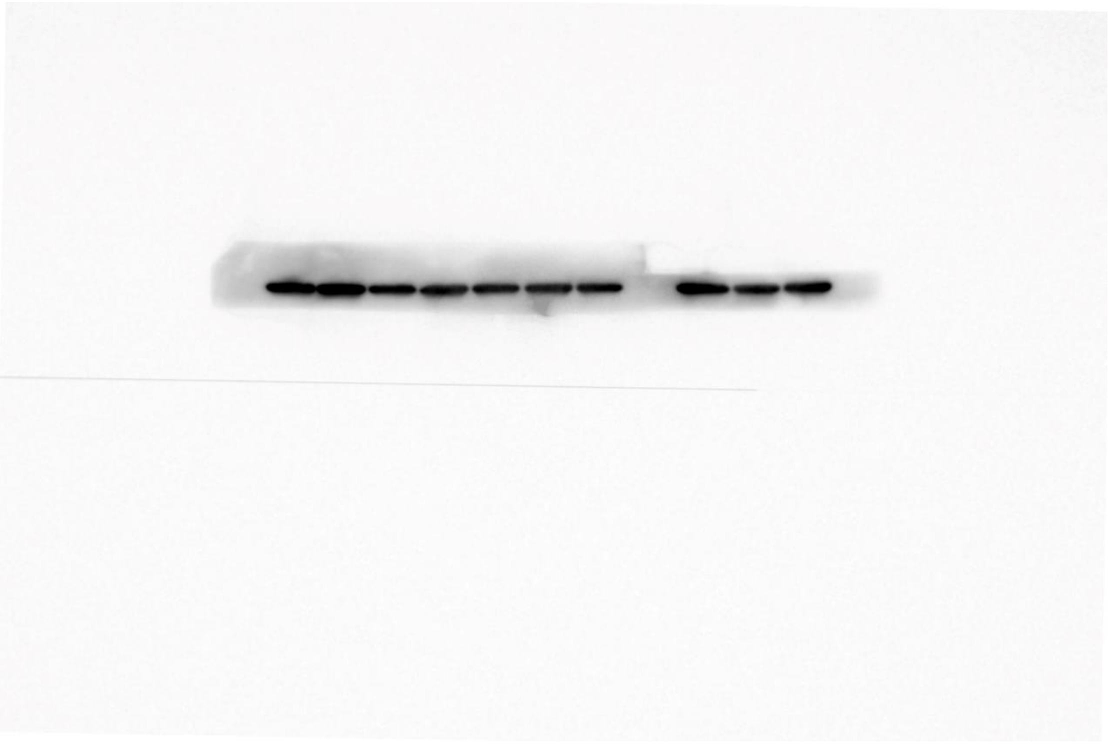

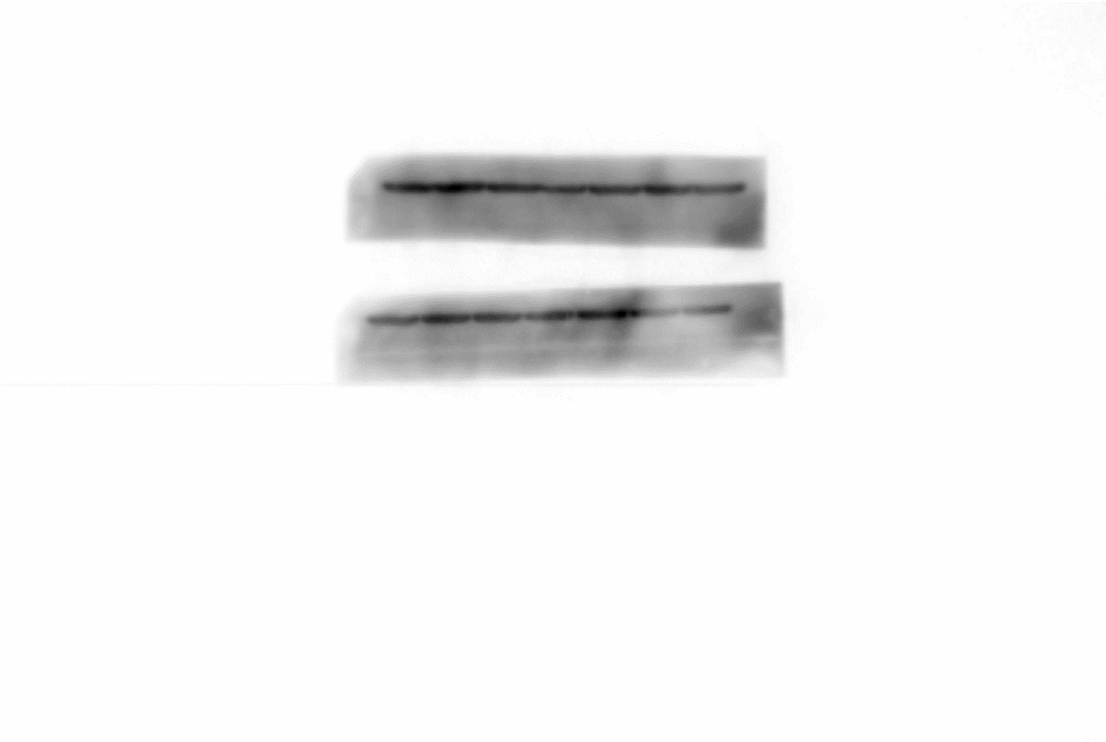

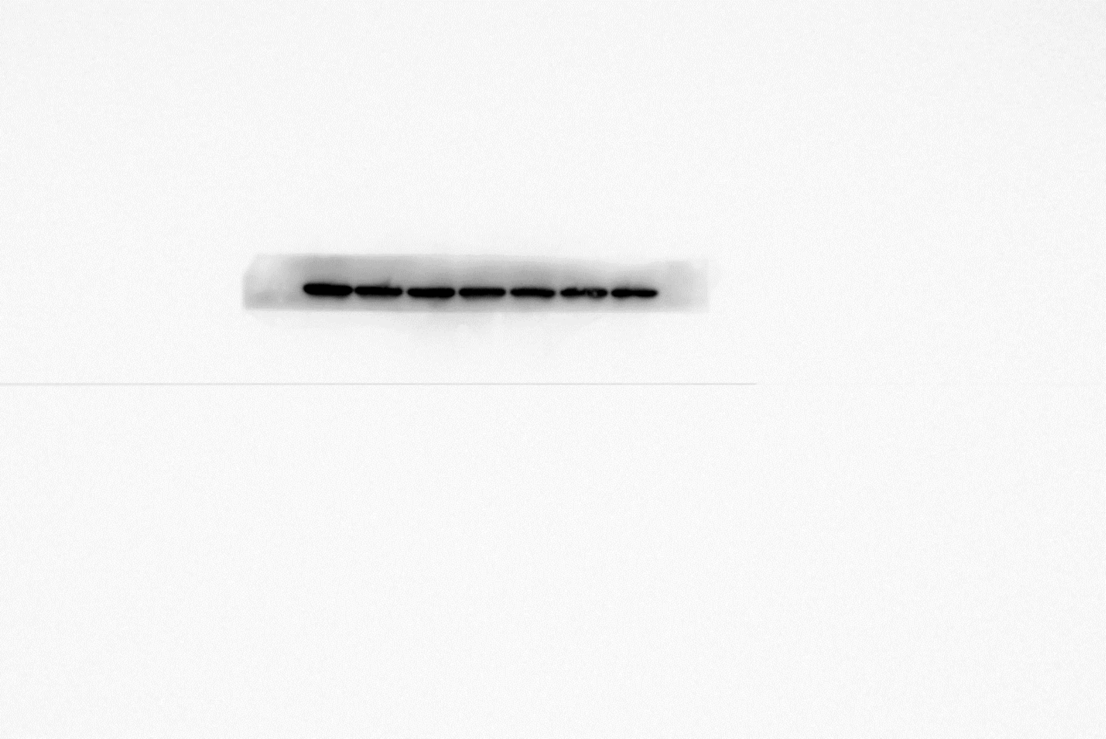

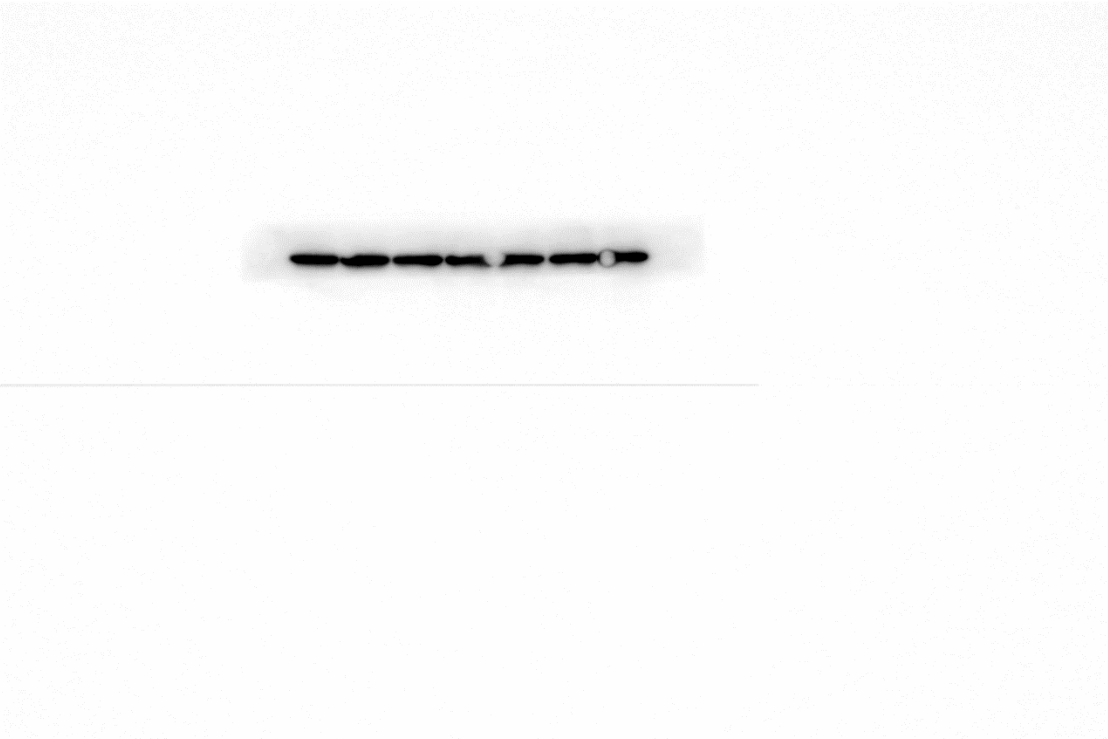

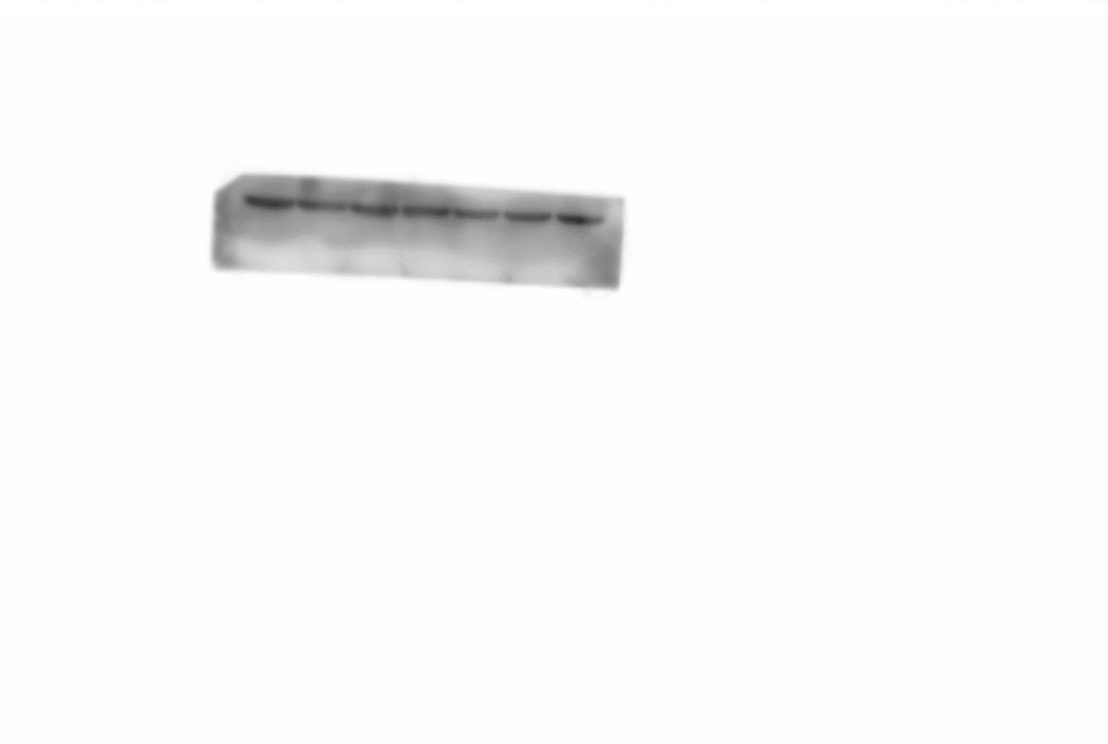

F4A LEFT

HA

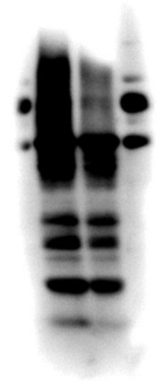

GAPDH

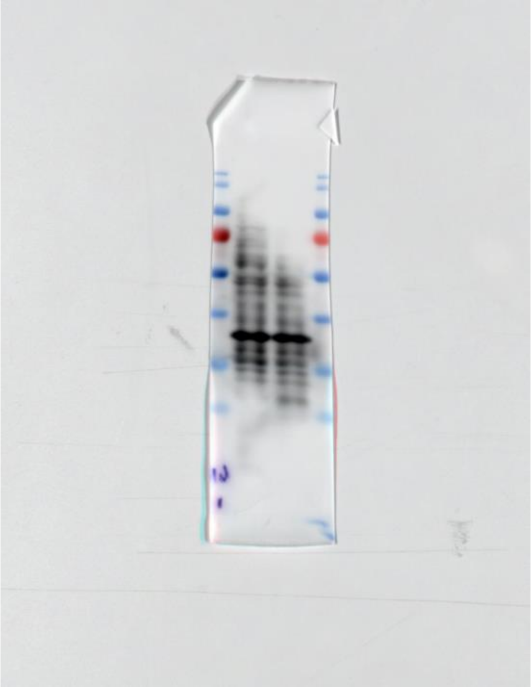

F4 A RIGHT   F4 B RIGHT

HA

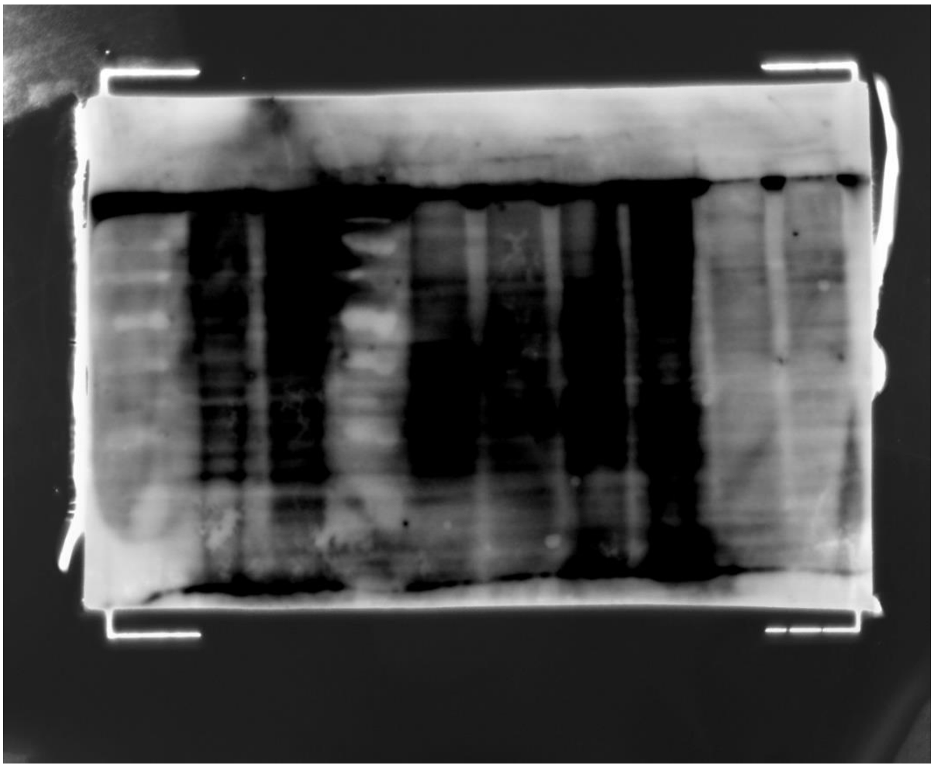

BAX IP

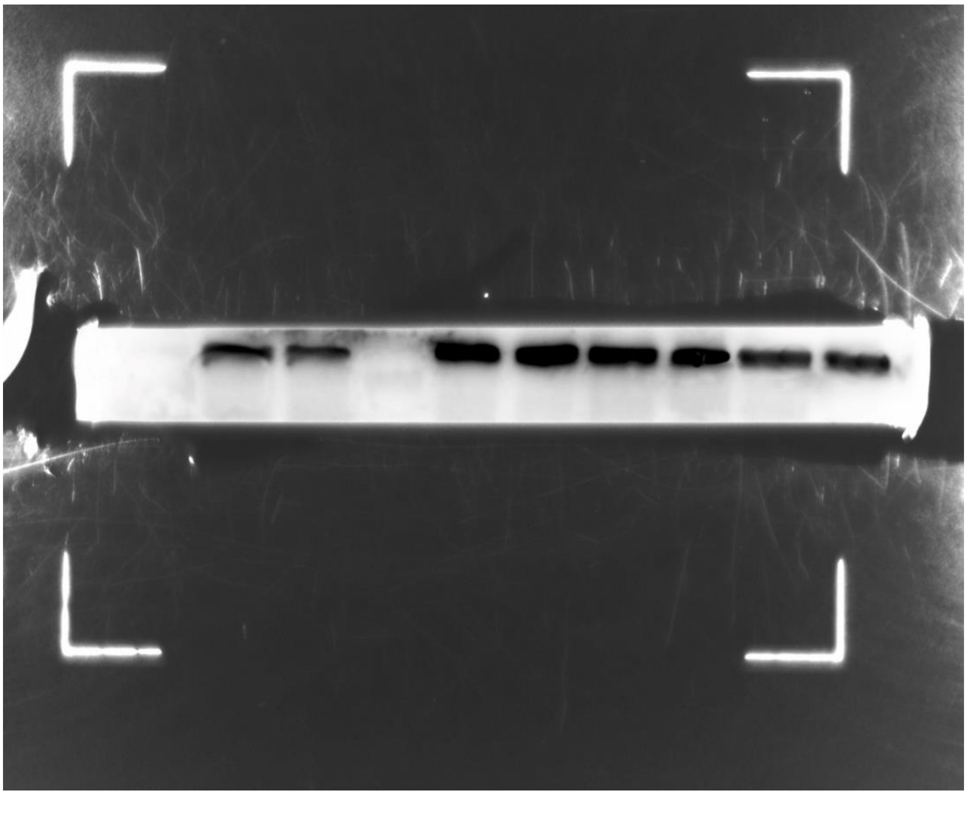

BAX

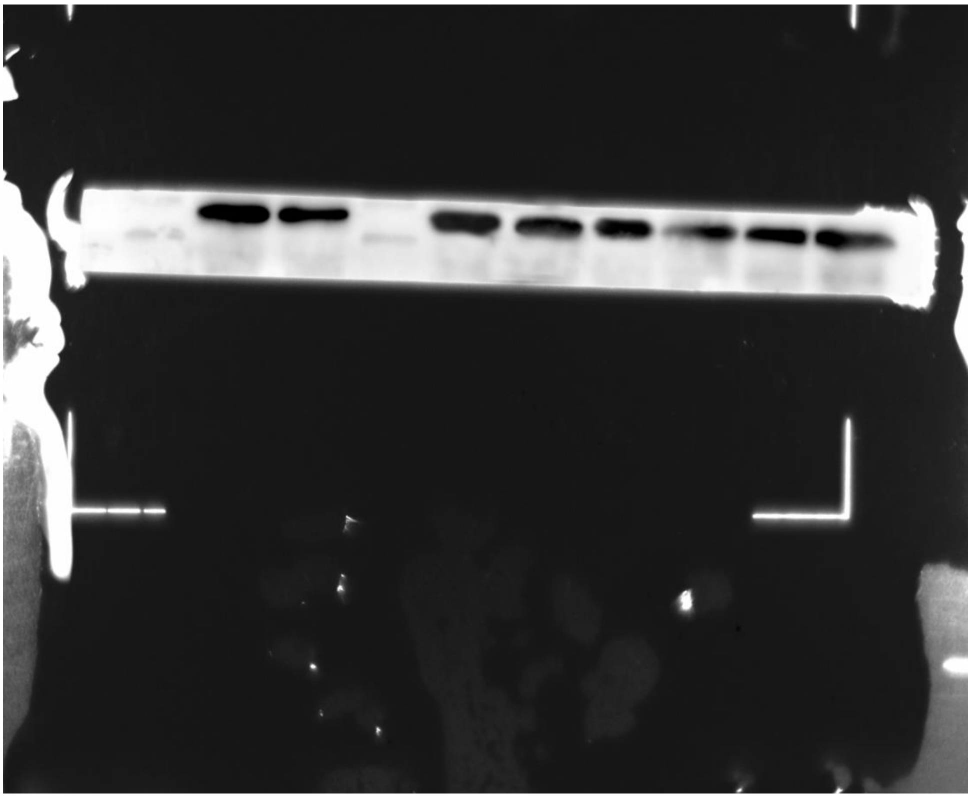

CRABP2

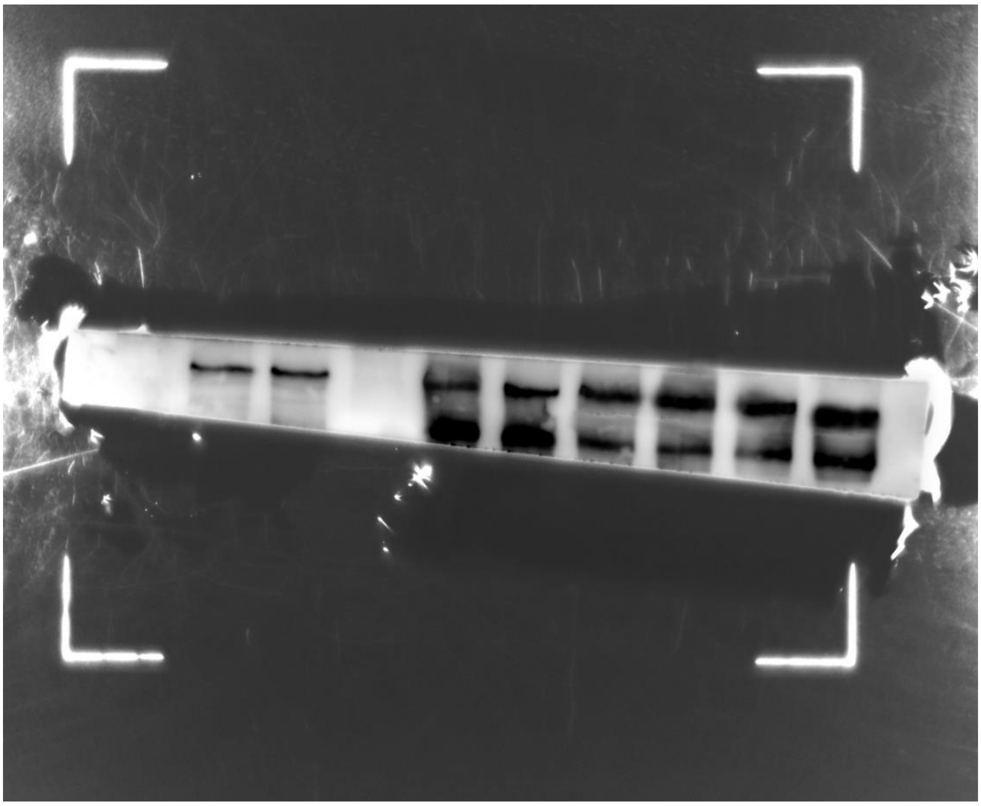

GAPDH

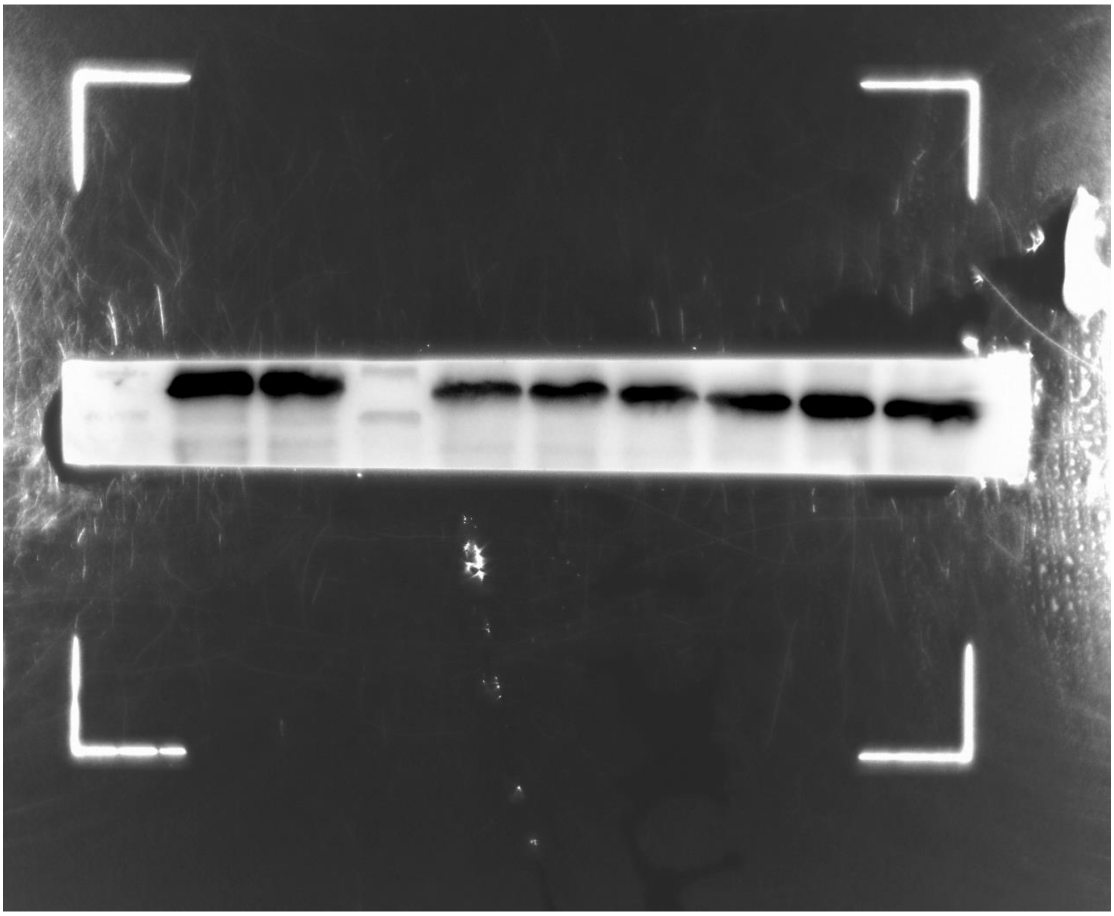

F4 B left

HA

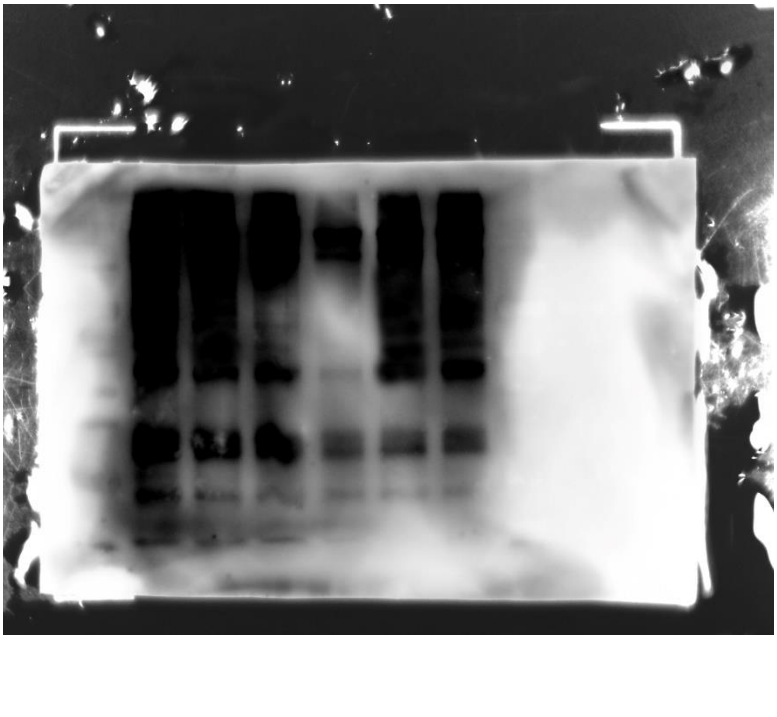

BAX IP

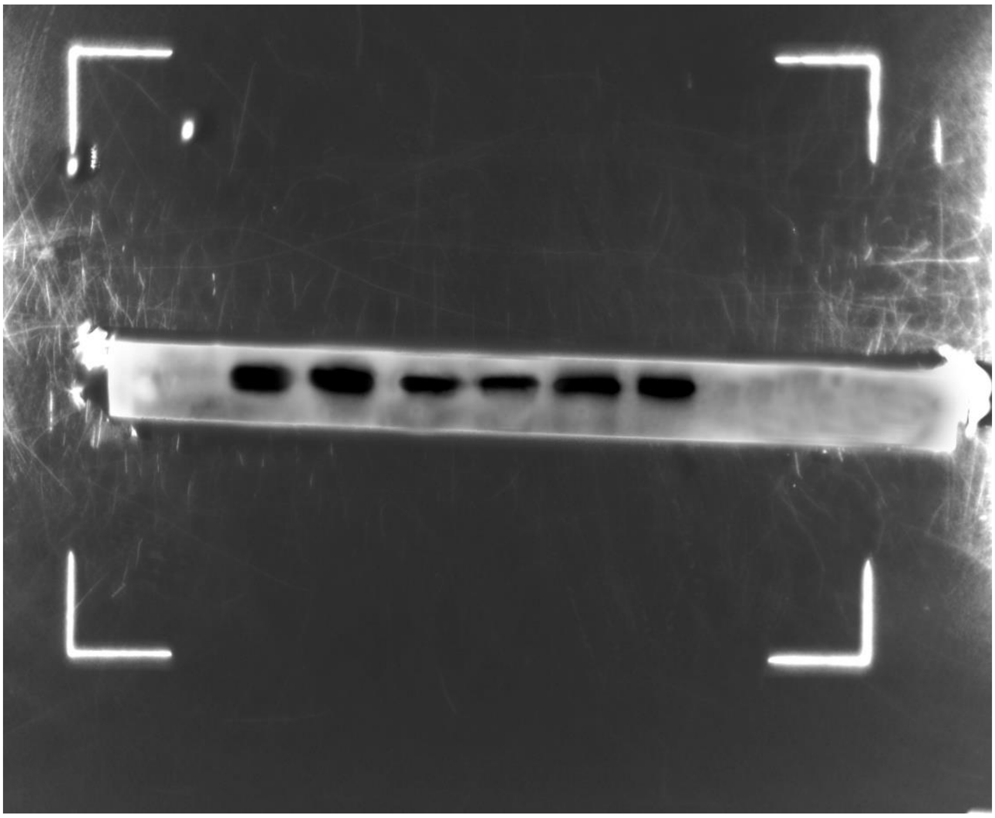

BAX

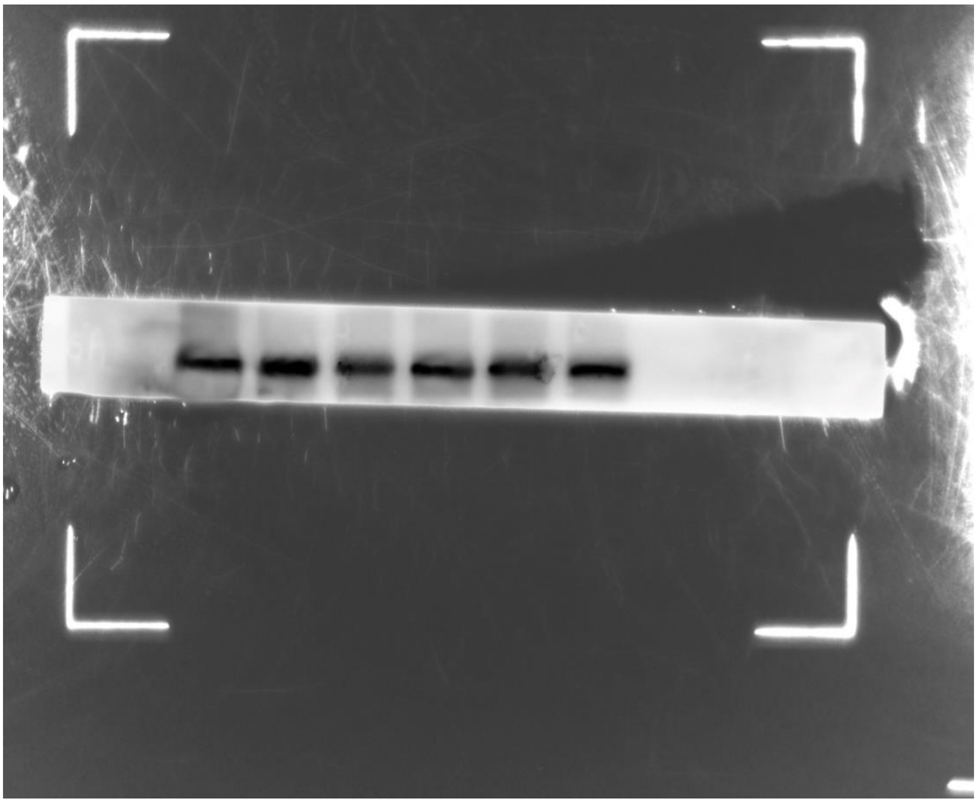

CRABP2

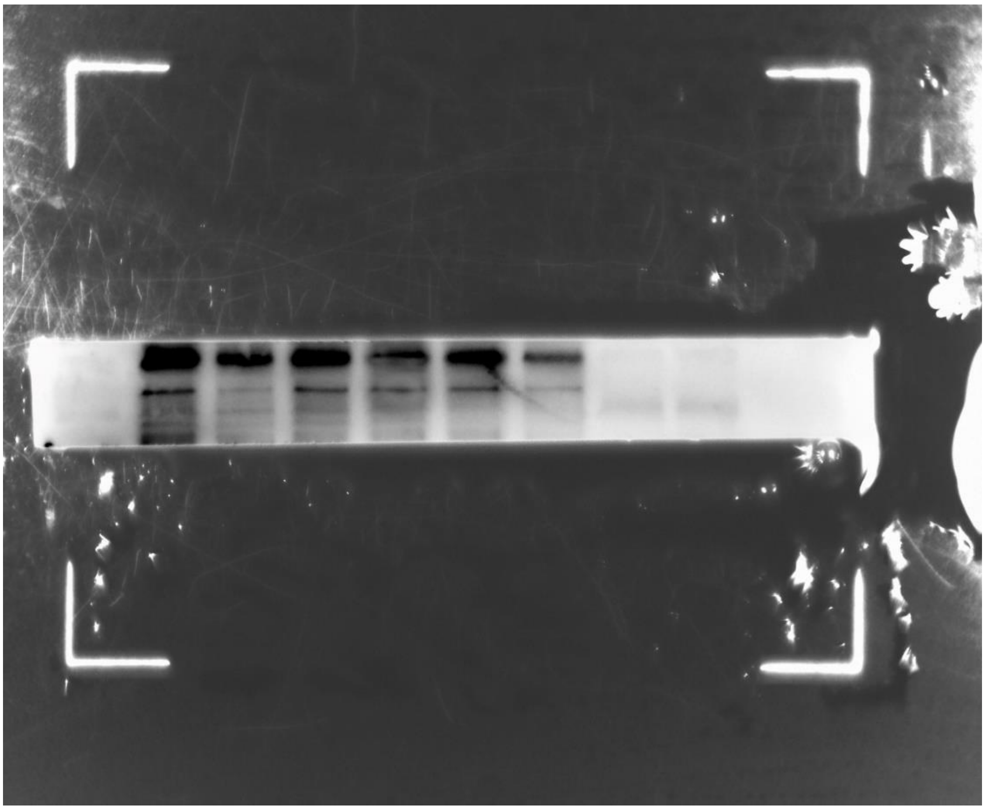

GADPH

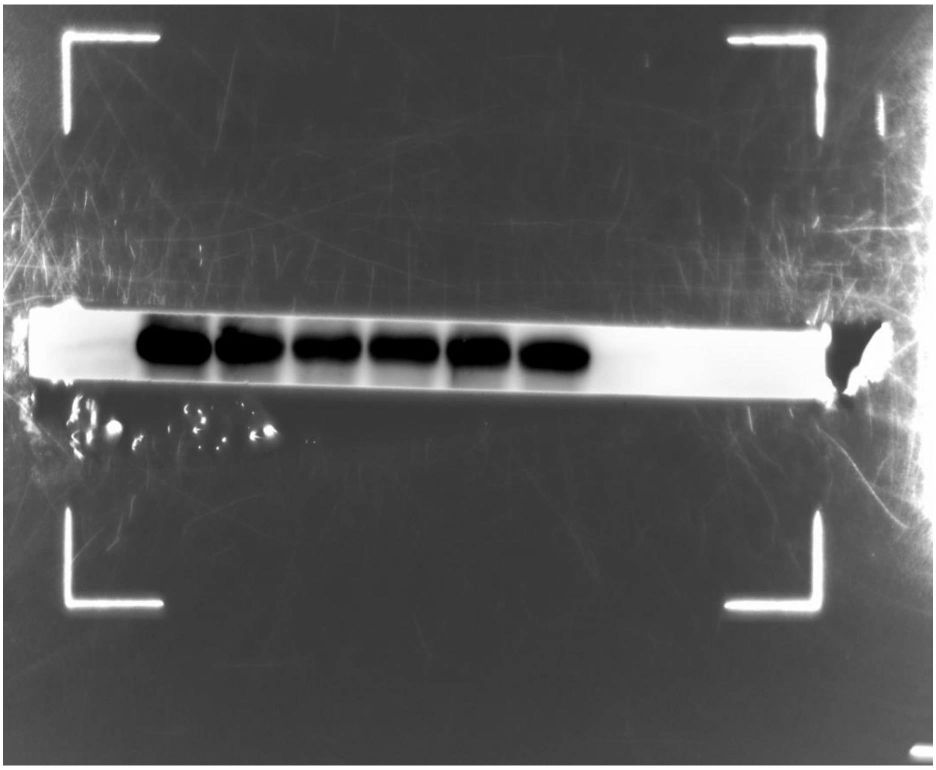

F4 E

GADPH

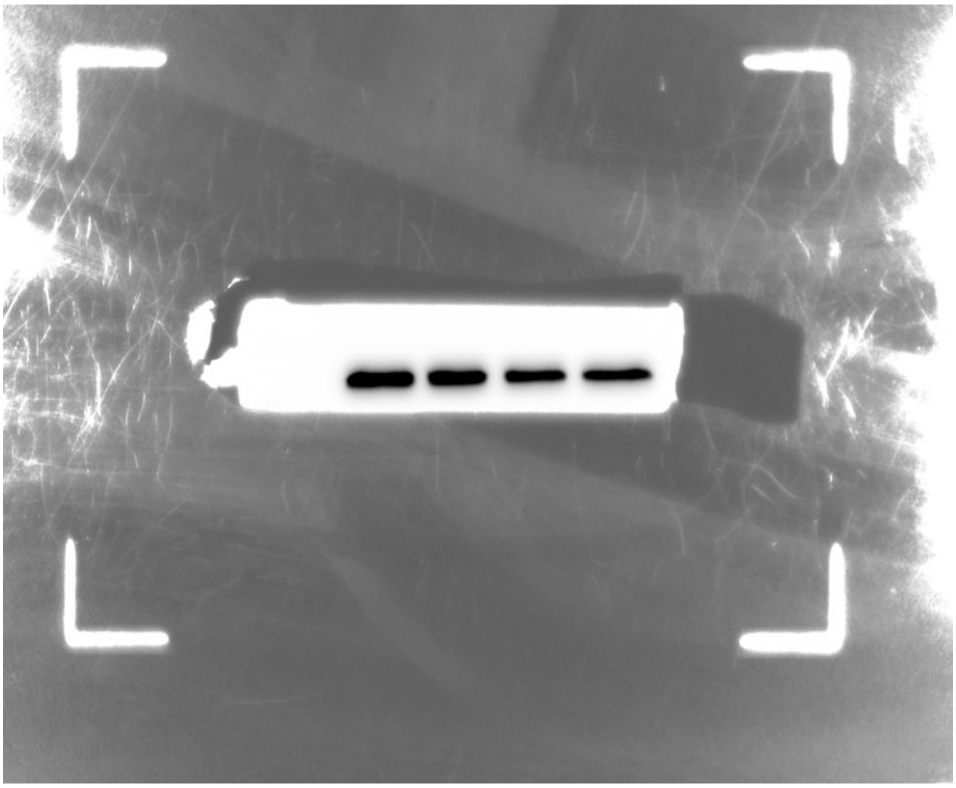

CASPASE

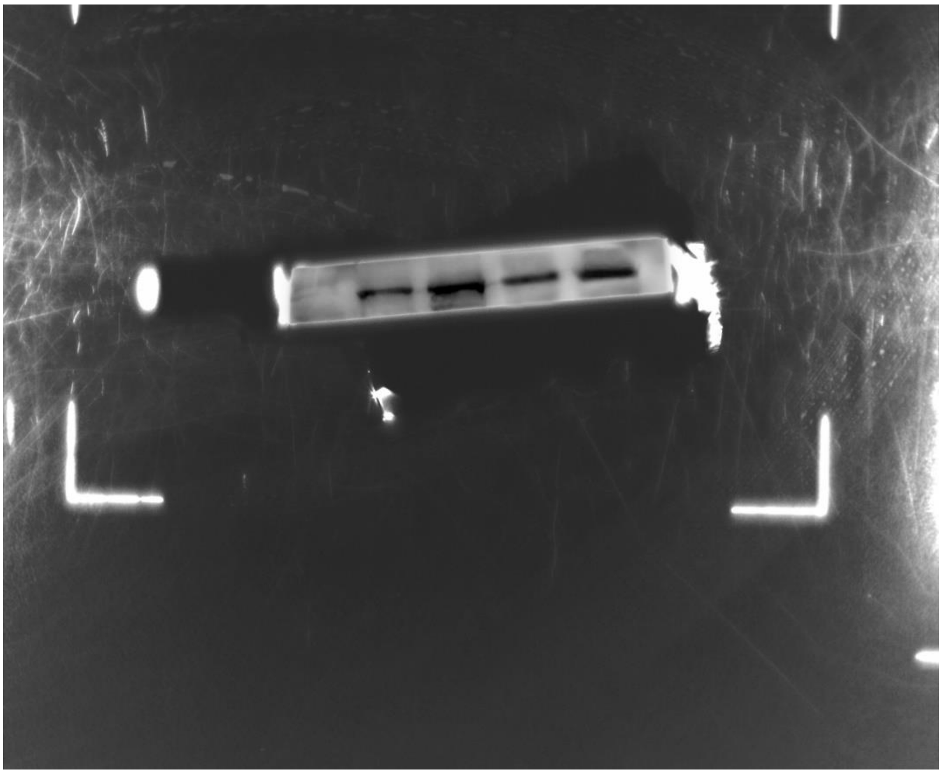

F4 F

ATR

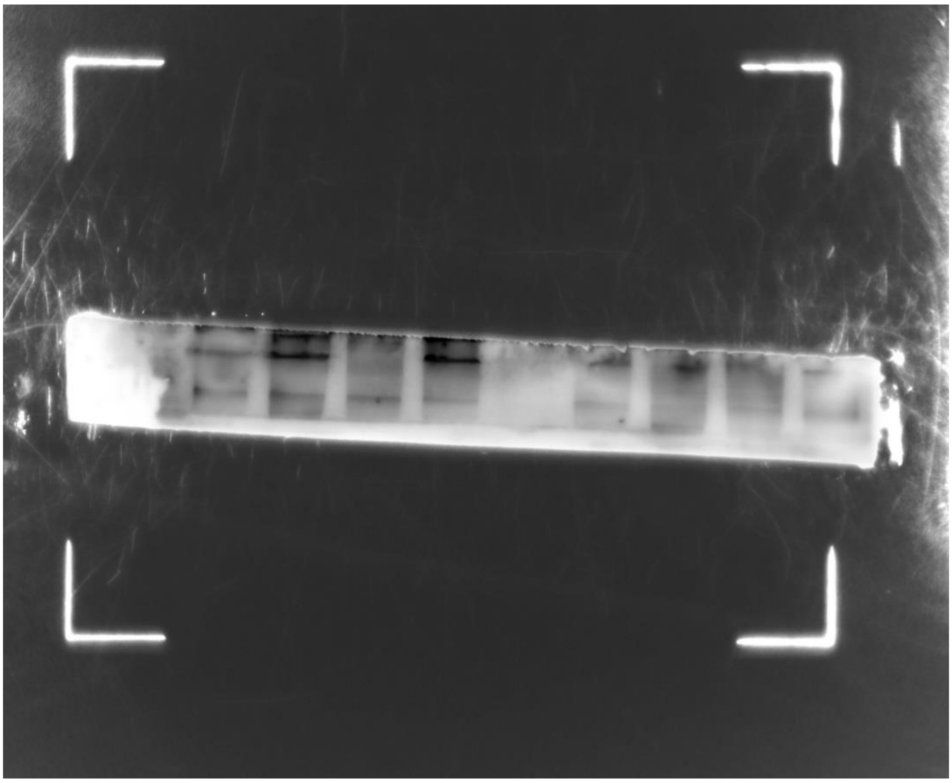

P-ATR

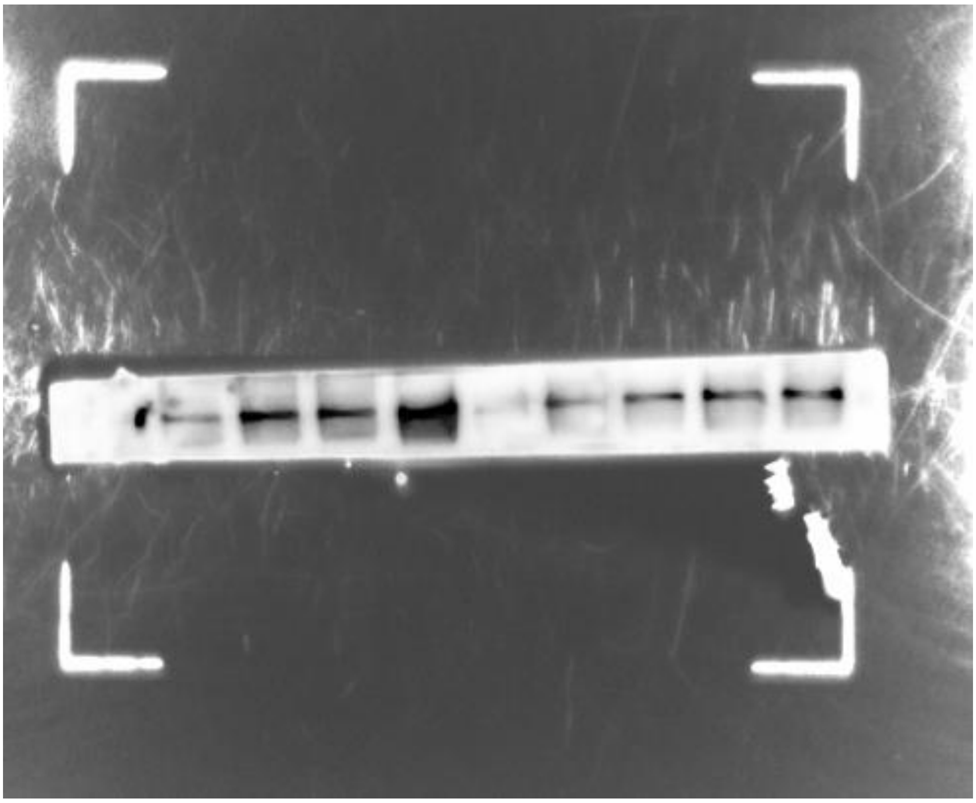

ATM

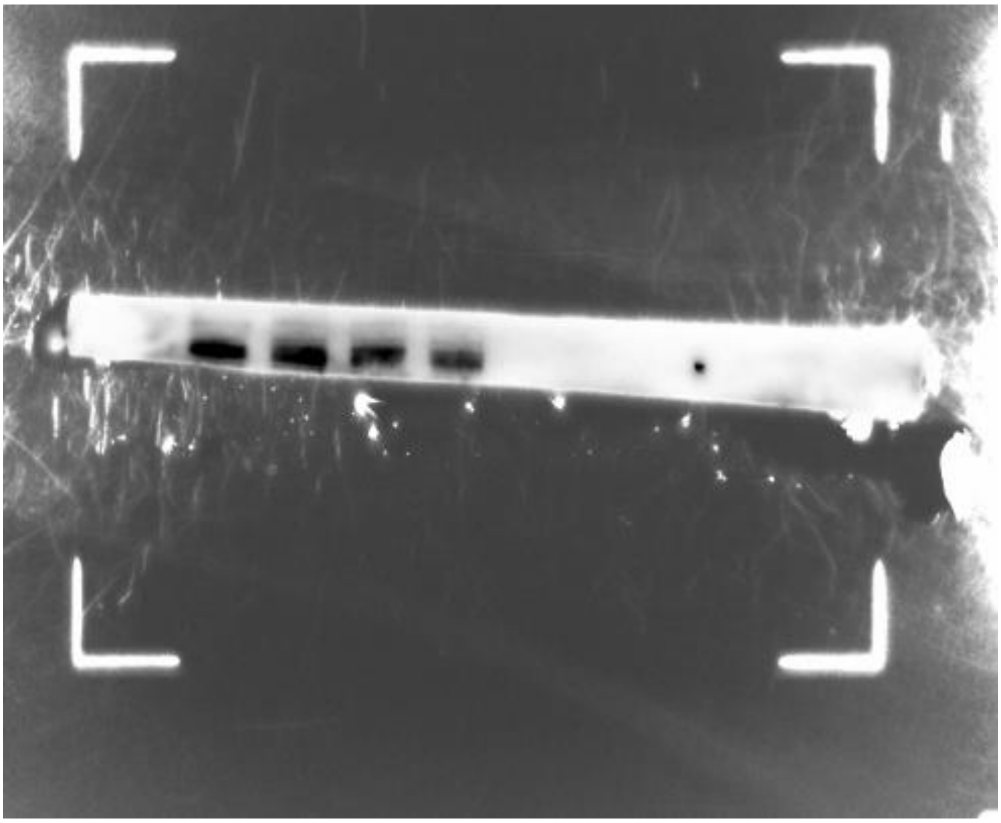

P-ATM

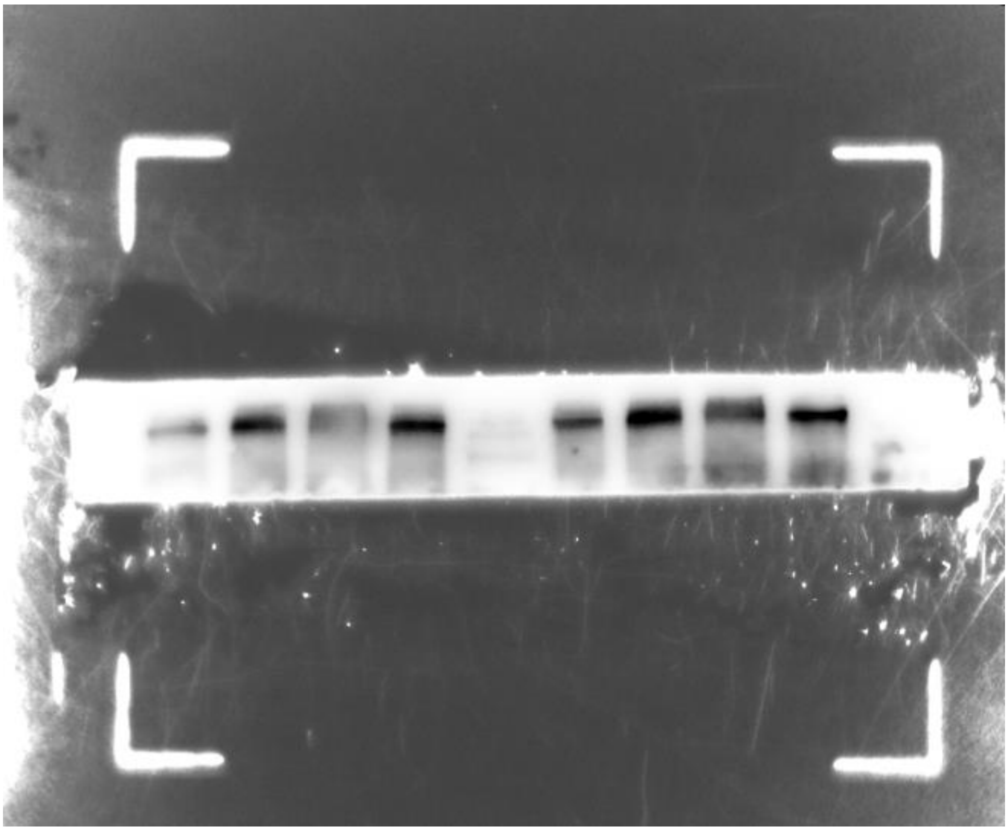

P53

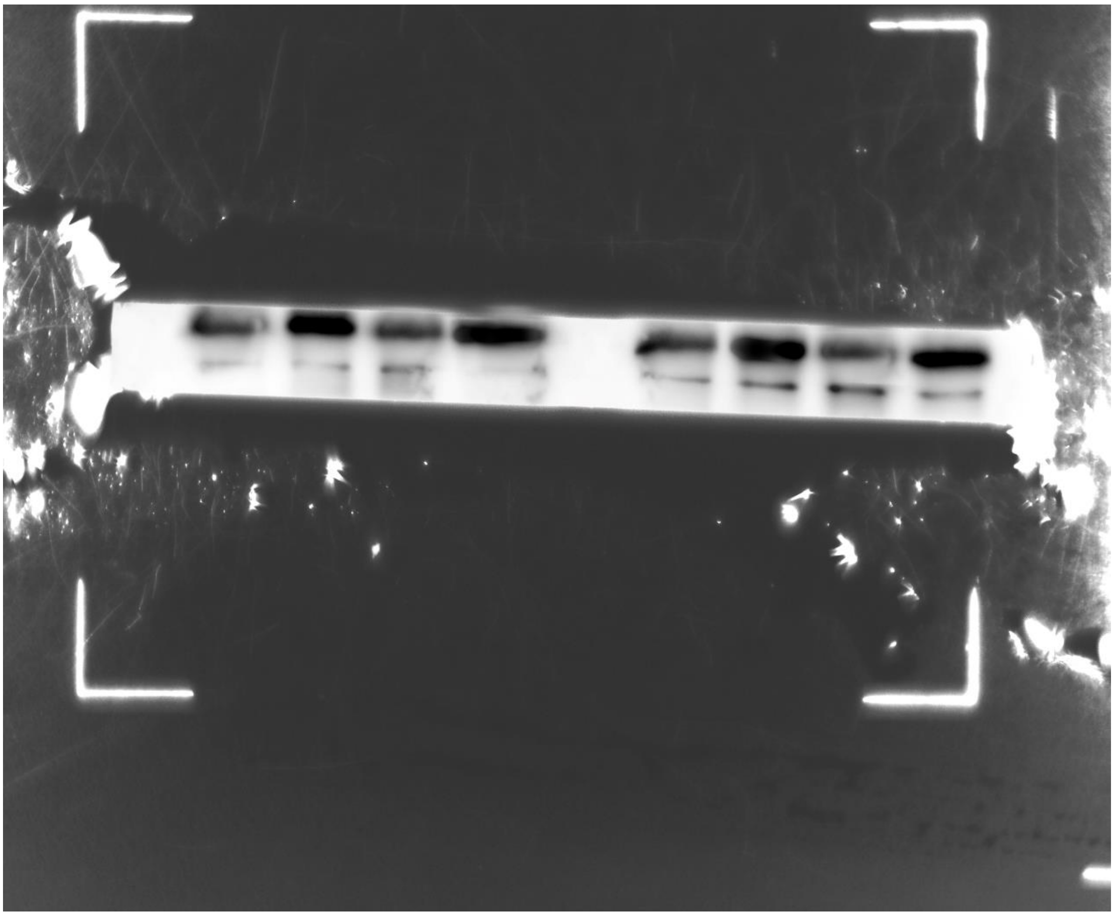

P-P53

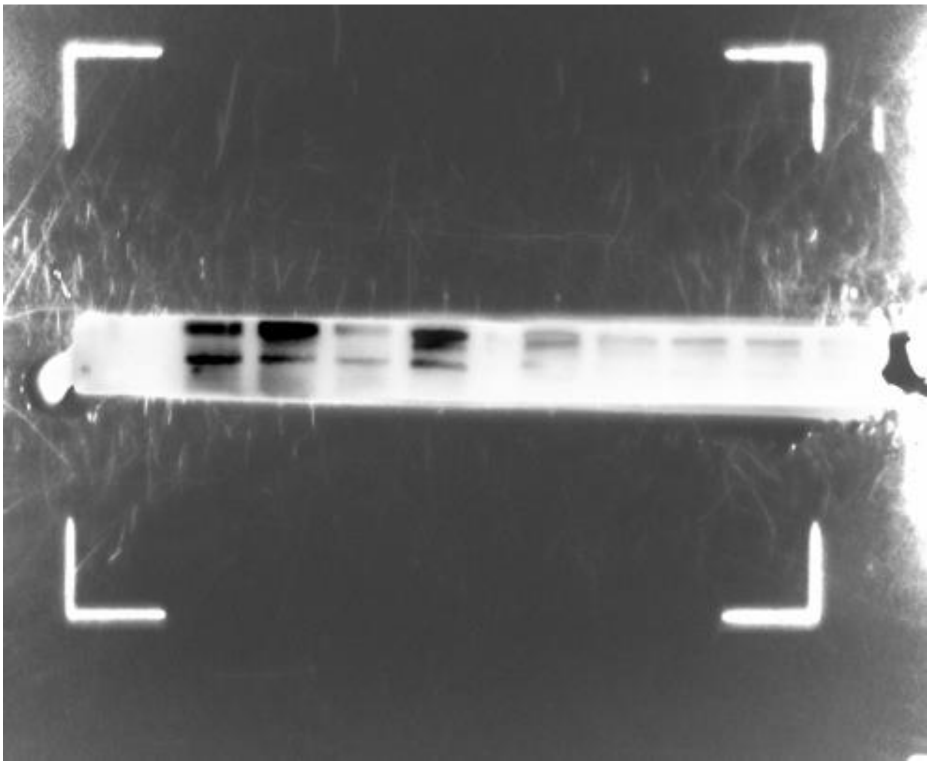

BAX

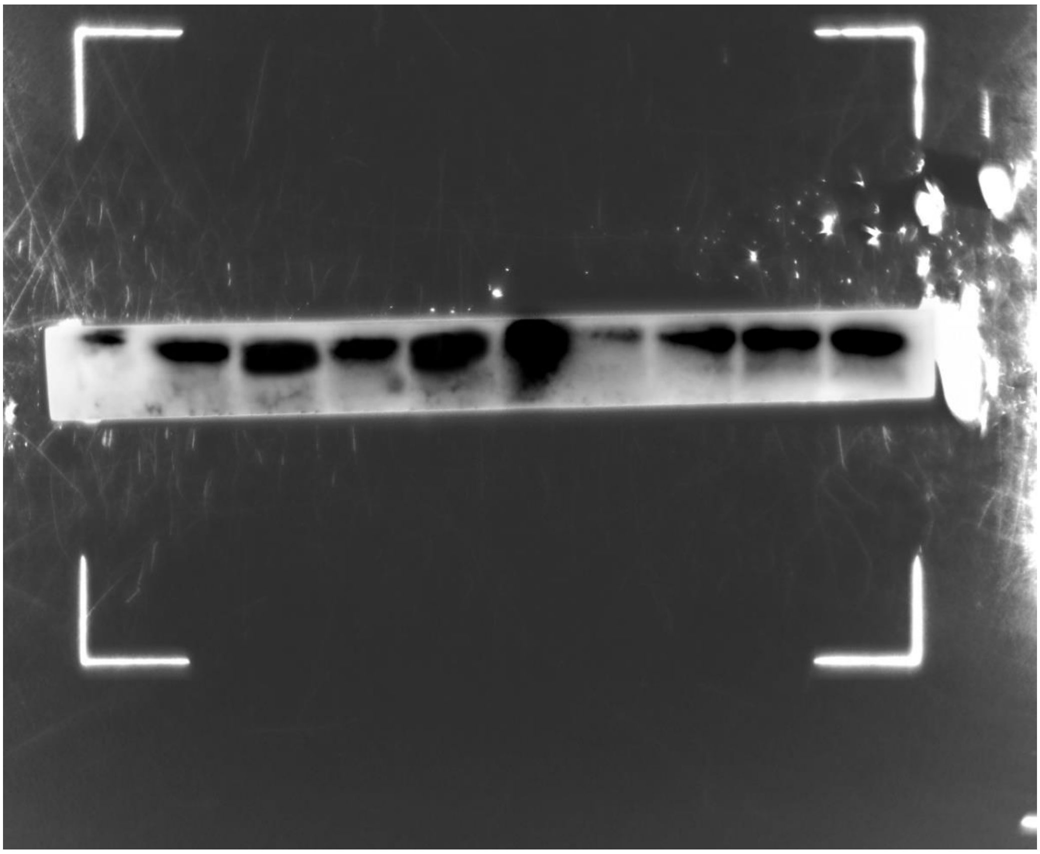

GAPDH

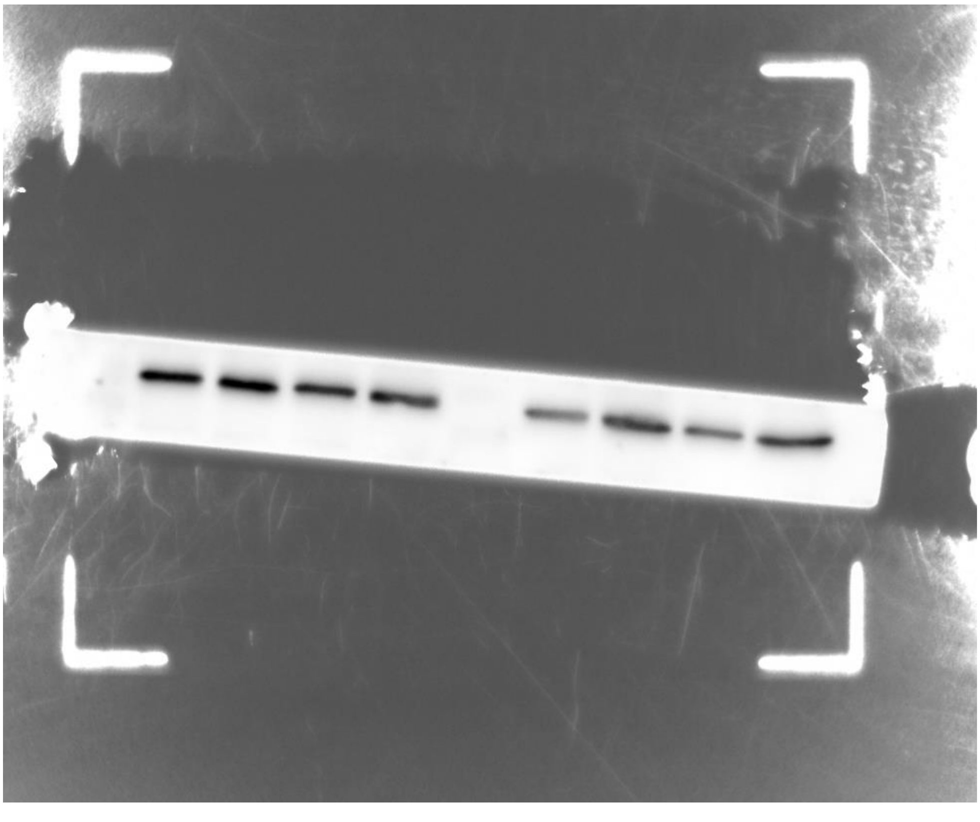

F4 G

BAX

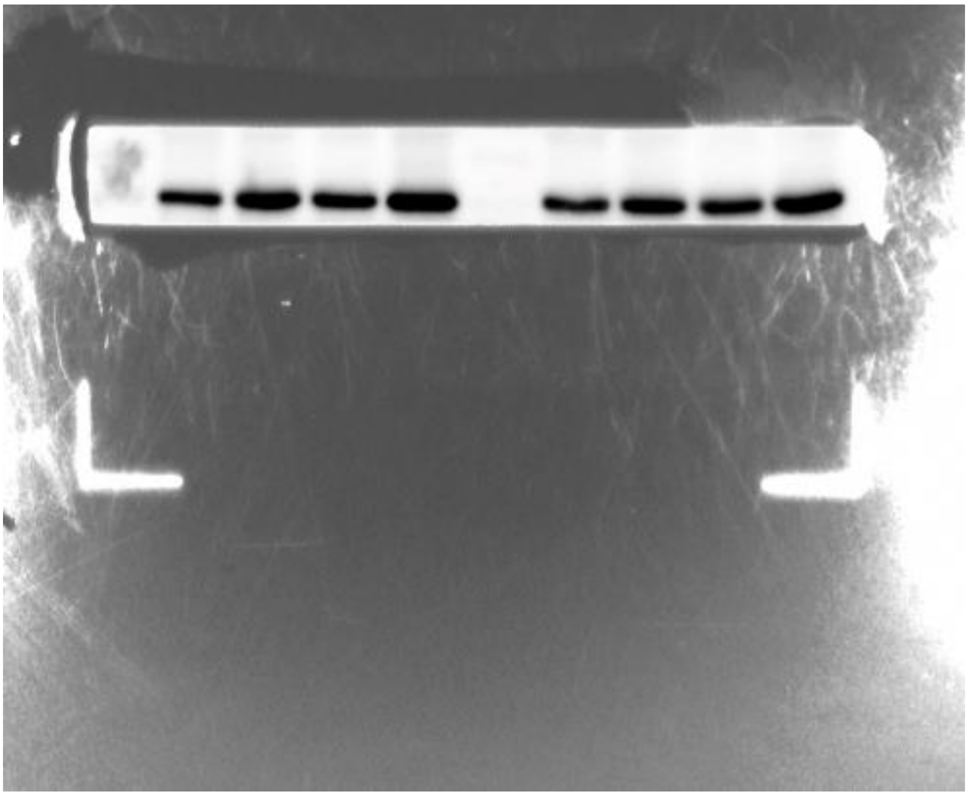

TOMM40

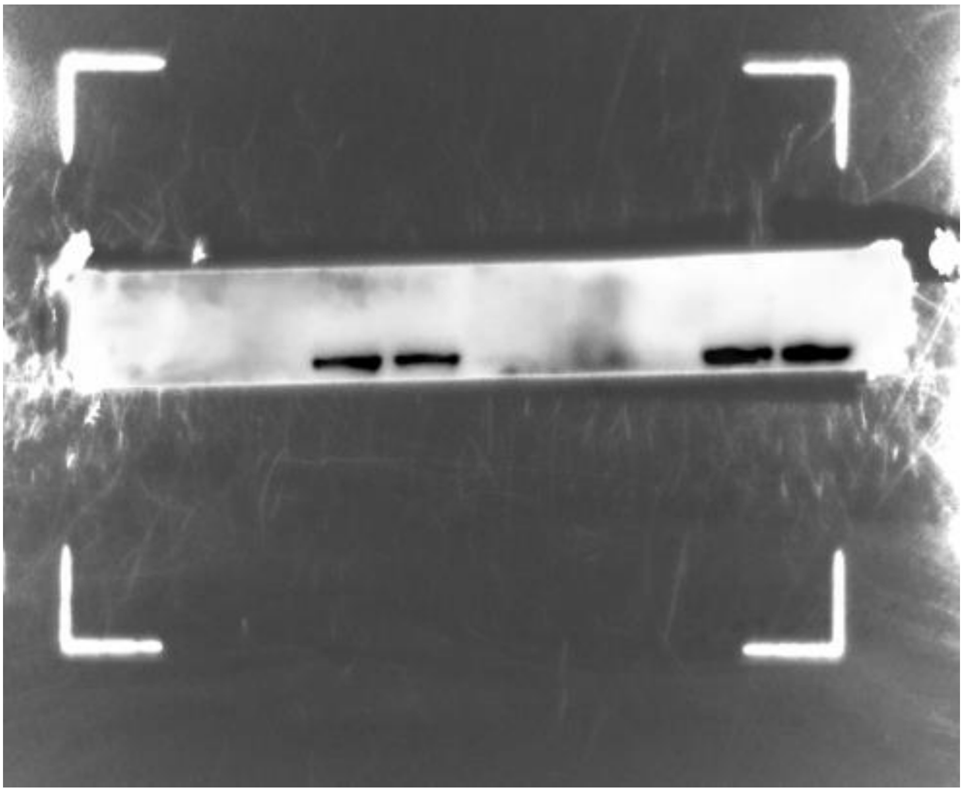

TUBULIN

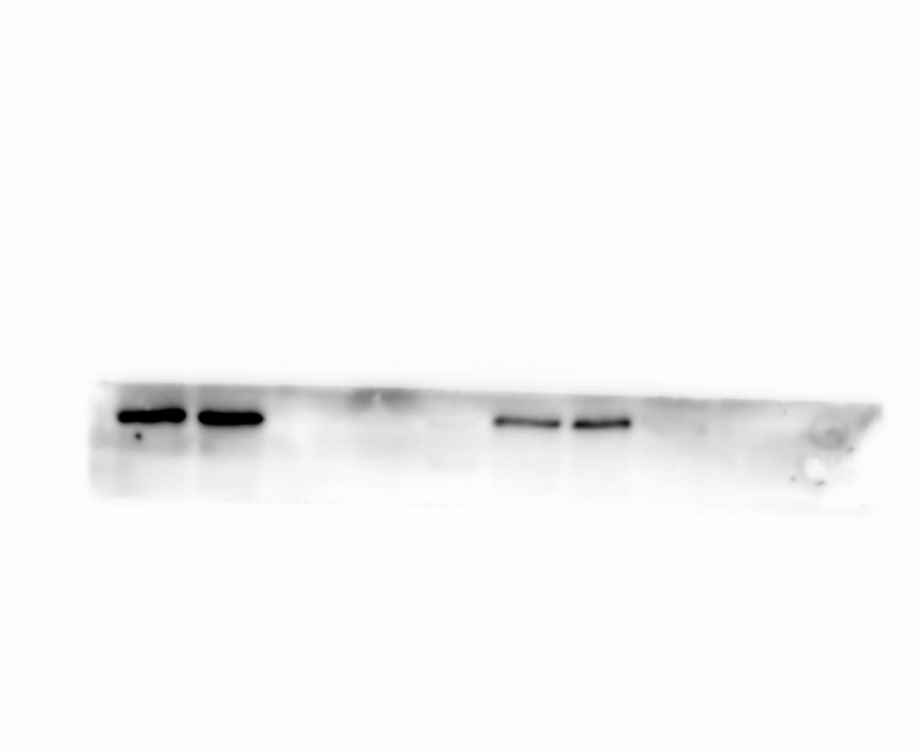

F4 I LEFT

GAPDH

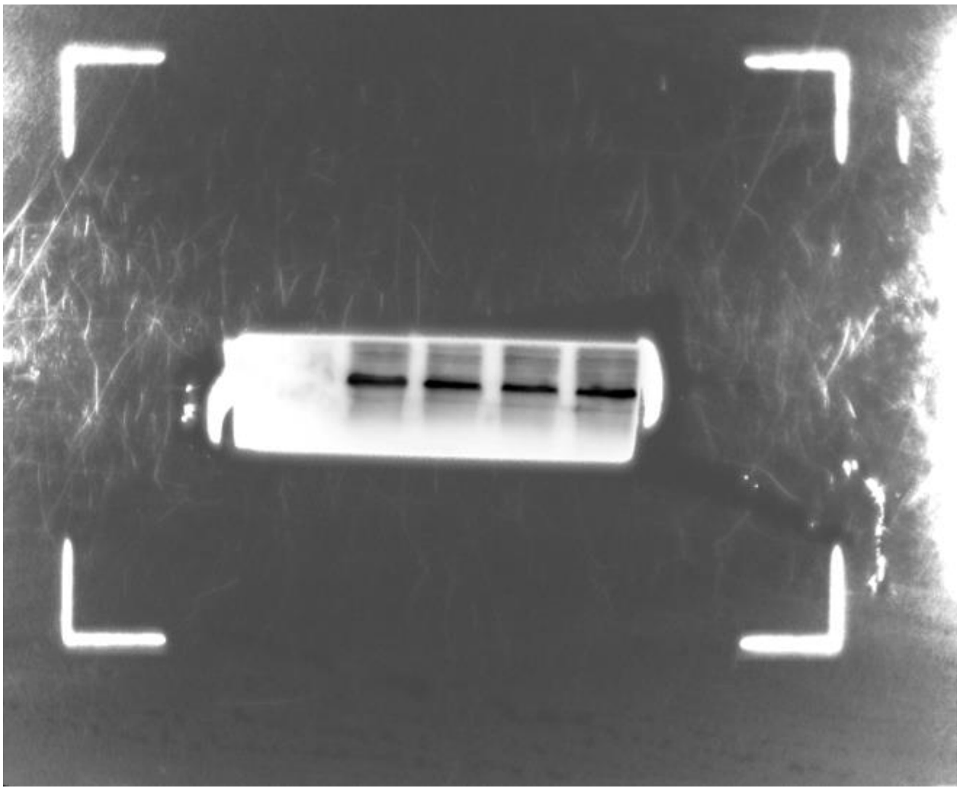

CRABP2

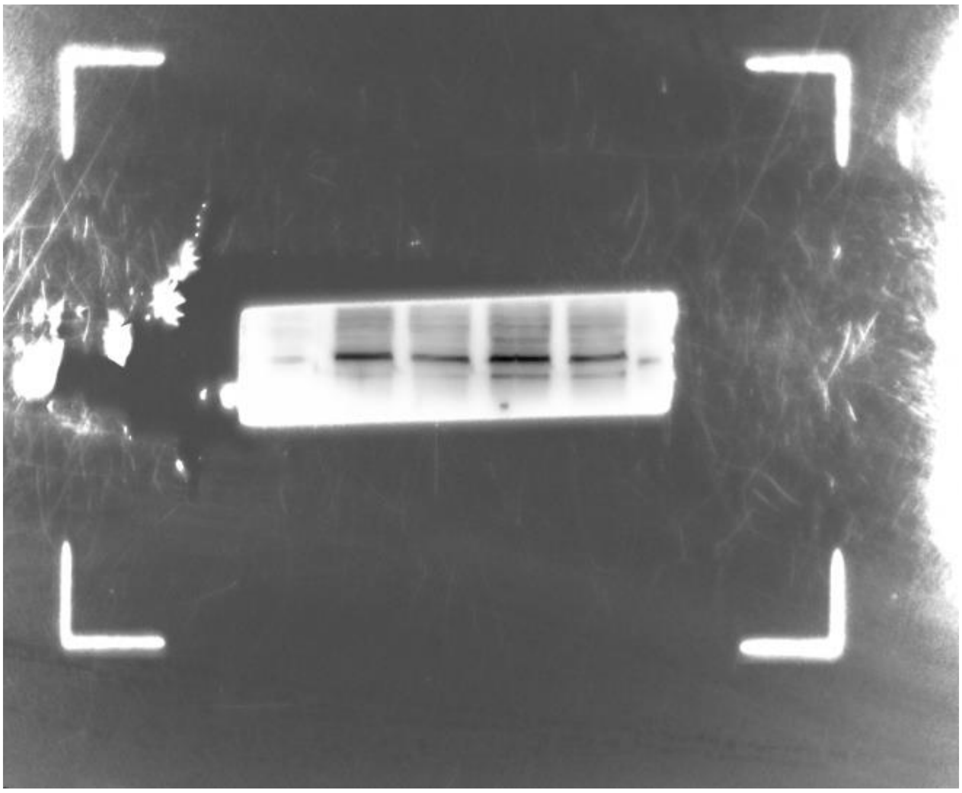

CASPASE

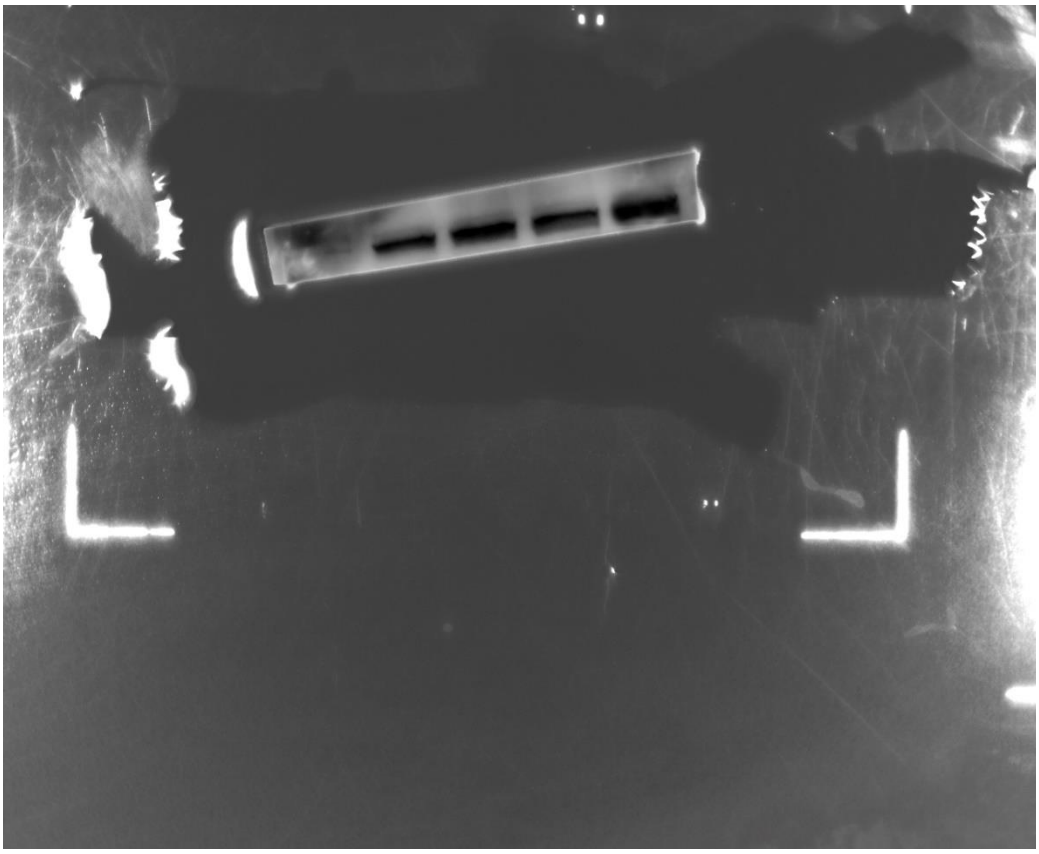

RIGHT

GAPDH

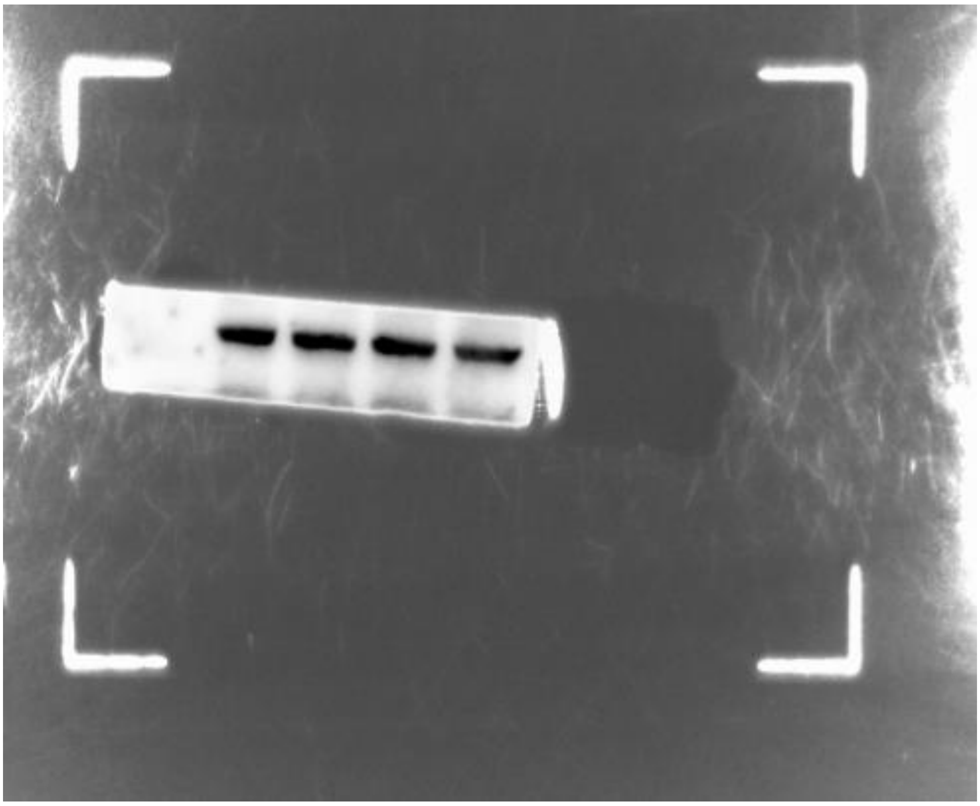

CRABP2

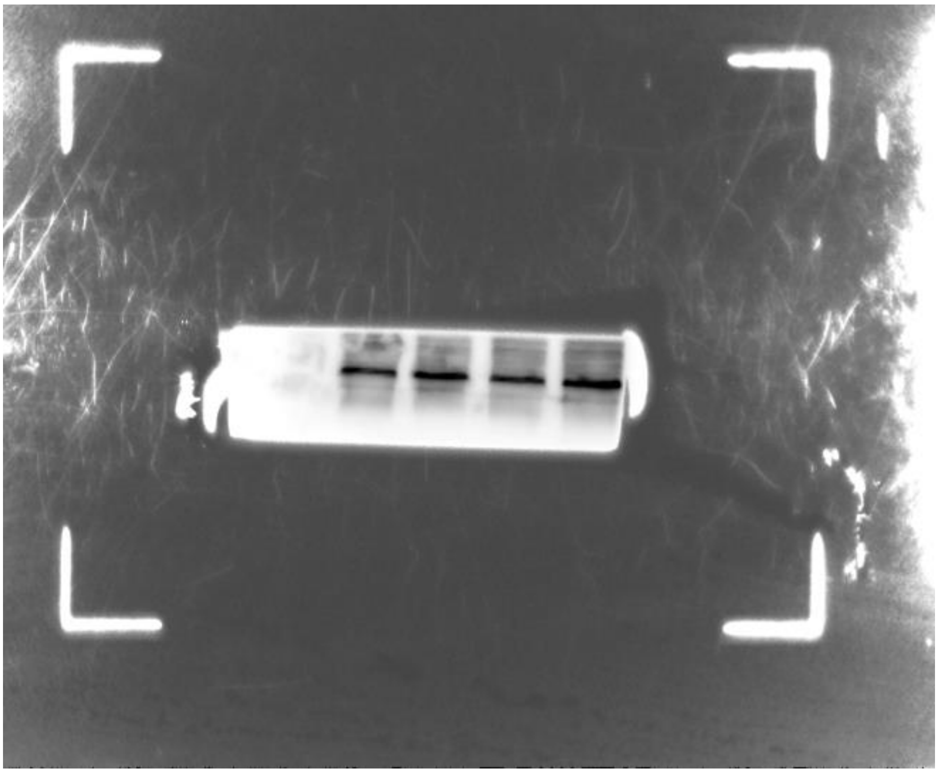

CASPASE

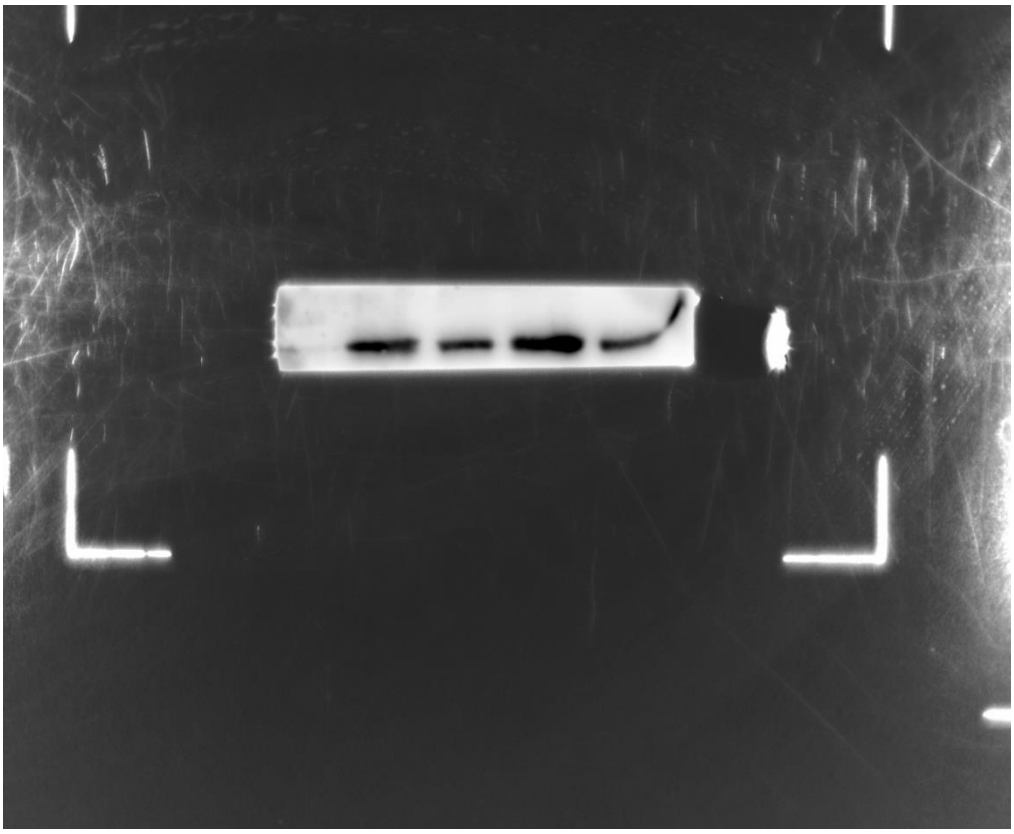

F4 j

ATM

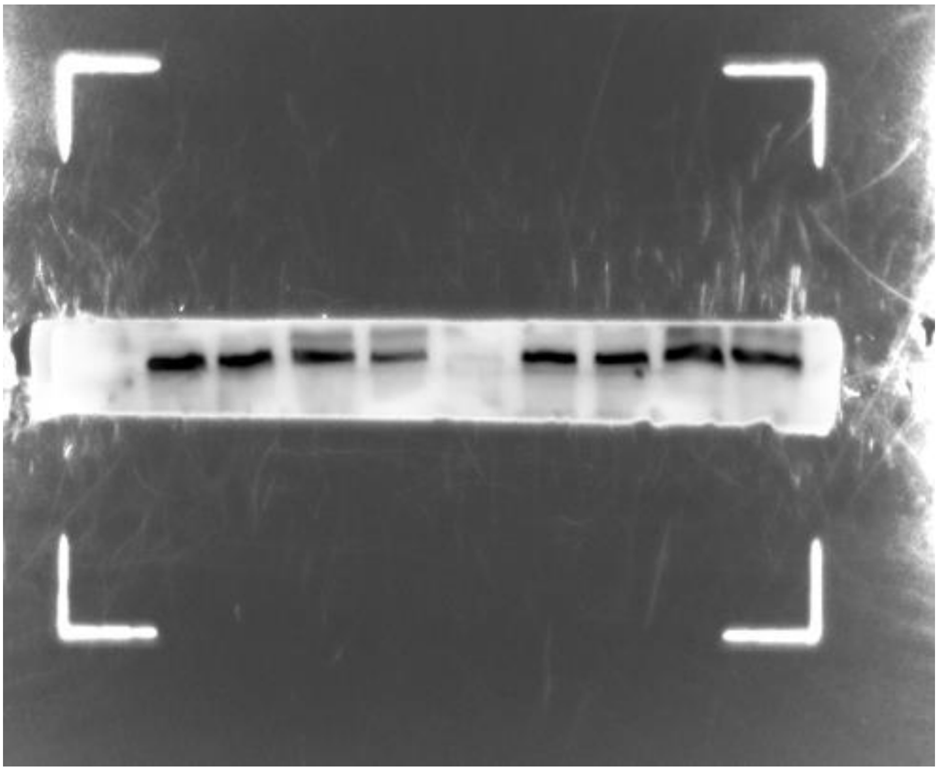

P-ATM

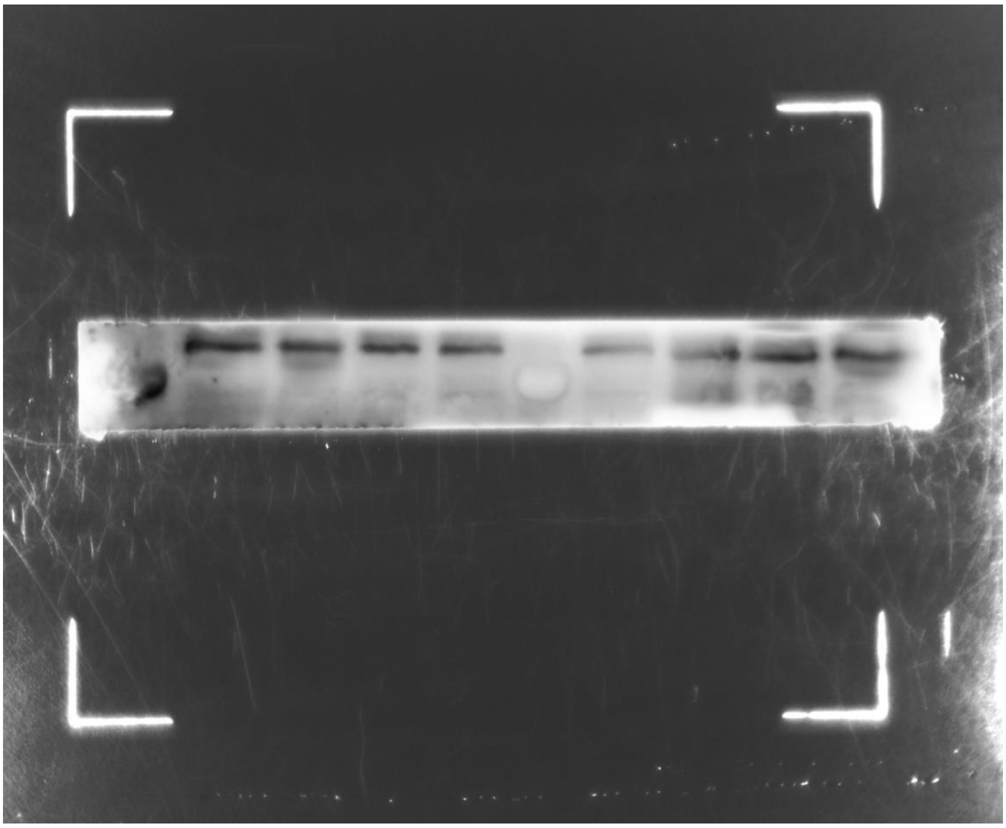

ATR

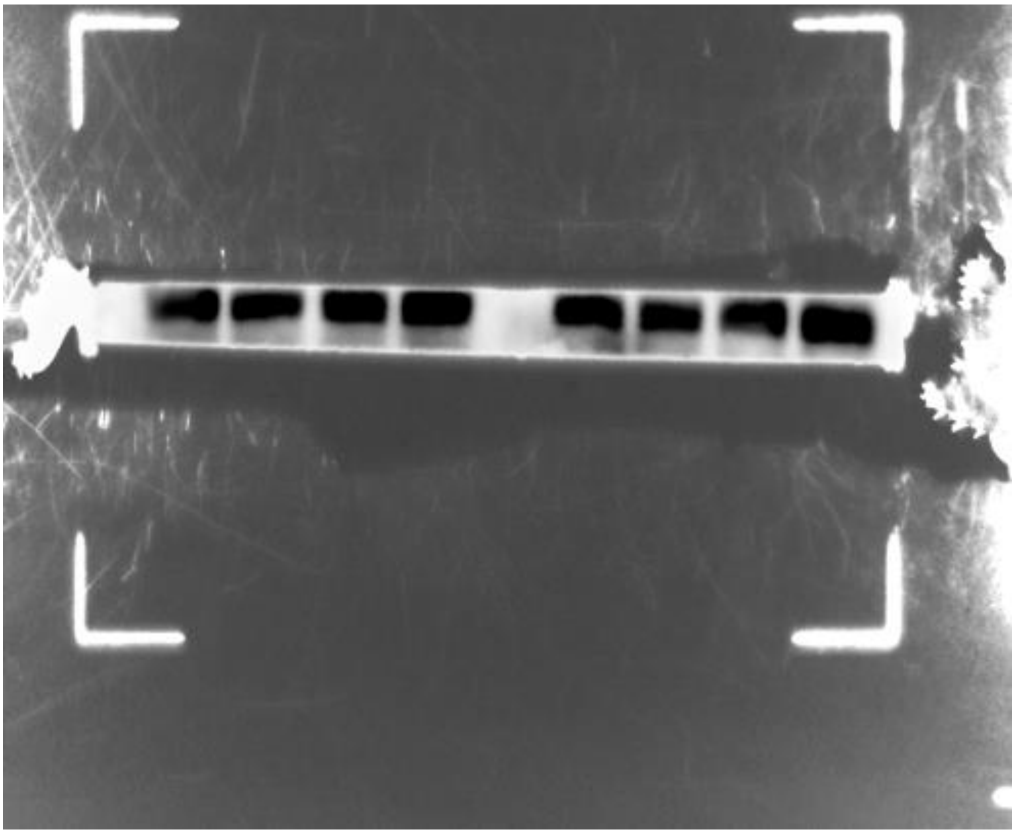

P-ATR

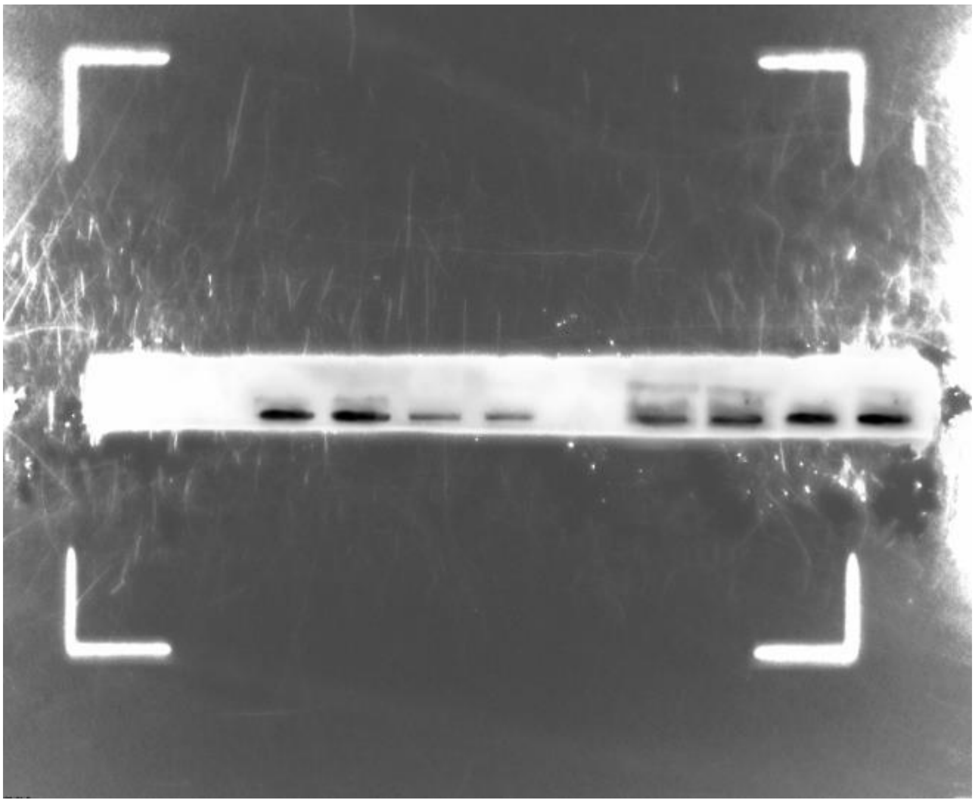

P53

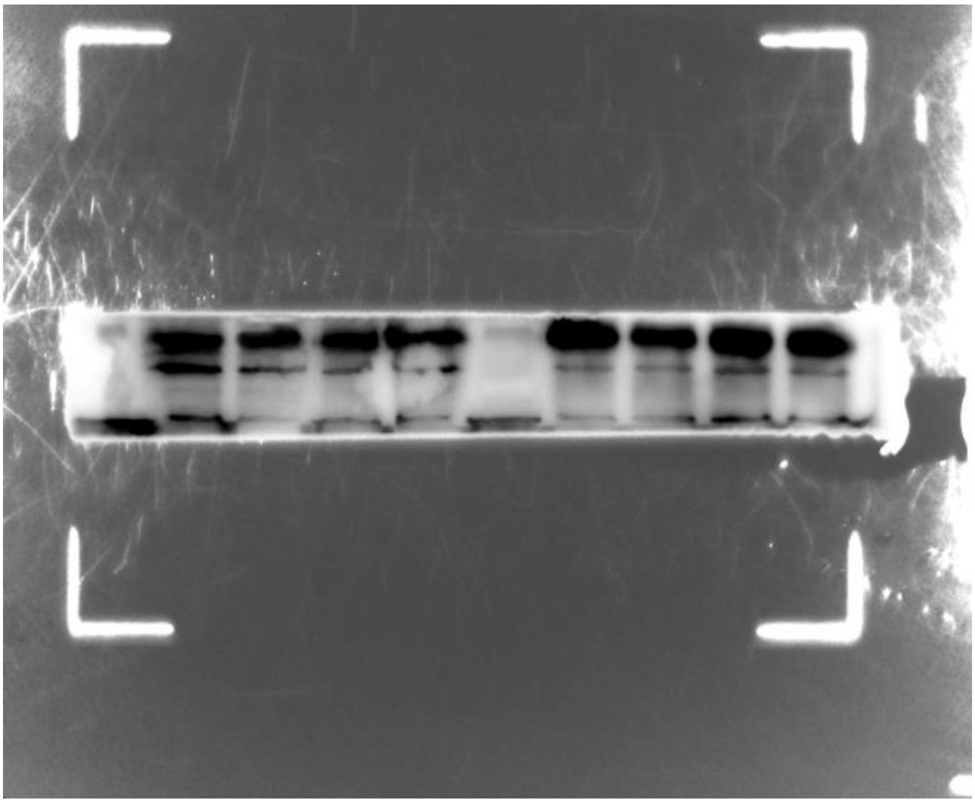

P-P53

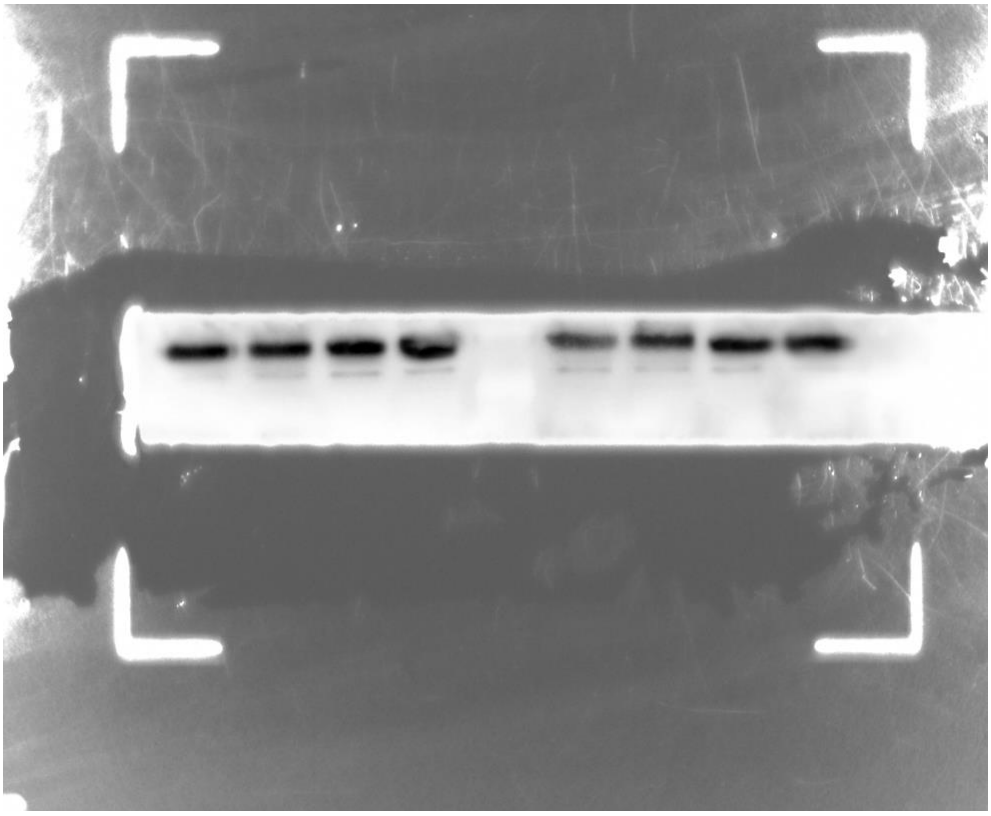

BAX

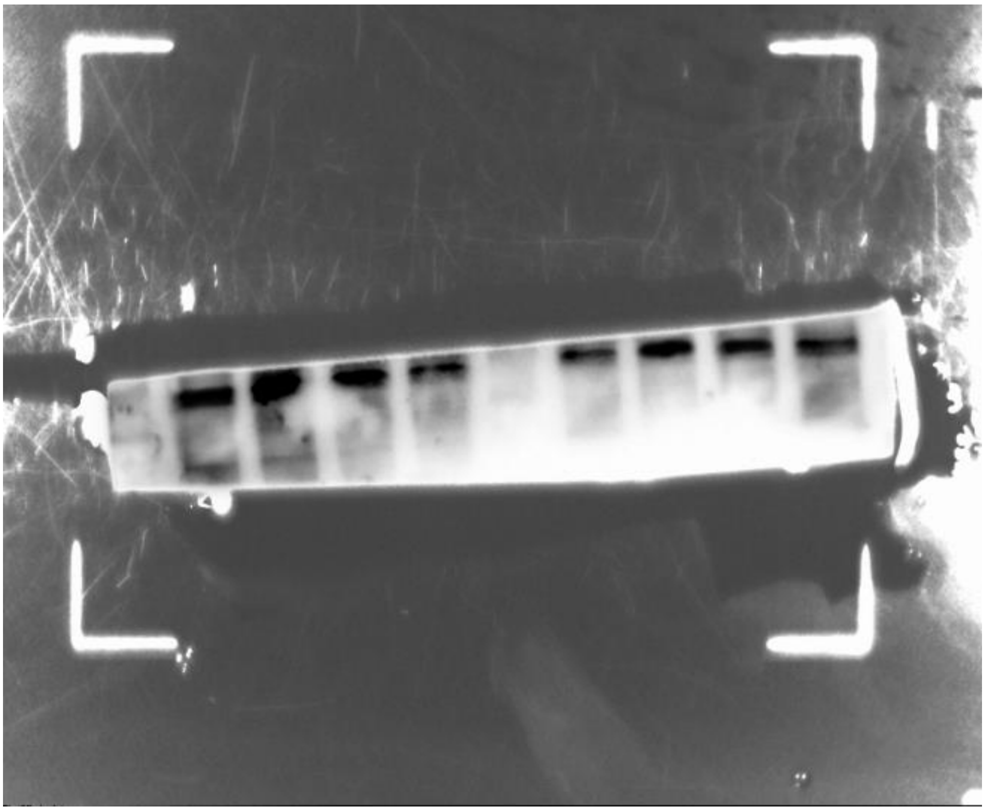

GAPDH

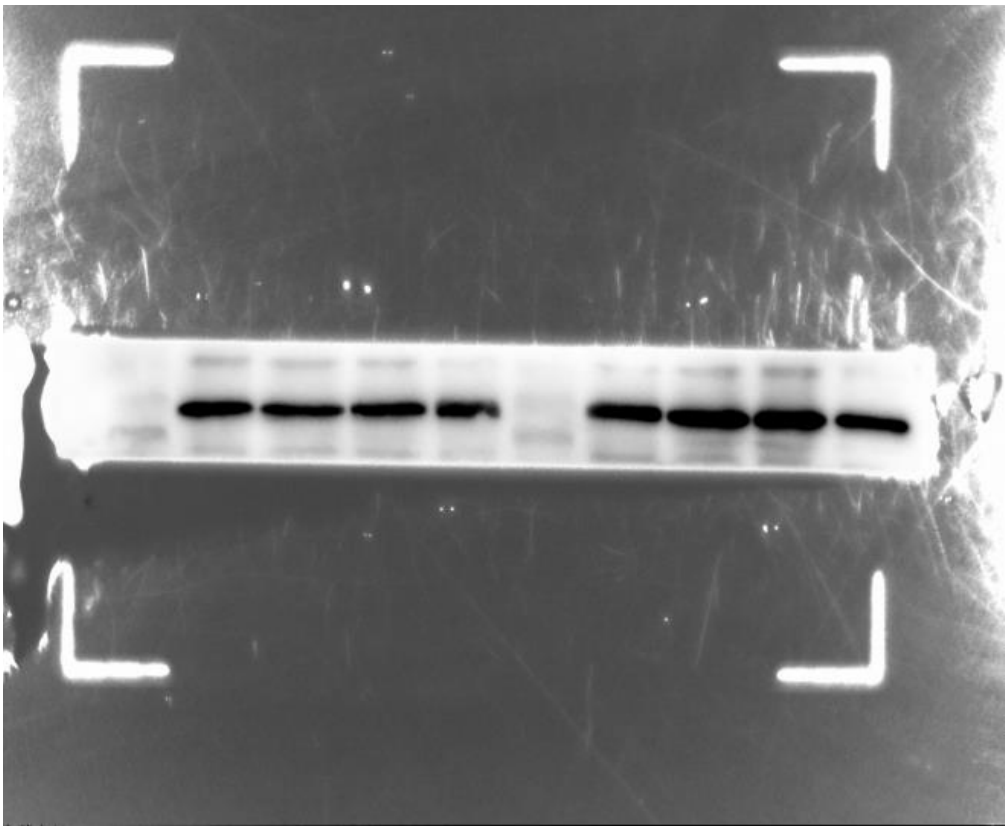

F4 K

BAX

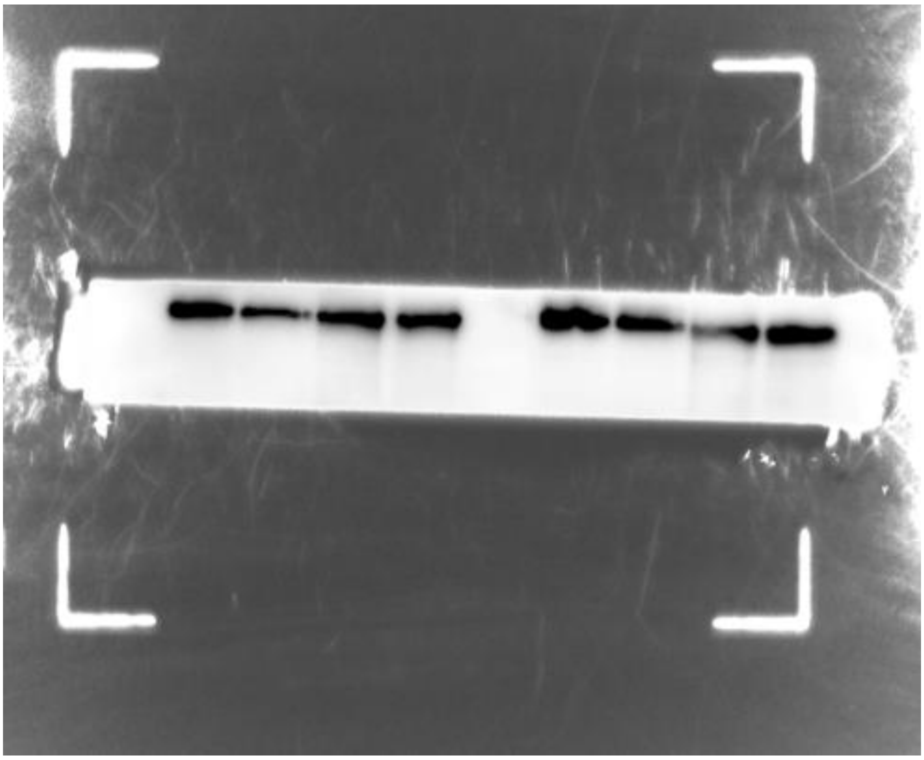

TOMM40

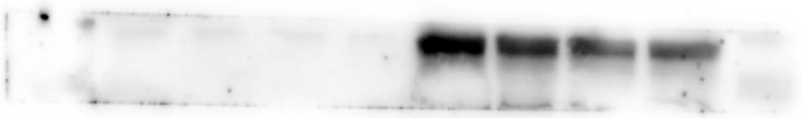

TUBULIN

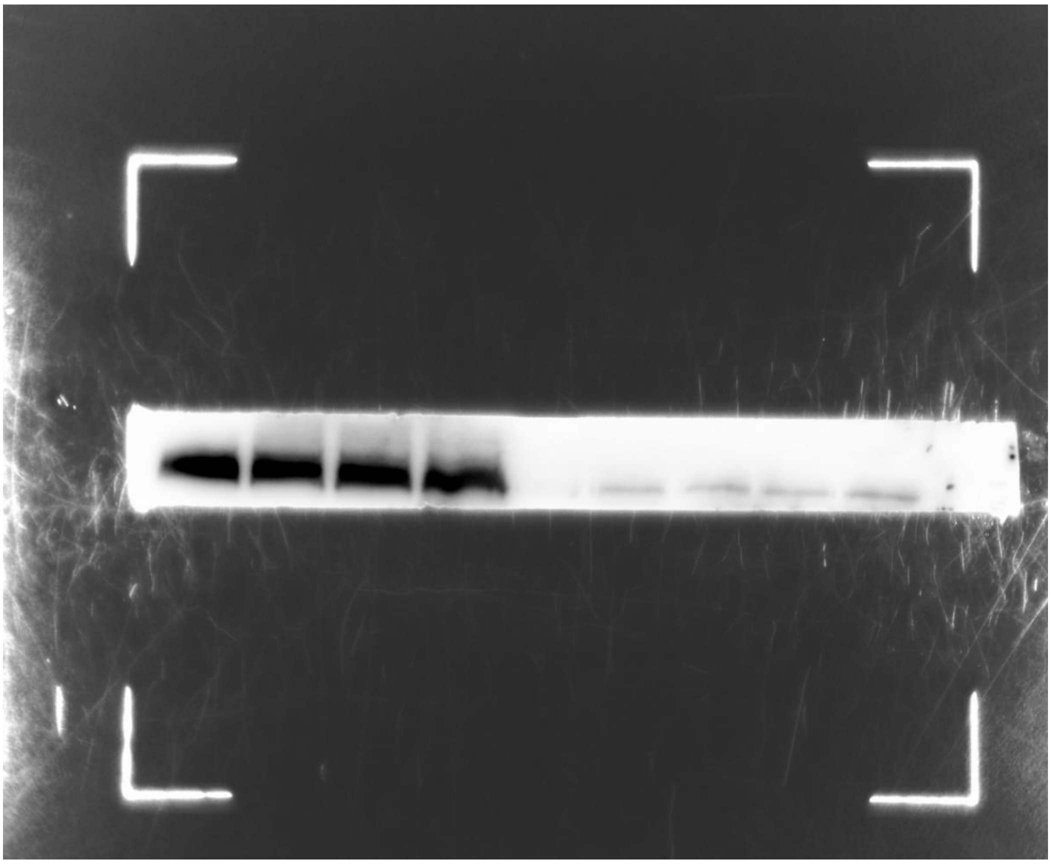

F4 L

BAX

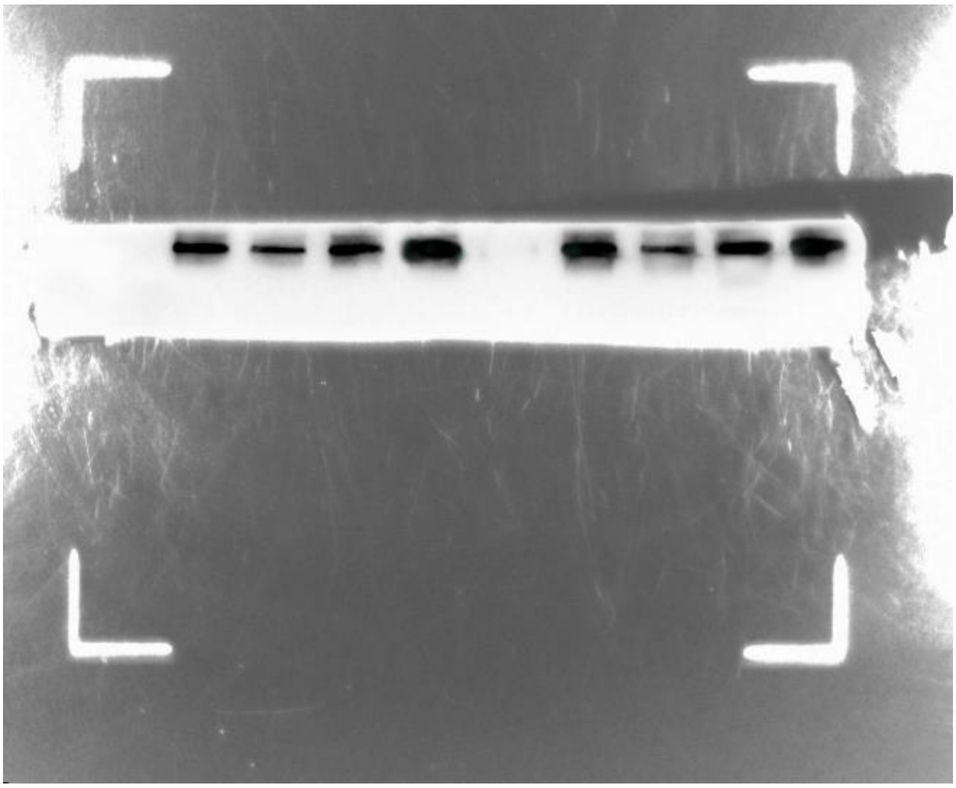

TOMM40

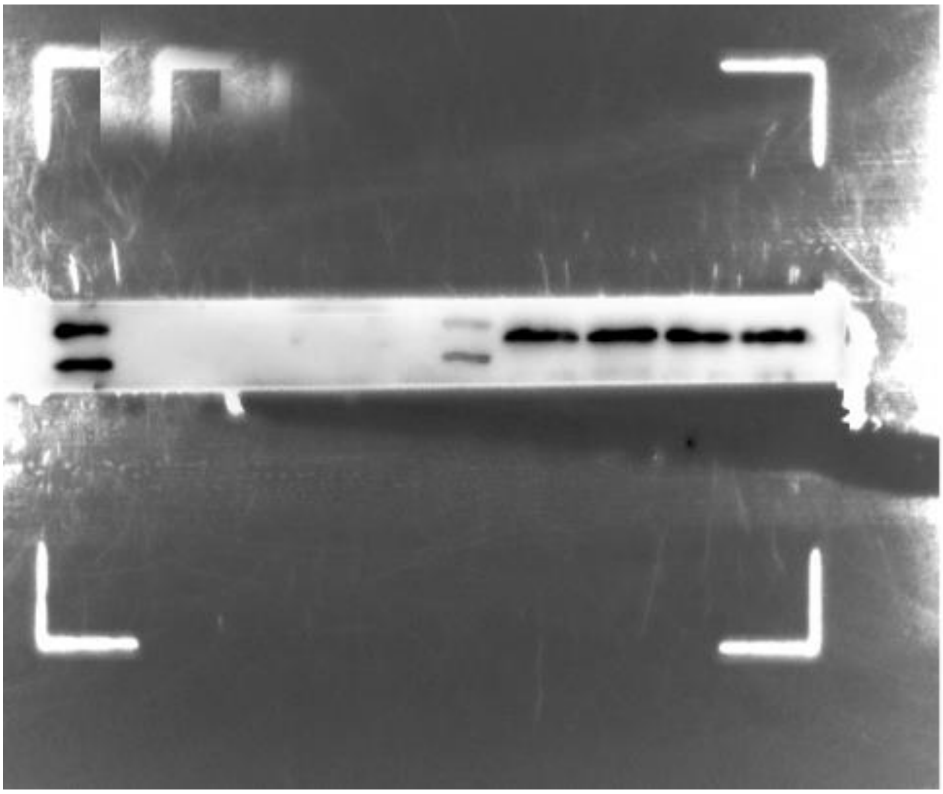

GAPDH

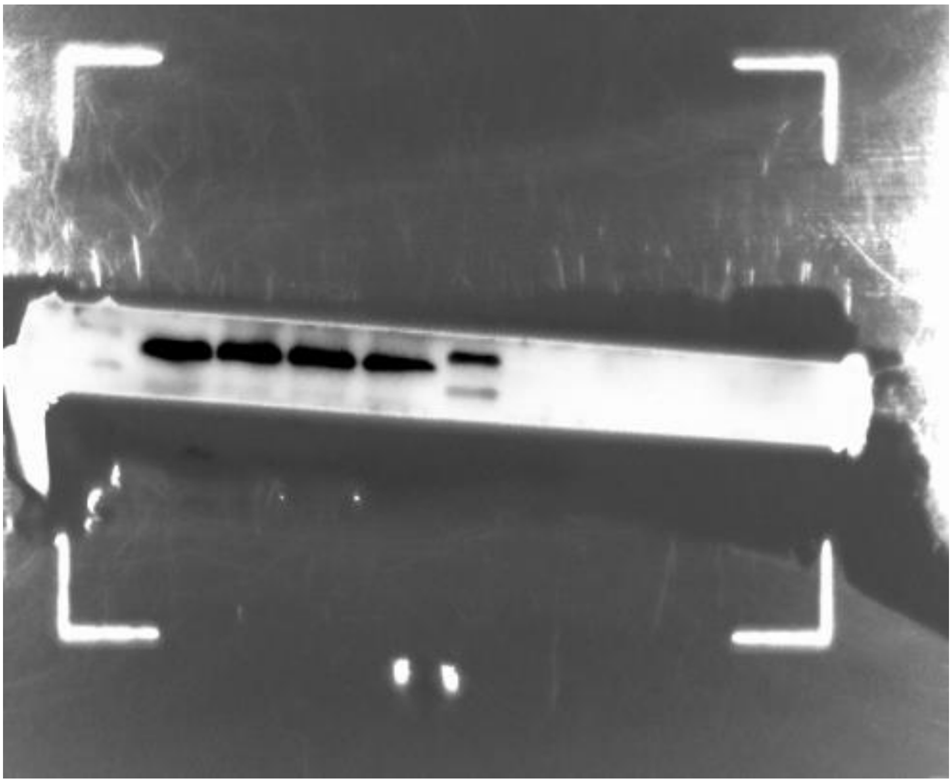

F5 C

Bax

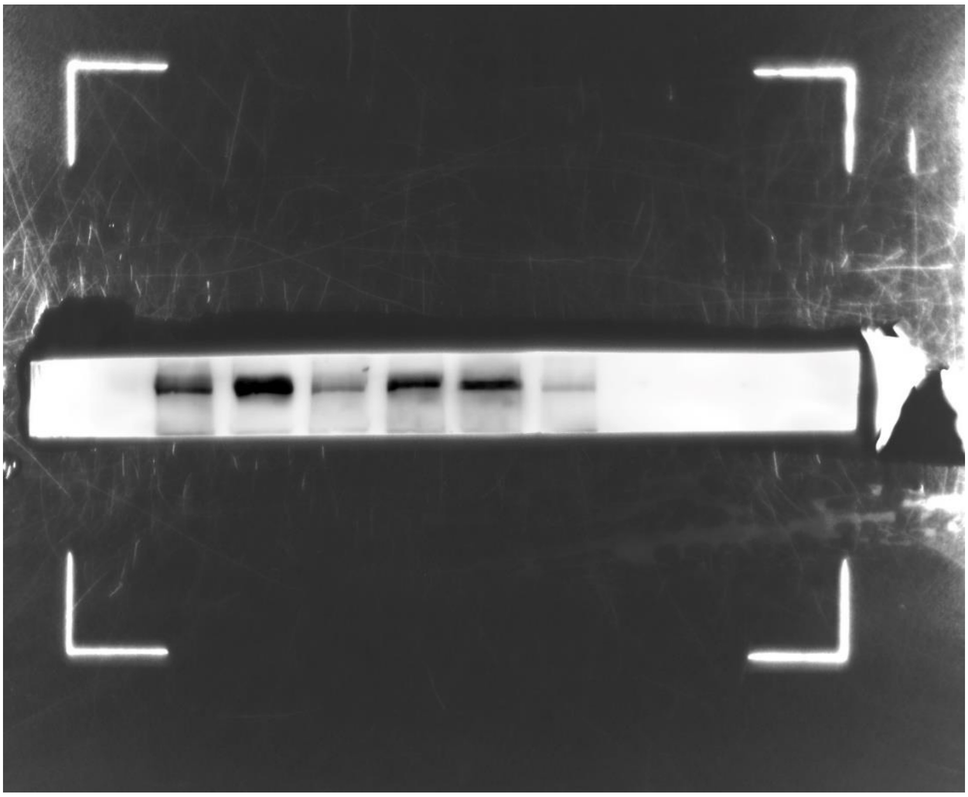

Caspase3

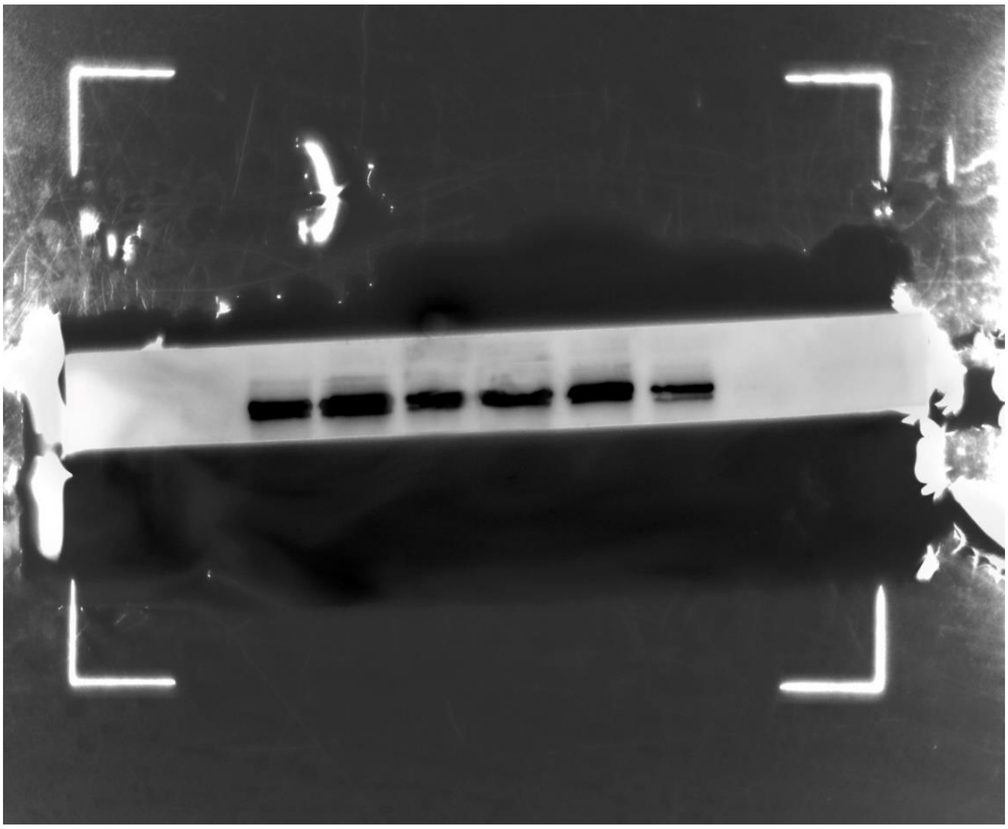

CRABP2

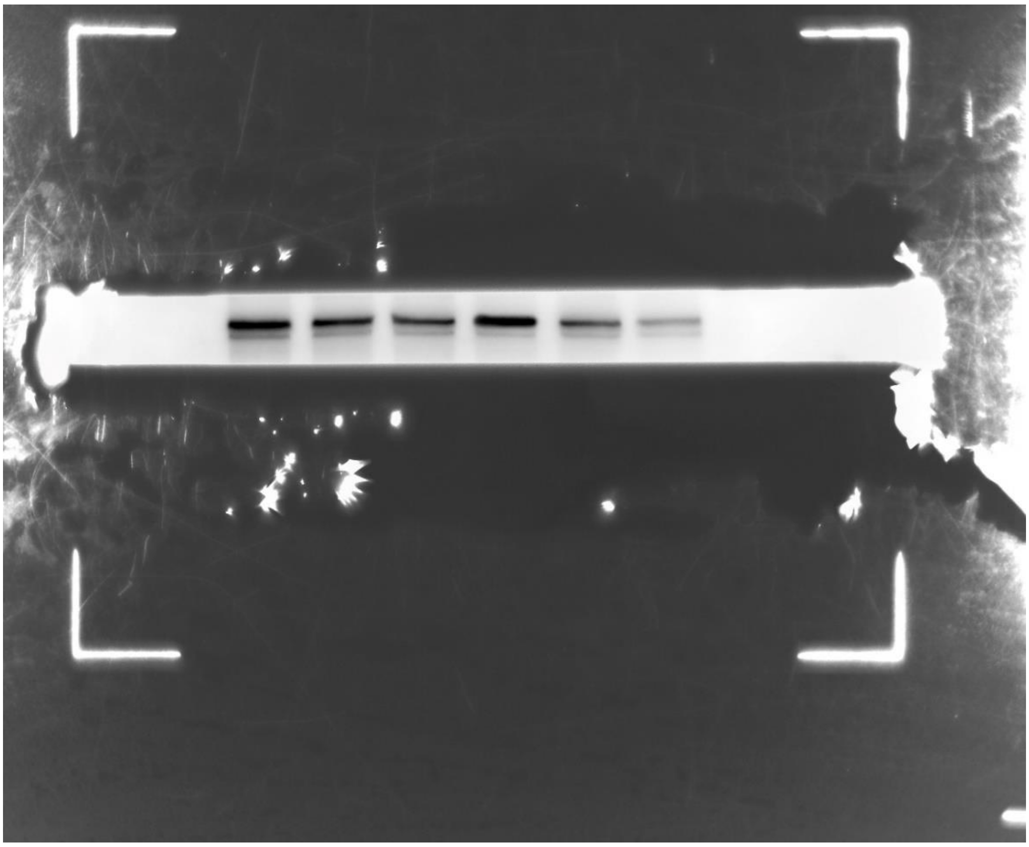

GAPDH

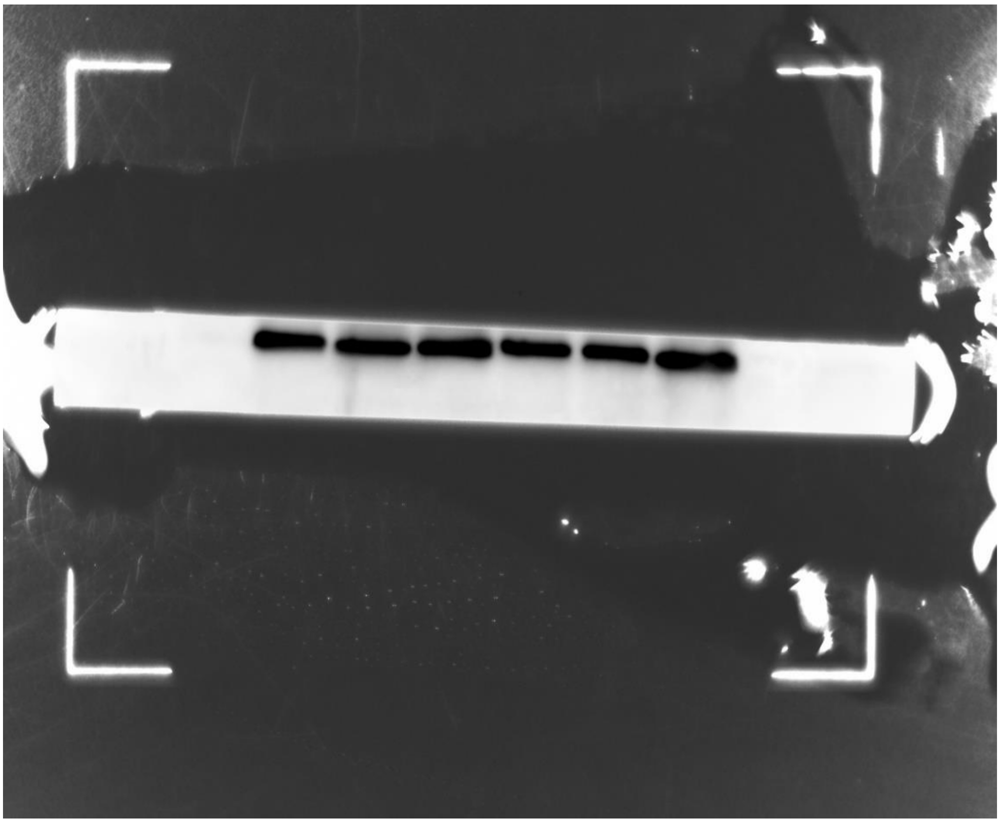

F5 H

BAX

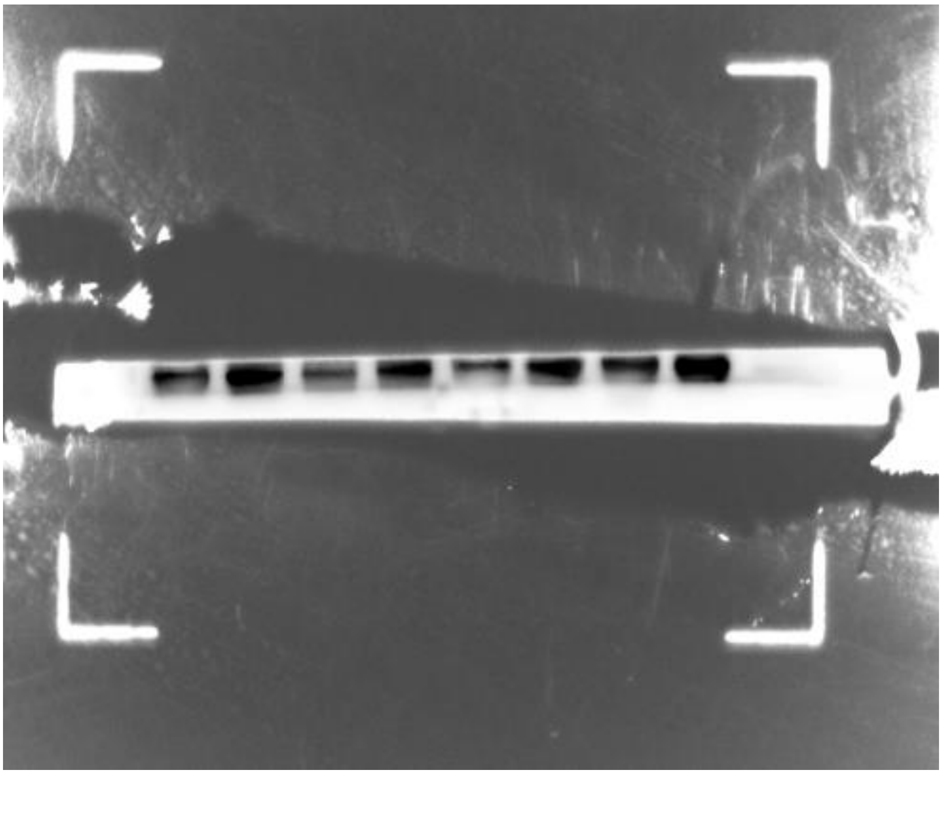

GAPDH

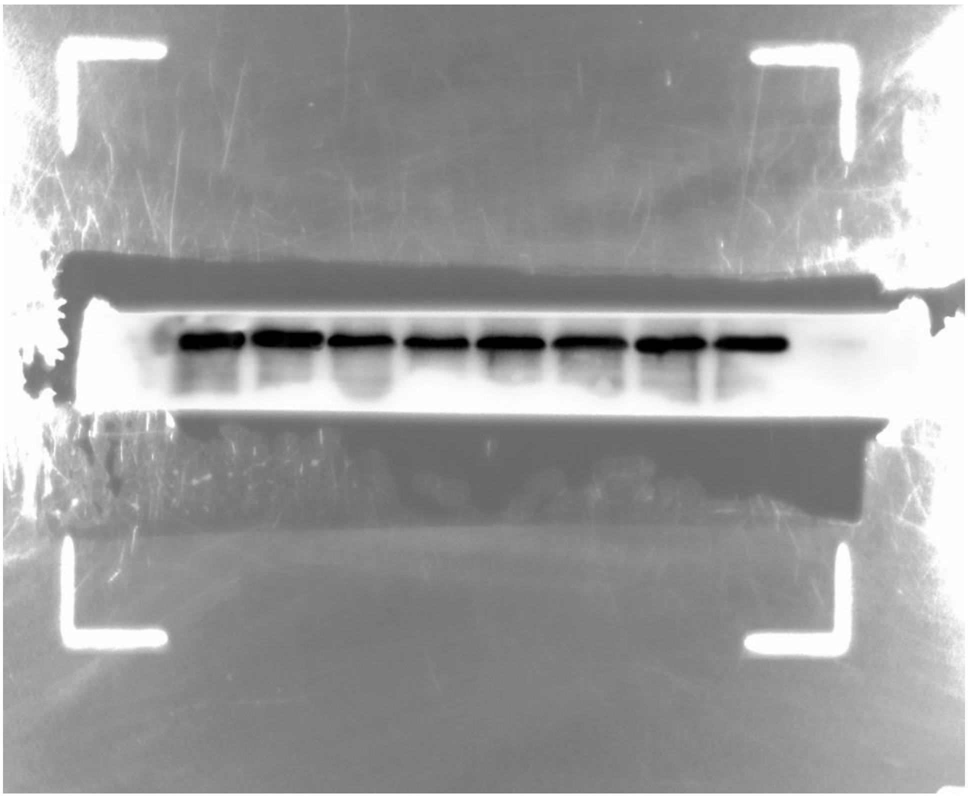

CRABP2

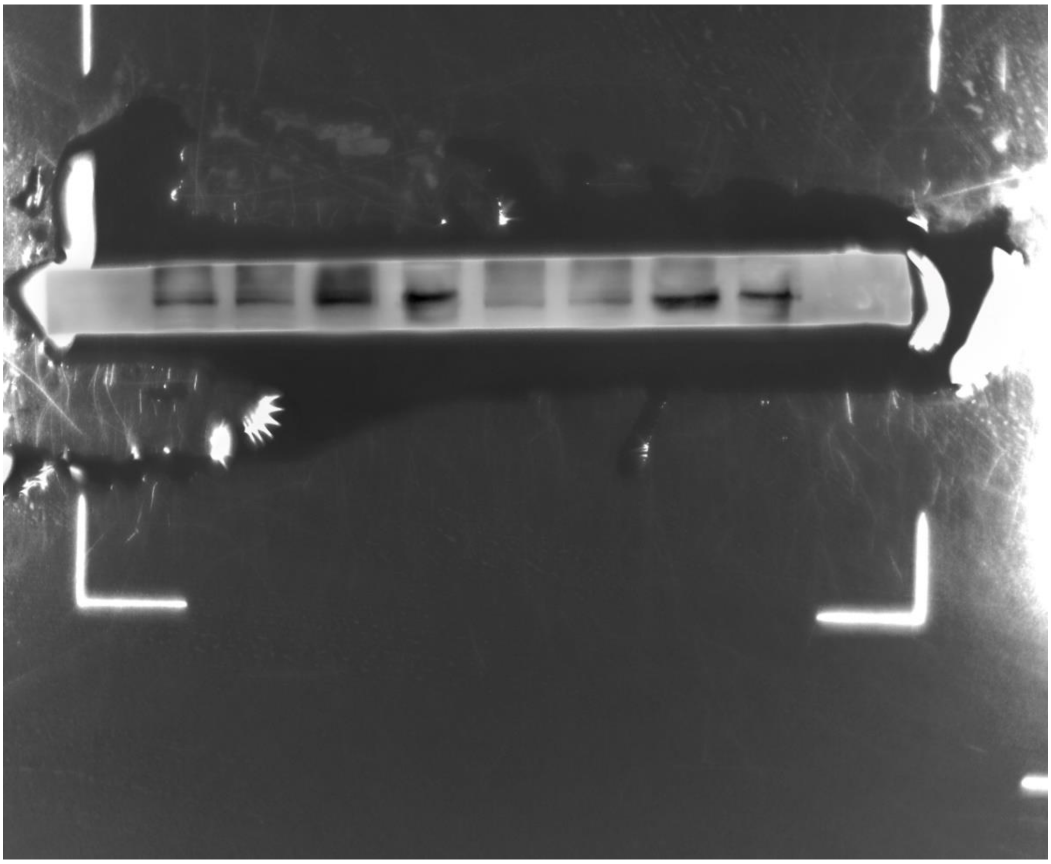

F6 D

TET1

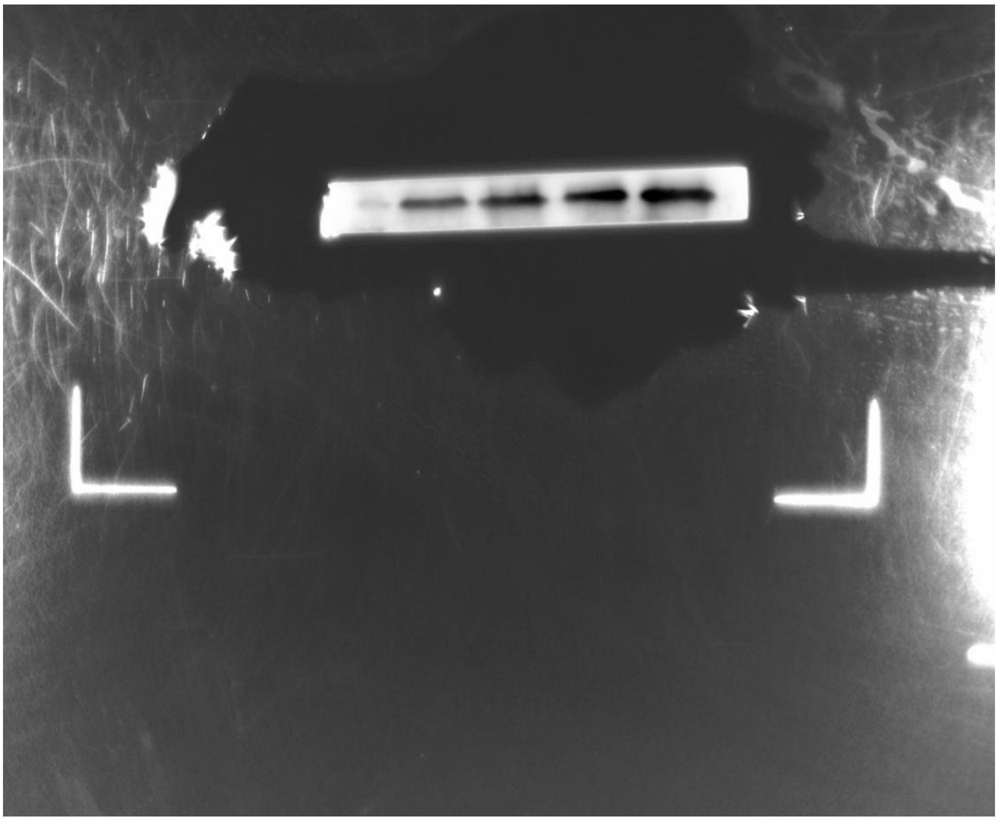

GAPDH

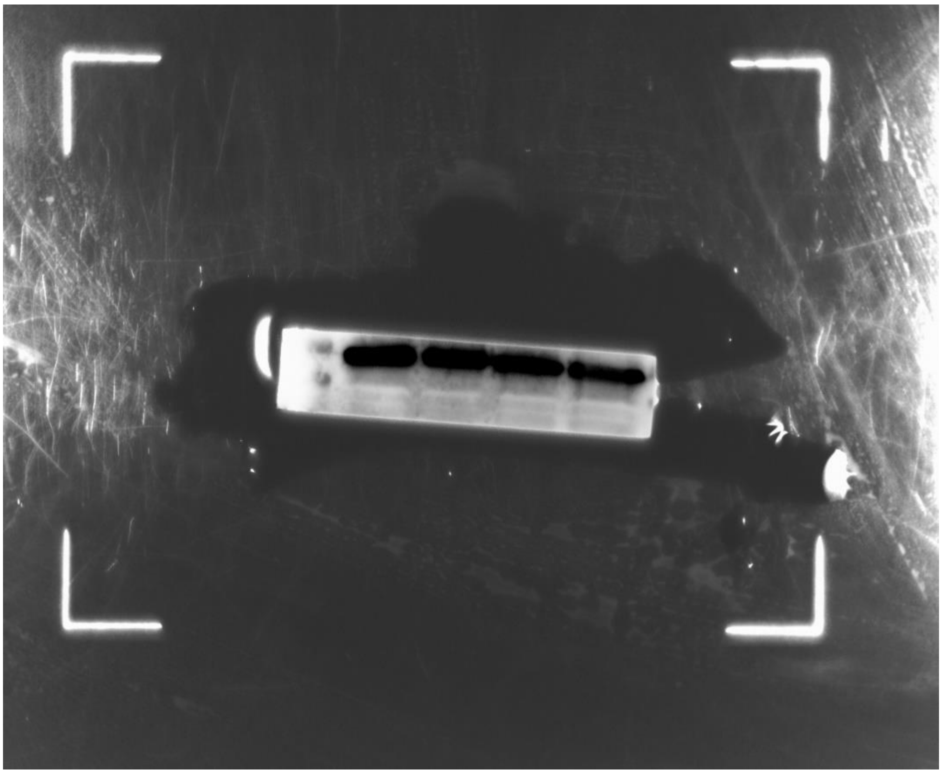

DNMT3A

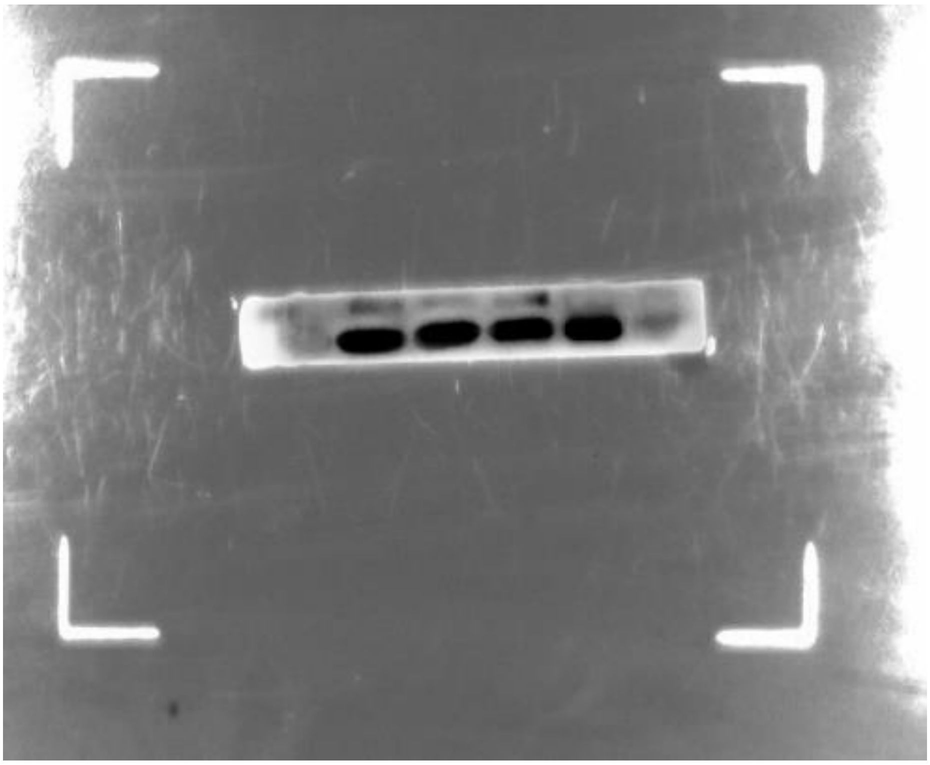

DNMT3B

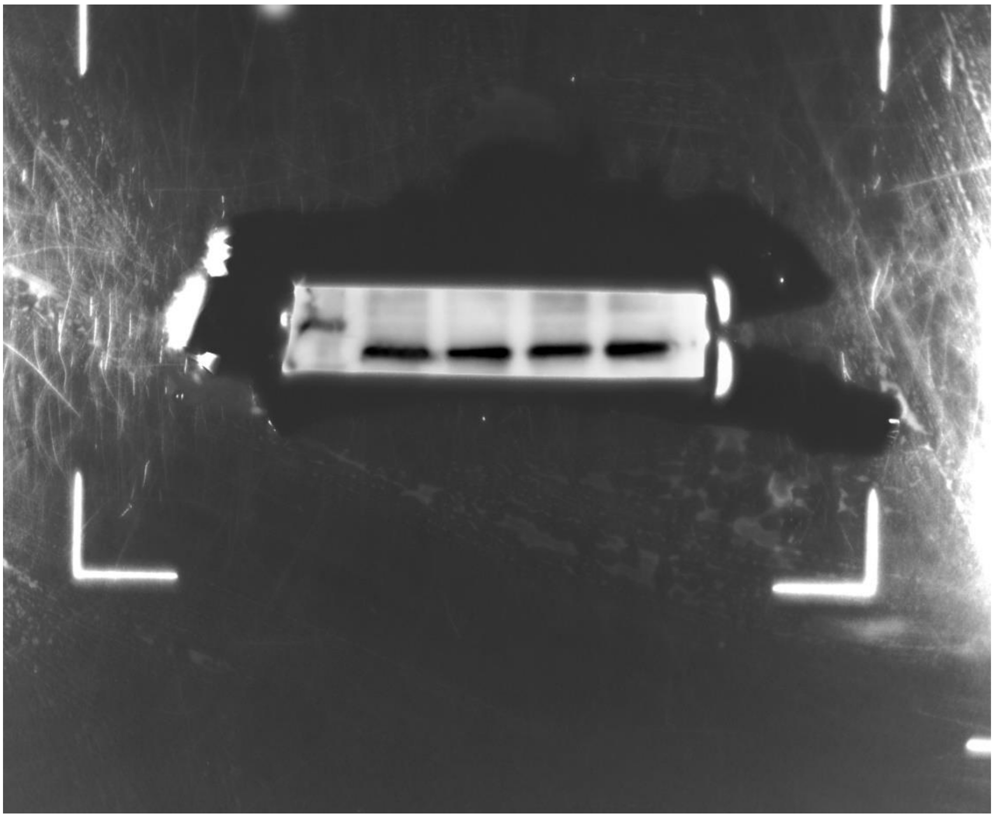

F6J

TET1

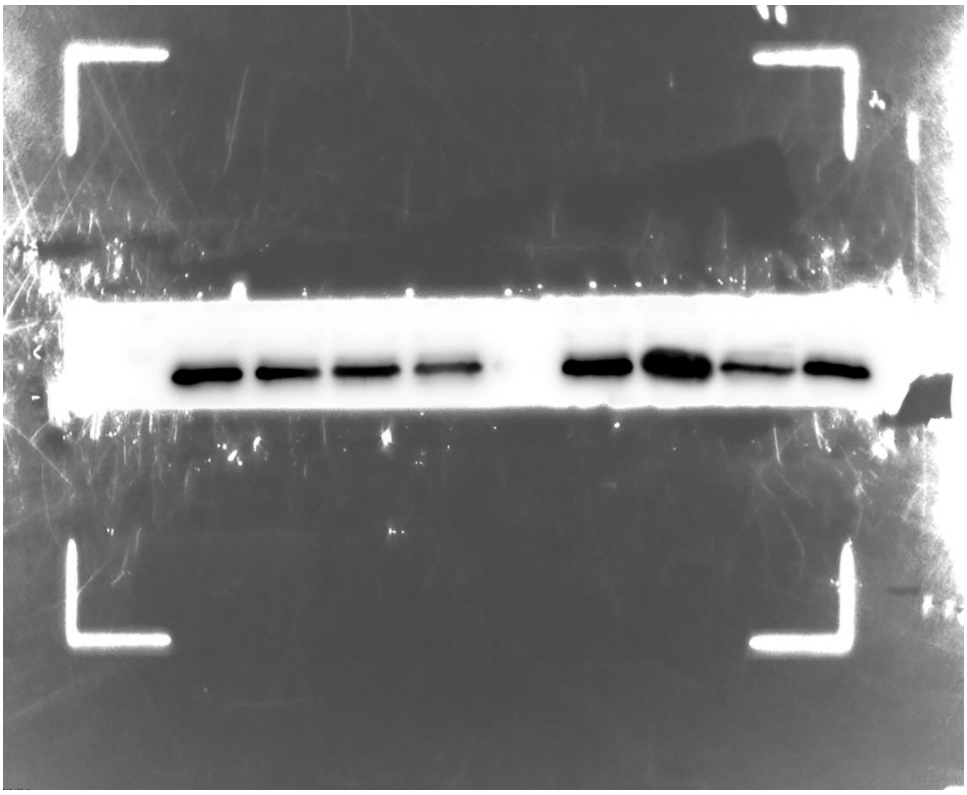

GAPDH

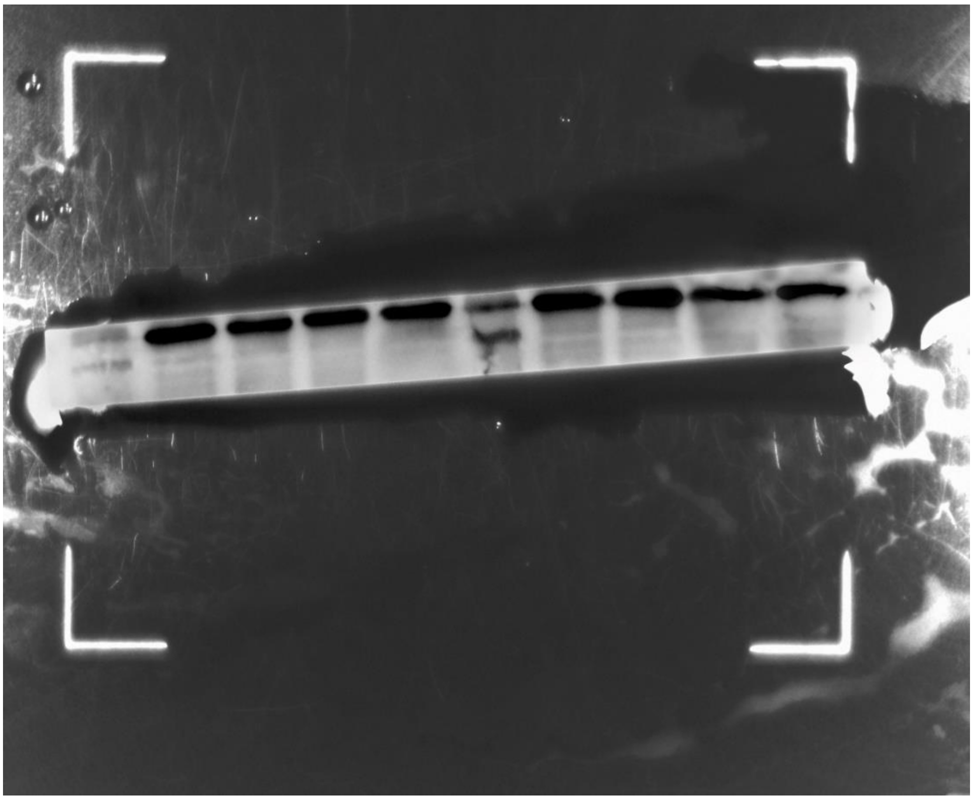

CRABP2

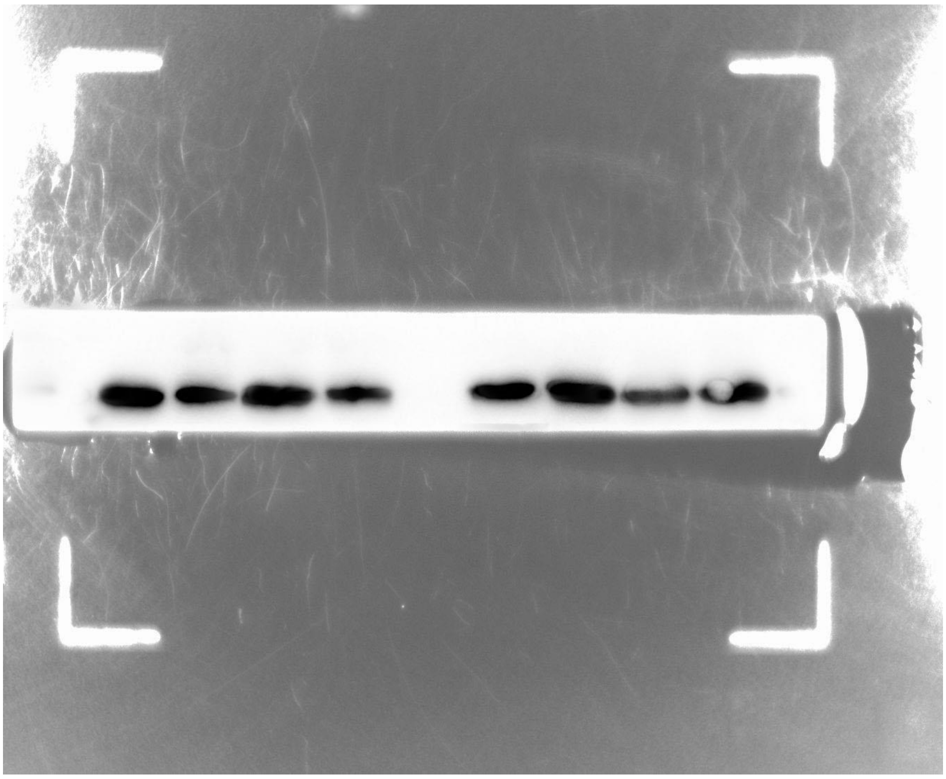

F7 E

GAPDH

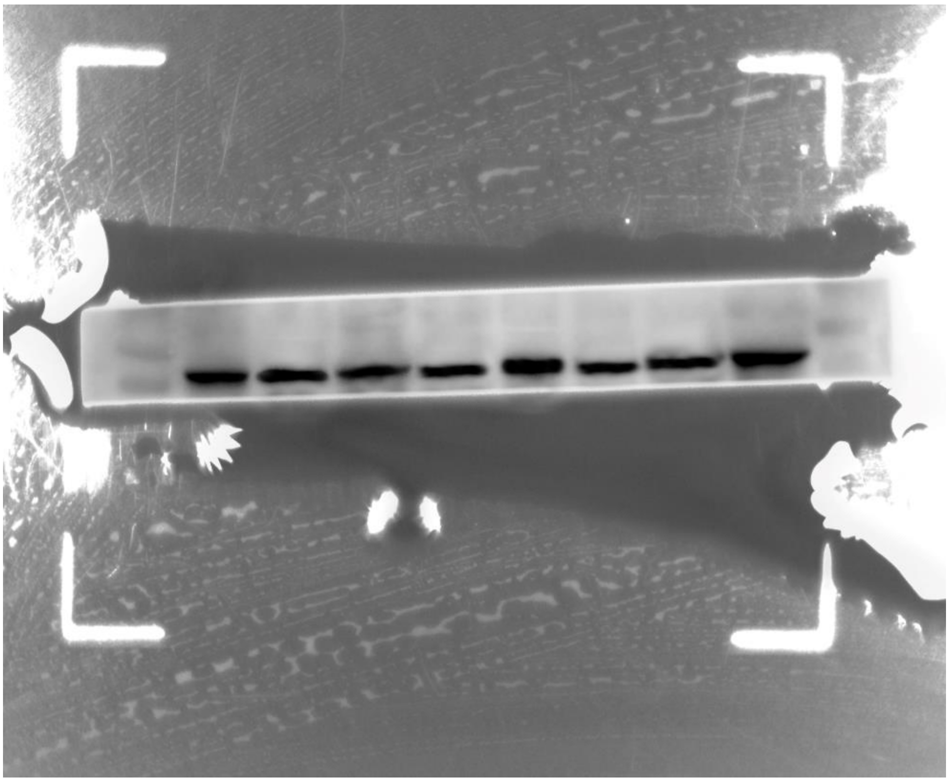

CRABP2

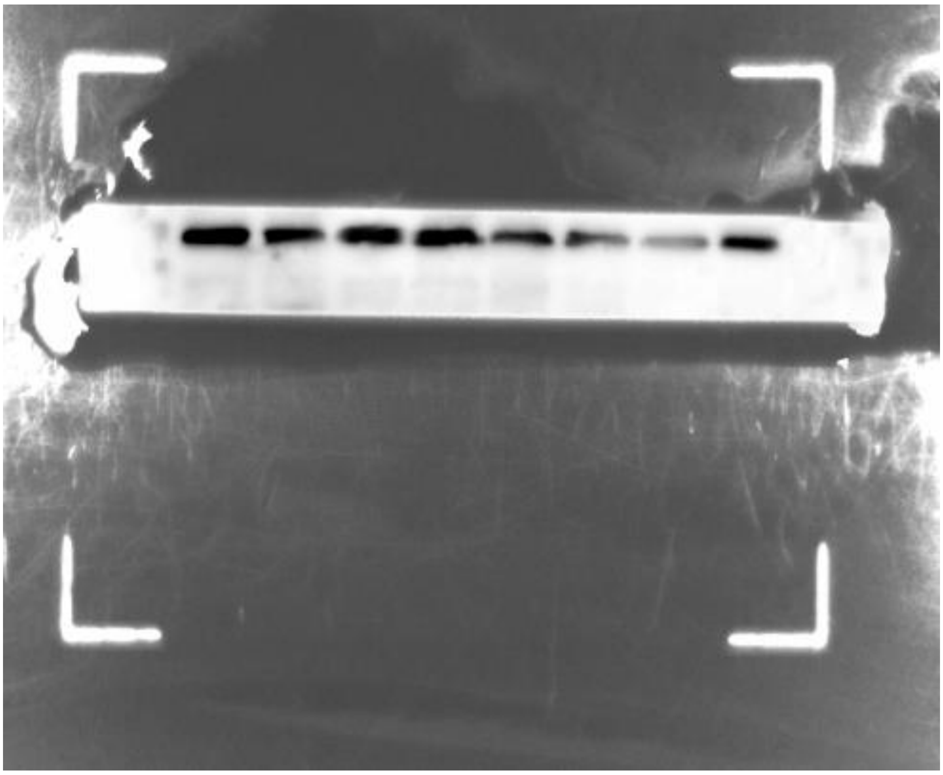

BAX

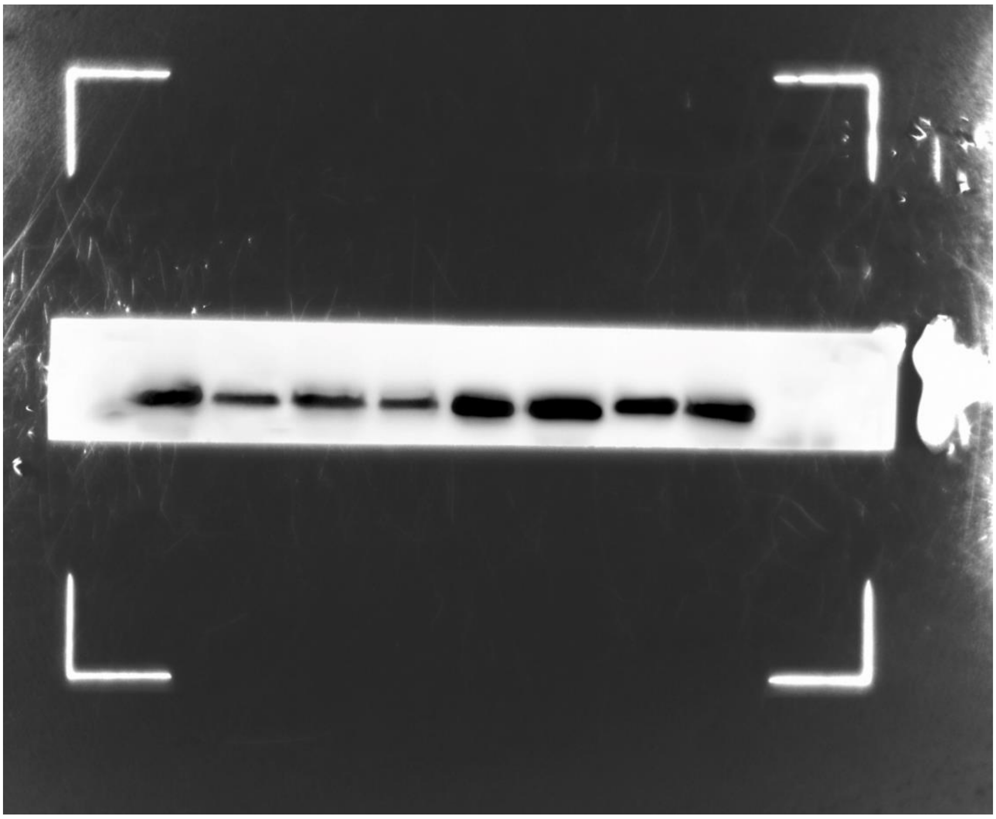

S7A

A

CRABP2

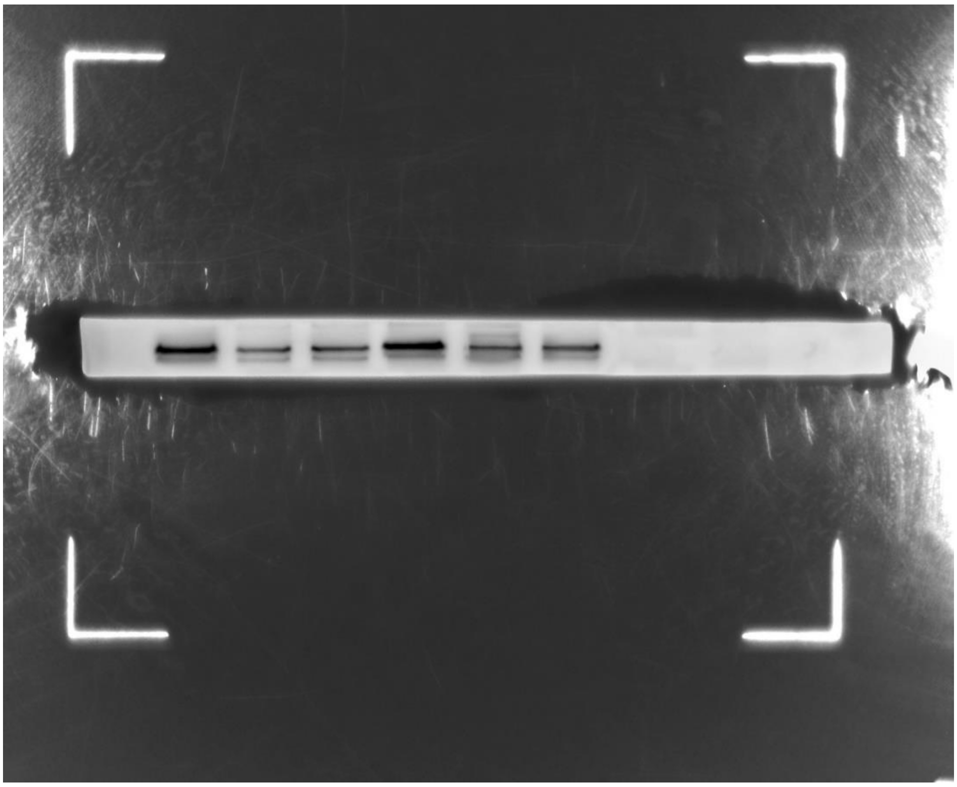

GAPDH

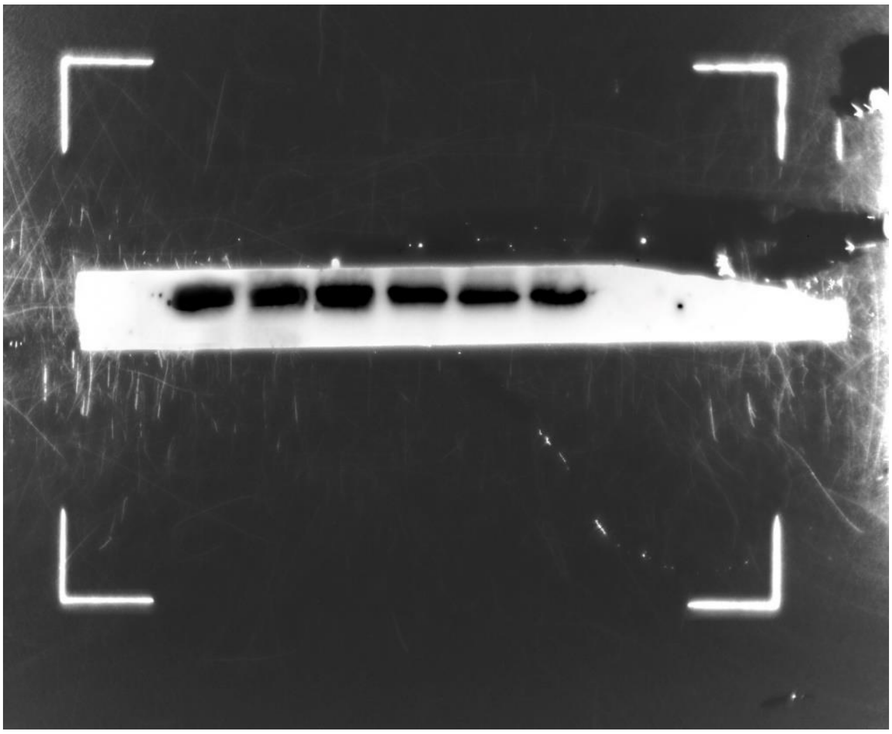

7B

BAX

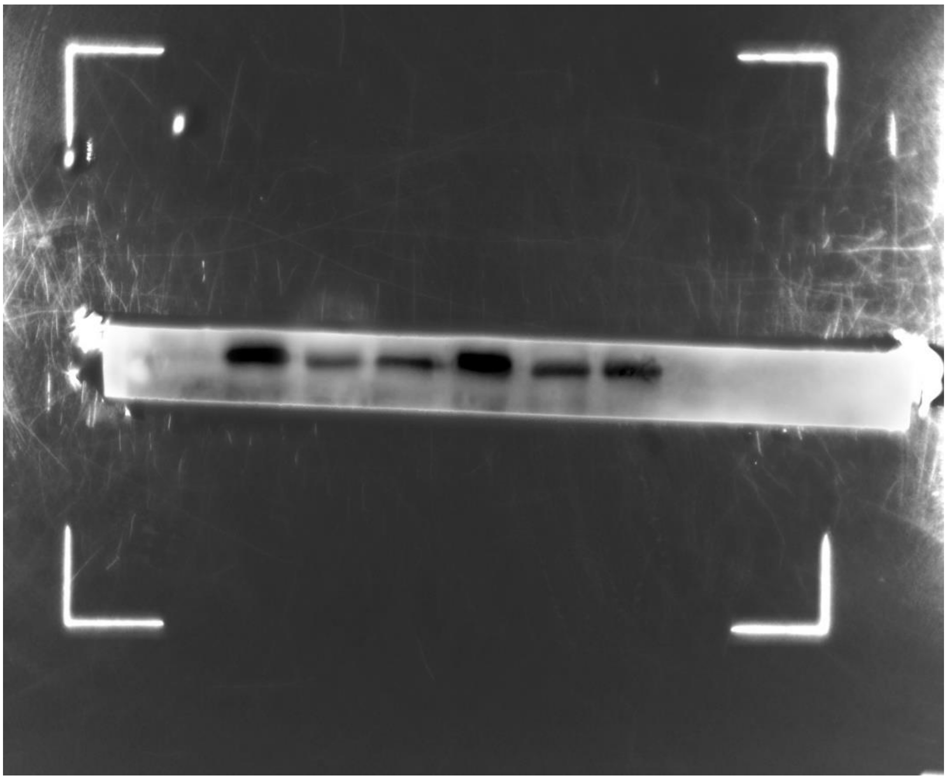

GAPDH

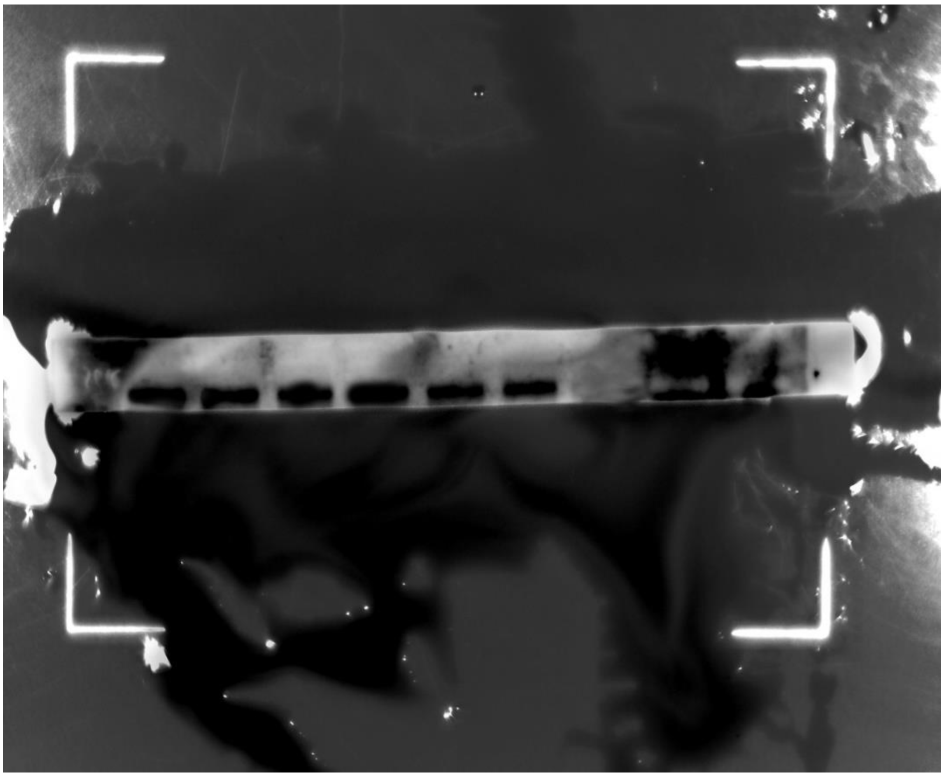

7C

PARKIN

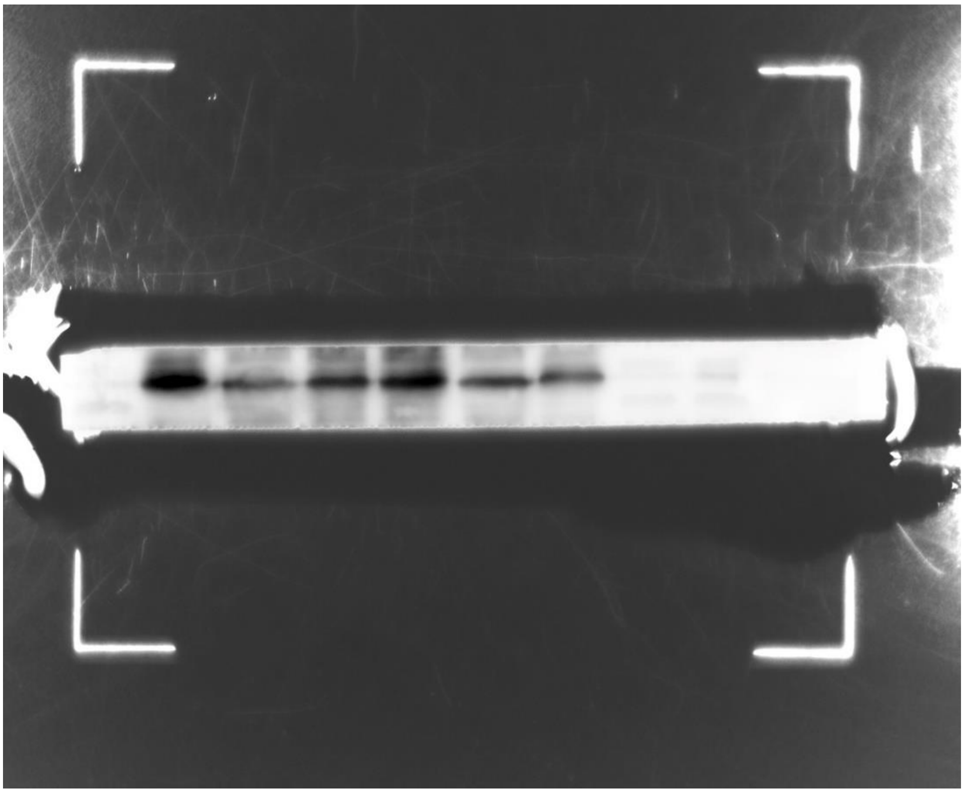

GAPDH

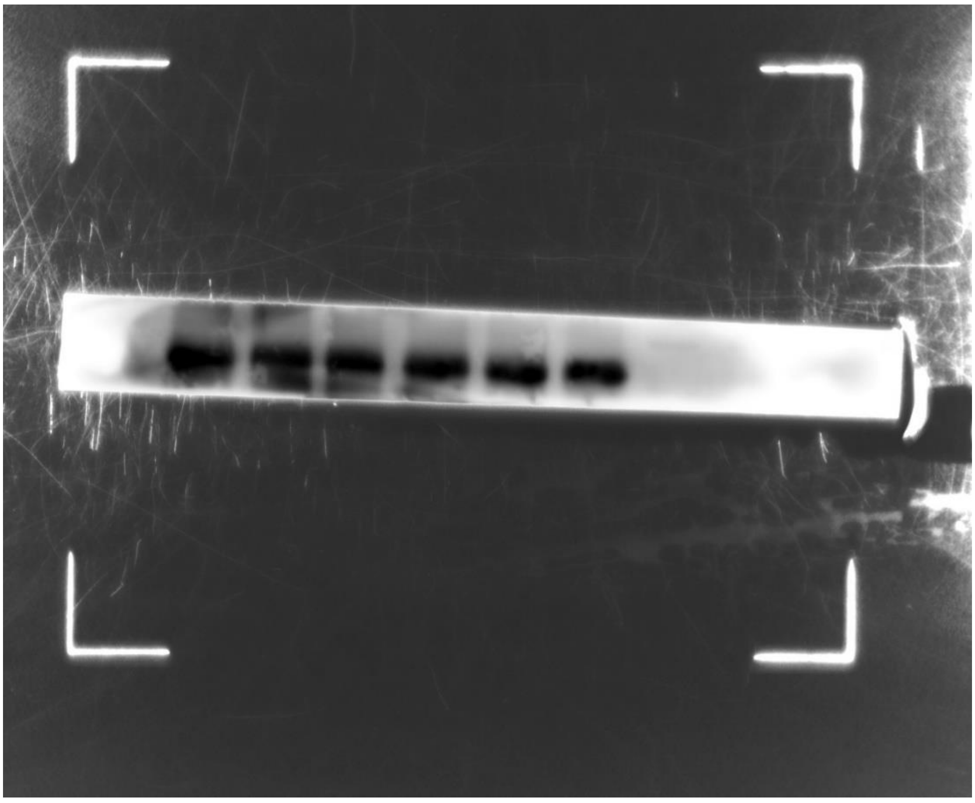

7D

TET1

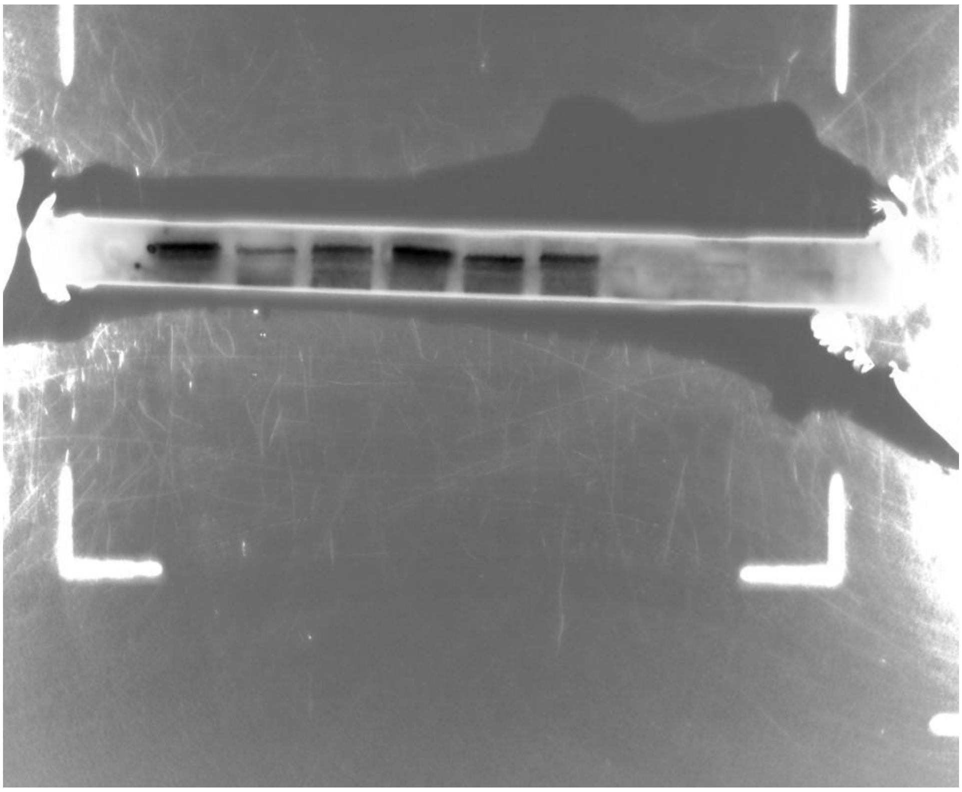

GAPDH

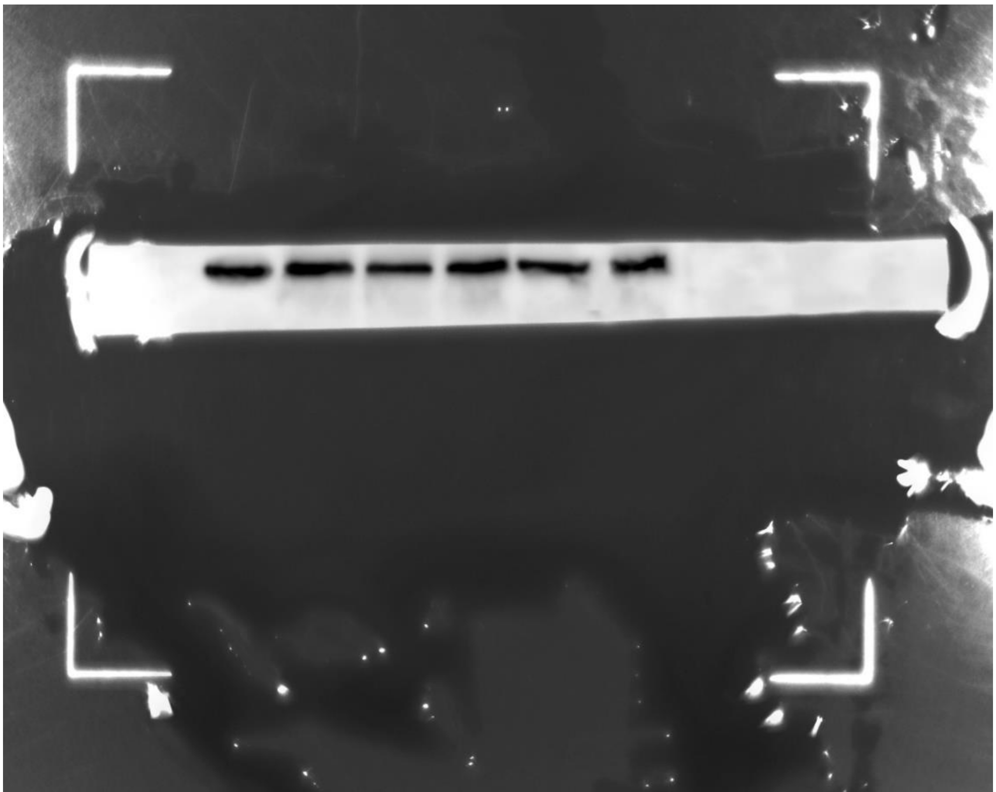

S7E

GAPDH

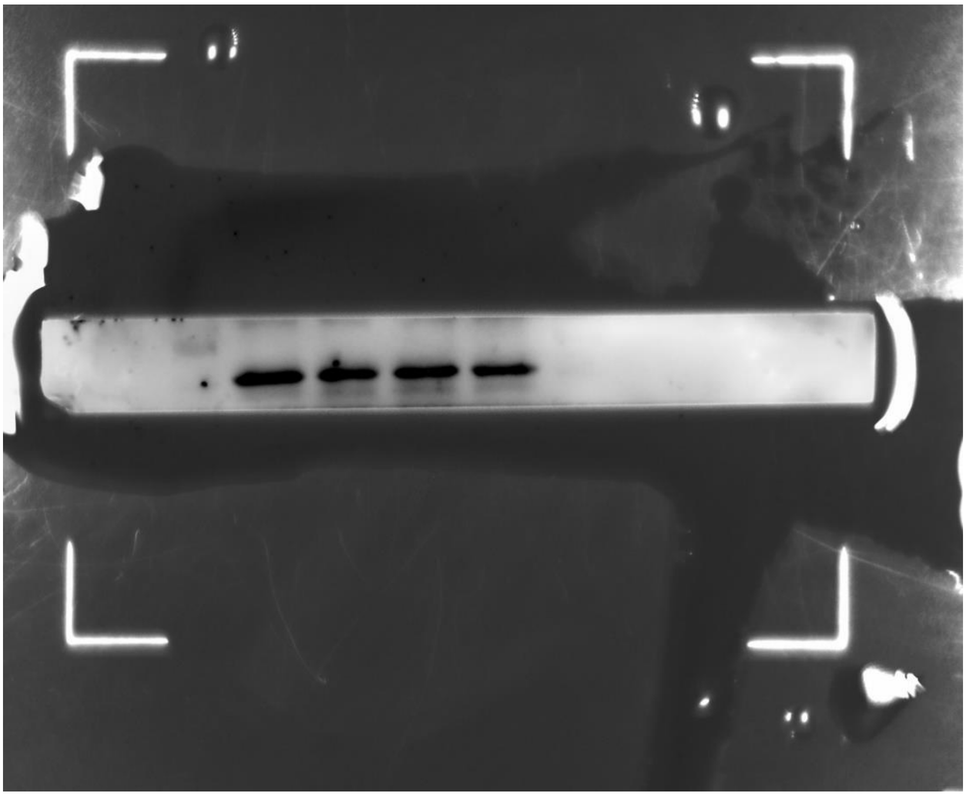

CRABP2

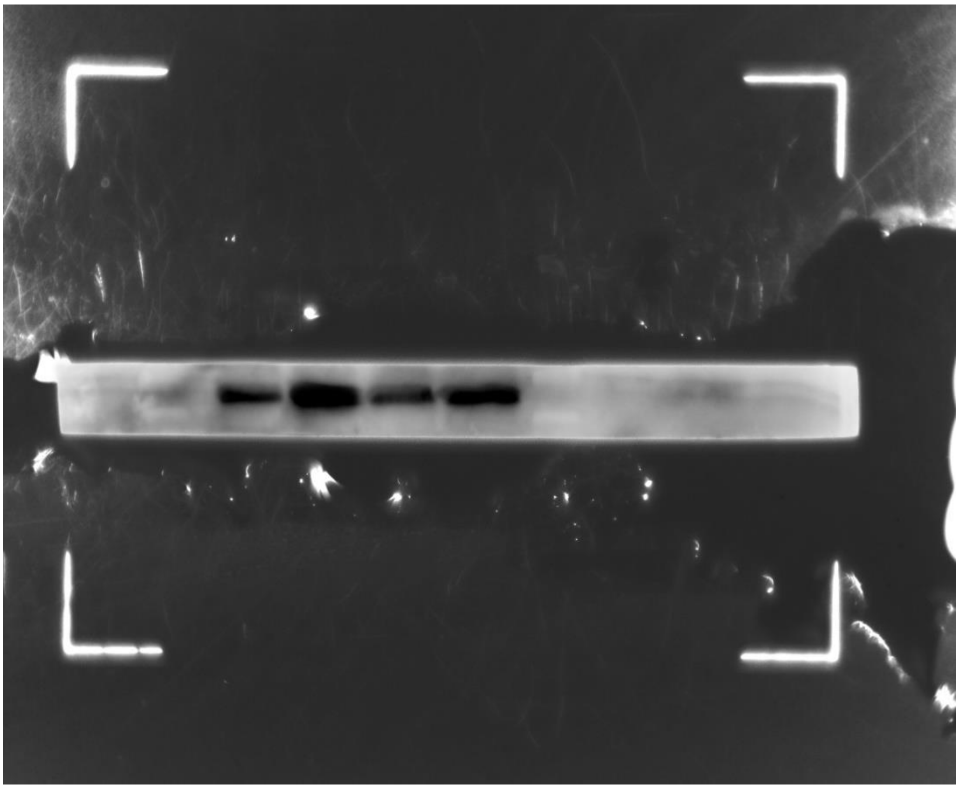

S7 F

GAPDH

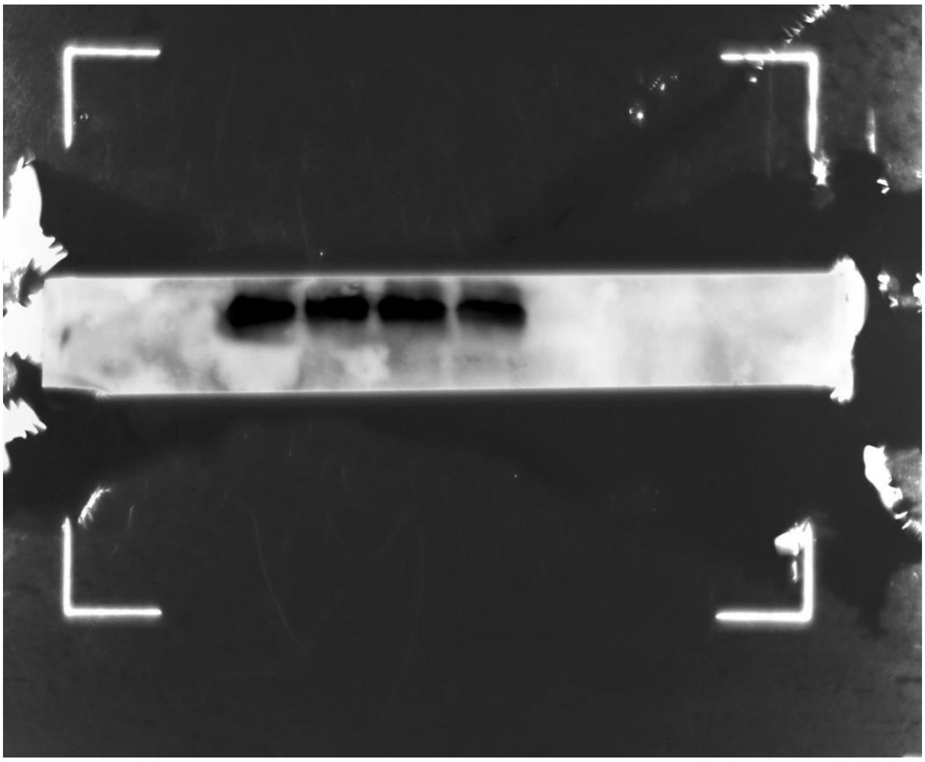

TET1

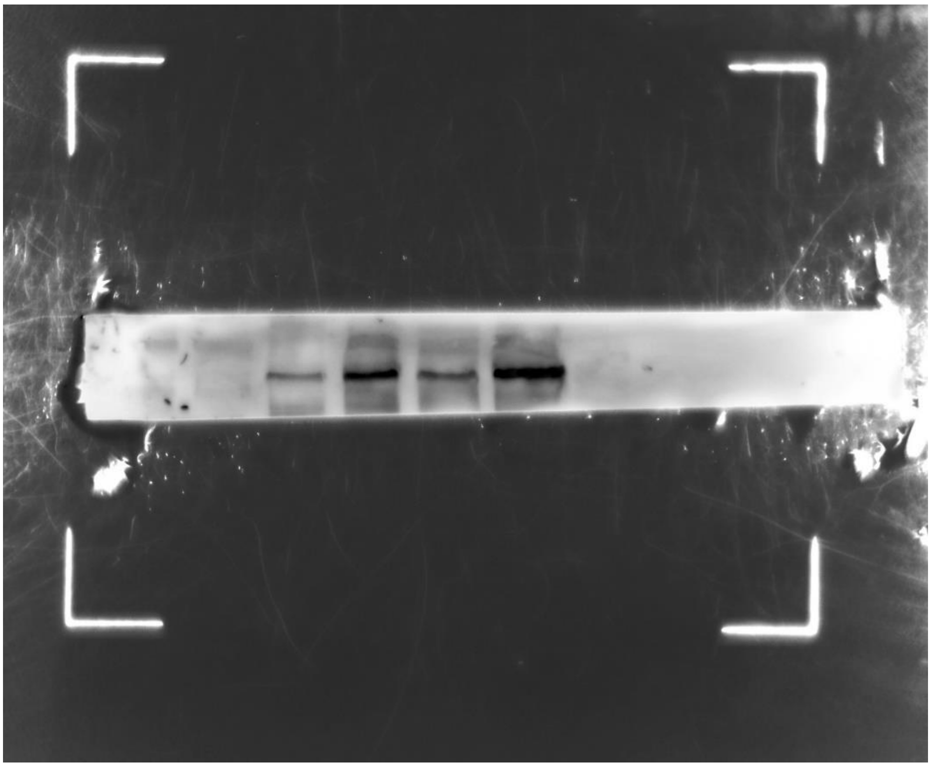

S5 A

BAX

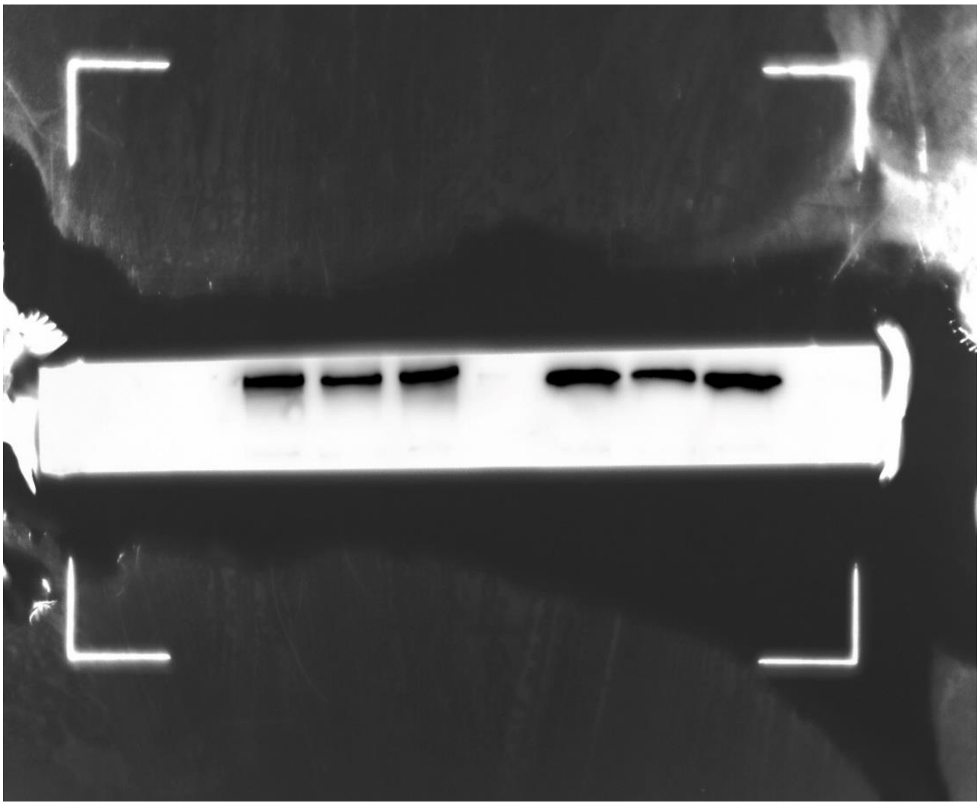

GAPDH

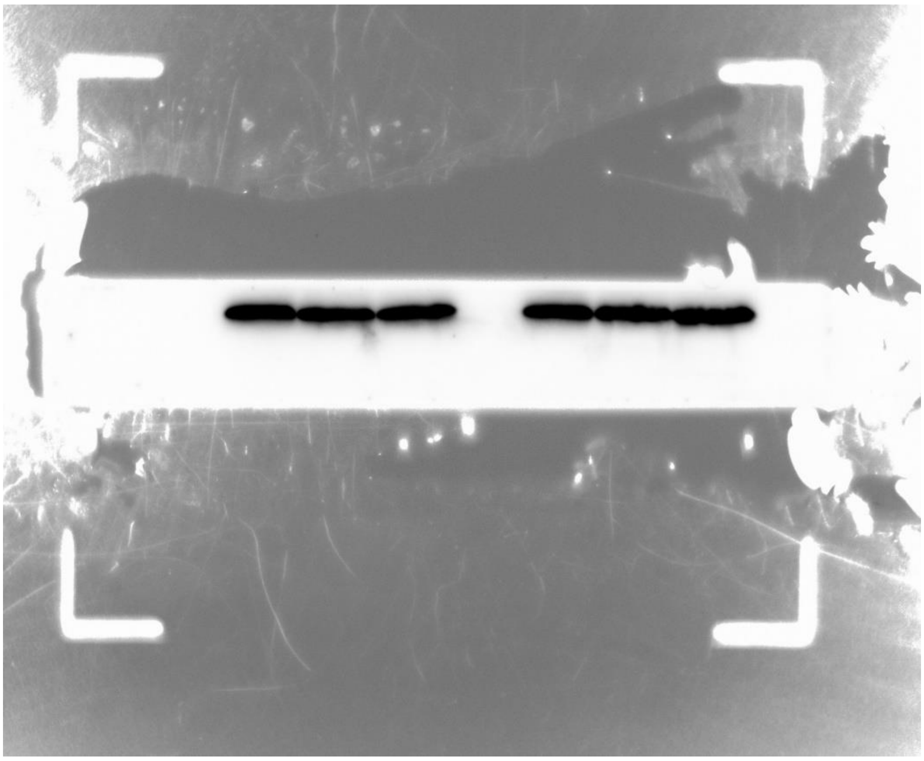

CRABP2

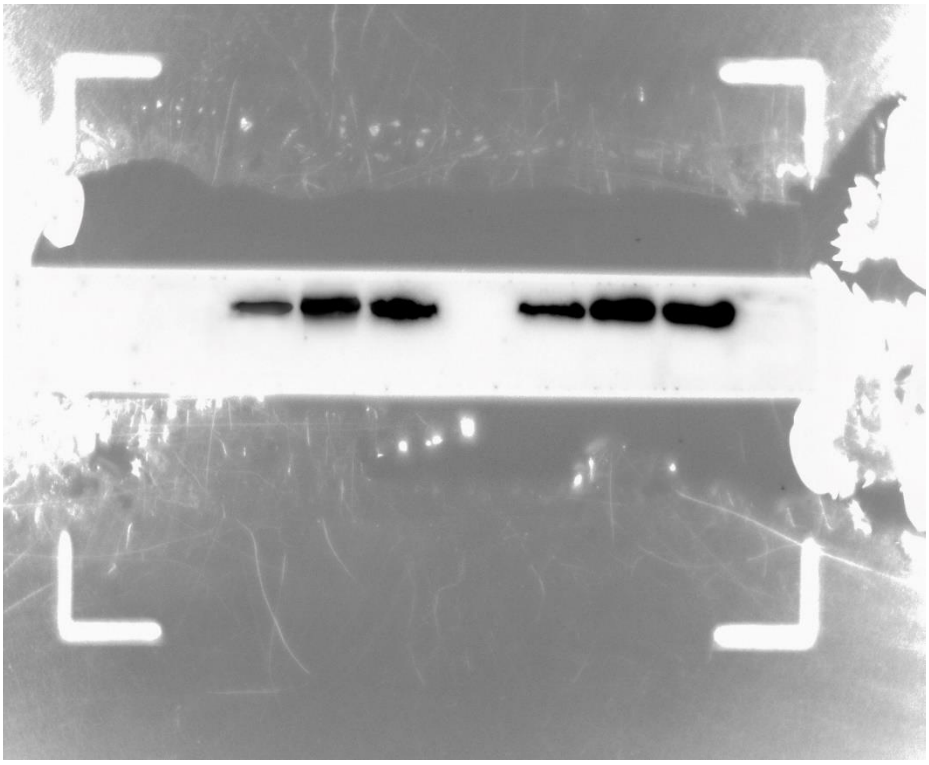

PARKIN

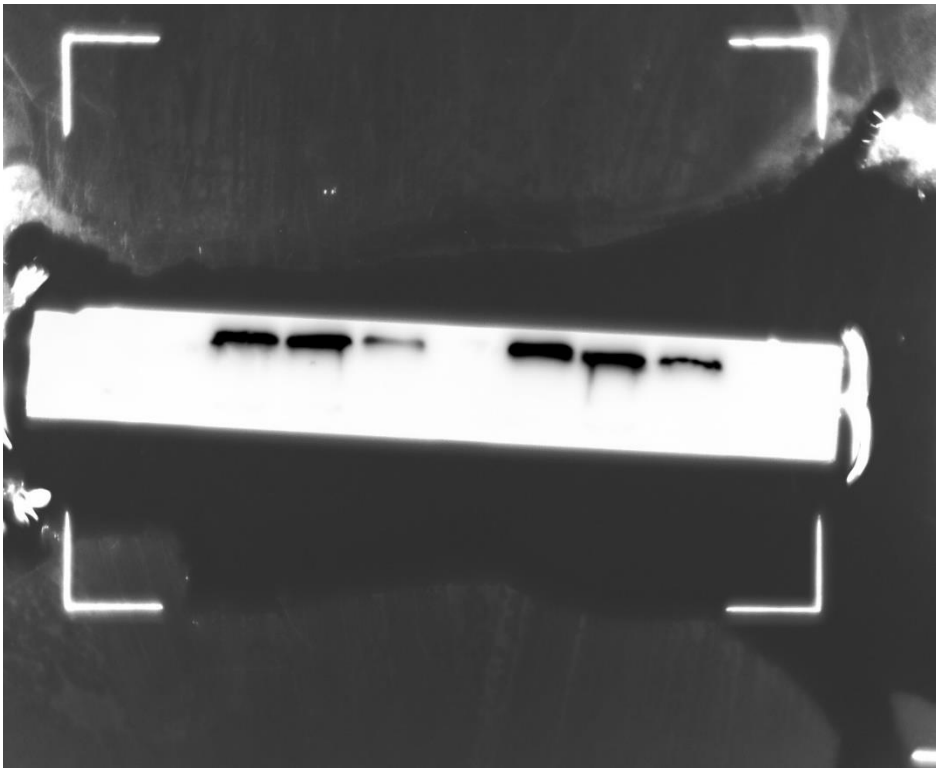

CASPASE-3

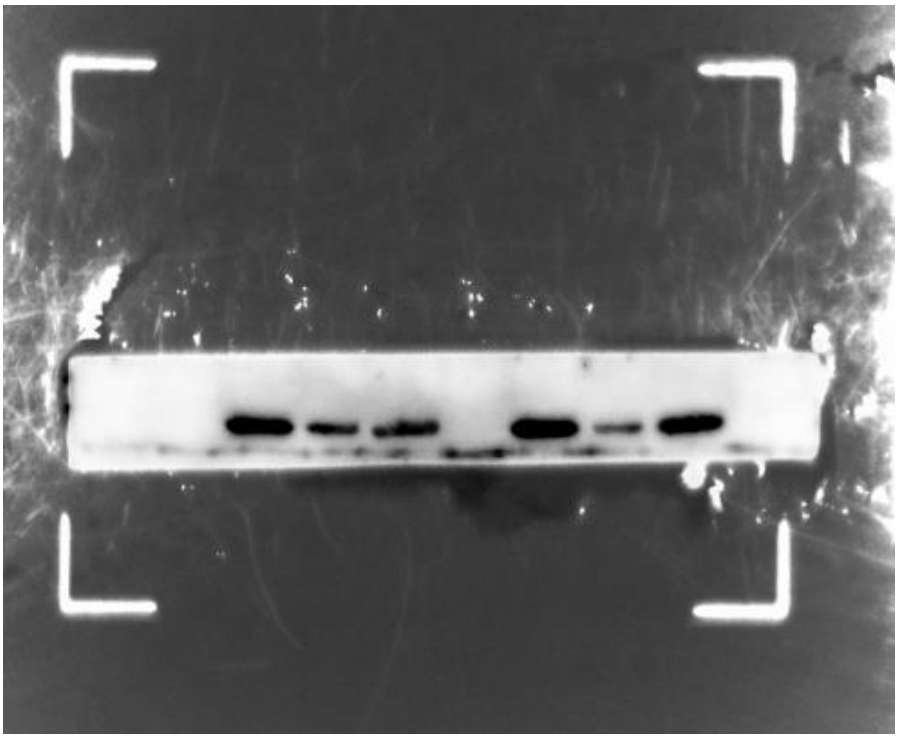

S5G

BAX

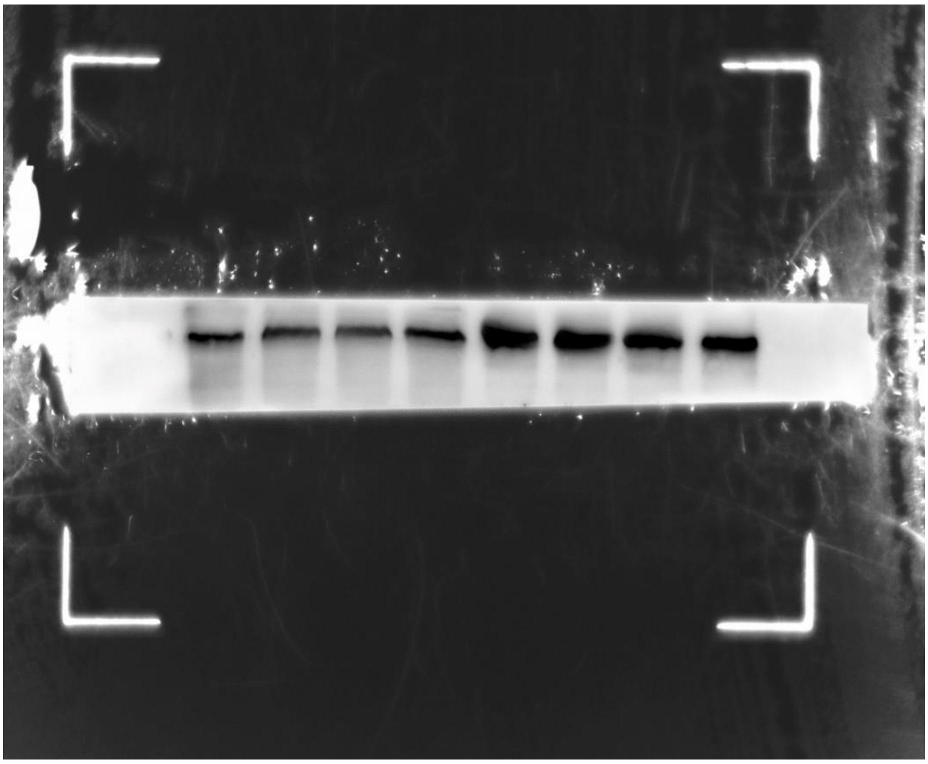

GAPDH

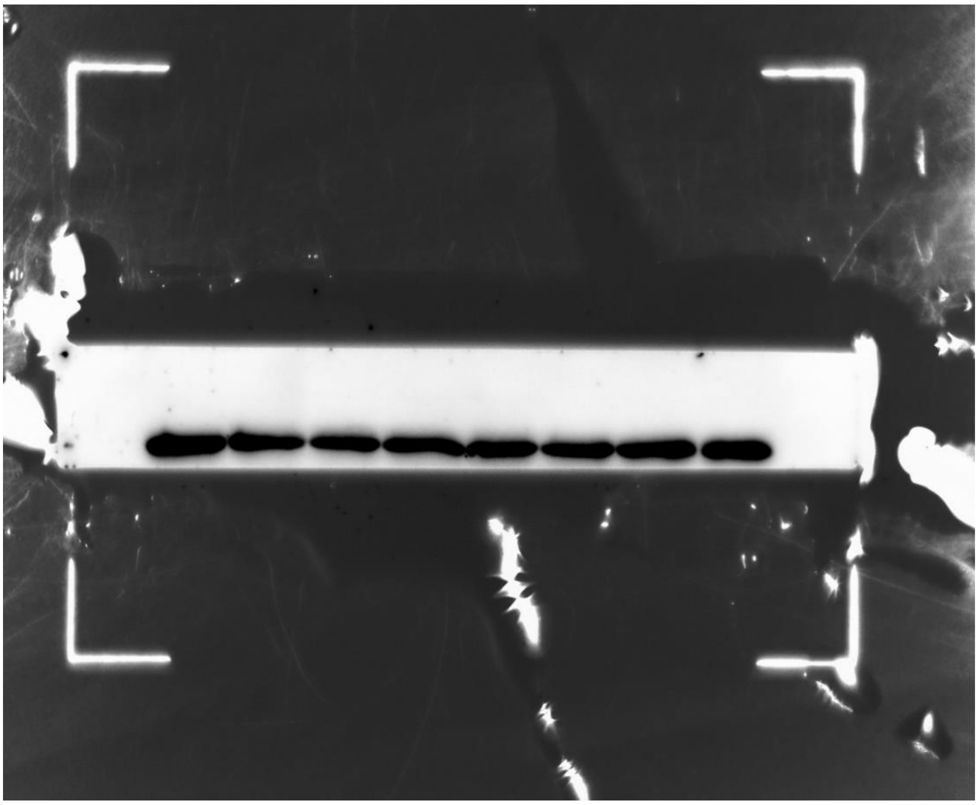

PARKIN

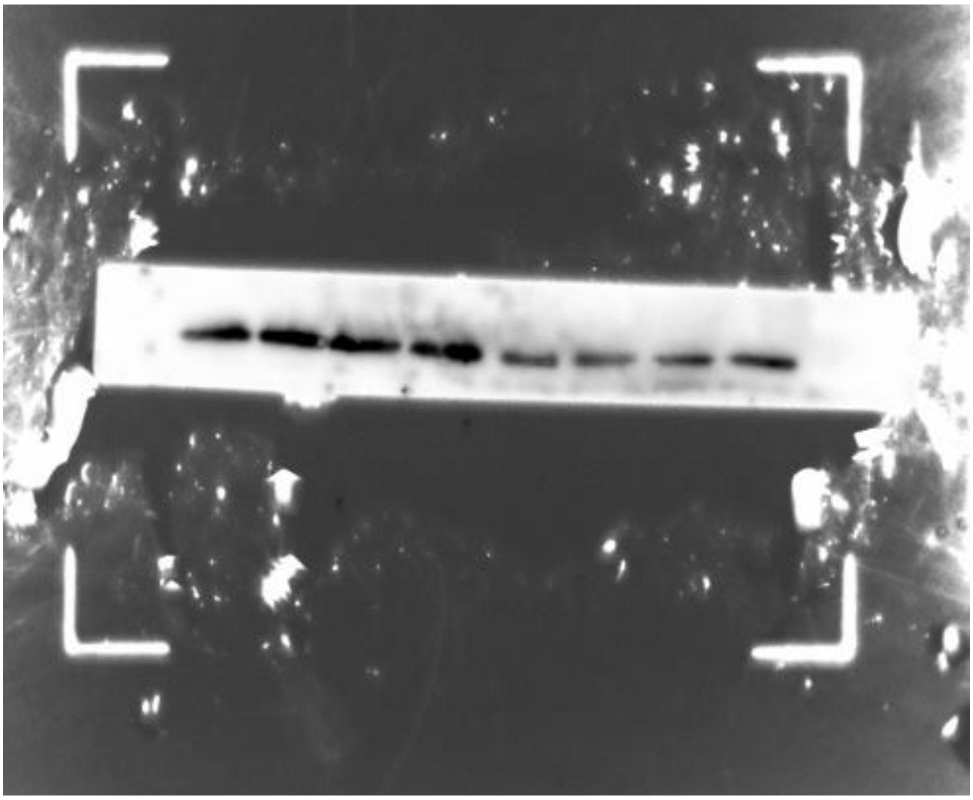

S6A LEFT

GAPDH

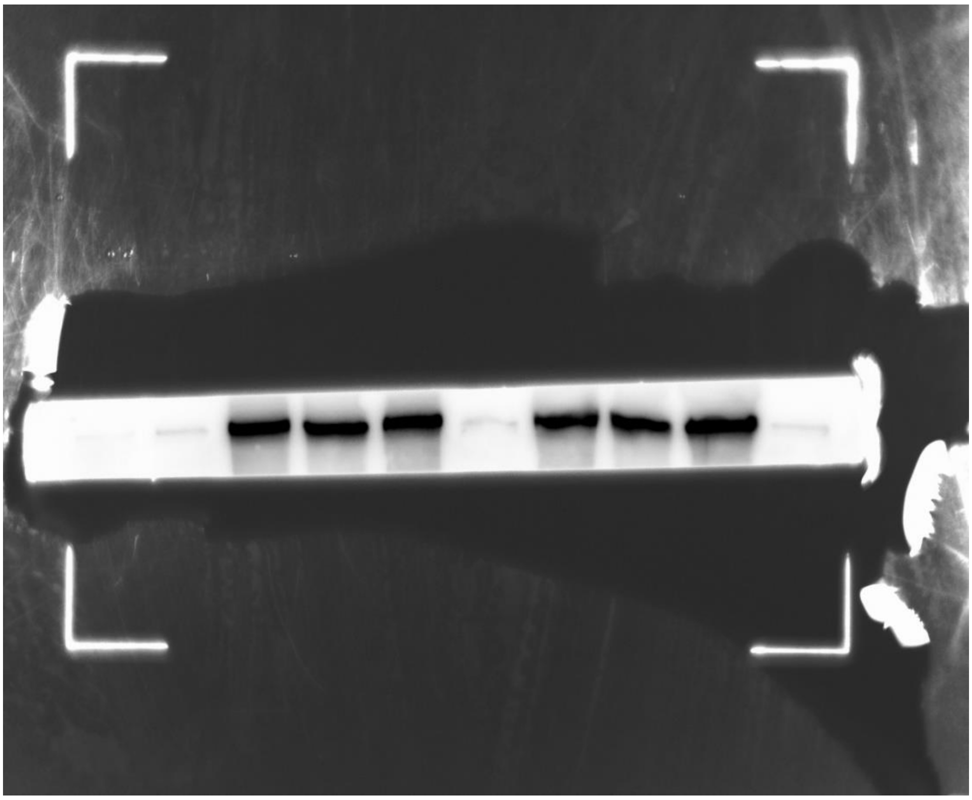

TET1

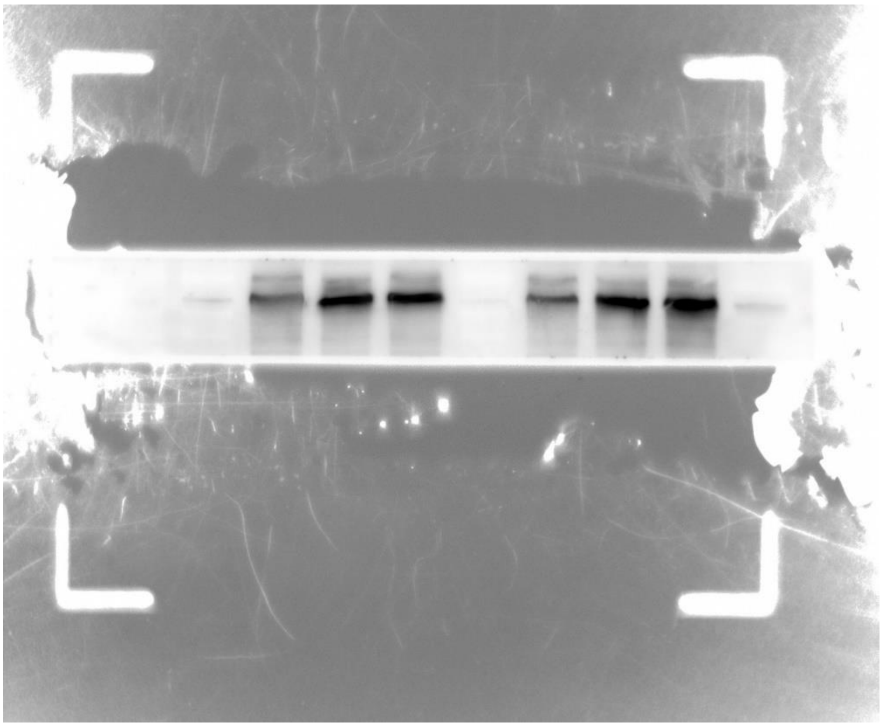

CRABP2

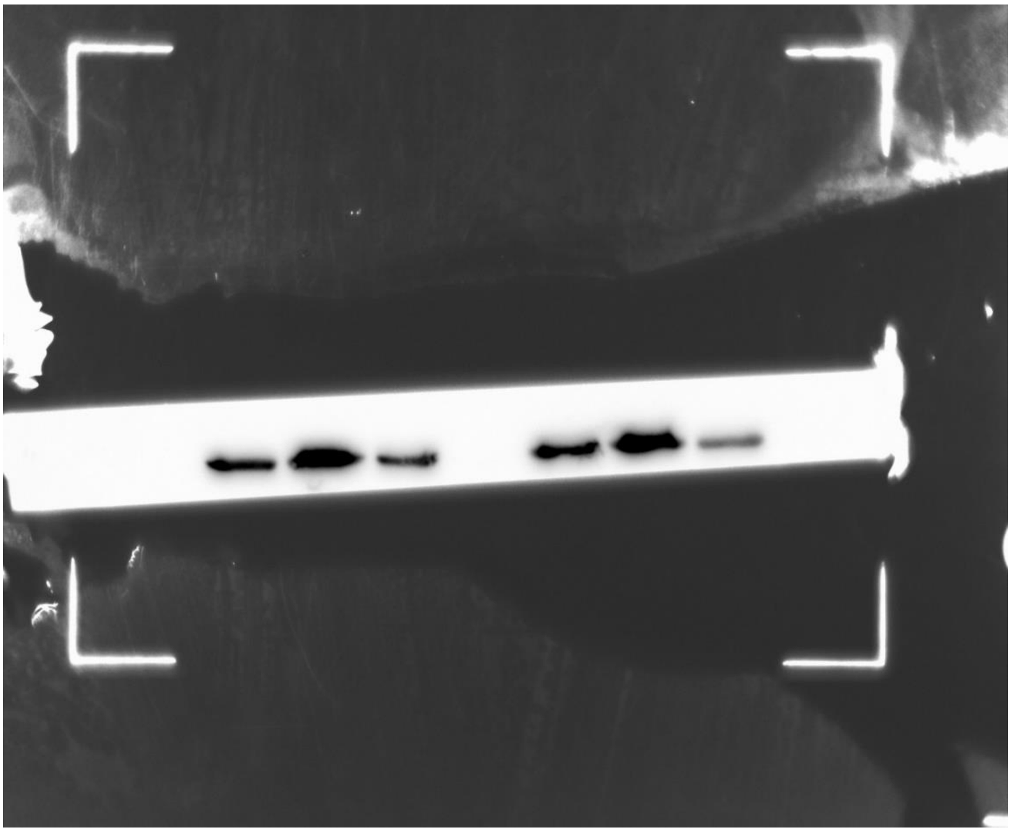

BAX

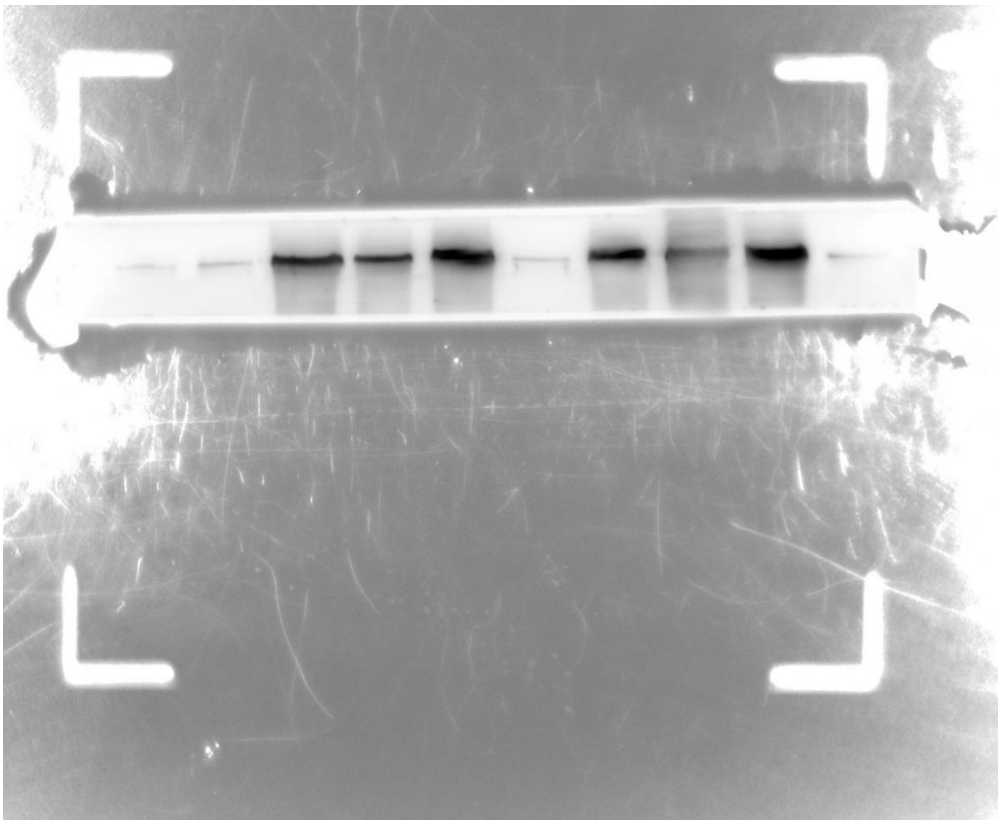

CAPASE3

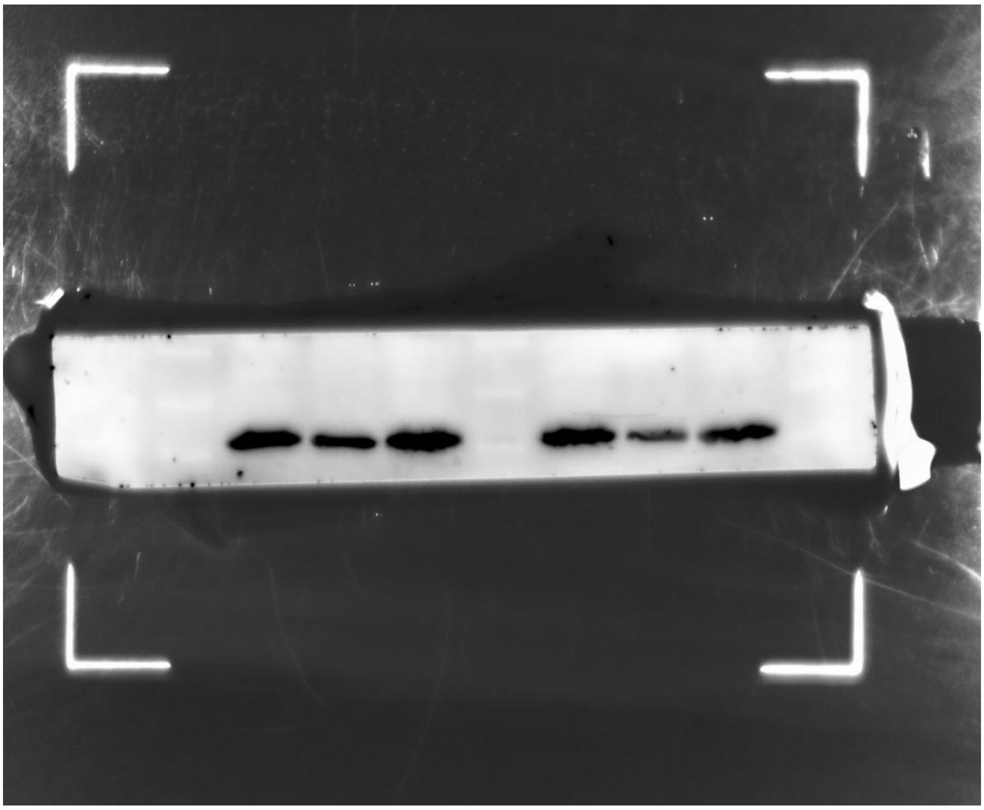

S6A RIGHT

GAPDH

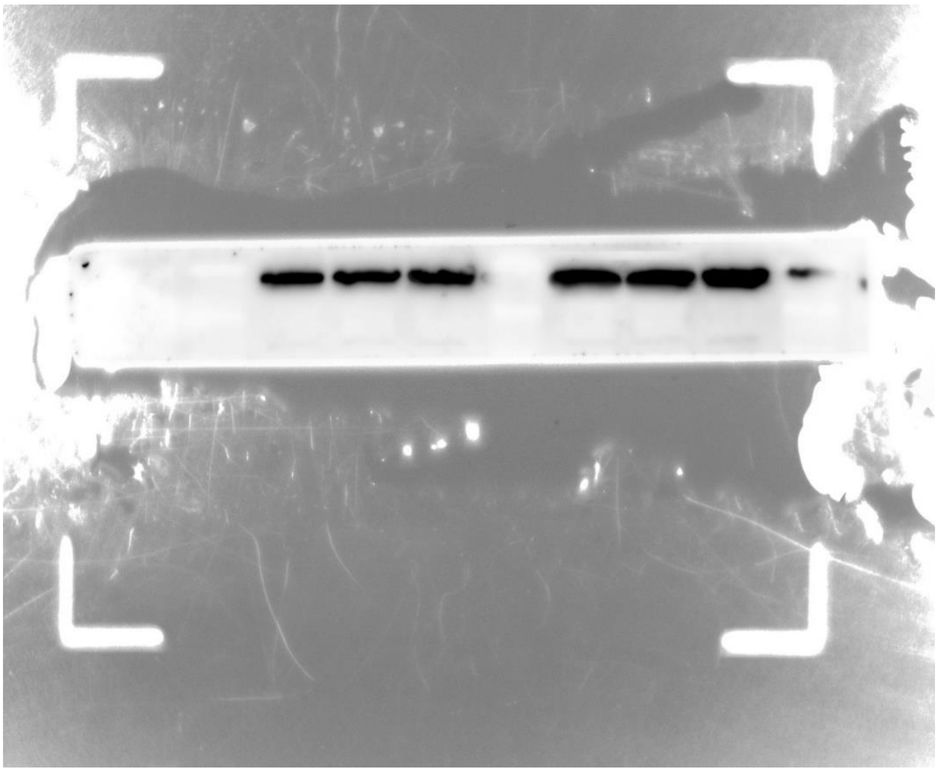

TET1

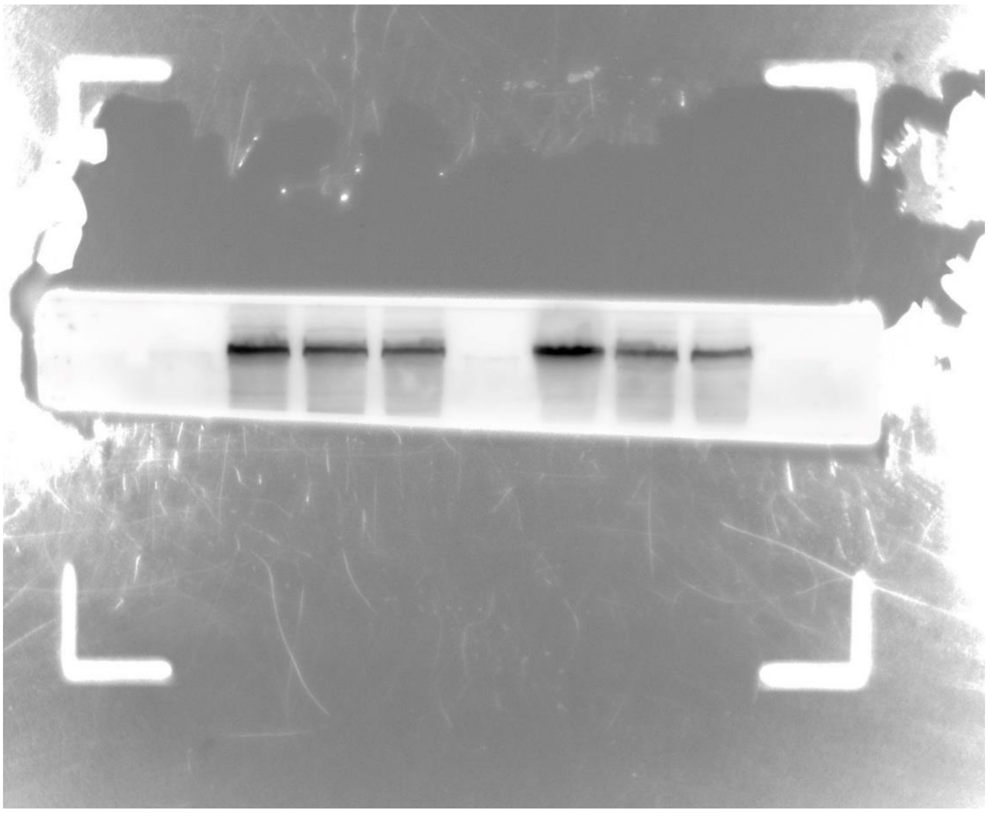

BAX

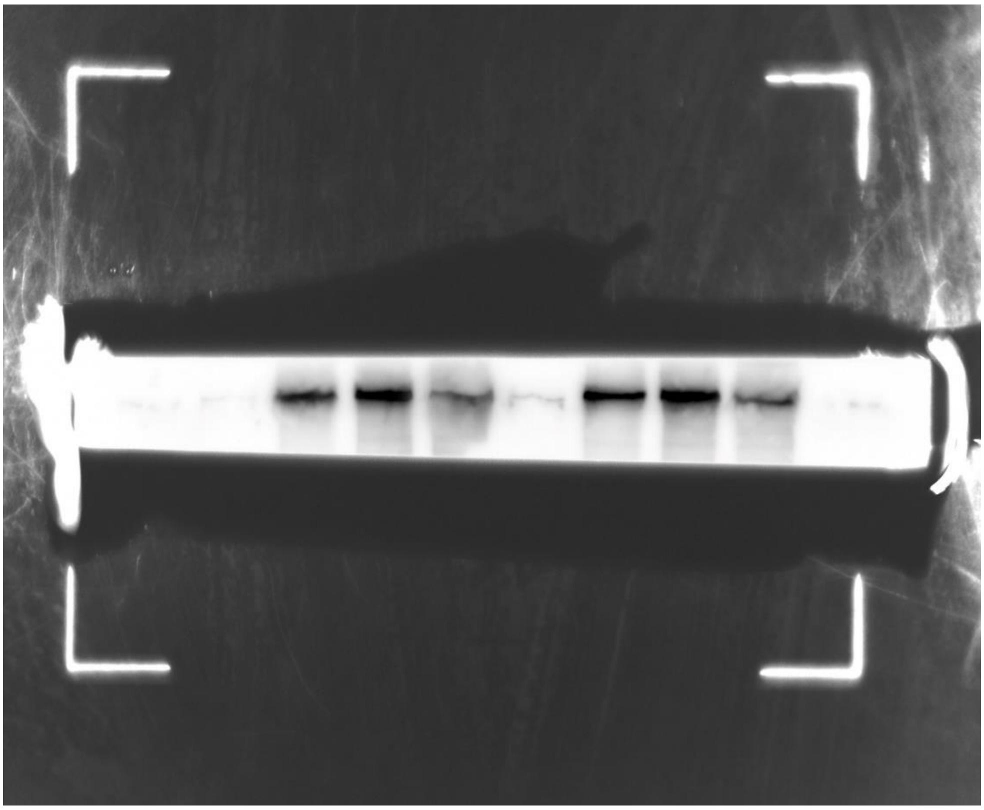

CRABP2

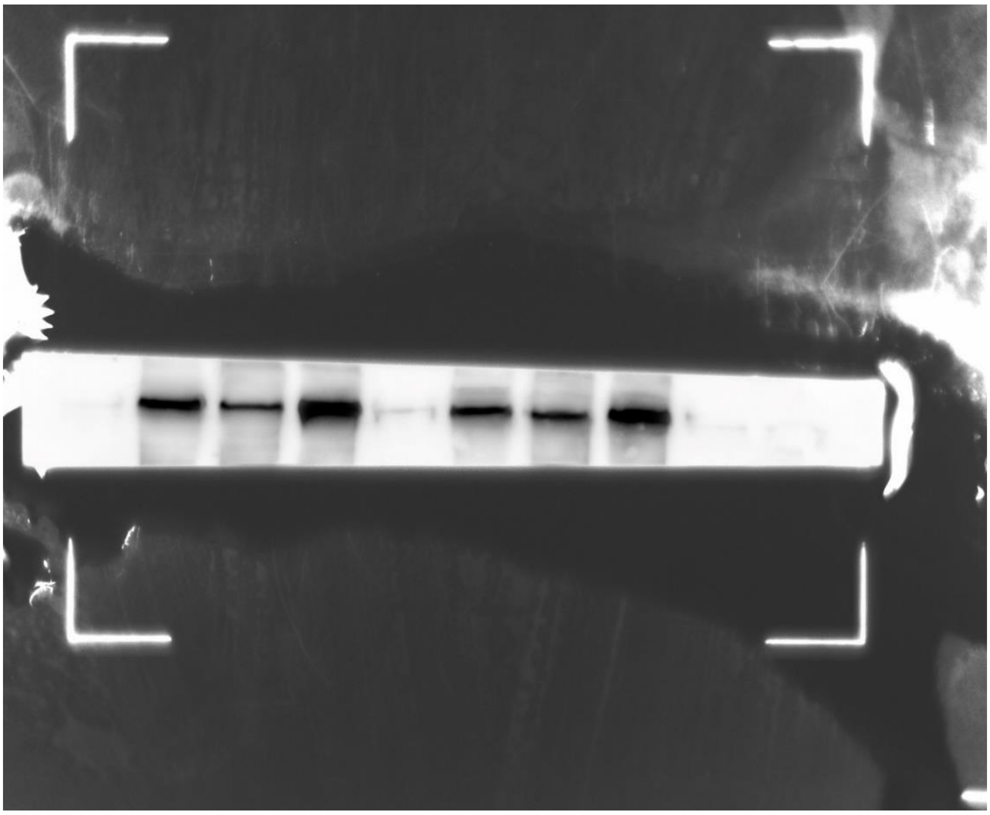

CASEPASE3

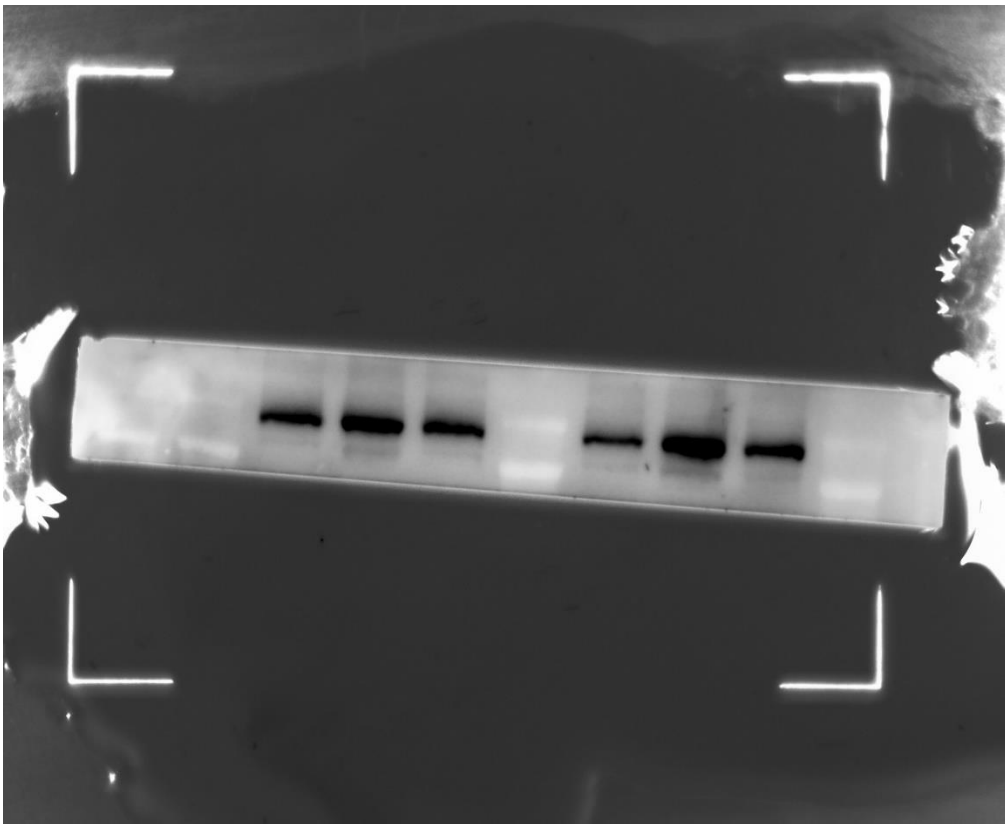

S6H UP

GAPDH

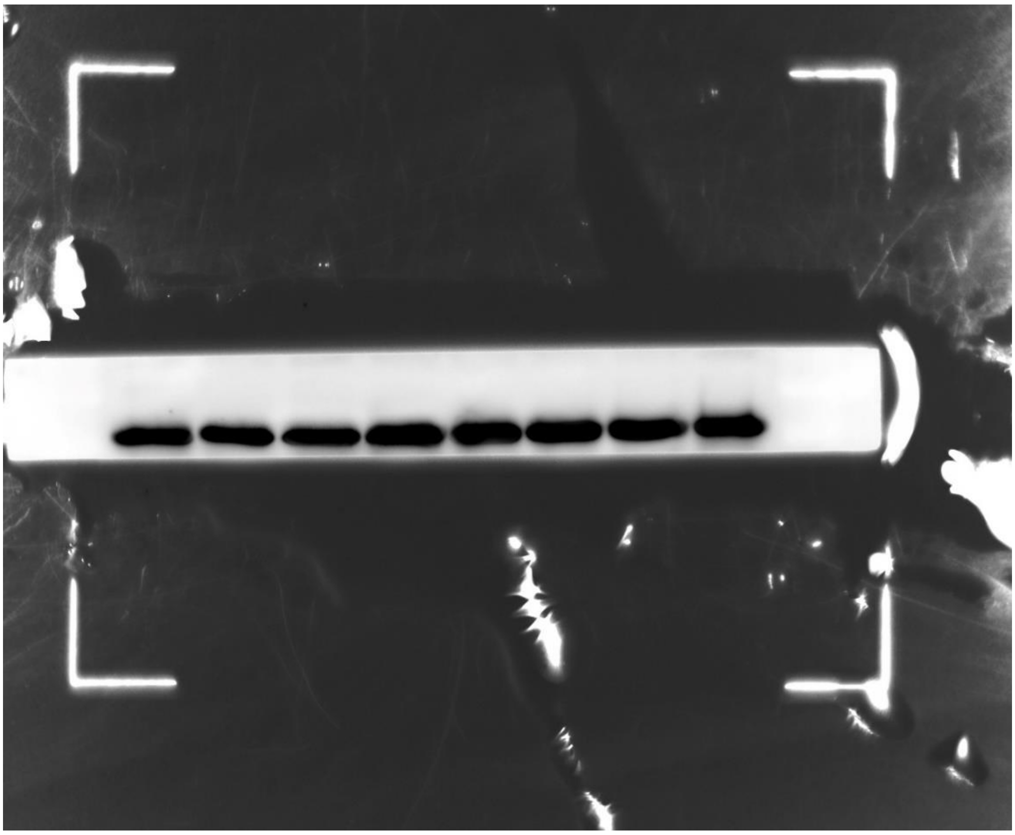

TET1

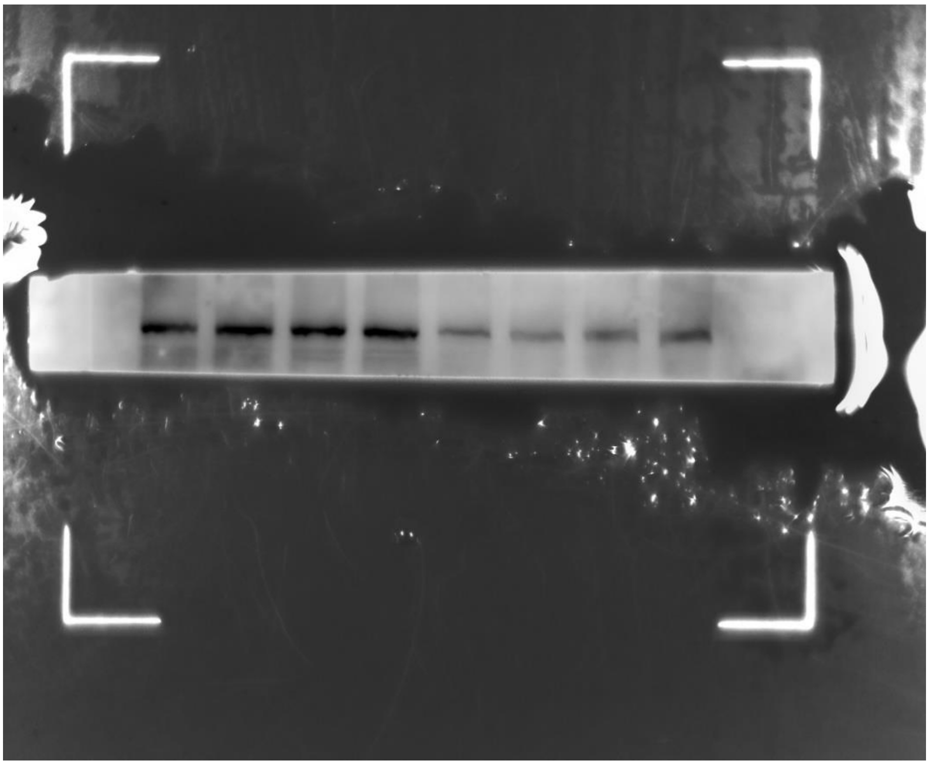

CRABP2

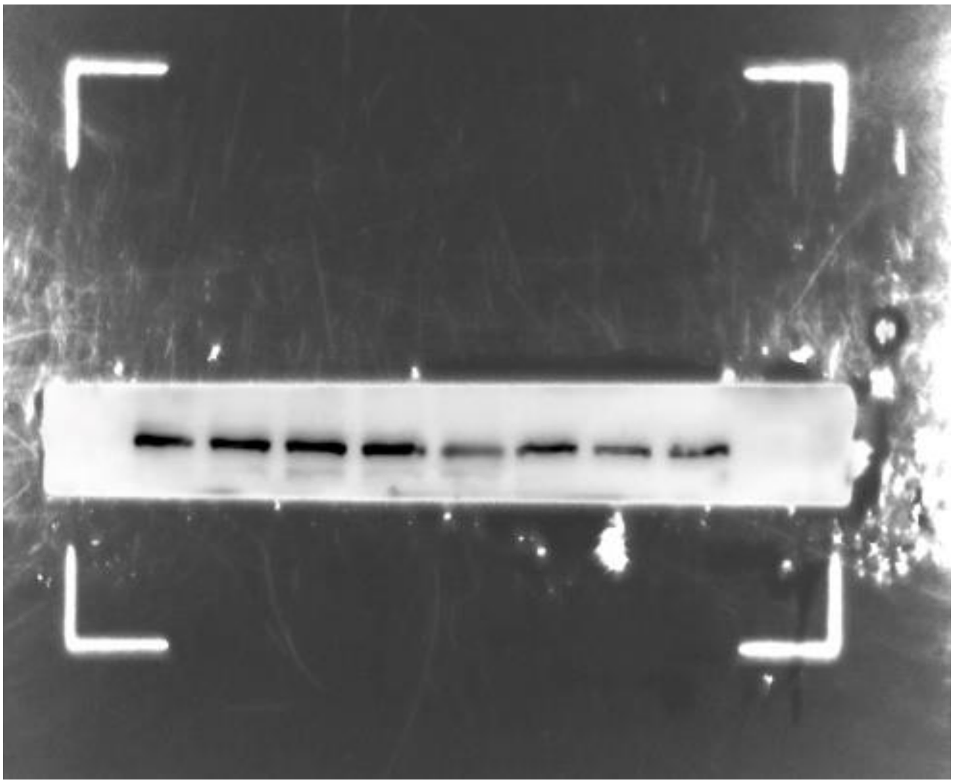

BAX

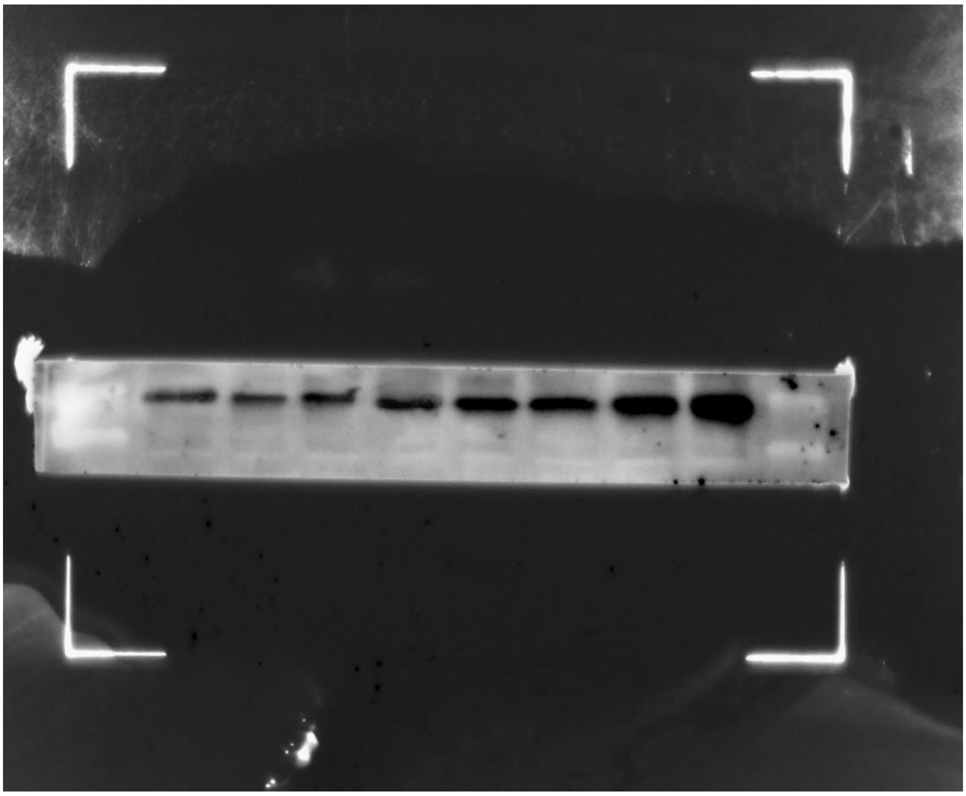

S6H DOWN

GAPDH

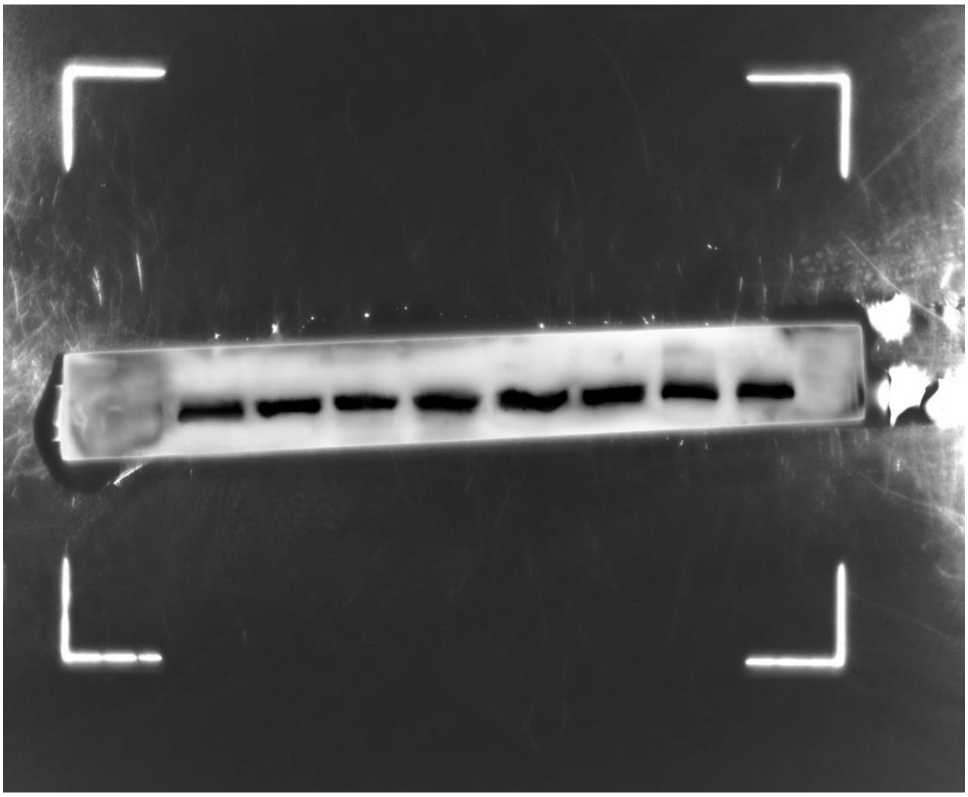

TET1

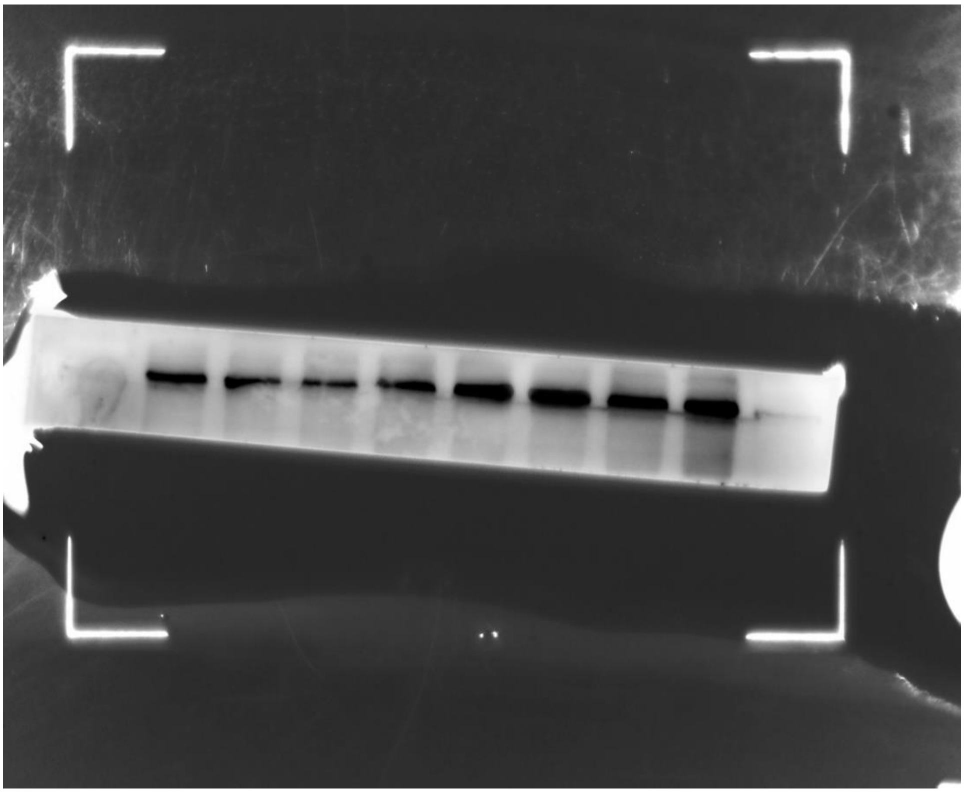

CRABP2

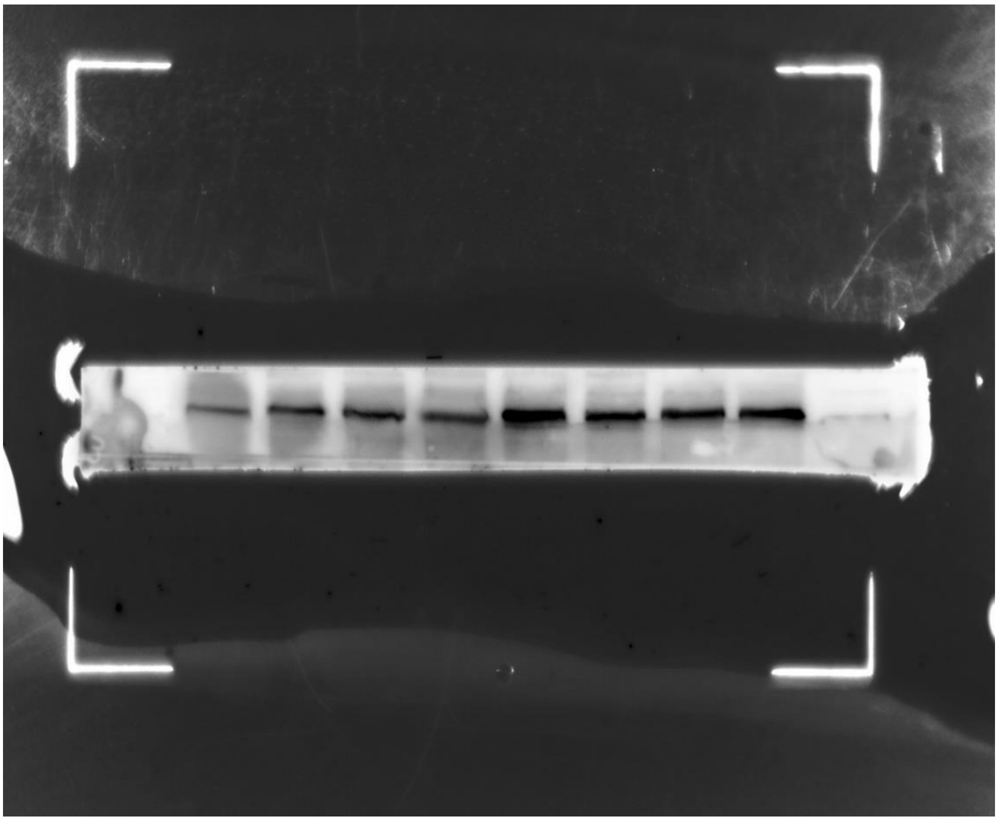

BAX

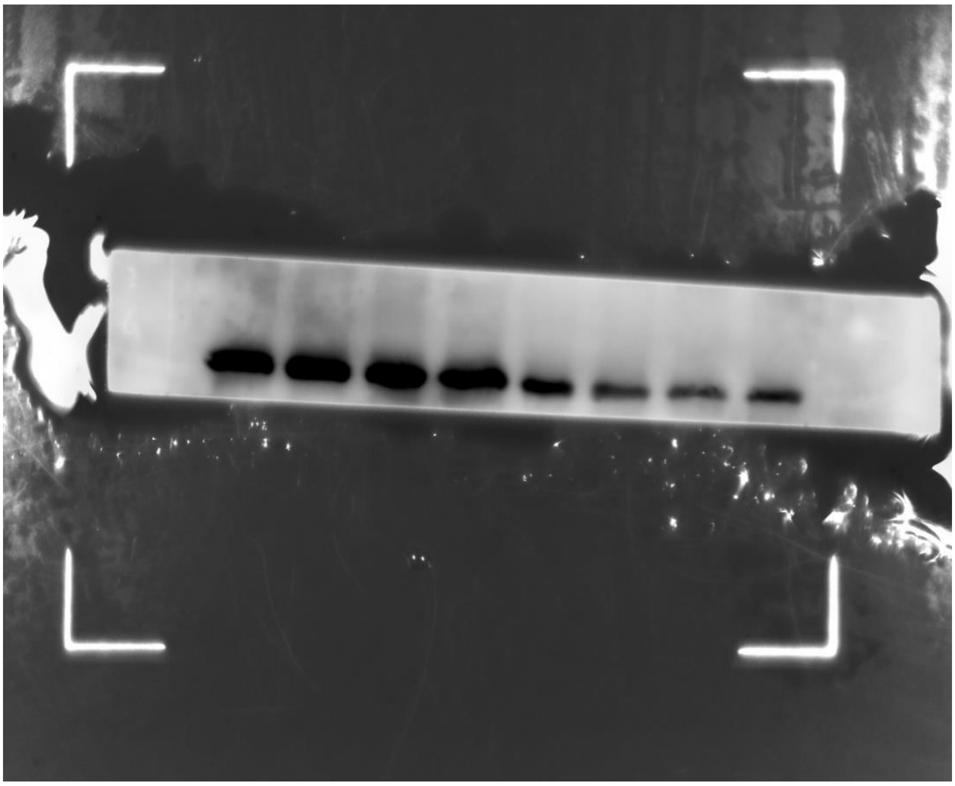

Supplement: Supplementary file 13 — The original gels of western blot [file 41419_2022_5299_MOESM13_ESM.pdf]
